# Synthesis, Properties, and Electrochemical Proton Reduction of a Homoleptic Tetrathiolato Ni-Site Model of [NiFe]-Hydrogenase

**DOI:** 10.1021/acs.inorgchem.5c03077

**Published:** 2025-09-05

**Authors:** Benjamin A. Yosen, Amelia G. Reid, Phan T. Truong, Tiara Hinton, Indranil Chakraborty, Marilyn M. Olmstead, Timothy L. Stemmler, Todd C. Harrop

**Affiliations:** † Department of Chemistry and Center for Metalloenzyme Studies, The University of Georgia, 302 East Campus Road, Athens, Georgia 30602, United States; ‡ Department of Chemistry and Biochemistry, 206928 Florida International University, Miami, Florida 33199, United States; § Department of Chemistry, 8789 University of California, Davis, California 95616, United States; ∥ Department of Pharmaceutical Sciences, 2954 Wayne State University, 259 Mack Ave, Detroit, Michigan 48201, United States

## Abstract

[NiFe]-hydrogenase enzymes process H_2_ at a nonplanar tetracysteinato-Ni site, the sole participator in proton binding/redox chemistry during turnover. With the objective of assessing whether a simple tetrahedral/tetrathiolato-Ni^2+^ core could promote H_2_ evolution reaction (HER), we synthesized (Et_4_N)_2_[Ni­(S-*p*-CF_3_–Ph)_4_] (**1**) employing *para*-trifluoromethylbenzenethiolate (^−^S-*p*-CF_3_–Ph) as a Ni-site analog of [NiFe]-hydrogenase. Spectroscopic measurements and X-ray crystallography confirm the distorted tetrahedral geometry of **1**. Dissolution of **1** results in partial thiolate dissociation and formation of *S*,*S*-bridged complexes such as (Et_4_N)_2_[Ni_2_(S-*p*-CF_3_–Ph)_6_] (**3**) among other ill-defined species. Dissociation is further accelerated in the presence of Brønsted acids, complicating the assessment of **1** for proton reduction. However, this dissociation/proton instability is suppressed in the presence of additional thiolate ligand to ensure tetrahedral/tetrathiolato **1** persists in solution. Electrochemical HER activity was evaluated by monitoring the current response of an MeCN solution of **1**/excess thiolate after sequential titration with a weak Brønsted acid (acetic acid). The results suggest that **1** is a modest electrocatalyst for the HER with a turnover frequency of 14.5 ± 3.6 s^–1^ and an overpotential of 0.72 ± 0.02 V. Control experiments and supplementary DFT computations indicate that **1**, or a species derived from **1**, is responsible for the HER and suggest an ECCE-type mechanism.

## Graphical abstract

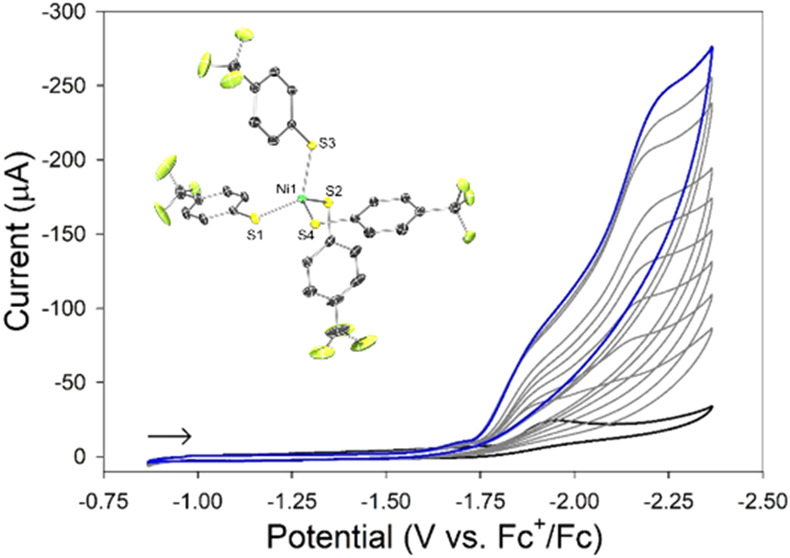

## Introduction

Hydrogen gas (H_2_) offers a potential solution for a clean, renewable, and energy-dense alternative to fossil fuels, a significant source of anthropogenic CO_2_ emissions.^1–3^ Steam methane reforming (SMR) is predominately used to produce H_2_; however, SMR is energy intensive and considered only a gray source of H_2_ due to formation of CO/CO_2_ as byproducts.^4^ Electrocatalytic water splitting offers a cleaner option for H_2_ production, but this method relies heavily on expensive noble metal catalysts, e.g., Pt and Pd, for proton reduction.^5,6^ In contrast, anaerobic bacteria have evolved H_2_ processing hydrogenase (H_2_ase) enzymes that utilize inexpensive/Earth-abundant metal-containing active sites ([M]-H_2_ase; M = NiFe, FeFe, or Fe) that provide inspiration for the development of cost-effective H_2_ evolving catalysts.^7,8^ [NiFe]-H_2_ase contains a redox-active cofactor that relies on Ni (Figure 1) to catalyze the reversible oxidation of H_2_ with turnover frequencies (TOFs) up to 6000 s^–1^ for H_2_ oxidation^9^ and ∼700 s^–1^ for H_2_ production,^10,11^ performed at or near the thermodynamic equilibrium potential *E°*(2H^+^/H_2_). Although Ni and Fe play important roles in [NiFe]-H_2_ase activity, only Ni participates in redox catalysis by alternating through putative Ni^3+/2+/1+^ oxidation states.^7^ In the resting Ni–SI_a_ state, the Ni center is in a distorted tetrahedral/seesaw geometry coordinated to four cysteinates (CysS), two of which bridge to a low-spin Fe^2+^ center.^5^ This S-rich anionic Ni­(SCys)_4_ core is proposed to depress the Ni^3+/2+^ couple, with a terminal CysS serving as a base/proton relay site (see Ni-R structure in Figure 1) to facilitate H_2_ evolution through the heterolytic coupling of the bridged hydride with a protonated CysSH.^5,12–15^

**Figure 1:**
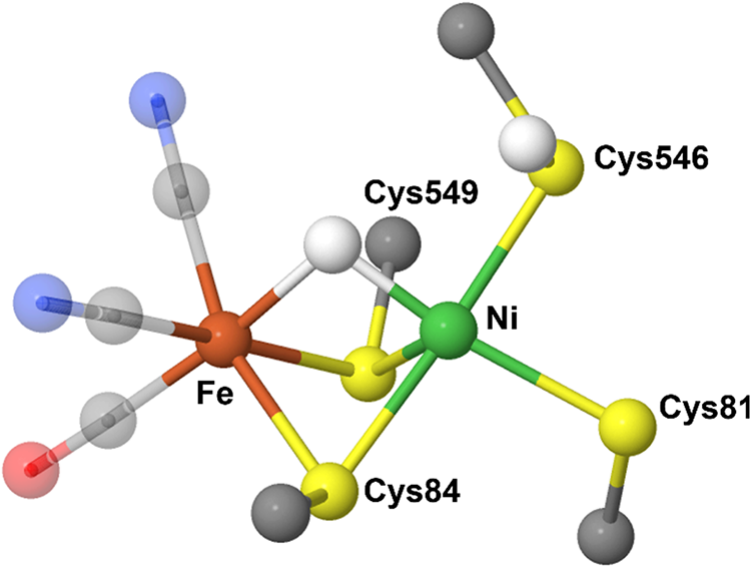
Active site of [NiFe]-H_2_ase from *Desulfovibrio vulgaris* Miyazaki F (PDB: 4U9H) in the Ni-R state depicting coordinating ligands to Ni (green) and Fe (red). Image created with Jmol.

Since the first reported X-ray absorption spectroscopic^16^ and X-ray diffraction^17^ data on [NiFe]-H_2_ase ∼30 years ago, synthetic chemists have developed various low molecular weight (LMW) model complexes to explore the underlying principles governing H_2_ase activity at the Ni­(SCys)_4_ active site.^18–27^ Several mononuclear complexes focus on the generation of highly active electrocatalysts for the H_2_ evolving reaction (HER) drawing inspiration from the structural and electronic features of the active site. For example, Ni complexes with soft P-rich coordination spheres have been reported to achieve TOFs up to 10^7^ s^–1^.^28^ Indeed, these Ni–P_4_ HER electrocatalysts have provided valuable information regarding factors that influence efficiency and turnover utilizing chelating diphosphine ligands. Their work highlights that selective positioning of pendant N-bases on the ligand serve as effective proton relays to increase HER rates (Chart 1).^28,29^ Drawing more parallels to the enzyme active site, the development of bimetallic mimics utilizing chelating diphosphine/dithiolate-P_2_S_2_ ^22,30^ and diamino/dithiolate-N_2_S_2_ ^31–35^ ligands to hold Ni in a square-planar geometry with thiolate bridges to other first-row metals (Chart 1) have also contributed to the development of key synthetic strategies and ligand design principles to generate active HER catalysts. For example, these models have shed light on the relationship between the electron richness of the catalyst and overpotential (η), the potential role of bridging thiolates as proton-binding sites, and the difficulty in centering redox activity solely on Ni.

**Chart 1:**
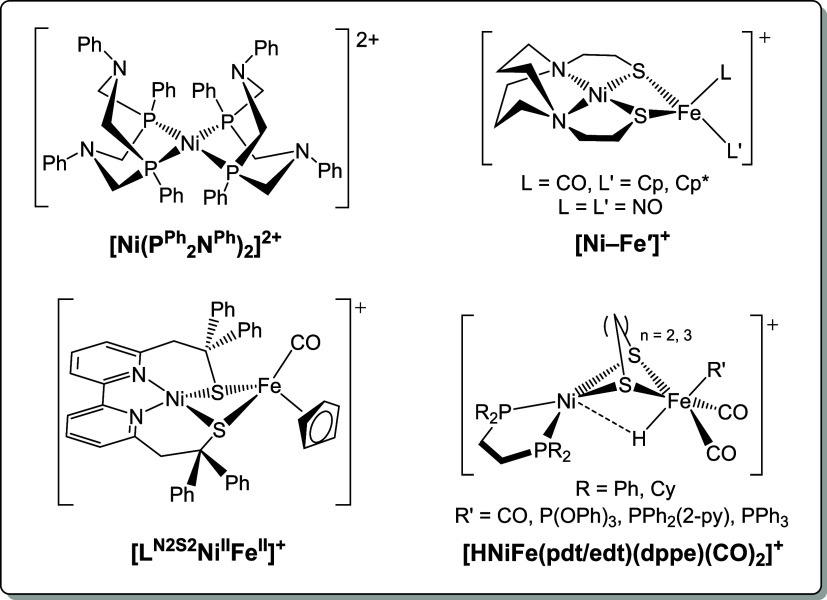
Structures of Select Functional Models of [NiFe]-H_2_ase Featuring Various Donor Types and Coordination Geometries About Ni^2+^

HER activity of Ni complexes in thiolate-only/nonplanar coordination geometries are underexplored. Indeed, metal–thiolate coordination chemistry is associated with many challenges^36–39^ including: (i) autoredox chemistry resulting in irreversible oxidation/deligation of the thiolate to generate disulfide with concomitant reduction of the metal center; (ii) instability under protic conditions; and (iii) the tendency to form higher nuclearity species, sometimes intractable, through bridging thiolates. These obstacles must be considered when constructing mononuclear Ni-thiolate systems as mimics of the Ni­(SCys)_4_ core in [NiFe]-H_2_ase. Two classes of mononuclear [Ni­(SR)_4_]^2–^ are known containing either chelating dithiolates or homoleptic complexes with monodentate thiolates (select examples shown in Chart 2).^18,40–51^ Maintaining a mononuclear species is highly dependent on solvent, identity of the thiolate ligand, and the metal/thiolate ratio employed during synthesis. In addition, these complexes are generally in planar/distorted planar coordination geometries except for homoleptic Ni complexes featuring aryl-thiolates that almost exclusively exist in distorted tetrahedral geometries much like that in [NiFe]-H_2_ase (Chart 2). The proposed driving force being the favorable π-overlap of the out-of-plane S-pπ orbitals (with respect to the benzene ring^52^) and the benzene-pπ system when the plane defined by Ni–S–C is coplanar with the phenyl ring.^49,53,54^ Homoleptic Ni-aryl-thiolate, and even Ni-thiolate complexes with bidentate dithiolates,^43,44,50^ are proposed to exist in equilibrium with S-bridged species (via thiolate dissociation) as reflected by their concentration-dependent NMR, UV–vis, and solution-state magnetic moment (μ_eff_) values.^40,55,56^ Although it has been postulated that the equilibrium is due to thiolate dissociation,^55,56^ it is unclear what the identity of this species is. Two proposed species are solvent-bound [Ni­(SR)_3_(solv)]^−^,^56^ or some higher order species, such as *S*,*S*-bridged dimers [(RS)_2_Ni­(μ-SR)_2_Ni­(SR)_2_]^2–^.^57^ Extensive studies for the latter have not been performed. For example, addition of H_2_O or EtOH to MeCN solutions of [Ni­(SPh)_4_]^2–^ leads to precipitation of intractable polymers, denoted as [Ni­(SPh)_2_]_∞_.^42^ However, there is no strong evidence to suggest the identity of this species nor the mechanism of its formation. Issues associated with LMW Ni-thiolate complexes are nullified in [NiFe]-H_2_ase and other Ni-enzymes due to the protection enabled by the protein scaffold. For example, Shafaat and co-workers utilize the protein scaffold in rubredoxin (Rd), a small electron-transfer protein whose native metal is Fe, to create a tetrahedral Ni­(SCys)_4_ site (NiRd) that exhibits H_2_ase activity (TOF up to 100 s^–1^, η = 540 mV).^58,59^ This work suggests that tetrahedral/tetrathiolato-Ni complexes, barring autoredox and protic stability/solubility issues, should also be functional models for the HER. However, the electrocatalytic HER activity of homoleptic Ni-aryl-thiolate complexes has not been reported. This absence is most likely due to their ill-defined solution speciation, instability in protic conditions, and irreversible redox events.

**Chart 2:**
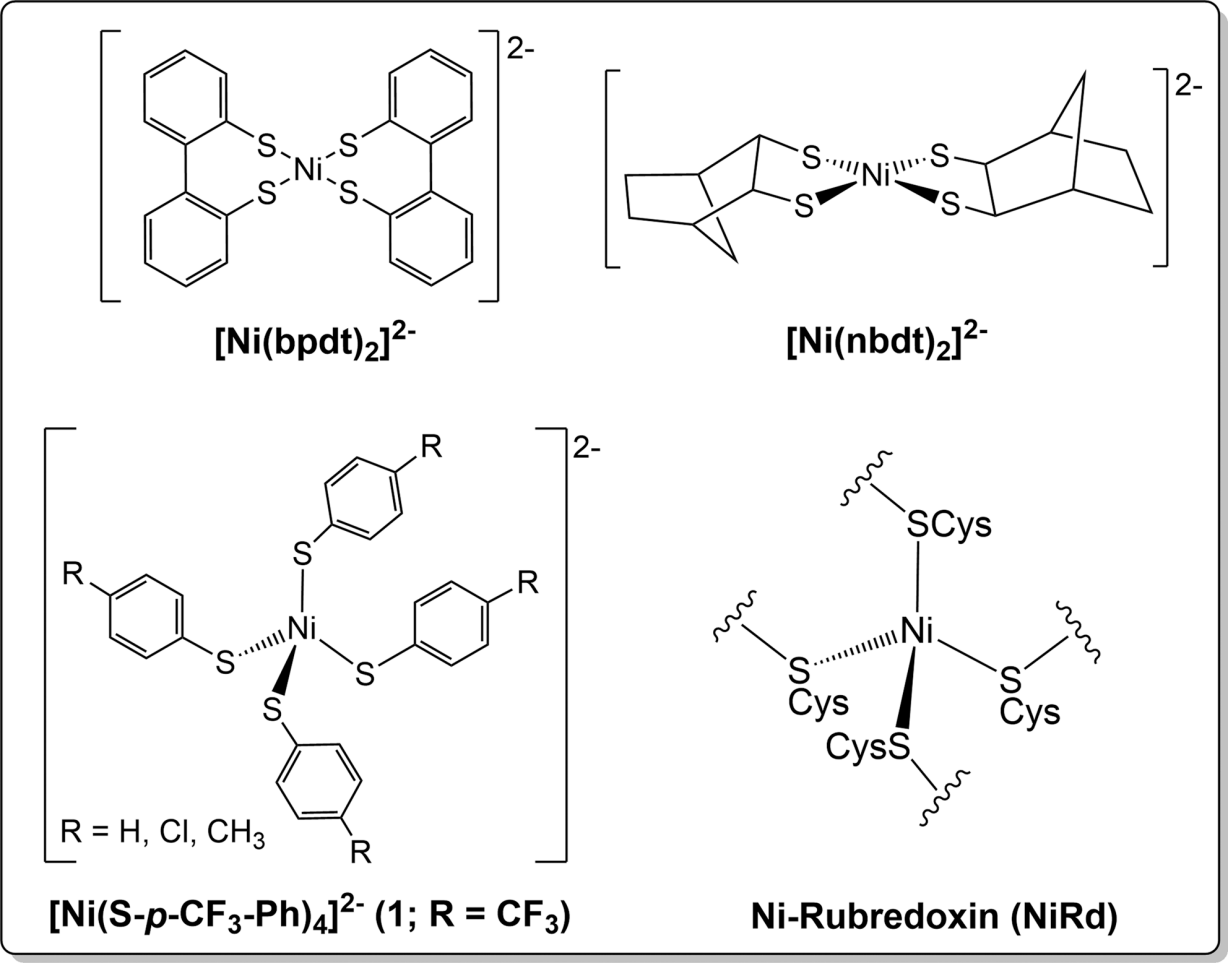
Structures of Thiolate-rich Monomeric Ni^2+^ Complexes

The objective of this work was to synthesize, characterize, and evaluate the capability of a tetrahedral Ni^2+^ complex in a thiolate-only ligand environment, a discrete model of the tetracysteinato-Ni site in [NiFe]-H_2_ase, for electrochemical HER. With this goal in mind, we employed *para*-trifluoromethylbenzene thiolate to afford Ni-site model (Et_4_N)_2_[Ni­(S-*p*-CF_3_–Ph)_4_] (**1**) (Chart 2). As stated above, LMW homoleptic complexes featuring aryl-thiolates almost exclusively afford tetrahedral Ni^2+^ species; however, and despite their structural similarity to the Ni site in [NiFe]-H_2_ase, none have been assessed for H_2_ evolution. This absence is likely due to the noted speciation and protic solvent instability of such complexes.^42,55,56^ We thus posited that using the strong electron-withdrawing *p*-CF_3_ group (σ_p_ = 0.54 ^60^) in **1** would diminish the nucleophilicity of the thiolate to prevent or depress such speciation while also providing an additional spectroscopic handle to monitor through ^19^F NMR. Reported herein is the synthesis, structural/spectroscopic/electrochemical properties, solution speciation, and electrochemical HER activity of the homoleptic tetrathiolato Ni^2+^ complex **1** and its redox-inert Zn^2+^ analogue (Et_4_N)_2_[Zn­(S-*p*-CF_3_–Ph)_4_] (**2**). Despite incorporation of the *p*-CF_3_ group, complex **1** affords thiolate-bridged species such as a proposed *S*,*S*-bridged dimer (Et_4_N)_2_[(*p*-CF_3_–PhS)_2_Ni­(μ-S-*p*-CF_3_–Ph)_2_Ni­(S-*p*-CF_3_–Ph)_2_] (**3**) even when dissolved in aprotic MeCN due to thiolate dissociation. Unsurprisingly, dimer/oligomerization is further accelerated in the presence of Brønsted acids. However, the equilibrium can be shifted back to mononuclear/tetrahedral complex **1** by adding excess thiolate equivalents to freshly dissolved solutions of **1**. With thiolate in excess, complex **1** was measured for HER in MeCN using acetic acid (AcOH; p*K*_a_ = 23.5 ^61^) as the proton source to result in a modest electrocatalyst. Electrochemical control measurements with free thiolate (Et_4_N)­(S-*p*-CF_3_–Ph) and Zn^2+^ complex **2** support that **1**, or a species derived from **1**, is responsible for the observed electrocatalysis. Kinetic isotope measurements with AcOD-*d*_4_ and supporting theoretical computations from density functional theory (DFT) suggest that **1** operates via an ECCE (E = electrochemical; C = chemical step) type mechanism.

## Results/Discussion

### Synthesis

Preparation of the tetrahedral/tetrathiolato Ni^2+^ complex (Et_4_N)_2_[Ni­(S-*p*-CF_3_–Ph)_4_] (**1**) was adopted from Mascharak.^55^ This method employs slightly high thiolate-to-Ni ratios and polar aprotic solvents to prevent formation of intractable thiolato-bridged polymers (vide infra).^42,55^ Dropwise addition of a blue MeCN slurry of (Et_4_N)_2_[NiCl_4_] into an MeCN solution containing upward of 4.5 mol-equiv of the tetraethylammonium (Et_4_N^+^) salt of *para*-trifluoromethylbenzenethiolate (Et_4_N)­(S-*p*-CF_3_–Ph) produced a homogeneous dark maroon-red solution indicative of **1**.^42,55,62^ After workup, **1** was isolated as a dark-red powder in 77% yield. The corresponding tetrahedral/tetrathiolato Zn^2+^ complex (Et_4_N)_2_[Zn­(S-*p*-CF_3_–Ph)_4_] (**2**) was isolated in 90% yield using near identical conditions.^63^ Synthesis of the *S*,*S*-bridged planar complex (Et_4_N)_2_[(*p*-CF_3_–PhS)_2_Ni­(μ-S-*p*-CF_3_–Ph)_2_Ni­(S-*p*-CF_3_–Ph)_2_] (**3**) denoted as (Et_4_N)_2_[Ni_2_(S-*p*-CF_3_–Ph)_6_], a potential product during reactions with H^+^, was also attempted. Combining complex **1** with a stoichiometric amount of a methylating (Me_3_OBF_4_) or an oxidizing (ferrocenium hexafluorophosphate = FcPF_6_) agent, reagents that result in modification of a coordinated thiolate to methyl-thioether or disulfide, respectively, were employed to generate **3**.^39,64^ While **3** was produced (see Experimental section in the Supporting Information = SI), both syntheses afford product mixtures that were difficult to separate, thus isolation of **3** was not successful. The Ni-nitrosyl complex (Et_4_N)_2_[Ni­(S-*p*-CF_3_–Ph)_3_(NO)] (**4**) was also synthesized, a proxy for a putative Ni–H intermediate, in 53% yield by addition of Ph_3_CSNO^65^ to **1** (1:1) in MeCN. Standard characterization from UV–vis, FTIR, ^1^H/^19^F NMR, ESI-MS, XAS, magnetic moment, X-ray diffraction, and CHN elemental analysis support the formulation of the complexes (see below).

### X-ray Crystallography, X-ray Absorption Spectroscopy, and Electronic Structures

X-ray diffraction of single crystals of **1**-**2** confirmed their monomeric nature and distorted tetrahedral coordination geometries. Thermal ellipsoid plots of **1**-**2** and all relevant structural data are provided in Figure 2 and Tables 1 and S1–S2 of the SI (both structures contain disorder in one or more of the *p*-CF_3_–Ph rings; see Figures S32–S33). The crystal structures of homoleptic **1** and **2** display four aryl-thiolato ligands coordinating M^2+^ in approximate tetrahedral geometry with average M–S bond distances of 2.282 and 2.350 Å for **1** and **2**, respectively (Table 1). Additional structural details, obtained by calculating the tetrahedral distortion parameter τ_4_ (1 for a perfect tetrahedron; 0 for a perfect square-plane^66^), reveal that the distortion for **1** (τ_4_ = 0.79), indicating primarily, albeit distorted, tetrahedral is expectedly greater than in **2** (τ_4_ = 1.00). Complex **2** crystallizes in the tetragonal *I*4_1_/*a* space group, with 1/4th of the complex within the asymmetric unit and thus it is strictly tetrahedral. Apart from the predictable M–SPh elongation and more ideal tetrahedral geometry for the *d* ^10^ Zn^2+^ complex, **1** and **2** are largely isostructural and approach *D*_*2d*_ point symmetry (ignoring the *p*-CF_3_ groups). Comparing **1** to previously reported homoleptic tetrahedral/tetrathiolato complexes [Ni­(S-*p*-X-Ph)_4_]^2–^ (X = H, Cl) suggest similar structures throughout with only subtle differences (Table 1).^41,42,48,55^ While these distances are similar among homoleptic [Ni­(SR)_4_]^2–^, they are significantly elongated when comparing to neutral tetrahedral (*S* = 1) Ni^2+^ complexes with tripodal supporting ligands featuring a single Ni-SPh bond (avg = 2.21 Å), likely a consequence of overall complex charge.^67–69^ There is a modest trend with respect to the *p*-X Hammett parameter, Ni–SPh distance and τ_4_, i.e., electron-withdrawing *p*-X yields shorter Ni–SPh bonds and the more distorted tetrahedron. Furthermore, three of the four aryl-thiolate rings are nearly coplanar with the plane defined by Ni–S–C (Table S2), a consistent structural feature in other reported tetra-aryl-thiolato-Ni^2+^ complexes, one proposed driving force for tetrahedral coordination.^41,49,53,55^

**Figure 2:**
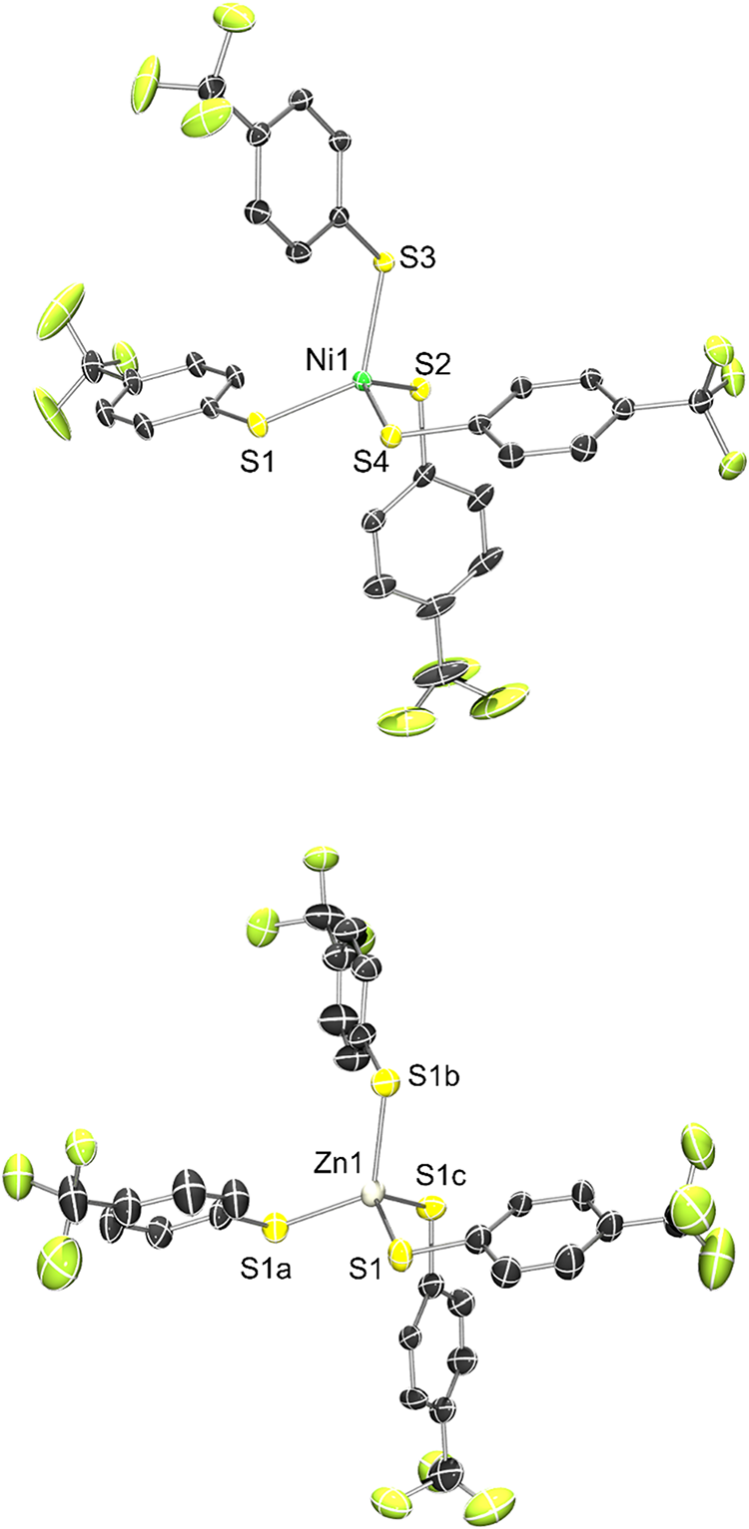
X-ray structures of the dianions of **1** (*top*) and **2** (*bottom*) at 50% thermal probability ellipsoids with the atom labeling scheme. H atoms and Et_4_N^+^ counterions are omitted for clarity.

**Table 1:** Select Bond Distances (Å) and *τ*_4_ Values^65^ from XRD and DFT of Select Tetrathiolato Ni^2+^ and Zn^2+^ Complexes^a^

complex	Ni–S (Å)	Ni–S_avg_ (Å)	*τ*_4_	ref
**Tetrahedral Ni/Zn-thiolates**				
(Et_4_N)_2_[Ni(S-*p*-CF_3_–Ph)_4_] (**1**)	2.296, 2.290, 2.275, 2.266	2.282	0.79	TW
DFT-optimized **1** (*S* = 1, MeCN)	2.331, 2.349, 2.312, 2.336	2.332	0.82	TW
(Et_4_N)_2_[Ni(S-*p*-Cl-Ph)_4_]	2.279, 2.281, 2.293, 2.269	2.281	0.81	^55^
(Et_4_N)_2_[Ni(SPh)_4_]^b^	2.287, 2.306, 2.296, 2.279	2.292	0.83	^42^
(Et_4_N)_2_[Ni(SPh)_4_]^c^	2.258, 2.331, 2.293, 2.302	2.296	0.81	^48^
(Ph_4_P)_2_[Ni(SPh)_4_]	2.303, 2.289, 2.272, 2.287	2.288	0.83	^41^
(Et_4_N)_2_[Ni(S-*o*-Ph-Ph)_4_]	2.288	2.288	0.90	^49^
NiRd^d^	2.254, 2.381, 2.477, 2.134	2.304	0.87	^70^
DFT-optimized NiRd^e^	2.345, 2.282, 2.332, 2.287	2.312	0.84	^71^
(Et_4_N)_2_[Zn(S-*p*-CF_3_–Ph)_4_] (**2**)	2.350	2.350	1.00	TW
DFT-optimized **2** (*S* = 0, Gas)	2.392, 2.403, 2.385, 2.392	2.393	0.95	TW
(Ph_4_P)_2_[Zn(SPh)_4_]	2.362, 2.363, 2.329, 2.357	2.352	0.87	^41^
**Planar Ni-thiolates**				
DFT-optimized **1** (*S* = 0, MeCN)	2.251, 2.252, 2.266, 2.254	2.255	0.08	TW
[Ni(HPyS)_4_](NO_3_)_2_	2.213, 2.215, 2.214, 2.204	2.212	0.00	^51^
[Li(DMF)(MeOH)]_2_[Ni(nbdt)_2_]	2.185, 2.182	2.184	0.00	^45^
(Ph_4_P)_2_[Ni(edt)_2_]^f^	2.201, 2.188	2.195	0.00	^44^
(Ph_4_P)_2_[Ni_2_(edt)_3_]^f^	2.211^br^, 2.221^br^, 2.174^t^, 2.161^t^ 2.158^br^, 2.166^br^, 2.194^t^, 2.181^t^	2.183	0.11	^43^
(Et_4_N)_2_[Ni(bpdt)_2_]	2.214, 2.219, 2.221, 2.209	2.216	0.04	^50^
(Et_4_N)_2_[Ni_2_(bpdt)_3_]^f^	2.201^br^, 2.191^br^, 2.204^t^, 2.203^t^ 2.233^br^, 2.224^br^ 2.195^t^, 2.192^t^	2.205	0.03, 0.15	^50^
(Et_4_N)_2_[Ni_2_(S-*p*-Cl-Ph)_6_]^f^	2.212^br^, 2.220^br^, 2.204^t^, 2.219^t^	2.214	0.07	^57^
DFT-optimized **3** (*S* = 0)^f^	2.246^br^, 2.247^br^, 2.211^t^, 2.243^t^	2.237	0.18	TW

a Abbreviations: S-*o*-Ph-Ph = 2-phenylbenzenethiolate; Rd = rubredoxin; HPyS = prydinium-2-thiolate; bpdt = biphenyl-2,2′-dithiolate; edt = ethane-1,2,-dithiolate; nbdt = norborane-1,2-dithiolate. TW = this work.

b Crystallized in the monoclinic space group.

c Crystallized in the orthorhombic space group.

d From *Clostridium pasteurianum*, pdb = 1R0J, see ref 69.

e Values taken from the “full” model of NiRd, see ref 70.

f Ni–S distances defined as terminal (t) or bridging (br).

Additional structural information from Ni K-edge X-ray absorption spectroscopic (XAS) and magnetic moment (μ_eff_) measurements support the tetrahedral geometry of **1** in the solid-state. X-ray absorption near edge spectroscopic (XANES) analysis reveals a relatively intense pre-edge feature at 8331 eV (area = 0.165 eV^2^) corresponding to a 1s → 3d transition with a rising edge 1s → 4p feature at 8345 eV, both features are consistent with tetrahedral coordination at Ni^2+^.^72^ Extended X-ray absorption fine structure (EXAFS) for **1** (Figure 3) indicates coordination of four S-scatterers at a Ni–S­(avg) distance of 2.274 Å (Table S3), in-line with that obtained from XRD (2.282 Å) (Table 1; vide supra). In contrast, square-planar Ni^2+^ complexes generally display shorter Ni-SR distances, 2.17–2.21 Å,^18,43–46,73,74^ (Table 1) and weak intensity/symmetry-forbidden 1s → 3d transitions in XAS.^75^ The solid-state μ_eff_ was determined to be 3.47 μ_B_ (spin-only μ_eff_ = 2.83 μ_B_) and further supports tetrahedral (*S* = 1) coordination. Collectively, all solid-state measurements are on-par with other monodentate tetrathiolato-Ni^2+^ complexes containing aryl-thiolate ligands that, with one exception (i.e., [Ni­(HPyS)_4_]^2+^, HPyS^–^ = N-protonated form of 2-pyridinethiolate; see Table 1), afford tetrahedral geometries. In contrast, [Ni­(SR)_4_]^2–^ complexes with mono/multidentate alkyl-thiolate or multidentate aryl-thiolate donors, are invariably square-planar (Table 1).^43–46,50,73^

**Figure 3:**
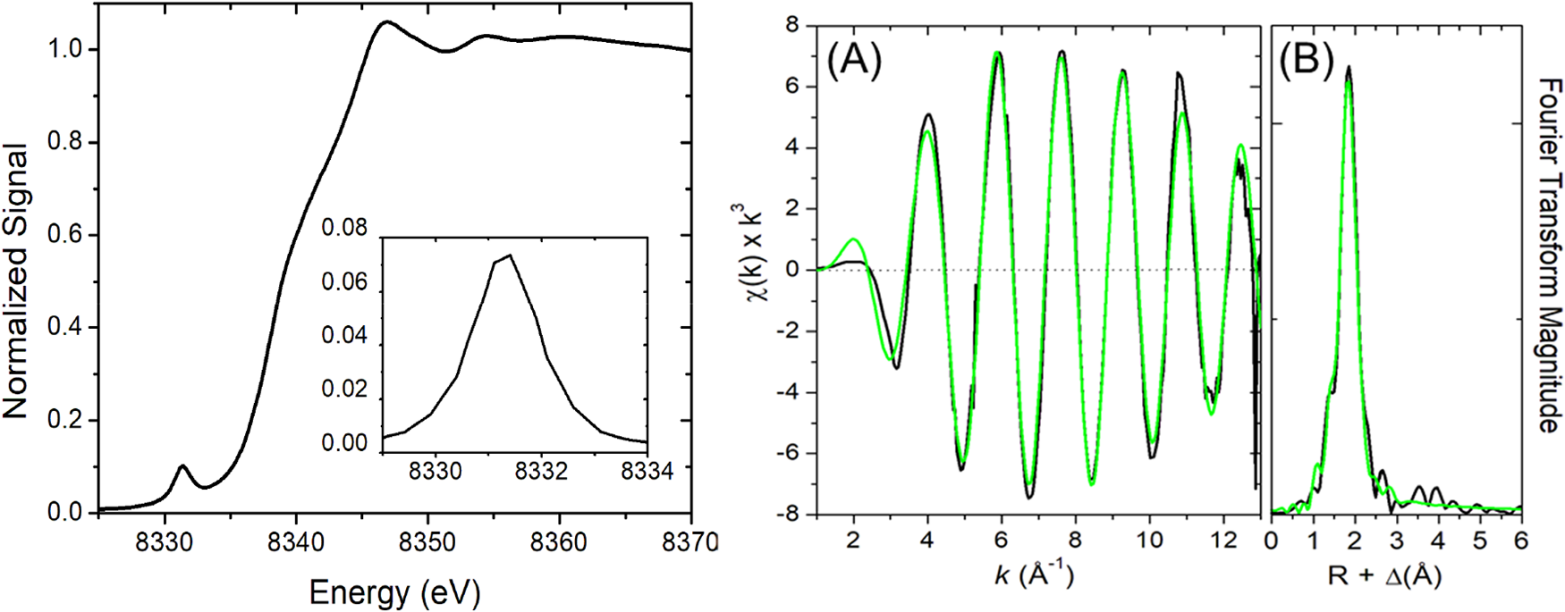
*Left*: Ni K-edge XANES for solid **1** diluted with BN (see Experimental section in the SI). *Inset*: baseline subtracted expansion of pre-edge feature for **1**, offset for clarity (coding same as XANES). *Right*: Raw *k* ^3^-weighted EXAFS data (panel A; black) and phase shifted Fourier transform EXAFS (panel B; black) of **1** with best-fit simulation (green).

DFT computations (B3LYP/def2-TZVPP) were employed to gain insight on electronic structure. Input structures for the geometry optimization of **1** and **2** were taken from the crystallographic coordinates. For *S*,*S*-bridged dimeric complex **3**, initial coordinates were obtained from the crystal structure of the *p*-Cl-SPh analogue, *p*-Cl replaced with *p*-CF_3_, published by Maroney.^57^ The DFT-optimized structure of **1** (Figure S34) largely resembles that determined by XRD with a modest overestimation of the Ni-thiolate distances (Δ: +0.04 Å) from XRD and reasonable approximation of the distorted tetrahedral geometry (τ_4_ = 0.75). Overestimation of the Zn-thiolate bonds from XRD (Δ: +0.04 Å) in geometry-optimized **2** (Figure S36) was also observed and appears to be a common outcome when employing hybrid functionals such as B3LYP.^76^ The electronic structure of **1** indicates a lowest unoccupied spin-down MO (β-LUMO) that is mostly Ni-based (63%, Figure 4). A large Ni contribution in the β-LUMO, coupled with the Löwdin spin density of **1** (76% on Ni, Figure 4), suggests a Ni-centered redox process (vide infra). Expectedly, square-planar/dimeric **3** is structurally/electronically quite different from **1**. Shorter Ni-thiolate bonds (∼2.2 Å) and significant Ni-*d*π and S-*p*π contributions to the π* HOMO (23% Ni, 51% S) and HOMO–1 (15% Ni, 57% S; 0.05 eV from HOMO), suggest more covalent character (Figure S37).^39,77,78^ Apart from a modest lengthening of the Ni-thiolate bonds and slight distortion in τ_4_ (0.18; see Table 1), DFT-computed **3** is comparable to the structurally characterized *p*-Cl analogue. These small deviations may reflect the stronger electron-withdrawing effect, and hence weaker nucleophilicity, of the *p*-CF_3_-substituted thiolate in **3**. The LUMO in **3** is a σ*-based MO with major contributions from Ni-*d*_*x2‑y2*_ (42%) and S-*p*σ orbitals (21%).

**Figure 4:**
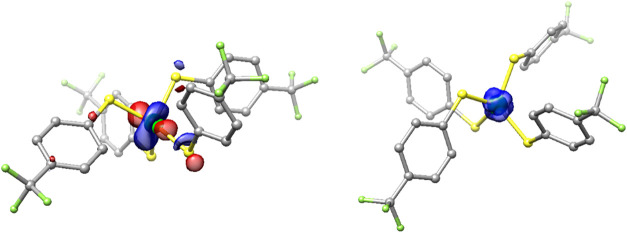
DFT-generated isosurface plots of the β-LUMO of **1** (*left*) and spin-density plot of **1** (*right*) (blue denotes positive spin). Color scheme: Ni, green; S, yellow; F, green-yellow; C, gray. H atoms are omitted for clarity.

### Solution-State Characterization

Solution studies of related homoleptic tetrahedral/tetra-aryl-thiolato Ni^2+^ complexes indicate thiolate loss is facile and results in putative and transient [Ni­(SPh)_3_(L)]^−^ (L = donor solvent such as DMSO) planar complexes.^56^ Thiolate dissociation is further accelerated in the presence of protic solvents (vide infra) resulting in intractable thiolato-bridged complexes labeled as [Ni­(SPh)_2_]_∞_.^42^ An initial sign of the speciation of **1** was observed in its UV–vis spectrum, which exhibits a nearly featureless and flat profile in MeCN (Figure 5). Contrastingly, (Ph_4_P)_2_[Ni­(SPh)_4_] displays two closely spaced charge-transfer (CT) bands at 460 and 510 nm (ε ∼ 2500 M^–1^ cm^–1^) with *d*-*d* transitions at 676 and 725 nm (ε ∼ 300 M^–1^ cm^–1^) in DMF/toluene (1:1) (Table 2).^79^ Notably, this spectrum was acquired in the presence of an additional 10 mol-equiv of PhS^–^. Examination of the literature indicates near identical features for [Ni­(S-*p*-X-Ph)_4_]^2–^ (X = H, Cl) when spectra are recorded in polar aprotic solutions (MeCN, DMSO) containing ***excess thiolate***.^56,80^ The reported UV–vis of (Et_4_N)_2_[Ni­(S-*p*-Cl-Ph)_4_] in MeCN, *in the absence of excess thiolate*, matches the featureless spectrum reported here for **1**.^55,57^ Clearly, dissolution of [Ni­(SPh)_4_]^2–^ complexes, even in aprotic solvents, results in partial thiolate dissociation. Additionally, thiolate dissociation, and in some cases further thiolate reactivity, is also observed in tetrahedral/charge neutral [(κ^3^-L)­Ni-SAr] (L = substituted tris­(pyrazolyl)­borate, phenyltris­((*tert*-butylthio)-methyl)­borate tripodal ligands) complexes and may be a general property for Ni-thiolates in a tetrahedral ligand field.^67,68^ Adding 20 mol-equiv of (Et_4_N)­(S-*p*-CF_3_–Ph) to MeCN solutions of **1** converts the featureless spectrum to one that reproduces the tetrahedral complex, i.e., CT bands at 465 and 508 nm (ε = 3400 and 3800 M^–1^ cm^–1^, respectively) and *d*-*d* transitions at 680 and 720 nm (ε ∼ 600 M^–1^ cm^–1^) (Figure 5; Table 2). The origin of these transitions align with those proposed for (PPh_4_)_2_[Ni­(SPh)_4_][Bibr ref79] and NiRd.^71,79^ For example, the UV–vis for NiRd is comparable with two intense bands at 360 and 450 nm attributed to CysS-to-Ni^2+^ LMCT transitions, and *d*-*d* bands at 670 and 720 nm (Table 2).^71,79,80^ Time-dependent DFT (TD-DFT) of **1** reproduce the experimental spectrum quite well and supports the proposed assignments (Figure 5), i.e., two CT bands (S-*p*π-to-Ni-*d*π) at 463 and 518 nm followed by *d*-*d* transitions between 640 and 700 nm. These absorption features thus represent general UV–vis signatures for tetrahedral/tetra-aryl-thiolato-Ni^2+^ complexes (Table 2).^79,80^ In contrast, the experimental and computed UV–vis of dimer/planar **3** comprises of an intense LMCT centered at 380 nm (λ_sh_: 415 nm) and is largely featureless in the visible region with a broad band centered at 650 nm composed of several *d*-*d* bands (Figures S21 and S43).

**Figure 5:**
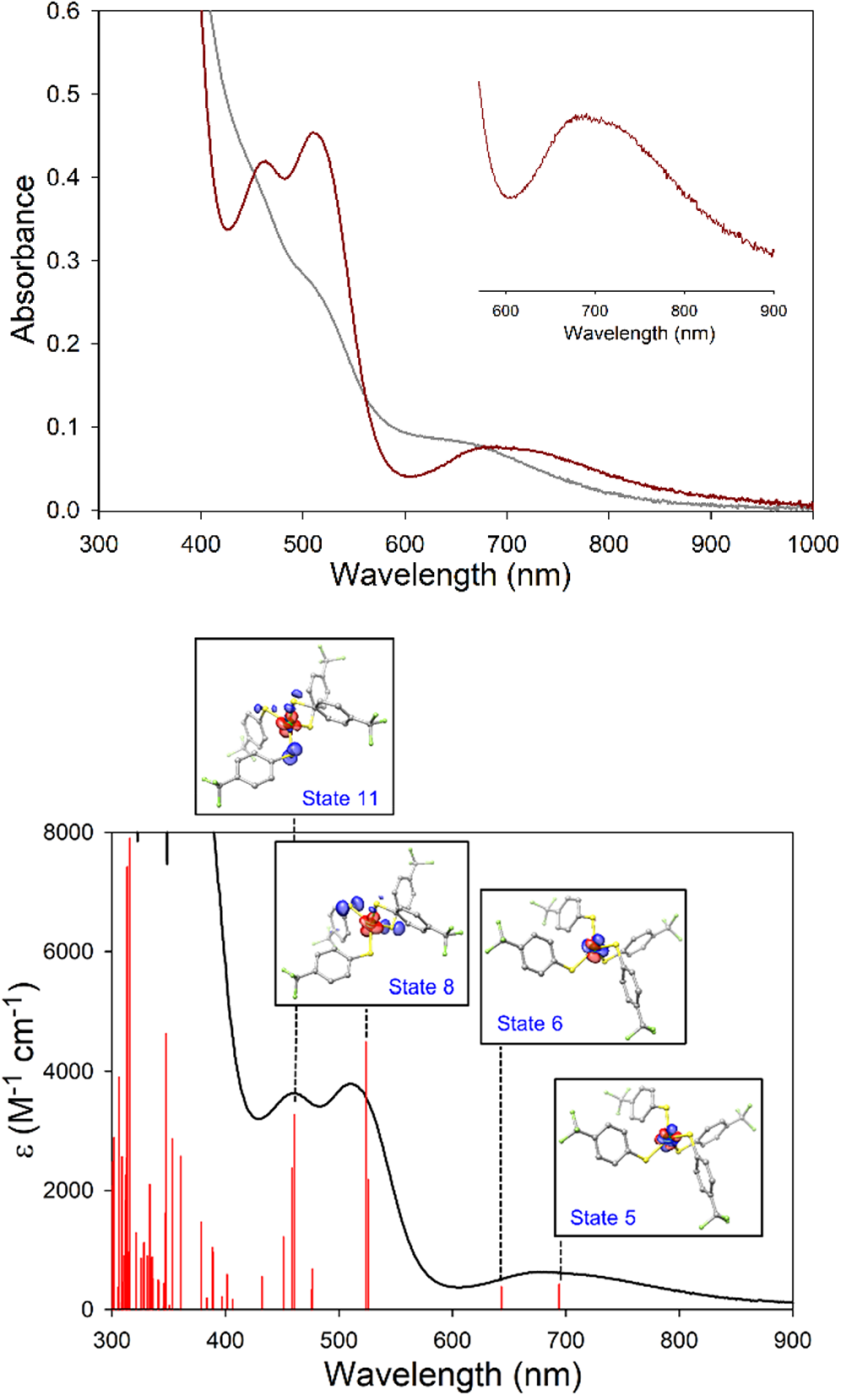
*Top*: UV–vis spectrum of an MeCN solution of **1** (0.13 mM; gray trace) and after the addition of 20 mol-equiv (Et_4_N)­(S-*p*-CF_3_–Ph) (red trace). *Inset*: zoom-in of the ligand-field bands. *Bottom:* Experimental UV–vis of **1**/20 mol-equiv (Et_4_N)­(S-*p*-CF_3_–Ph) in MeCN (black) with TD-DFT calculated optical transitions (red sticks). *Insets*: Electron difference density plots for select transitions: blue and red lobes reflect loss and gain of electron density, respectively.

**Table 2:** Select UV–vis and Electrochemical Properties (Reported or Converted^81^ vs. Fc^+^/Fc) of Tetrathiolato Ni^2+^ Complexes^a^

complex	λ (nm) (ε (M^–1^ cm^–1^))	*E*_ox_ (V)^b^	*E*_red_ (V)^b^	ref
(Et_4_N)_2_[Ni(S-*p*-CF_3_–Ph)_4_] (**1**)	720 (580), 680 (600), 508 (3800), 465 (3400)^c^ ^,^ ^d^	–0.20, −0.08^c^	–1.94^c^ ^,^ ^d^	TW
Computed **1**	694 (640), 643 (650), 518 (7200), 463 (8000)^c^	NR	–2.69 (r, Ni^2+/1+^)	TW
(Et_4_N)_2_[Ni(S-*p*-Cl-Ph)_4_]	660–680 (NR), 300–500 (NR)^c^	NR	NR	^57^
(Et_4_N)_2_[Ni(SPh)_4_]	660 (780 sh), 430 (6000 sh)^c^	–0.44^c^, −0.57^e^	NR	^42,82^
(Ph_4_P)_2_[Ni(SPh)_4_]	725 (∼300), 676 (∼300), 510 (∼2500), 460 (∼2400)^f^	NR	NR	^79^
NiRd^g^	720 (460), 670 (385), 450 (3200), 360 (5460)^h^	–0.20^i^	NR	^71,79^
Computed NiRd	732 (640), 661 (70), 455 (1800), 351 (4700)^j^	–0.52^k^	–2.70^k^	^59,71^
[Ni(HPyS)_4_](NO_3_)_2_	634 (30), 371 (32,700), 291 (82,500)^c^	NR	–1.78^l^	^51^
(Ph_4_P)_2_[Ni(edt)_2_]	NR	–1.06^c^ (r, Ni^3+/2+^)	NR	^43^
(^n^Bu_4_N)_2_[Ni_3_(pdt)_5_]	NR	NR	–2.20, −2.40^m^	^83^
Ni_6_(PET)_12_	∼550 (br), ∼420, ∼340^m^	∼+0.4^m^	∼−2.4^m^	^84^

a Abbreviations: sh = shoulder; Rd = rubredoxin; HPyS = pyridinium-2-thiolate; edt = ethane-1,2,-dithiolate; pdt = propane-1,2,-dithiolate; PET = 2-phenylethanethiolate’ br = broad. TW = this work; NR = Not reported.

b Unless indicated (r = reversible), all potentials represent irreversible couples;

c MeCN.

d Values obtained in the presence of excess thiolate (50 mol-equiv (Et_4_N)­(S-*p*-CF_3_–Ph) for **1**; 10 mol-equiv KSPh for (Ph_4_P)_2_[Ni­(SPh)_4_]).

e DMSO.

f DMF/Toluene 50% (v/v).

g NiRd from *Desulfovibrio gigas*.

h pH 7.4 Tris-HCl buffer, with 50% (v/v) ethylene glycol.

i 100 mM KCl, pH 4.5 acetate buffer.

j Values taken from the vertical transition energies of the “full” model of NiRd, see ref 58.

k H_2_O.

l DMF.

m THF.

^1^H NMR spectra of [Ni­(SPh)_4_]^2–^-derived complexes including **1**,^41,42,55,56^ exhibit paramagnetically shifted peaks (Figure 6). Most indicative of tetrahedral **1** is the peak at ∼20 ppm, assigned to the *meta*-H in [S-*p*-CF_3_–SPh]^−^ (Figure 6).^55,56^ The *ortho*-H resonance is obscured by Et_4_N^+^ peaks, but appears at ∼1 ppm. A group of low intensity peaks at ∼7 ppm corresponding to a diamagnetic (*S* = 0) species is also present in all preparations of **1**. The ^19^F NMR signal in **1** serves as an additional spectroscopic handle and confirms both *S* = 0 (δ = −62.0 ppm; compared to δ = −61.7 ppm for Zn^2+^ complex **2**, Figure S18), and *S* = 1 (δ = −19.0 ppm) complexes in solution (Figure 7). The ^19^F peak (Figure 7) corresponding to tetrahedral **1** account for ∼50% of the Ni species ([**1**] = 2.0 mM in CD_3_CN; see Figure S46); however, adding 20 mol-equiv of (Et_4_N)­(S-*p*-CF_3_–Ph) pushes the speciation back to tetrahedral/monomer **1** (∼85%; Figure S46). Comparing the ^1^H NMR of (Et_4_N)­(S-*p*-CF_3_–Ph) to **1** eliminates free thiolate as the diamagnetic contributor (Figure S2). Comparable peaks at ∼7 ppm are also described in the ^1^H NMR spectra of previously reported [Ni­(S-*p*-X-Ph)_4_]^2–^ (X = H, Cl, Me) and [Ni­(S-*m*-Cl-Ph)_4_]^2–^ complexes.^55^ In contrast to **1**, the ^1^H NMR of **2** displays sharp/correctly integrated proton resonances, although the analogous Zn^2+^ complex [Zn­(SPh)_4_]^2–^ exhibits broad NMR signals that suggests thiolate dissociation in the simpler unsubstituted benzenethiolate-ligated system, previously attributed to the overall dianionic charge of the complex.^85^ The attenuated basicity of *p*-CF_3_–PhSH (p*K*_a_: 8.4, computed in DMSO;^86^ cf. p*K*_a_ (*p*-Br-PhSH): 9.0 in DMSO^87^) versus PhSH (p*K*_a_: 10.3 in DMSO)^87^ likely alleviates electron density at the metal to suppress ligand dissociation by minimizing Zn-*d*/S-*p* electron/electron repulsion given their identical overall charge.

**Figure 6:**
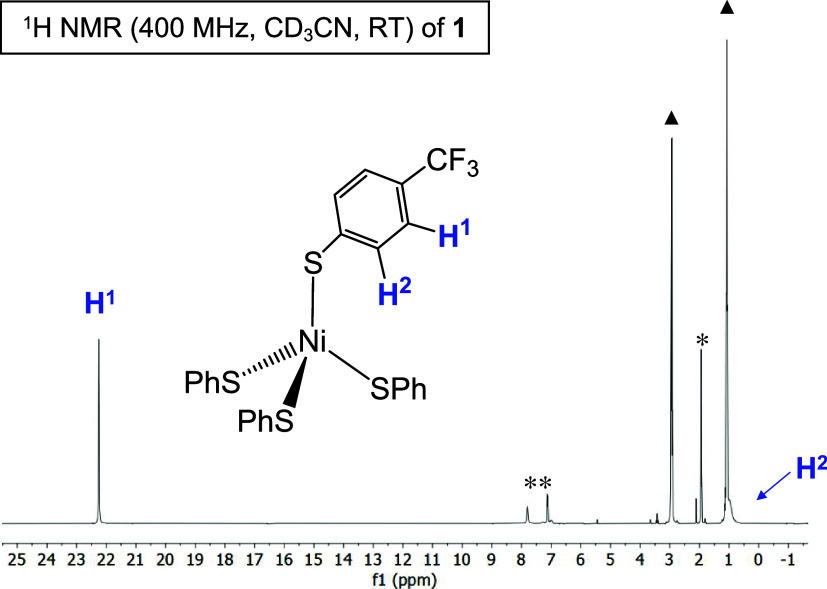
^1^H NMR spectrum of complex **1** in CD_3_CN at RT (δ vs residual protio solvent signal*). Peaks associated with *S* = 0 species indicated with **. *Note*: SPh = *p*-CF_3_–SPh.▲ = Et_4_N^+^ peaks.

**Figure 7:**
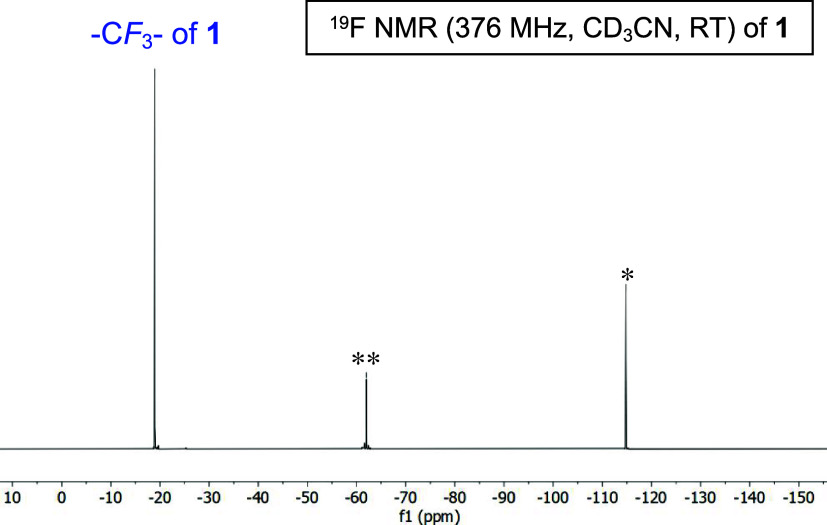
^19^F NMR spectrum of complex **1** in CD_3_CN at RT (δ vs CFCl_3_). Peak at −114.81 ppm is from PhF (internal standard) as indicated with *. Peak associated with *S* = 0 species indicated with **.

Solution ESI-MS and μ_eff_ analyses further confirm the speciation described above. Unlike the solid-state μ_eff_ value of 3.47 μ_B_, measurements for **1** in CD_3_CN ([**1**] = 10.3 mM) yield μ_eff_ of 2.75 μ_B_, characteristic of [Ni­(S-*p*-X-Ph)_4_]^2–^ complexes (μ_eff_ = 2.7–2.9 μ_B_ in CD_3_CN; 2.5–3.0 μ_B_ in DMSO-*d*_6_).^40,55^ Addition of 20 mol-equiv thiolate to solutions of **1** increase μ_eff_ to 3.05 μ_B_, suggesting more tetrahedral species. ESI-MS(−) analysis of MeCN solutions of **1** indicate charged complexes symptomatic of *S*,*S*-bridged species, e.g., [Ni_2_(S-*p*-CF_3_–Ph)_5_]^−^ (*m*/*z* = 1001.0; dimer **3** - thiolate; Figures S11–S12). However, various ligand substitution and redox reactions can happen in coordination complexes examined by ESI-MS.^88^ Recording ESI-MS(−) of **1** with 20 mol-equiv (Et_4_N)­(S-*p*-CF_3_–Ph) resulted in disappearance of signals assigned to the bridged complex and enhancement of peaks associated with **1**, e.g., (Et_4_N)­[Ni­(S-*p*-CF_3_–Ph)_4_]^−^ (*m*/*z* = 896.1) and [Ni­(S-*p*-CF_3_–Ph)_3_]^−^ (*m*/*z* = 589.1) (Figure S13). Collectively, all solution measurements suggest **1** partially converts to a diamagnetic complex upon dissolution, likely thiolate-bridged complexes such as [(RS)_2_Ni­(μ-SR)_2_Ni­(SR)_2_]^2–^ (dianion of **3**, R = S-*p*-CF_3_–Ph); however (depending on concentration), the bulk speciation leans toward the paramagnetic tetrahedral Ni^2+^ species in the presence of excess thiolate.

To confirm that bridged complexes such as **3** contribute to the ‘diamagnetic’ NMR signals, we attempted to synthesize **3** by several routes, e.g., chemical oxidation, methylation, and protonation (vide supra), all of which should afford the suspected *S*,*S*-bridged dimer. However, each synthesis resulted in a spectroscopically identical mix of compounds where no single complex was readily isolated, although one species was observed that we tentatively identify as **3**. Ultimately, NMR analysis points to diamagnetic complexes (Figures S22 and S24), one of which indeed has signals identical to those observed in solutions of **1** (Figure S23). While this result does not undoubtedly confirm **3**, it points to the complexity of the speciation. We posit that multiple species are observed due to varying types of soluble thiolate-bridged [Ni­(S-*p*-CF_3_–Ph)_2_]_n_ complexes with variable n (≥2). For example, complexes featuring linked aryl-thiolato-bridges such as the oligomeric ring complexes [Ni­(SPh)_2_]*_n_* (*n* = 9, 11; all planar Ni^2+^),^89^ the face-sharing tetrahedral dimer [(RS)­Ni­(μ_2_-SR)_3_Ni­(SR)]^−^ (R = 2,4,5-triisopropylbenzenethiolate),^90^ and the *p*-Cl analogue of **3** ^57^ (vide infra) provide support for this proposal. Bridged complexes such as **3** being in equilibrium with monomer/tetrahedral **1** in solution may advocate against the presence, or sufficient lifetime, of a putative [Ni­(SPh)_3_(solv)]^−^ (**I**) species.^56^ Multimeric planar Ni-thiolate complexes form under low thiolate-to-Ni ratios employed in synthesis and can occur upon thiolate dissociation due to protonation (or spontaneously) and substitution with donor solvents.^37,91,92^ Indeed, spontaneous thiolate dissociation readily occurs even with chelating alkyl-thiolato-bound Ni^2+^ complexes to afford bridged species.^93^ Thus, intermediates such as **I** would be fleeting at best. Yamamura suggested that tetrahedral [Ni­(SPh)_4_]^2–^ is in equilibrium with the planar version (*S* = 0) of complex **I** in DMSO, which is the source of the peaks at ∼7 ppm in the ^1^H NMR.^56^ However, Maroney demonstrated that these peaks originate from a thiolate-bridged dimeric complex analogous to **3**, and were successful in growing crystals and obtaining ^1^H NMR, albeit through an ill-defined reaction between [Ni­(Me_6_tren)­Cl_2_] (Me_6_tren = tris­(2-(dimethylamino)­ethyl)­amine and (Et_4_N)­(S-*p*-Cl-Ph).^57^ Thus, spectral comparisons with this bridged species suggest that the peaks at ∼7 ppm belong to complexes analogous to **3**. Based on the results presented here, it is possible, although difficult to verify, that the MeCN-solvated complex [Ni­(S-*p*-CF_3_–Ph)_3_(MeCN)]^−^ (**I** in eqs 1–2; Scheme 1) is a common intermediate between **1** and **3** according to Scheme 1 (DFT-estimated Δ*G* in blue).

**Scheme 1:**
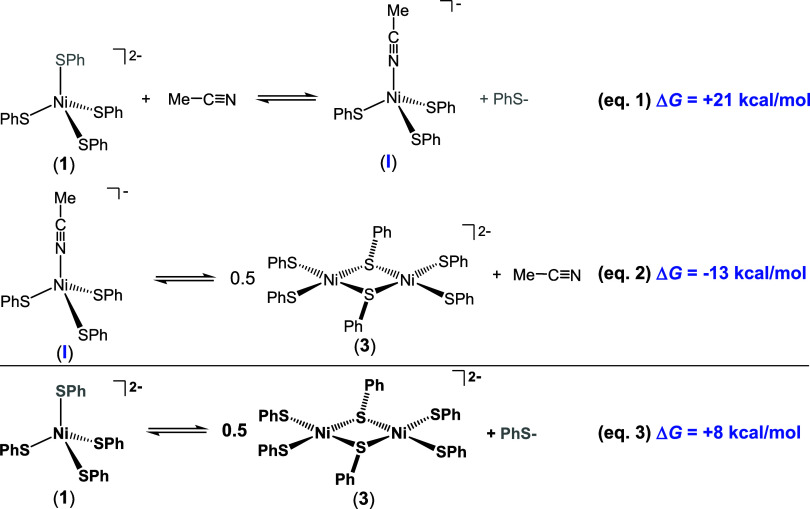
Stepwise Path to Formation of *S*,*S*-Bridged Planar **3** from Monomer/tetrahedral **1** via Putative [Ni­(SPh)_3_(MeCN)]^−^ Intermediate **I** ^a^

a PhS designation is used for simplicity, complex **1** contains coordinated *p*-CF_3_–PhS; MeCN was used for this work but can represent any donor solvent in **I**; square-planar **I** (*S* = 0), suggested in ref 55 (where solv = DMSO), was computed to be 14 kcal/mol less stable than the tetrahedral (*S* = 1) geometry.

ESI-MS­(−) measurements of **1** reveal that [Ni­(S-*p*-CF_3_–Ph)_3_]^−^, a likely dimer precursor/fragment, is the most intense ion detected (Figure S11) under electrospray conditions. Additionally, strong donor solvents favor the product side of eq 1 of Scheme 1 that may eventually yield dimer. Furthermore, delivering excess thiolate equivalents to solutions of **1**, favor the tetrahedral [Ni­(S-*p*-CF_3_–Ph)_4_]^2–^ complex based on UV–vis (see Figure 5). As written in Scheme 1, excess thiolate (eq 1, eq 3) and suitable donor solvents (eq 2) favor monomer; however, prior reports indicate that planar dimers featuring aryl-thiolates such as **3** are substitutionally inert and do not readily convert back to monomeric/tetrahedral **1** even with additional thiolate.^55,57^ In contrast, adding thiolate to solutions containing a mixture of thiolate-bridged complexes including **3** (reverse of eq 3) indicates monomeric/paramagnetic **1**, evidenced by characteristic peaks in ^1^H/^19^F NMR (Figures S47–S49), and the appearance of the two adjacent CT bands in the UV–vis (Figure S50). Several groups also report using thiolates or neutral N-donors to readily cleave *S*,*S*-bridged square-planar Ni-dimers into their corresponding monomeric species.^64,94–97^ Thus, we assign the peaks at δ ∼ 7 ppm in the ^1^H NMR of freshly dissolved monomer **1** to soluble thiolate-bridged complexes such as **3** or of higher nuclearity that cannot be eliminated or identified at present. Both **1** and the bridged complex­(es) are interconvertible, with **1** being predominant when thiolate is in excess.

DFT allowed us to gain further insight into the solution speciation represented by eqs 1–3 (Scheme 1). To estimate the preference for each of the aforementioned species, we computed the Gibbs free energy difference (Δ*G*) of the reactions described by eqs 1–3 using DFT at the B3LYP/def2-TZVPP level of theory with an MeCN solvent field (ε = 37.5). A clear preference for tetrahedral monomer **1** was indicated. For example, the Δ*G* of eq 3, which describes the overall *S* = 1 monomer **1**/*S* = 0 dimer **3** conversion, computes as an endergonic reaction (8 kcal/mol) in favor of the reactant/monomer side, i.e., **1**. If one considers the stepwise path to **3** via eqs 1–2, then **1** or **3** are favored by 21 and 13 kcal/mol, respectively, over a postulated *S* = 1 tetrahedral intermediate [Ni­(S-*p*-CF_3_–Ph)_3_(MeCN)]^−^ (**I**). The possibility of a planar intermediate **I** was also investigated but not included due to a high Δ*G* (26 kcal/mol) in favor of the reactant side of eq 1. An optional proposal regarding the peaks in the ∼7 ppm region of the ^1^H NMR is an equilibrium between tetrahedral complex **1** and its *S* = 0 square-planar isomer. However, the computed Δ*G* between these geometric isomers is 14 kcal/mol in favor of tetrahedral **1**.

### Solution Behavior of **1** in the Presence of Brønsted Acids

With the intent of utilizing **1** for HER electrocatalysis, we examined the behavior of **1** in the presence of strong and weak Brønsted acids such as HBF_4_·Et_2_O (p*K*_a_ = 1.8 in MeCN^98^) and AcOH (p*K*_a_ = 23.5 in MeCN^61^), respectively. Earlier studies indicate formation of insoluble complexes, represented as [Ni­(SPh)_2_]_∞_, when weak acids such as EtOH or H_2_O are added to solutions of [Ni­(SPh)_4_]^2–^.^42,56^ Consistent with prior reports, addition of either stoichiometric HBF_4_·Et_2_O or 25 mol-equiv AcOH to dark-red MeCN solutions of **1** result in precipitation of a fine purple powder and a nearly colorless solution. IR/NMR analysis of this precipitate and the bleached MeCN solution suggest a [Ni­(S-*p*-CF_3_–Ph)_2_]*_n_*-type complex and thiol HS-*p*-CF_3_–Ph, respectively. Full characterization of the purple precipitate was limited due to poor solubility. A qualitative UV–vis spectrum of this solid in DMSO revealed an intense band at 510 nm (Figures S52–S53). Monitoring the NMR of **1** upon addition of AcOD-*d*_4_ suggest loss of **1** even in the presence of low acid/**1** ratios (e.g., 5:1), indicated by the change in solution color to pale brown and attenuation of the NMR signals of **1** (Figures S56–S58). The fine purple precipitate is eventually observed when >20 mol-equiv of AcOD-*d*_4_ are added, as described above. Clearly, as-isolated **1**, even in the presence of weak acids, readily protonates a coordinated thiolate to yield intractable species. To impede polymerization and shift to tetrahedral/monomer **1**, excess (Et_4_N)­(S-*p*-CF_3_–Ph) was added to CD_3_CN solutions of **1** and the same NMR experiment with AcOD-*d*_4_ was conducted. Titration of AcOD-*d*_4_ up to 60 mol-equiv indicates 95% of tetrahedral/monomer **1** remains based on ^1^H/^19^F NMR (Figures S59–S61). Thus, undesired polynuclear Ni-thiolate complexes in acidic organic media can be suppressed by utilizing excess ligand.

### Electrochemistry

Electrochemical experiments were completed in MeCN with 0.25 M ^*n*^Bu_4_NPF_6_ as supporting electrolyte using a GC working electrode; all potentials are reported versus an externally referenced ferrocenium/ferrocene (Fc^+^/Fc) couple. For comparative purposes, potentials for previously reported Ni-thiolate complexes are converted to the same reference potential.^99^ The cyclic voltammogram (CV) of **1** is complex and displays several irreversible features (Figure S63). Scanning cathodically revealed one irreversible reduction at −1.98 V (*E*_red_), assigned to the Ni^2+/1+^ couple, along with several irreversible oxidation waves. Recording the CV with excess thiolate, to ensure only monomeric/tetrahedral species (vide supra), revealed the same *E*_red_ event at −1.94 V consistent with the Ni^2+/1+^ assignment, and an irreversible oxidation (*E*_ox_) at −0.38 V (Figure 8). The irreversibility of the reduction wave, even with excess thiolate, indicates a structural change accompanies this process, proposed to be thiolate dissociation to afford three-coordinate (3C) [Ni­(S-*p*-CF_3_–Ph)_3_]^2–^. Reversing the scan resulted in a similar current response at *E*_ox_ = −0.38 V and a new reduction event at −1.60 V in addition to the Ni^2+/1+^ couple ∼−2 V (Figure 8). Comparing the CV of **1** to (Et_4_N)­(S-*p*-CF_3_–Ph) (*E*_ox_ = −0.33 V, *E*_red_ = −1.68 V; Figure S62) and other *p*-substituted aryl-thiols/disulfides^100^ suggests these waves are due to free thiolate oxidation/disulfide reduction. The CV of Zn^2+^ complex **2** provides further justification for this assignment as *E*_red_ ∼ −1.70 V only appears with scans initiated anodically, i.e., oxidation of the Zn-coordinated thiolate (*E*_ox_ = 0.11 V) is required to generate disulfide and enable *E*_red_ (Figure S64). Other waves in the CV of **1** without excess thiolate are difficult to assign due to the speciation. Expectedly, these oxidation events are minimized in the CV of **1** recorded with additional thiolate (Figure 8). Because Fc^+^ oxidation of **1** affords disulfide and a mixture of thiolate-bridged complexes such as **3** (vide supra), we assign oxidation events more positive than −0.40 V (see Figure S63) to coordinated thiolates, i.e., no Ni^3+^ intermediate(s).

**Figure 8:**
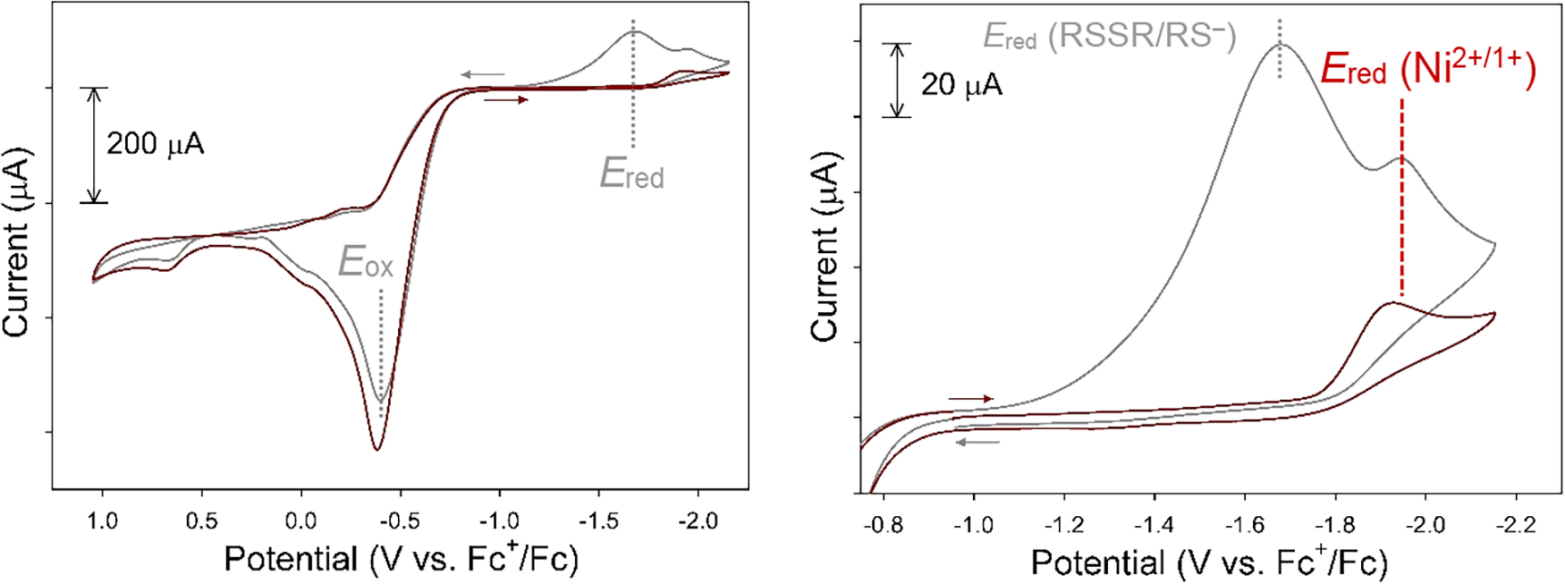
CV of complex **1** (1.89 mM) with 20 mol-equiv (Et_4_N)­(S-*p*-CF_3_–Ph) in MeCN at RT (0.25 M ^*n*^BuN_4_PF_6_ supporting electrolyte, GC working electrode, 100 mV/s scan rate). *Left*: full CV window measured. *Right*: expansion of cathodic region to display the Ni^2+/1+^ couple. Arrows display the scan direction (red = cathodic scan, gray = anodic scan). The *E*_ox_ (RS^–^/RSSR) and *E*_red_ (RSSR/RS^–^) of the thiolate are listed in gray (left); the Ni^2+/1+^ couple is marked in red (right).

Making comparisons to analogous tetrahedral homoleptic Ni-aryl-thiolates is limited, but all propose primarily ***irreversible redox*** events. For example, voltammetric measurements on [Ni­(SPh)_4_]^2–^ report *E*_ox_, assigned as Ni^3+/2+^, in different solvents (−0.57 V in DMSO;^42^ −0.44 V in MeCN;^82^ see Table 2). The close match to the *E*_ox_ of PhSH (−0.49 V in DMF^100^) suggests this oxidation is likely due to free thiolate and not a Ni^3+^ species. Notably, reversible Ni^3+/2+^ couples have been reported for planar dicarboxamido/dithiolato-NiN_2_S_2_ complexes which are quite negative (*E*_1/2_ = −0.70 to −0.90 V in DMF or MeCN^101,102^ ). Tetrathiolato/planar complexes containing chelating dithiolates also display rather low (quasi)­reversible Ni^3+/2+^ events that center around −1 V in DMF (Table 2).^43–46^ Irreversible S-based redox have been reported in the CVs for planar/anionic Ni^2+^ complexes containing monodentate aryl-thiolates with supporting tridentate ancillary ligands such as the complex anions [Ni­(pdmt)­(SPh)]^−^ (pdmt = pyridine-2,6-dimethanethiolate; *E*_ox_ = −0.47 V, *E*_red_ = −1.73 V in DMF^94^), [Ni­(L^S^)­(Mes^S^)]^−^ (L^S^ = bis­(2-mercaptophenyl)­sulfide, Mes^S^ = 2,4,6-trimethylthiophenolate; *E*_ox_ = −0.83 V, *E*_red_ = −2.03 V in MeCN^103^), and [Ni­(nmp)­(S-*p*-CF_3_–Ph)]^−^ (nmp = dianion of *N*-(2-mercaptoethyl)­picolinamide; *E*_ox_ = −0.19 V, *E*_red_ = −1.64 V in DMF^97^). The latter complex contains the identical aryl-thiolate as in **1**, albeit in a planar geometry. Given that *E*_ox_ of Zn complex **2** is +0.11 V, coupled with the *E*_ox_ values of the aforementioned planar Ni-thiolate complexes, we assign the *E*_ox_ at −0.08 V to the coordinated thiolates of **1**, whereas the *E*_ox_ at −0.20 V may be due to Ni-bound thiolates of thiolate-bridged complexes such as **3** (see Figure S63). Indeed, the *E*_ox_ values (−0.20, +0.13 V in MeCN^57^) for the related *S,S*-bridged dimer [Ni_2_(S-*p*-Cl-Ph)_6_]^2–^ are close to those reported here.

Although oxidative events have been described for homoleptic tetrahedral and planar Ni-thiolate complexes, descriptions of reductive events are scarce. The reversible Ni^2+/1+^ couple for a related tetrahedral NiS_4_ complex [(PhTt^tBu^)­Ni-SPh] is −1.17 V vs Fc^+^/Fc in THF and provides one benchmark, but this complex is charge neutral with only one Ni-thiolate bond and hence the reduction is expectedly facile.^67^ For the homoleptic/tetrahedral Ni­(SCys)_4_ site in NiRd, a direct analogue of **1**, Shafaat reports an estimated Ni^2+/1+^ potential of −2.01 V vs NHE^59^ (∼−2.70 vs Fc^+^/Fc in MeCN^81^), considerably more negative than **1** due to coordination of alkyl- vs aryl-thiolates. However, this value is in-line with what we compute for the reversible *E*_1/2_ (Ni^2+/1+^) for **1** at −2.69 V (MeCN; vide infra), i.e., no thiolate dissociation during redox. Homoleptic/square-planar tetra-aryl-thiolato-Ni complex [Ni­(HPyS)_4_]^2+^ (HPyS = N-protonated form of pyridine-2-thiolate) demonstrates an irreversible Ni^2+/1+^ couple at −1.78 V in DMF, slightly more positive than **1**.^51^ While this value may be contrary to that expected for a planar Ni complex, the more positive *E*_ox_ likely arises from the overall cationic charge on the complex. For comparison, the *E*_1/2_ (Ni^2+/1+^) values for square-planar neutral diamino/dithiolato-NiN_2_S_2_ complexes are considerably more cathodic with potentials ∼−2.40 V in MeCN.^93,104^ More in-line with the expectation for a planar tetrathiolato-Ni complex is the irreversible reduction of the *S*,*S*-bridged hexameric complex [Ni_6_(PET)_12_] (PET = 2-phenylethanethiolate), which exhibits *E*_red_ around −2.40 V in THF.^84^ Other planar and multimeric *S*,*S*-bridged Ni complexes with alkyl-thiolates show low Ni^2+/1+^ couples,^83,105–107^ down to −2.40 V (Table 2), comparable to NiRd and more negative than the analogous couple in **1**.

### HER Electrocatalysis

Complex **1** was investigated for its activity in electrocatalytic proton reduction to H_2_(g) or HER. CV measurements were acquired after titrating proton equivalents of the weak Brønsted acid AcOH into MeCN solutions of **1** (0.25 M ^*n*^Bu_4_NPF_6_ electrolyte, GC working electrode, scan rate = 100 mV/s). Addition of AcOH to solutions of **1** resulted in catalytic waves (*E*_cat_) at −1.47 and −1.92 V (Figure S65); however, only *E*_cat_ at −1.92 V produced significant current enhancement suggestive of electrocatalysis. Standard rinse tests indicate no deposition of catalytic nanomaterial on the working electrode (Figure S66).^108,109^ While these results suggest **1**, or a complex derived from **1**, is an electrocatalyst for HER, its speciation is complex under these specific conditions. For example, addition of only 4–6 mol-equiv AcOH resulted in a color change of the solution from dark maroon-red to brown with the appearance of a new peak at −1.47 V (Figure S65) that modestly increased with [AcOH]. Addition of >20 mol-equiv resulted in lightening of the solution color and a purple precipitate, likely insoluble [Ni*_n_* (S-*p*-CF_3_–Ph)_2*n*_]*_n_* (*n* ≥ 2) oligo/polymeric species^62^ (vide supra) as suggested by Yamamura ∼40 years ago.^42^ Extrapolating valid figures of merit under these conditions was thus not possible.

To circumvent the formation of intractable species, HER activity was next evaluated in the presence of excess (Et_4_N)­(S-*p*-CF_3_–Ph). Adding 20 mol-equiv of thiolate to MeCN solutions of **1** ensures the presence of tetrahedral monomer as confirmed by UV–vis and NMR spectroscopy (vide supra; see Figures 5 and S46). As with **1** alone, titrating AcOH into MeCN solutions of **1**/20 mol-equiv thiolate resulted in an electrocatalytic current response (Figure 9) with one major event near the Ni^2+/1+^ couple at *E*_cat_ = −2.23 V, and a minor response at −1.93 V. The primary difference with excess thiolate present is loss of *E*_cat_ ∼ −1.5 V (Figure S65), proposed to be due to formation of thiolate-bridged complexes upon increasing proton equivalents.^42,93^ The absence of this *E*_cat_ supports this hypothesis. From these results, the η of HER catalyzed by **1** was found to be 0.72 ± 0.02 V using a standard method that accounts for homoconjugation of AcOH (see eq iii in the Experimental Section),^110^ with a TOF of 14.5 ± 3.6 s^–1^, see eq iv in the Experimental Section, obtained from *i*_c_/*i*_p_ ratios at saturating acid conditions (∼20 mol-equiv AcOH). Additional metrics obtained from controlled potential electrolysis (CPE) such as turnover number (TON) and Faradaic efficiency (FE) were not performed due to the limited protic instability of **1** under the large [H^+^] required to generate detectable H_2_(g). While the electrocatalytic activity of **1** is modest, it is comparable to TOFs reported for other [NiFe]-H_2_ase models that employ two metal sites and/or more elaborate ligand constructs (see Chart 2, S1, and Table 3).^31,106,107,111–114^ Additionally, the solution color in the electrochemical cell remains red throughout the experiment with no precipitate, consistent with tetrahedral/monomer **1**. The presence of **1** under these conditions was further confirmed by the distinct double-humped UV–vis spectrum of the reaction mixture taken directly from the electrochemical cell at the end of acid titration (Figure S68). The amount of **1** remaining in solution, estimated from ^1^H/^19^F NMR and UV–vis experiments is >90% after addition of 20 mol-equiv of AcOH. Standard rinse tests indicate no deposition on the GC working electrode (Figure S67). Furthermore, CVs of AcOH in the presence of Zn^2+^ complex **2** and with (Et_4_N)­(S-*p*-CF_3_–Ph) (Figure S69) under identical conditions reveal that the Ni^2+^ complex is solely responsible for the electrocatalysis displayed in Figure 9. The HER activity of dimer **3** was not measured in this work because it could not be obtained in pure form. However, insight on its HER capacity can be extrapolated from a series of analogous, albeit charge neutral, planar [L-Ni­(μ-SAr)_2_Ni-L] (L = substituted aminobis­(thiophenolate) ligands) dimers constructed by Castillo and colleagues.^115–117^ Among the reported series of complexes, only dimers with close Ni---Ni separations (∼2.7 Å) and relatively acute hinge angles (angle defined by the intersection of the two Ni square planes) were adequate HER electrocatalysts. The large Ni---Ni separation (∼3.4 Å; Table S8) computed for **3**, along with coplanar NiS_4_ planes (hinge angle ∼ 180°) suggest **3** would be a poor H_2_ evolving electrocatalyst.

**Figure 9:**
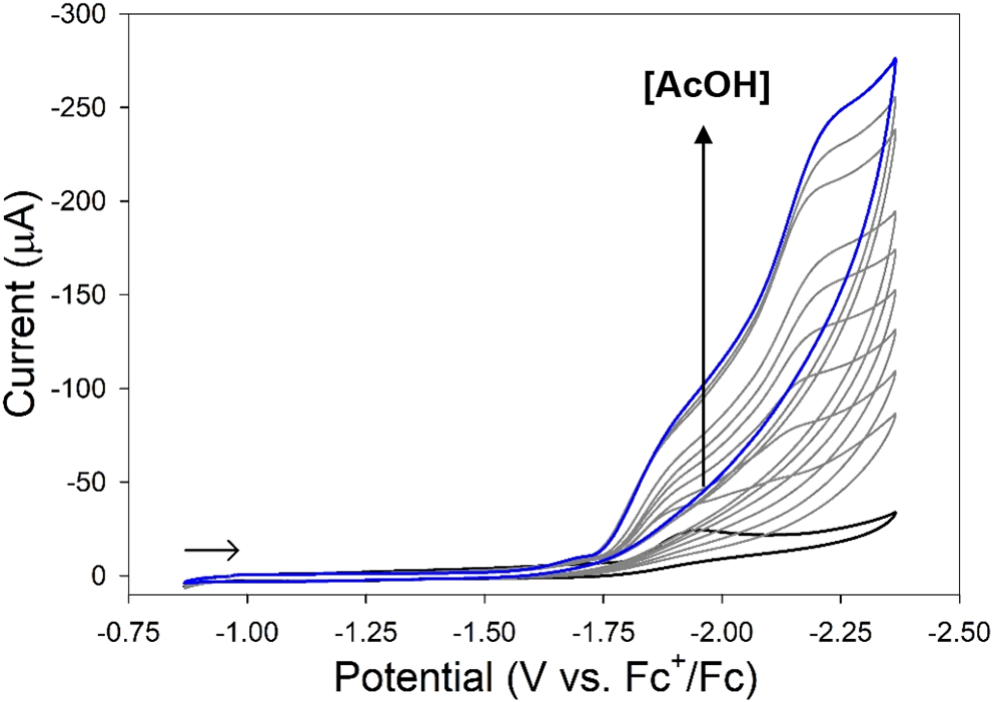
CV of complex **1** (1.89 mM) with 20 mol-equiv (Et_4_N)­(S-*p*-CF_3_–Ph) (black trace) and after the addition of AcOH in 2 mol-equiv increments (gray traces) up to a final of 18 mol-equiv (blue trace) in MeCN at RT. Conditions: 0.25 M ^*n*^BuN_4_PF_6_ supporting electrolyte, GC working electrode, 100 mV/s scan rate. Arrow displays the scan direction.

**Table 3:** Select Parameters for Ni-thiolate HER Electrocatalysts (See Charts 1–2, Chart S1 in the SI for Structures)^a^

complex	TOF (s^–1^)	*η* (V)	H^+^ source	solvent	ref
**1**	14.5	0.72	AcOH	MeCN	TW
[Ni(xsbms)]	<1.0	NR	Et_3_N·HCl	DMF	^105^
[Ni(‘S_4_′)]	NR	0.76	TFA	DMF	^107^
[Ni(PS)_2_]	51	1.10	AcOH	CH_2_Cl_2_	^113^
[NiN_2_S_2_]	1441, 5575	0.56, 0.67	AcOH	MeCN	^114^
[Ni_3_S_6_]	NR	0.59	Et_3_N·HCl	DMF	^106^
[HNiFe(pdt)(dppe)(CO)_3_]^+^	20	0.50	TFA	CH_2_Cl_2_	^112^
[HNiFe(edt)(dppe)(CO)_3_]^+^	240–310	0.49	TFA	CH_2_Cl_2_	^112^
[Ni(xsbms)FeCpCO]^+^	NR	0.73	TFA	DMF	^111^
[L^N2S2^Ni^II^Fe^II^]^+^	250	0.69	Et_3_N·HBF_4_	MeCN	^31,125^
[Ni–Fe′]^+^	52	0.94	TFA	MeCN	^32^
NiRd^b^	100	0.54	H_2_O^c^	buffer^c^	^58^

a Abbreviations: xsbms = 1,2-bis­(4-mercapto-3,3-dimethyl-2-thiabutyl)­benzene. ‘S_4_′ = 1,3-bis­(1,2-benzenedithiolate)­propane. PS = 2-(diphenylphosphanyl)­benzenethiolate. N_2_S_2_ = *N*,*N*′-dimethyl-*N*,*N*′-bis­(2-sulfanylethyl)­ethylenediamine. S_6_ = (tritylthio)­ethylthio)­methyl)-1,2-dithiolate. dppe = diphenylphosphinoethane, pdt = propane dithiolate, edt = ethane dithiolate. L^N2S2^ = (2,2′-(2,2′-bipyridine-6,6′-diyl)­bis­(1,1-diphenylethanethiolate)). Ni–Fe′ = Ni­(bismercaptoethane diazacycloheptane)-Fe­(CO)­(η^5^-C_5_H_5_). TW = this work; NR = Not reported.

b NiRd from *Desulfovibrio desulfuricans*.

c Acetate buffer, pH = 4.5, 4 °C.

Additional kinetic parameters were obtained from plots of the catalytic current (*i*_c_) or ratio of *i*_c_ to the reduction peak current of **1** in the absence of acid (*i*_p_), i.e., *i*_c_/*i*_p_ with respect to various conditions. For instance, *i*_c_/*i*_p_ exhibits a linear dependence on [H^+^] until the acid-independent region is reached at ∼30 mM (16 equiv) AcOH and a maximum *i*_c_/*i*_p_ ratio of ∼10 (Figure S70). The linear plot of *i*_c_ versus [H^+^] indicates a second-order dependence on [H^+^].^118^ Similarly, *i*_c_ plotted against [**1**] (Figure S71) is also linear to indicate a first-order dependence of the catalytic rate on [**1**]. Hence, the results obtained correspond to the overall rate expression: rate = *k*[**1**]­[H^+^]^2^, using AcOH as the H^+^ source. Further insight into the rate-limiting step and mechanism can be gleaned from the kinetic isotope effect (KIE, *k*_H_/*k*_D_). Utilizing AcOD-*d*_4_, the KIE for HER was determined to be 1.62, obtained from the ratio of the TOF values under saturating conditions of the H^+^ and D^+^ sources (*i*_c_/*i*_p_ values at 20 mol-equiv (40 mM) of AcOH or AcOD-*d*_4_). This value falls within the so-called “normal” KIE regime, and contrasts with that expected for an inverse KIE (<1), generally attributed to rate-determining metal-hydride formation,^119–121^ or large KIE values (>8), due to hydrogen tunneling.^122^ The KIE determined here, to some degree, is comparable to the reported KIE of 2.6 for H_2_ production by [NiFe]-H_2_ase^123^ and 3.81 for NiRd.^59^ In the case of **1**, the rate-determining step is visualized by intramolecular coupling of a Ni-hydride to a proton on a coordinated thiol, viz. intramolecular H^+^/H^–^ coupling toward H_2_ evolution (vide infra) as suggested for other LMW Ni complexes.^32,33,107,124^

### Proposed Mechanism of HER by **1**

Mechanistic possibilities were investigated through DFT (B3LYP, def2-TZVPP) using the conductor-like polarizable continuum model (CPCM) to approximate the MeCN solvent environment (details in the SI; see Figures S73–S77 and Tables S15–S19). Because *E*_red_(Ni^2+/1+^) at −1.94 V coincides with *E*_cat_ (see CV Figure 9), our working mechanism (Scheme 2) begins from the 3C trigonal planar (τ_3_ = 0.04;^59^ ∑Ni_α_ = 359.9°; see Figure S74, Table S17) Ni^1+^ doublet dianion ^**2**^ **1** ^**R**^, generated after reduction of **1**. Attempts to synthesize and characterize a Ni^1+^-thiolate species derived from **1** via chemical reduction with strong reducing agents such as KC_8_ or CoCp*_2_ were not successful and only led to ill-defined materials. However, experimental support for this probable structural change is indicated by the irreversible nature of the Ni^2+/1+^ wave in the CV (see Figure 8). Additionally, dissociation of one aryl-thiolate from the putative 4C-tetrahedral (τ_4_ = 0.81) Ni^1+^ species [Ni­(S-*p*-CF_3_–Ph)_4_]^3–^ is favorable and computed to be exergonic by 18 kcal/mol. To benchmark the method in estimating Ni reduction potentials (*E*_1/2_), the absolute potential of the Fc^+^/Fc reference couple was calculated to be +5.009 V (Table S15 in the SI^126–130^ ). Utilizing known values for the absolute potential of the SHE (+4.281 V^131^) coupled with its conversion against a common reference electrode (SHE = +0.244 V vs SCE in MeCN^81^) results in a computed *E*_1/2_(Fc^+^/Fc) of +0.48 V vs SCE in MeCN, comparable to the experimental value of +0.38 V.^99^ The reversible, i.e., no thiolate loss during redox, Ni^2+/1+^ potentials were then separately computed for the 4C and 3C complexes. *E*_1/2_(Ni^2+^/Ni^1+^) was estimated to be −2.69 and −1.68 V (vs Fc^+^/Fc) for 4C-tetrahedral [Ni­(S-*p*-CF_3_–Ph)_4_]^2–/3–^ and 3C-trigonal [Ni­(S-*p*-CF_3_–Ph)_3_]^1–/2–^, respectively, bracketing a range where the experimental value (*E*_red_ = −1.94 V) falls close to the mean.

**Scheme 2:**
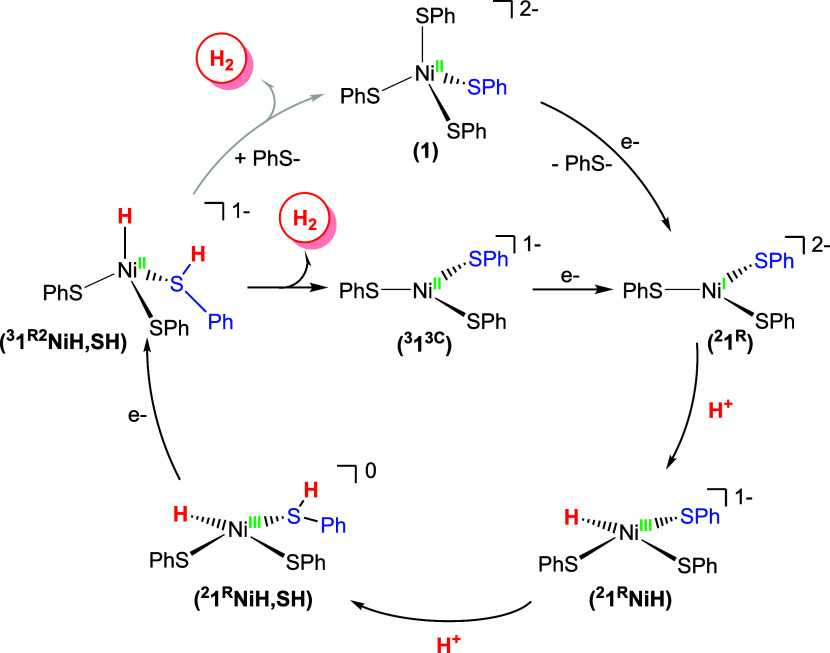
Proposed ECCE Mechanism for the HER Catalyzed by Complex **1** ^a^

a After the first complete cycle, the following cycles begin with ^**3**^ **1** ^**3C**^ following an ECCE mechanism.

Beginning with the doublet (*S* = 1/2)/3C species ^**2**^ **1** ^**R**^ (Ni–S_avg_: 2.296 Å), four plausible paths were considered: (i) ECCE (E = electrochemical step; C = chemical step), and (ii) ECEC mechanisms where a ***Ni–H intermediate*** is traversed after the first “E” step (reduction of **1** to ^**2**^ **1** ^**R**^); or (iii) ECCE, and (iv) ECEC paths where ***ligand protonation*** occurs after reduction. A second consecutive reduction step, or EECC mechanism, was also computed but not further considered because of the unfavorable barrier to access a Ni(0)-thiolate species (*E*_1/2_(Ni^1+^/Ni^0^) ∼ −4 V; data not shown). Planar and tetrahedral intermediates for each path were also considered (see Scheme S3; Figures S73–S75). Paths iii and iv, involving a thiolate protonation second “C” step, were eliminated due to large differences in Δ*G*, upward of 50 to 70 kcal/mol. Focusing on mechanisms i and ii, the first “C” step (second overall) results in a Ni-hydride (NiH) intermediate (**1** ^**R**^ **NiH** = one-electron reduced from ^**3**^ **1** ^**3C**^/Ni-hydride intermediate) that can either be square-planar (*S* = 1/2; ^**2**^ **1** ^**R**^ **NiH**) or tetrahedral (*S* = 3/2; ^**4**^ **1** ^**R**^ **NiH**); however, computations suggest that the NiH intermediate is more stable by ∼12 kcal/mol in square-planar geometries (Δ*G* values for both isomers depicted in Figure S73, ECCE pathway) and accordingly we concentrate the initial discussion on these species.

Protonation of ^**2**^ **1** ^**R**^ yields ^**2**^ **1** ^**R**^ **NiH** (Δ*G* = +14 kcal/mol; Scheme 2), formally a Ni^3+^–H species, resulting in an intermediate four-coordinate complex with only a slight geometric bias toward planar geometry (τ_4_ = 0.43; see Figure S75 and Table S18 for structural information). The calculated Ni–H length (1.46 Å) compares favorably to other DFT-estimated distances in thiolato-ligated Ni, including a tetrahedral Ni^3+^ complex from Peters (Ni–H: 1.550 Å)^132^ and the five-coordinate Ni^3+^–H in NiRd reported by Shafaat (Ni–H: 1.41 Å).^59^ Experimentally, terminal Ni–H bond lengths appear over a broad range (1.32–1.65 Å), with planar systems at the lower end as observed here.^133^ Two paths follow ^**2**^ **1** ^**R**^ **NiH**; mechanism i, ligand protonation (ECCE, vide infra), or mechanism ii, reduction (ECEC). Although both routes lead to the same common intermediate before H_2_ evolves (**1** ^**R2**^ **NiH,SH** = doubly reduced from **1**/Ni-hydride, protonated/coordinated thiol intermediate), protonation (*path i*, Scheme 2) is favored by ∼10 kcal/mol over reduction (*path ii)*. Protonation of coordinated thiolate affords the distorted planar complex ^**2**^ **1** ^**R**^ **NiH,SH** (τ_4_ = 0.40, +23 kcal/mol from ^**2**^ **1** ^**R**^ **NiH**). The diminished charge on the bound thiol results in elongation of the Ni–thiol bond by ∼0.08 Å and contraction of the remaining Ni-thiolate bonds (∼0.08 Å) from the ^**2**^ **1** ^**R**^ **NiH** precursor (Figure S75, Table S18). The final “E” step computes *E*_1/2_(Ni^3+/2+^) = −0.58 V (Δ*G* = +13.4 kcal/mol; Figure S73) going from ^**2**^ **1** ^**R**^ **NiH,SH** to ^**1**^ **1** ^**R2**^ **NiH,SH** (*S* = 0). The anionic intermediate ^**1**^ **1** ^**R2**^ **NiH,SH** is more square-planar (τ_4_ = 0.20; Figure S75, Table S18) than its precursor with the anticipated lengthening of all Ni–S bonds after reduction to Ni^2+^. Notably, the analogous tetrahedral (*S* = 1) species ^**3**^ **1** ^**R2**^ **NiH,SH** is nearly isoenergetic (ΔΔ*G*: 1.6 kcal/mol) with planar ^**1**^ **1** ^**R2**^ **NiH,SH**, indicating that involvement of either coordination geometry is valid (vide infra). Regardless of the exact geometry of the penultimate species in our working model, experimental support for such intermediates exists in the report of the tetrahedral Ni-nitrosyl species [Ni­(SPh)_3_(NO)]^2–^ (**II**; *S* = 0) obtained from the reaction of [Ni­(SPh)_4_]^2–^ with NO­(g) or Ph_3_CSNO.^64^ Indeed, complexes such as **II** are isolable with the thiolate used in this work; (Et_4_N)_2_[Ni­(S-*p*-CF_3_–Ph)_3_(NO)] (**4**) has been synthesized in modest yield with characterization (Figures S27–S31) suggestive of a structure identical to **II**. Notably, IR indicates the N–O stretching frequency for **4** (ν_NO_: 1683 cm^–1^ in KBr) is ∼30 cm^–1^ upshifted from **II** because of the electron-withdrawing *p*-CF_3_ group. Although H^+^ is not exactly isolobal with NO, one can view both as redox-active (to some degree) small molecules that react with tetra-aryl-thiolato nickelates in a similar fashion to yield 4C [Ni­(SAr)_3_(H/NO)]^n–^ analogous to intermediates such as ^**3**^ **1** ^**R2**^ **NiH,SH** in Scheme 2.

The formation of the **1** ^**R2**^ **NiH,SH** intermediate regardless of geometry, i.e., square-planar or tetrahedral, sets up H_2_ evolution via a heterolytic coupling pathway, consistent with other proposed mechanisms for transition metal hydride systems with adjacent thiol/M-H species^32,34^ and NiRd.^59^ To assess a potential transition state involved in this pathway, a relaxed surface potential energy scan (PES) was performed by probing the potential energy landscape as the Ni–H^–^---H^+^-SR unit is brought closer in space, in increments of 0.11 Å. The scan was performed by reoptimizing the structure at each point, while keeping the H---H bond distances fixed at each increment change with the remaining atoms remaining relaxed. Although the PES of both geometries were computed, only the PES of the tetrahedral intermediate ^**3**^ **1** ^**R2**^ **NiH,SH** (Scheme 2, Figures 10 and S76), revealed one clear transition state (TS_1_), as confirmed by the presence of one imaginary frequency, with a modest barrier for heterolytic coupling (Δ*E* ∼ 12 kcal/mol; Δ*G* ∼ 14 kcal/mol), comparable in magnitude to the computed barrier for H_2_ evolution from NiRd (Δ*G* ∼ 10 kcal/mol).^59^ The transition state results from the lengthening of Ni–H (Δ = +0.29 Å) and a slight contraction of S–H (Δ = −0.04 Å) bonds of ^**3**^ **1** ^**R2**^ **NiH,SH** with minimal distortion in the geometry (Figure 10). As the H---H distance approaches ∼0.7 Å, H_2_ is formed resulting in distorted trigonal-planar species [Ni^2+^(S-*p*-CF_3_–Ph)_3_]^−^ (^**3**^ **1** ^**3C**^; τ_3_ = 0.12;^59^ ∑Ni_α_ = 353.3°; see Figure S77, Table S19). Under experimental conditions, i.e., excess free thiolate, it is feasible for thiolate to rebind and regenerate 4C **1** (top of Scheme 2); however, during turnover, and on the electrochemical time scale, we propose the path likely proceeds from the ^**3**^ **1** ^**3C**^ intermediate as depicted in the center of Scheme 2 after one catalytic cycle.

**Figure 10:**
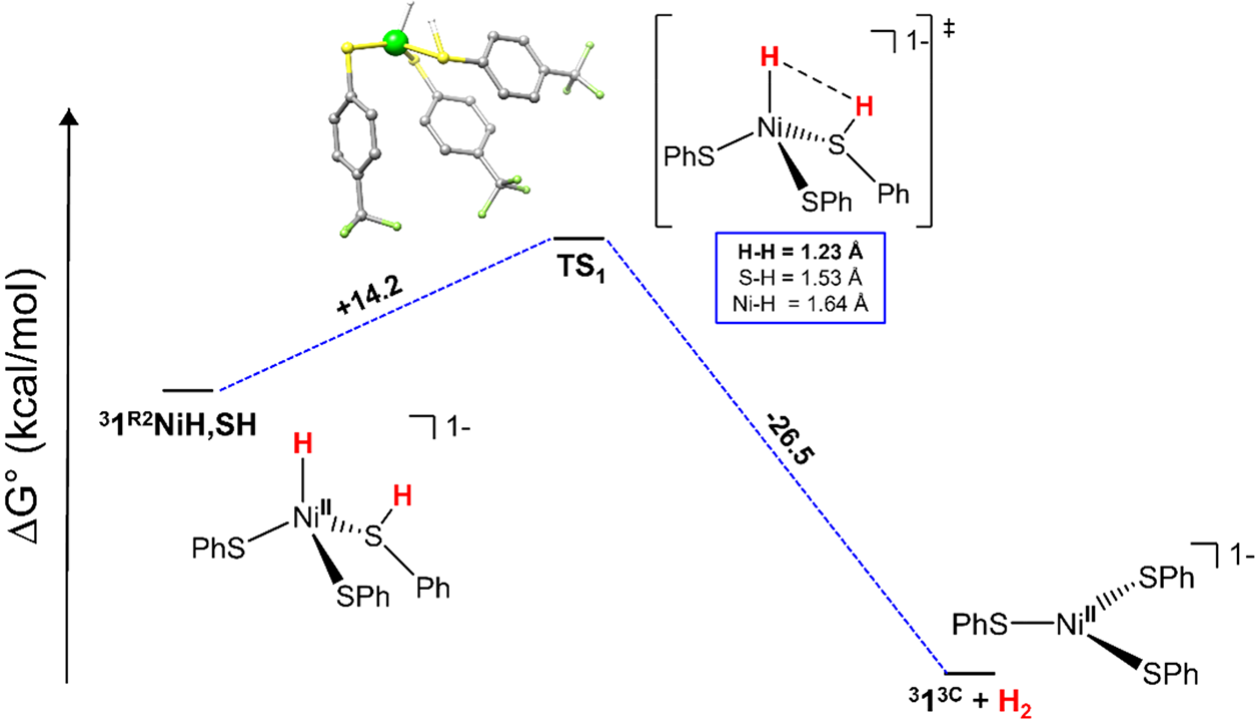
DFT-computed free energy profile (B3LYP/def2-TVZPP) of the transition state of the HER reaction (see Experimental section in the SI for details).

## Conclusion

This work demonstrates, for the first time, that simple homoleptic LMW tetrathiolato/tetrahedral complexes such as **1** are reasonable structural and moderate functional analogues for the tetracysteinato-ligated Ni center housed in the active site of [NiFe]-H_2_ase. While the activity of **1** for HER is rather modest (TOF ∼ 14 s^–1^, η ∼ 0.70 V in MeCN, acid: AcOH), standard electrode rinse tests and control electrochemical measurements with tetrathiolato-Zn^2+^ complex **2** and free thiolate clearly establish that the Ni-thiolate complex is the active species. This result is not surprising given the previously reported, and superior, H_2_ase activity of the Ni­(SCys)_4_ site in NiRd.^58^ While these Ni-site models were initially reported ∼40 years ago, their ability to perform HER was never evaluated likely due to issues regarding speciation to S-bridged complexes especially in protic solvents. As observed in prior reports of tetrahedral Ni-thiolate complexes,^55,56^ and even many square-planar Ni complexes with chelating dithiolates,^43,44,50^ **1** converts to a new species when dissolved in MeCN consistent with thiolate dissociation and formation of S-bridged and diamagnetic entities such as **3**. Contrary to what has been reported,^55,57^ these bridged species are not substitutionally inert and readily revert back to tetrahedral monomer **1** upon addition of extra thiolate equivalents. The presence of excess thiolate further enhances the acid stability of **1**. However, and even in the presence of additional thiolate, complex **1** is only irreversibly reduced to a Ni^1+^ complex at −1.94 V (Figure 8), proposed to be the 3C trigonal planar complex anion ^**2**^ **1** ^**R**^, which would alleviate buildup of negative charge at a putative 4C [Ni^1+^(SR)_4_]^3–^ species. Nevertheless, **1** is the (pre)­electrocatalyst for HER and is computed to go through an ECCE type mechanism with the Ni^1+^ complex ^**2**^ **1** ^**R**^ being one of the first intermediates (Scheme 2). Despite being structurally different from H_2_ase and NiRd due to thiolate loss after reduction to Ni^1+^, similar intermediates are traversed (Scheme 2). Although **1** closely resembles the active site structure of native [NiFe]-H_2_ase, its activity is lower compared to other Ni-electrocatalysts that lack thiolate-only donors (Charts 2 and S1, Table 3). We speculate the diminished activity of **1** is due to: (i) limited stability and multiple speciation upon addition of acid (dissociation of coordinated thiolate to yield 3C complexes, see Scheme 2 and Figure 10), (ii) lack of a second ligand-bridged/redox-active metal ion, (iii) less rigid/fluxional coordination sphere, (iv) absence of neighboring second-sphere proton relays, and (v) its overall dianionic charge. These issues are largely circumvented in the electrocatalysts displayed in Charts 2 and S1, and highlighted in Table 3, by employing rigid/multidentate chelates possessing less basic neutral coordinating ligands (e.g., phosphines, amines, thioethers) that result in an overall neutral or cationic complex charge. Despite this shortcoming, **1** serves as the ‘base model’ to initiate further studies and improve upon stability/catalytic capability in a simple construct. Future work is focused on preventing dissociation without the need for additional thiolate, and the resulting multimeric speciation, by employing more rigid scaffold-like multidentate ligands to fix the coordination geometry about Ni. This strategy has been successful in H_2_-activating mononuclear Fe models of the Fe-only H_2_ase.^134^ Dissociation can be further prevented by better abating the nucleophilicity of the Ni-bound thiolate, one reason for utilizing *p*-CF_3_–PhSH, by appending suitable H-bond donor functionalities in future ligand frames.^135,136^ Lastly, installation of proton-relay units, widely successful in other mononuclear Ni-HER^28,29^ and Co-CO_2_-reducing^137^ electrocatalysts is also planned.

## Experimental Section

### General Information

All reagents were procured from commercial suppliers and used as received unless otherwise noted. Acetonitrile (MeCN), tetrahydrofuran (THF), and diethyl ether (Et_2_O) were purified by passage through activated alumina columns using an MBraun solvent purification system (MB-SPS). 2-methyltetrahydrofuran (2-MeTHF) was used as received and degassed by three freeze–pump–thaw cycles. All solvents were stored over 3 Å molecular sieves in an anaerobic glovebox before use. Methanol (MeOH) was degassed by at least three freeze–pump–thaw cycles and stored over 3 Å molecular sieves for a minimum of 1 week before use. The metal salts, (Et_4_N)_2_[MCl_4_] (M = Ni, Zn),^138^ and triphenylmethyl-*S*-nitrosothiol (Ph_3_CSNO)^139^ were synthesized according to the published procedures. All reactions were performed under an inert atmosphere of N_2_ using Schlenk-line techniques or in an MBraun Unilab glovebox.

### Physical Methods

FTIR spectra were collected on a ThermoNicolet 6700 spectrophotometer running the OMNIC software. Solid-state samples were prepared as pressed KBr pellets. UV–vis spectra were recorded at 25 °C using a Cary-50 spectrophotometer containing a Quantum Northwest TC 125 temperature control unit. All UV–vis samples were prepared in gastight/screw-cap quartz cells with an optical path length of 1 cm. ^1^H (400 MHz), ^13^C (125 MHz), and ^19^F NMR (376 MHz) spectra were recorded in the reported solvent on a 400 MHz Bruker BZH 400/52 NMR spectrometer with chemical shifts referenced to the residual protio signal of the deuterated solvent.^140^ ^19^F NMR spectra are referenced to CFCl_3_ by employing a fluorobenzene internal standard (δ = −114.81 ppm in CD_3_CN vs CFCl_3_ ^141^). Magnetic susceptibility measurements were performed in both the solid- and solution-state. Solid-state magnetic measurements were recorded with a Johnson Matthey Mark 1 magnetic susceptibility balance. Solution-state magnetic measurements (Evans method^142^) and NMR titration experiments with acetic acid-*d*_4_ (CD_3_CO_2_D = AcOD-*d*_4_ from Cambridge Isotope Laboratories Inc., 99.5% D; 32 scans) were recorded in CD_3_CN at 25 °C on a 600 MHz (^19^F = 564 MHz) Varian Unity INOVA NMR spectrometer. Pascal’s constants for diamagnetic corrections were taken from the literature.^143^ Electrochemical measurements were performed with a Pine Research Inc. WaveDriver potentiostat; more details are provided in the stand-alone electrochemistry section below. Low-resolution electrospray ionization mass spectrometry (LR-ESI-MS) data were collected using a PerkinElmer Sciex API I Plus quadrupole mass spectrometer or a Bruker Esquire 3000 plus ion trap mass spectrometer. Elemental microanalysis for C, H, and N was performed by Midwest Microlab in Indianapolis, IN.

### Synthesis

#### (Et_4_N)­(S-*p*-CF_3_–Ph)

A batch of 4-(trifluoromethyl)­benzenethiol (1.038 g, 5.826 mmol) and sodium metal (0.135 g, 5.87 mmol) were mixed in ∼5 mL of MeOH to afford a gas-evolving, pale-yellow solution. The mixture was stirred for 10 min at RT while removing the headspace gas by applying an occasional vacuum. The heterogeneous mixture became homogeneous and pale-yellow in color after 10 min of stirring. To this solution was added a 3–4 mL MeOH solution of Et_4_NCl (0.966 g, 5.83 mmol). After 30 min of stirring, the solvent was removed to afford a light-yellow oil. To this oil was added ∼5 mL of THF and the mixture was stirred for 10 min. The white insoluble solid was removed by filtering over Celite and the bright yellow THF filtrate was concentrated to an oil. After 30 min of stirring in 5 mL of Et_2_O at RT, a pale-yellow solid formed that was filtered off, washed with Et_2_O (3 × 3 mL), and dried under vacuum to afford the product (1.084 g, 3.526 mmol, 61%). FTIR (KBr matrix), ν_max_ (cm^–1^): 1328 (s, ν_CF_), 1088 (s, ν_CF_). ^1^H NMR (400 MHz, CD_3_CN, δ from protio solvent): 7.24 (d, 2H, *J* = 8.1 Hz), 6.93 (d, 2H, *J* = 8.2 Hz), 3.16 (q, 10H, *J* = 7.3 Hz), 1.17 (t, 15H, *J* = 7.3 Hz). ^13^C­{^1^H} NMR (100 MHz, CD_3_CN, δ from solvent signal): 169.3 (s), 134.2 (s), 127.4 (q, *J* = 268.1 Hz), 123.8 (q, *J* = 3.8 Hz), 117.9 (q, *J* = 31.3 Hz), 53.1 (s), 7.8 (s). ^19^F NMR (376 MHz, CD_3_CN, δ from CFCl_3_): −60.86. LR-ESI-MS (*m*/*z*): [M-Et_4_N]^−^ calcd for C_7_H_4_F_3_S (relative abundance), 177.0 (100.0), 178.0 (8.4), 179.0 (4.8); found, 177.0 (100.0), 178.0 (8.8), 179.0 (5.3). Mp: 56–58 °C.

#### (Et_4_N)_2_[Ni­(S-*p*-CF_3_–Ph)_4_] (**1**)

A blue MeCN slurry (5 mL) of (Et_4_N)_2_[NiCl_4_] (0.097 g, 0.21 mmol) was added dropwise to a pale-yellow MeCN solution (2 mL) of (Et_4_N)­(S-*p*-CF_3_–Ph) (0.269 g, 0.875 mmol). An immediate color change to dark-red occurred upon addition of the Ni^2+^ salt. After stirring at RT for 1 h, the solvent was removed in vacuo resulting in a sticky dark-red material. This residue was stirred in 10 mL of THF, and the solution immediately became dark-red in color with the appearance of a pale solid, determined to be Et_4_NCl by ^1^H NMR. After stirring for 30 min, the mixture was filtered over a pad of Celite, and the insoluble material was washed with ∼ 10 mL of THF until the Celite remained colorless. The resulting dark-red filtrate was taken to dryness and ∼5 mL of Et_2_O was added. After 30 min of stirring, the dark-red insoluble solid was isolated by vacuum filtration on a glass-frit, washed with Et_2_O (3 × 3 mL), and dried under vacuum to afford dark-red complex **1** (0.165 g, 0.161 mmol, 77%). FTIR (KBr matrix), ν_max_ (cm^–1^): 1324 (s, ν_CF_), 1059 (s, ν_CF_). ^1^H NMR (400 MHz, CD_3_CN, δ from protio solvent): 22.25 (s, 6H), 2.94 (q, 16H, *J* = 7.2 Hz), 1.07 (m, 25H), 0.99 (br s, 4H); a minor amount of a diamagnetic species is evident from peaks at ∼7.1 and 7.8 ppm, discussed in the main text. ^19^F NMR (376 MHz, CD_3_CN, δ from CFCl_3_): −18.96 (complex **1** signal), −62.00 (diamagnetic species signal). UV–vis in the presence of 50 mol-equiv (Et_4_N)­(S-*p*-CF_3_–Ph) (MeCN, 25 °C), λ_max_, nm (ε, M^–1^ cm^–1^): 720 (580), 680 (600), 508 (3800), 465 (3400). LR-ESI-MS (*m*/*z*): [M-SR-2Et_4_N]^−^ calcd for C_21_H_12_F_9_S_3_Ni (relative abundance), 588.9 (100.0), 589.9 (25.3), 590.9 (55.1), 591.9 (15.0), 592.9 (13.2); found, 589.0 (100.0), 589.8 (25.1), 590.9 (58.8), 591.8 (14.7), 592.8 (14.9); [M-Et_4_N]^−^ calcd for C_36_H_36_F_12_S_4_NNi (relative abundance), 896.1 (100.0), 897.1 (42.9), 898.1 (65.6), 899.1 (27.0), 900.1 (19.5), 901.1 (6.9); found, 895.9 (100.0), 896.9 (51.4), 897.9 (65.7), 898.9 (31.4), 899.8 (20.0), 900.6 (8.6); [2M-SR-2Et_4_N]^−^ calcd for C_35_H_20_F_15_S_6_Ni_2_ (relative abundance), 1000.9 (92.4), 1001.9 (38.9), 1002.9 (100.0), 1003.9 (42.7), 1004.9 (50.9), 1005.9 (20.6), 1006.9 (17.7), 1007.9 (6.7), 1008.9 (4.6), 1009.9 (1.7); found, 1001.0 (98.7), 1001.9 (47.7), 1002.8 (100.0), 1003.8 (40.9), 1004.8 (52.3), 1005.8 (22.7), 1006.7 (15.9), 1007.7 (6.8), 1008.7 (4.5), 1009.7 (2.3). Solid-state μ_eff_ = 3.47 μ_B_ at 25 °C. Solution-state μ_eff_ = 2.75 μ_B_; (+20 mol-equiv (Et_4_N)­(S-*p*-CF_3_–Ph)) μ_eff_ = 3.05 μ_B_; both recorded at 25 °C in MeCN. Anal. Calcd for C_44_H_56_F_12_N_2_S_4_Ni: C, 51.42; H, 5.49; N, 2.73. Found: C, 51.04; H, 5.49; N, 2.69.

#### (Et_4_N)_2_[Zn­(S-*p*-CF_3_–Ph)_4_] (**2**)

The synthesis of complex **2** was performed in a manner nearly identical to **1** with the exception of using the following reagents: (Et_4_N)­(S-*p*-CF_3_–Ph) (0.135 g, 0.439 mmol) in 5 mL of MeCN and (Et_4_N)_2_[ZnCl_4_] (0.049 g, 0.11 mmol) in 5 mL of MeCN. No significant color change was observed after the addition of the Zn^2+^ salt to the thiolate solution. Workup was similar to **1**, and complex **2** was isolated as a white solid (0.102 g, 0.0986 mmol, 90%). FTIR (KBr matrix), ν_max_ (cm^–1^): 1323 (s, ν_CF_), 1090 (vs, ν_CF_). ^1^H NMR (400 MHz, CD_3_CN, δ from protio solvent): 7.64 (d, 8H, *J* = 8.0 Hz), 7.09 (d, 8H, *J* = 8.0 Hz), 3.13 (q, 20H, *J* = 7.3 Hz), 1.17 (m, 28H, *J* = 7.3 Hz). ^19^F NMR (376 MHz, CD_3_CN, δ from CFCl_3_): −61.69. ^13^C­{^1^H} NMR (100 MHz, CD_3_CN, δ from solvent signal): 156.8 (s), 133.7 (s), 126.6 (q, *J* = 269.7 Hz), 124.3 (q, *J* = 3.9 Hz), 122.2 (q, *J* = 31.2 Hz), 53.1, 7.7 (s). LR-ESI-MS (*m*/*z*): [M-SR-2Et_4_N]^−^ calcd for C_21_H_12_F_9_S_3_Zn (relative abundance), 594.9 (100.0), 595.9 (25.3), 596.9 (74.0), 597.9 (26.5), 598.9 (51.3), 599.9 (13.4), 600.9 (8.6); found, 595.2 (100.0), 596.1 (21.9), 597.0 (74.7), 597.9 (27.9), 598.9 (49.8), 599.9 (12.9), 600.9 (8.0). Anal. Calcd for C_44_H_56_F_12_N_2_S_4_Zn·CH_3_CN: C, 51.37; H, 5.53; N, 3.91. Found: C, 52.17; H, 5.40; N, 3.90.

#### Attempted Synthesis of (Et_4_N)_2_[Ni_2_(S-*p*-CF_3_–Ph)_6_] (**3**)

To a dark-red MeCN solution (3 mL) of **1** (0.090 g, 0.088 mmol) was added Me_3_OBF_4_ (0.013 g, 0.088 mmol) dropwise at RT. The initial addition of Me_3_OBF_4_ resulted in a solution color change to brown; this color became darker after complete addition of the reagent. No further changes were observed to the dark-brown solution after stirring for 2 h. Vacuum distillation of the solvent resulted in a dark-brown solid. To this solid was added ∼3 mL of 2-MeTHF and the reaction flask was stored in a −20 °C freezer for 30 min. The solution was next filtered over a Celite-packed frit. The Celite retained a red-solid (0.054 g, 0.053 mmol) confirmed to be unreacted **1** by ^1^H/^19^F NMR. After taking the dark-brown filtrate to dryness, 5 mL of Et_2_O was added, and the mixture was stirred for 1 h to yield a dark-brown solid suspended in solution. This solid was isolated by vacuum filtration, rinsed with Et_2_O (3 × 3 mL), and dried under vacuum to afford 0.023 g of a mixture of *S*,*S*-bridged complexes. FTIR (KBr matrix), ν_max_ (cm^–1^): 1324 (s, ν_CF_), 1086 (s, ν_CF_). UV–vis (MeCN, 25 °C), λ_max_, nm: 630 (sh), 456 (br sh). ^1^H NMR (400 MHz, CD_3_CN, δ from protio solvent): 21.87 (s, 0.24H), 7.77 (d, 3H, *J* = 7.9 Hz), 7.64 (d, 6H, *J* = 8.0 Hz), 7.55 (br s, 4H), 7.12 (d, 3H, *J* = 8.0 Hz), 7.06 (d, 10H, *J* = 8.2 Hz), 6.82, (br, 1H), 3.15 (q, 16H, *J* = 7.3 Hz), 1.20 (m, 24 H, *J* = 7.3 Hz). ^19^F NMR (376 MHz, CD_3_CN, δ from CFCl_3_): −19.93, −61.93, −62.35, −62.47, −63.30. LR-ESI-MS (*m*/*z*): [M-SR-2Et_4_N]^−^ calcd for C_35_H_20_F_15_S_5_Ni_2_ (relative abundance), 1000.9 (92.4), 1001.9 (38.9), 1002.9 (100.0), 1003.9 (42.7), 1004.9 (50.9), 1005.9 (20.6), 1006.9 (17.7), 1007.9 (6.6); found, 1001.2 (91.1), 1002.2 (44.8), 1003.1 (100.0), 1004.1 (43.7), 1005.0 (45.2), 1006.0 (19.5), 1007.0 (14.3), 1008.0 (5.3); [2M-2Et_4_N+Ni]^−^ calcd for C_84_H_48_F_36_S_12_Ni_5_ (relative abundance), 1206.6 (23.3), 1207.3 (23.5), 1207.8 (69.1), 1208.3 (63.1), 1208.8 (100.0), 1209.3 (83.4), 1209.8 (84.2), 1210.3 (71.3), 1201.8 (65.3), 1211.3 (45.4), 1211.8 (35.7), 1212.3 (22.9), 1212.8 (16.1), 1213.3 (9.6); found, 1206.0 (21.2), 1207.5 (21.9), 1208.0 (67.2), 1208.5 (61.7), 1209.0 (100.0), 1209.5 (86.5), 1210.0 (96.6), 1210.5 (71.7), 1211.0 (65.9), 1211.5 (48.1), 1212.0 (35.7), 1212.5 (25.6), 1213.0 (16.6), 1213.5 (10.9). Anal. Calcd for C_58_H_64_F_18_N_2_S_6_Ni_2_: C, 48.35; H, 4.48; N, 1.94. Found: C, 44.84; H, 3.54; N, 1.98. *Note*: The lack of a suitable fit to the CHN analysis is due to mixed and inseparable products.

#### (Et_4_N)_2_[Ni­(S-*p*-CF_3_–Ph)_3_(NO)] (**4**)

To a dark-red MeCN solution (4 mL) of **1** (0.062 g, 0.060 mmol) was slowly added a green MeCN solution (3 mL) of Ph_3_CSNO (0.020 g, 0.065 mmol). Upon addition of Ph_3_CSNO, the dark-red solution took on a green color and, as more was added, the solution became dark-green. After stirring the solution for 30 min at RT, 70 mL of Et_2_O was added, and the mixture was placed in a −20 °C freezer. After cooling for 12 h, a dark-green solid appeared in solution, which was filtered off by vacuum filtration on a glass-frit, washed with Et_2_O (3 × 3 mL), and dried under vacuum to afford **4** as a green powder (0.028 g, 0.032 mmol, 53%). FTIR (KBr matrix), ν_max_ (cm^–1^): 1683 (m, ν_NO_), 1322 (s, ν_CF_), 1084 (s, ν_CF_). FTIR (CaF_2_ windows, MeCN), ν_max_ (cm^–1^): 1772 (ν_NO_), 1683 (ν_NO_). UV–vis (MeCN, 25 °C), λ_max_, nm (ε, M^–1^ cm^–1^): 799 (br, 780), 425 (sh, 1300), 373 (sh, 4700). ^1^H NMR (400 MHz, CD_3_CN, δ from protio solvent): 7.73 (d, 6H, *J* = 8.1 Hz), 7.20 (d, 6H, *J* = 8.1 Hz), 3.15 (q, 19H, *J* = 7.3 Hz), 1.20 (t, 28H, *J* = 7.3 Hz). ^19^F NMR (376 MHz, CD_3_CN, δ from CFCl_3_): −61.51 (s). LR-ESI-MS (*m*/*z*): [M-SR-2Et_4_N]^−^ calcd for C_14_H_8_F_6_S_2_NONi (relative abundance), 441.9 (100.0), 442.9 (17.2), 443.9 (49.2), 444.9 (9.9), 445.9 (10.1); found, 442.1 (100.0), 443.1 (17.5), 444.0 (49.2), 445.0 (10.2), 446.0 (10.0). Anal. Calcd for C_37_H_52_F_9_N_3_S_3_ONi: C, 50.46; H, 5.95; N, 4.77. Found: C, 46.85; H, 5.40; N, 4.68. *Note*: The lack of a suitable fit to the CHN analysis may be due to potential lability of the nitrosyl ligand.

### Speciation Studies

#### Reaction of **1** with Stoichiometric HBF_4_·Et_2_O

To a 3 mL MeCN solution of **1** (0.076 g, 0.074 mmol) was added HBF_4_·Et_2_O (0.014 g, 0.086 mmol; 1.2 mol-equiv with respect to **1**; p*K*_a_ = 1.8 in MeCN^98^). Immediately upon addition of acid, the solution changed color from dark-red/brown, to brown, and finally to purple within 1 min, and a dark precipitate appeared. The mixture was stirred for 1 h at RT and then placed in a −20 °C freezer for 30 min. The dark-purple solid was isolated by vacuum filtration, washed with cold MeCN (3 × 3 mL), and dried on a vacuum line to afford 0.034 g of what is assigned as polymeric [Ni­(S-*p*-CF_3_–Ph)_2_]_n_ material. FTIR (KBr matrix), ν_max_ (cm^–1^): 1327 (s, ν_CF_), 1063 (s, ν_CF_). UV–vis (MeCN, 25 °C), λ_max_, nm: 510, 462 (sh). ^1^H NMR (400 MHz, DMSO-*d*_6_, δ from residual deuterated solvent): 22.08 (s), 7.69 (s), 7.51 (d), 7.40 (s), 7.10 (d), 7.03 (d), 3.24 (s), 2.07. ^19^F NMR (376 MHz, DMSO-*d*_6_): −14.37, −16.87, −60.14, −60.65, −61.76.

#### Reaction of **1** with Excess AcOH

To a 2 mL MeCN solution of **1** (0.073 g, 0.071 mmol) was added a 1 mL MeCN solution containing AcOH (0.106 g, 1.77 mmol; 25 mol-equiv with respect to **1**; p*K*_a_ = 23.5 in MeCN^61^). Similar to reaction with stoichiometric HBF_4_·Et_2_O, immediately upon addition of AcOH, the solution changed color from dark red-brown to brown to purple within 1 min, and a dark precipitate appeared. After 1 h of stirring, the precipitate was isolated by vacuum filtration, washed with cold MeCN (3 × 3 mL), and dried on a vacuum line to afford 0.032 g of what is assigned as polymeric [Ni­(S-*p*-CF_3_–Ph)_2_]*_n_* material. Spectroscopic characterization (IR, UV–vis, ^1^H, and ^19^F NMR) of this product is nearly identical to the product isolated from the reaction of **1** and HBF_4_·Et_2_O (1:1).

#### Titration of **1** with AcOD-*d*_4_ (NMR)

*Without added thiolate*: A 56 mM solution of **1** (0.019 g, 0.018 mmol) in 0.32 mL of CD_3_CN was prepared in a J-Young NMR tube that was sealed with a rubber septum and secured with (in this order) parafilm, electrical tape, and copper wire. Separately, an 8.7 M solution of AcOD-*d*_4_ was made in CD_3_CN (total volume = 0.40 mL; 0.20 mL AcOD-*d*_4_, 3.5 mmol; 0.20 mL CD_3_CN) and stored in a septum-capped vial. After recording the ^1^H and ^19^F NMR of **1**, a 10 μL aliquot (0.087 mmol; 4.8 mol-equiv with respect to **1**) of the AcOD-*d*_4_/CD_3_CN solution was injected into the NMR tube via gastight syringe. The tube was gently shaken three times and sat undisturbed for 1 min, after which the NMR spectrum of the mixture was recorded. This procedure was repeated for each addition of AcOD-*d*_4_/CD_3_CN until no change was observed. The speciation of **1** with added AcOD-*d*_4_ was assessed by comparing the integration of the peak at ∼20 ppm (*m*-H of **1**) after each AcOD-*d*_4_ addition to that of the Et_4_N^+^ counterion. *With added thiolate*: The same procedure was performed using a 34 mM solution of **1** (0.017 g, 0.017 mmol) containing 19 mol-equiv of (Et_4_N)­(S-*p*-CF_3_–Ph) (0.098 g, 0.32 mmol) in 0.50 mL of CD_3_CN. Aliquots of AcOD-*d*_4_ (20 μL, 0.35 mmol; 21 mol-equiv with respect to **1**) were injected as described above. The speciation of **1** was measured in a manner similar to the experiments without thiolate (see above) considering integration of the Et_4_N^+^ counterion.

#### Quantification of **1**/Excess Thiolate with AcOH (UV–vis)

A 2.50 mM stock solution of **1** (0.010 g, 0.010 mmol) was prepared in 4.00 mL of MeCN; 0.12 mL of this stock was added to a vial containing 1.88 mL of MeCN to yield a final concentration of 0.15 mM for **1**. This solution was then used to dissolve a solid batch of (Et_4_N)­(S-*p*-CF_3_–Ph) (0.002 g, 0.007 mmol, 23 mol-equiv with respect to **1**). This solution was transferred to a quartz cuvette and its UV–vis spectrum recorded. To the cuvette was next added 20 μL of a 0.30 M AcOH solution in MeCN (20 mol-equiv with respect to **1**) and the spectrum recorded. The amount of **1** remaining was calculated by taking the ratio of absorbance values at λ = 508 nm described by eq 1:

i $$\frac{{Abs}_{508\;nm}\left(1+23\;SR+20\;AcOH\right)}{{Abs}_{508\;nm}\left(1+23\;SR\right)}\times 100\%$$

#### Speciation of **1** by ^19^F NMR in CD_3_CN

A 2.00 mM stock solution of **1** (0.004 g, 0.004 mmol) was prepared in 2.00 mL of CD_3_CN containing PhF (10 μL). A 400 μL portion of the **1**/PhF (0.0008 mmol **1**) CD_3_CN solution was placed in an NMR tube and the ^19^F NMR spectrum was recorded. The relative concentrations of **1** and *S*,*S*-bridged complexes such as **3** were calculated by the ratio of the integrated peaks associated with **1** (δ = −19.22 ppm) and the bridged complexes (peaks centered at δ = −62.00 ppm), normalized to the PhF integration. To calculate the relative amounts of **1** and *S*,*S*-bridged complexes upon addition of thiolate, (Et_4_N)­(S-*p*-CF_3_–Ph) (0.013 g, 0.042 mmol; 53 mol-equiv with respect to **1**) was added to the 400 μL CD_3_CN solution of **1**/PhF in the NMR tube and the ^19^F NMR spectrum was recorded, see Figure S46. To calculate the relative amounts of **1** and bridged complexes (e.g., **3**) integration of the ^19^F peak of **1** was taken relative to the integration of the peak assigned to PhF and the percentage of **1** was determined by eq 2:

ii $$\frac{\int \delta_{1+SR}}{\int \delta_{1}+\int \delta_{s,s‐\mathrm{brg}}}\times 100\%$$

where ∫δ_1_ + SR is the integration value of the peak at ∼−19 ppm (**1**) in the presence of excess (Et_4_N)­(S-*p*-CF_3_–Ph); ∫δ_1_ and ∫δ_s,s‑brg_ are the integration values for the peaks of **1** (−19 ppm) and *S*,*S*-bridged complexes (∼−62 ppm), respectively, in the absence of (Et_4_N)­(S-*p*-CF_3_–Ph), i.e., as-isolated **1**.

#### Quantification of **1**/Excess Thiolate by ^19^F NMR in CD_3_CN with AcOD-*d*_4_

A 400 μL aliquot of the same CD_3_CN stock of **1**/PhF described above was placed in an NMR tube and to this solution was added (Et_4_N)­(S-*p*-CF_3_–Ph) (0.0050 g, 0.016 mmol; 20 mol-equiv with respect to **1**) and the ^19^F NMR was recorded. Separately, a 1.57 M CD_3_CN stock solution of AcOD-*d*_4_ (0.09 mL, 1.57 mmol) in 0.91 mL of MeCN (total volume = 1.00 mL) was prepared. A 10 μL aliquot of the AcOD-*d*_4_ stock (0.016 mmol, 20 mol-equiv with respect to **1**) was added to the NMR tube containing a CD_3_CN solution of **1**/PhF/(Et_4_N)­(S-*p*-CF_3_–Ph) and the ^19^F NMR spectrum measured. Integration of the ^19^F peak of **1** (−19.0 ppm) was taken relative to the integration of the peak assigned to PhF and the percent of **1** was determined by eq 2.

#### Speciation of *S*,*S*-Bridged Complexes by NMR in CD_3_CN with (Et_4_N)­(S-*p*-CF_3_–Ph)

A mixture of *S*,*S*-bridged complexes including **3** (from isolated products of methylation reaction of **1**) (0.016 g) in 0.34 mL of CD_3_CN was prepared in a J-Young NMR tube that was sealed with a rubber septum and secured with (in this order) parafilm, electrical tape, and copper wire. Separately, a 0.44 M solution of (Et_4_N)­(S-*p*-CF_3_–Ph) (0.026 g, 0.085 mmol) was made in 0.2 mL of CD_3_CN and stored in a septum-capped vial. After recording the ^1^H and ^19^F NMR of the thiolate-bridged complex mixture, a 10 μL aliquot (0.0044 mmol) of the CD_3_CN solution containing (Et_4_N)­(S-*p*-CF_3_–Ph) was added to the solution in the NMR tube. The tube was shaken, and the spectrum was recorded after 1 min.

### Electrochemical Measurements

#### Electrochemistry

Cyclic voltammetry measurements were performed with a WaveDriver 40 bipotentiostat/galvanostat from Pine Research Instrumentation Inc. using an Ag/Ag^+^ pseudoreference electrode (Ag wire immersed in 0.25 M ^*n*^Bu_4_NPF_6_ electrolyte in MeCN), Pt-wire counter electrode (directly submerged in solution), and a stationary glassy carbon diskworking electrode (3 mm outer diameter). The potentiostat was interfaced with a PC using the Aftermath software. Measurements were collected at ambient temperature using ∼2.0 mM analyte in MeCN containing 0.25 M ^*n*^Bu_4_NPF_6_ as the supporting electrolyte under an N_2_ atmosphere. Analyte potentials were referenced against an external ferrocene standard under identical conditions. Purity of the electrolyte in the solvent medium was confirmed over the available electrochemical window through scans taken prior to the addition of analyte or external reference. Acid titration experiments for the HER were performed by addition of MeCN aliquots of a ∼4.2 mM stock of AcOH in MeCN. AcOH was added in 10 μL aliquots (∼2 mol-equiv with respect to complex). The solution was stirred for 20 s and sat undisturbed for an additional 10 s prior to measurement. The working electrode was cleaned between scans by washing with solvent, polishing on a felt cloth with a 0.3 μm alumina suspension from Allied High-Tech Products, and sonicating for 30 s in DI water. All CV are plotted according to the US convention.

#### Determination of Overpotential

The overpotential (η) was calculated using eq 3, which was developed for homogeneous electrocatalysis of the HER.^144^

iii $$\eta =|E_{H^{+}/H_{2}}^{{}^{\circ}}-E_{cat/2}|$$

*E°*_H+/H2_ is the standard reduction potential for the 2H^+^/H_2_ couple of AcOH in MeCN taking into account homoconjugation.^110^ *E°*_H+/H2_ was calculated to be −1.27 V vs Fc^+^/Fc in MeCN. *E*_cat/2_ is the potential for the HER catalyzed by the reported complexes at half the maximum of the catalytic current (*i*_c_).^144^

#### Determination of *k*_obs_

Observed rate constants (*k*_obs_) for HER were calculated under pseudo first-order conditions where the [H^+^] is high enough that it is not depleted during catalysis, commonly designated as the acid-independent regime. For **1**/20 mol-equiv thiolate ([**1**] = 2.0 mM), this condition was reached at 18 mol-equiv (∼36 mM) of AcOH. Under our experimental conditions, *k*_obs_ was defined as being equivalent to the maximum TOF or TOF_max_; *i*_c_/*i*_p_ value taken at 20 mol-equiv (∼40 mM) of AcOH with *k*_obs_ estimated according to eq 4.^118,145^

iv $$\frac{i_{c}}{i_{p}}=\frac{n}{0.4463}\sqrt{\frac{\mathrm{RT}k_{\mathrm{obs}}}{\mathrm{Fv}}}$$

In eq 4 *i*_c_ is the current under catalytic conditions, *i*_p_ is the current in the absence of acid, *T* is temperature (298 K), *n* is number of electrons in the catalytic process (*n* = 2 for HER), *v* is the scan rate (0.100 V/s), *R* is the gas constant, and *F* is the Faraday constant. We attempted to use foot-of-the-wave analysis (FOWA) to estimate *k*_obs_, but FOWA was not suitable for **1** because of the deviations in *i*_c_ at the onset of the catalytic wave and irreversibility of the Ni^2+/1+^ couple.

#### Quantifying Amount of **1** Remaining After CV Titration

After completing an electrocatalytic HER experiment of **1**/20 mol-equiv (Et_4_N)­(S-*p*-CF_3_–Ph) with AcOH, a 150 μL aliquot of the solution was taken from the electrochemical cell and placed in a quartz cuvette containing 2.00 mL MeCN and the UV–vis spectrum was recorded. Complex **1** was quantified using eq 5:

v $$\frac{\exp.\;\mathrm{Abs}.}{\left[1\right]\times \varepsilon_{508\;nm}}\times 100\%$$

where [**1**] = concentration of **1** (0.14 mM), ε_508 nm_ = molar absorptivity value (3800 M^–1^ cm^–1^), and exp. Abs. = experimental absorbance value obtained.

#### Determination of the Kinetic Isotope Effect (KIE)

Using eq 4, *k*_obs_ was calculated for HER experiments using CD_3_COOD (AcOD-*d*_4_) under the same conditions as described for AcOH (*i*_c_/*i*_p_ values taken at 20 mol-equiv (∼40 mM) of AcOD-*d*_4_). The ratio of *k*_obs_ with AcOH to *k*_obs_ with AcOD-*d*_4_ affords a KIE of 1.62.

#### Catalyst Concentration Dependence

A 100 mM stock solution of **1** (0.052 g, 0.051 mmol) containing 20 mol-equiv of (Et_4_N)­(S-*p*-CF_3_–Ph) (0.310 g, 1.01 mmol) was prepared in 0.50 mL of MeCN. To this solution was added 60 μL of a 4.2 M solution of AcOH (prepared by adding 0.12 mL (2.1 mmol) of AcOH to 0.38 mL of MeCN; total volume = 0.50 mL) resulting in a new 90 mM (with respect to **1**) stock solution of **1**/20SR^–^/5AcOH in 0.56 mL of total MeCN. A 62 μL aliquot of this solution was added to an electrochemical cell containing 10.00 mL of 0.25 M ^*n*^Bu_4_NPF_6_ electrolyte in MeCN. The solution was stirred for 20 s and sat undisturbed for 10 s prior to measurement. This procedure was repeated totaling five aliquots. The working electrode was cleaned between each scan as described prior. A linear relationship between *i*_c_ and [**1**] is indicative of a first-order dependence based on eq 6.^146^

vi $$i_{c}=\mathrm{nFA}\left[\mathrm{cat}\right]\sqrt{D\left(k\left[H^{+}\right]\right)}$$

#### AcOH Concentration Dependence

The order with respect to AcOH was calculated utilizing eq 6. A linear relationship between *i*_c_ and √[AcOH] is indicative of a first-order dependence, and a linear relationship between *i*_c_ and [AcOH] indicates a second-order dependence,^146^ Where *i*_c_ was taken at nonsaturating conditions (less than ∼30 mM AcOH) from the electrochemical AcOH titration experiments for the determination of *k*_obs_ (see above for details).

### X-ray Diffraction/X-ray Absorption Measurements

#### X-ray Crystallographic Data Collection and Structure Solution and Refinement

Red crystals of **1** were obtained from slow diffusion of Et_2_O into a saturated solution of **1** in MeCN at −20 °C. Colorless crystals of **2** were grown by slow diffusion of Et_2_O into a saturated MeCN/THF (100:1) solution of **2** at −20 °C for 1 week. The crystals of **1** and **2** were mounted in a 100 and 90 K nitrogen cold stream, respectively, (Cryo Industries low temperature apparatus) on the goniometer head of a Bruker D8 Quest equipped with a Bruker Photon II detector for **1**, and a Bruker D8 Venture Kappa DUO diffractometer equipped with a Bruker Photon 100 CMOS detector for **2**. Data were collected with the use of a Mo Kα (λ = 0.71073 Å) and Cu Kα (λ = 1.54178 Å) microsource for **1** and **2**, respectively. A multiscan absorption correction was applied with the program SADABS.^147^ The structure for **1** and **2** was solved by the dual space method, (SHELXT)^148^ and refined by full-matrix least-squares on F2 (SHELXL-2017).^149^ One fluorine atom (F8) of one CF_3_ group in **1** were located in two positions with ratios of 68:32. The structure for **2** is highly disordered and displays considerable diffuse scattering. The phenyl ring has two positions with two carbons shared; their occupancies are in the ratio of 48:52. The CF_3_ groups were located in three positions with ratios of 48:30:22 and the Et_4_N^+^ is slightly positioned off of the 2-fold axis of Wyckoff position e. A number of PART statements were employed to model the disorder. Further information on the structure determination is available in the deposited CIF, CCDC numbers 2443187 and 2443188 for **1** and **2**, respectively. These data can be obtained free of charge via http://www.ccdc.cam.ac.uk/conts/retrieving.html, or from the Cambridge Crystallographic Data Centre, 12 Union Road, Cambridge CB2 1EZ, UK; fax: (+44) 1223-336-033; or e-mail: deposit@ccdc.cam.ac.uk.

#### X-ray Absorption Spectroscopy (XAS)

XAS data were collected at the Stanford Synchrotron Radiation Lightsource (SSRL) beamline 9–3, which utilizes a Si[220] double-crystal monochromator with an inline mirror for X-ray focusing and for harmonic rejection. Duplicate independent samples were prepared anaerobically inside an MBraun glovebox, with solid samples diluted 3:1 with inert Boron Nitride, then wrapped in Kapton tape. During data collection, samples were maintained at 12 K using a liquid He continuous flow cryostat. Transmission spectra were measured in 0.25 eV increments in the edge region (8325–8370 eV) and 0.05 Å^–1^ increments in the extended X-ray absorption fine structure (EXAFS) region (out to *k* = 13.3 Å^–1^), integrating from 1 to 25 s in a *k* ^3^-weighted manner for a total scan length of approximately 50 min. Data represent the average of three scans. X-ray energy scans were individually calibrated by collecting a Ni-foil absorption spectrum simultaneously with the compound; the first inflection point of the Ni-foil spectrum was assigned to 8333 eV. The pre-edge features were investigated by normalizing the edge data using the EDG_FIT program (R-29). For analysis of the 1s → 3d transition, data was fit with an arctan background + a pseudo-Voigt peak to model the rising edge and 1s → 3d peak, XAS spectra were processed using the Macintosh OSX version of the EXAFSPAK program suite^150^ integrated with the Feff v8 software for theoretical model generation.^151^ XAS simulations followed a pre-existing published strategy.^152^

### Theoretical Studies

#### Computational Details

Density functional theory (DFT) calculations were performed with the ORCA electronic structure package, version 4.2.1. Geometry optimization and frequency analysis were performed using the B3LYP functional^76,153^ employing the RIJCOSX approximation^154^ and Grimme’s D3­(BJ) dispersion correction^155,156^ with coordinates from the crystal structures of **1** and **2** and the coordinates from the crystal structure of (Et_4_N)­[Ni_2_(S-*p*-Cl-Ph)_6_][Bibr ref57] with the replacement of Cl with CF_3_, referred to as complex **3**. The triple-ζ basis set def2-TZVPP^157,158^ was used for geometry optimization and frequency analysis with an automatically constructed auxiliary basis set on all atoms. No imaginary frequencies were found. Single point energy (SPE) calculations were performed on the optimized structures using the B3LYP functional^76,153^ employing the RIJCOSX approximation.^154^ The triple-ζ basis set def2-TZVPP^157,158^ was used for all atoms with the matching auxiliary triple-ζ basis set def2-TZVPP/J. The conductor-like polarizable continuum model (CPCM^159^) was utilized to model the solvent environment of MeCN (ε = 37.50). UCSF Chimera^160^ was used to generate model structures and visualize isosurface plots of MOs with isodensity values of 0.05 a.u. Spin-density plots were visualized with isodensity values of 0.003 a.u.

Absorption spectra for geometry-optimized **1** and **3** were computed using time-dependent functional theory (TD-DFT).^161,162^ The Tamm-Dancoff approximation was performed for the first 80 roots using the B3LYP functional, D3­(BJ) dispersion correction, and RIJCOSX approximations. The def2-SVP basis set was used for all atoms except for Ni, S, and F for which the def2-TZVPP basis set was used. CPCM^159^ was utilized to model the solvent environment of MeCN (ε = 37.50). Vertical excitation energies and molar extinction coefficients for electronic transitions were calculated using the ORCA_MAPSPC module. The absorption spectrum was generated with a Gaussian line width of 2500 cm^–1^. UCSF Chimera^160^ was used to generate model structures and visualize isosurface plots of electron difference density maps with isodensity values of 0.005 a.u.

For mechanistic considerations, all intermediates were computed utilizing the same level of theory as previously described for **1**. The geometry optimizations were performed in the gas phase with FREQ and SPE calculated with CPCM to model the solvent environment of MeCN. The Gibbs free energy of the protonation step (Δ*G*_C‑step_) was calculated with eq 7 utilizing the Gibbs free energy of Et_3_NH^+^ as H^+^ donor and its conjugate base Et_3_N:

vii $$\Delta G_{C‐step}=\Delta G\left(AH+{Et}_{3}N\right)-\Delta G\left(A^{-}+{Et}_{3}NH^{+}\right)$$

Both acid and conjugate base were optimized under the same level of theory as for **1** and frequency analysis was done with CPCM (MeCN). DFT-calculated redox potentials are described elsewhere in the SI and the energy of each reduction step (Δ*G°*) was calculated according to eq 8:

$$\Delta G^{{}^{\circ}}=-23.06\left(n\right)\left(E_{1/2}\right)$$ viii

where *n* is the number of electrons transferred (*n* = 1) and *E*_1/2_ is the compound reduction potential. Transition state calculations were performed utilizing a relaxed surface scan (ScanTS), under the same level of theory as for **1** utilizing CPCM (MeCN), to scan the distances between hydride and proton on coordinated thiolate (2.614-to-0.400 Å in 20 steps). UCSF Chimera^160^ was used to generate model intermediates and transition states. Transition state structures for the intermediates yield only one imaginary frequency.

## Supplementary Material



## Notes

Spectroscopic, X-ray crystallographic, standard electrochemistry, electrocatalytic, and computational details for all compounds (PDF)

## #

Imerys S.A., 43 Quai de Grenelle, Paris 75015, France. Email: amelia.reid@imerys.com

## ∇

Sacred Heart University, Chemistry & Physics Department, 5151 Park Avenue, Fairfield, Connecticut 06825, United States. Email: truongp2@sacredheart.edu.

## ○

Van Andel Institute, 333 Bostwick Avenue NE, Grand Rapids, Michigan 49503, United States. Email: tiara.hinton@vai.org.

## ◆

Medical University of South Carolina (MUSC), Department of Chemistry and Molecular Biology, 171 Ashley Avenue, Charleston, South Carolina 29425, United States. Email: stemmler@musc.edu.

## ⊥

Deceased September 30, 2020.
