# Supplementary material for: Synthesis, Properties, and Electrochemical Proton Reduction of a Homoleptic Tetrathiolato Ni-Site Model of [NiFe]-Hydrogenase
Source: Inorg Chem. 2025 Sep 5;64(45):22308–29. doi: 10.1021/acs.inorgchem.5c03077 (PMC12628296; doi:10.1021/acs.inorgchem.5c03077)
Supplement: Supplementary file 1 [file ic5c03077_si_001.pdf]

Supporting Information for:

Synthesis, Properties, and Electrochemical Proton Reduction of a Homoleptic Tetrathiolato Ni-Site Model of [NiFe]-Hydrogenase

*Benjamin A. Yosen,[†] Amelia G. Reid,[†] Phan T. Truong,[†] Tiara Hinton,[§] Indranil Chakraborty[±], Marilyn M. Olmstead,[‡] Timothy L. Stemmler,[§] and Todd C. Harrop[†,]**

*Email: tharrop@uga.edu

†Department of Chemistry and Center for Metalloenzyme Studies, The University of Georgia, 302 East Campus Road, Athens, GA, 30602, United States

[±]Department of Chemistry and Biochemistry, Florida International University, Miami, FL, 33199, United States

[‡]Department of Chemistry, University of California, Davis, CA, 95616, United States

[§]Department of Pharmaceutical Sciences, Wayne State University, 259 Mack Ave, Detroit, MI, 48201, United States

Table of Contents:

Section	Pages
1. **Standard Characterization**	S3-S29
2. **XRD/XAS**	S30-S33
3. **DFT**	S34-S46
4. **Speciation Studies**	S47-S51
5. **DFT Analysis of Speciation**	S52-S53
6. **Bulk Protonation Studies**	S54-S62
7. **Electrochemical Characterization**	S63-S65
8. **Electrocatalysis**	S66-S71
9. **DFT Computed Mechanism**	S72-S121
10. **References**	S122

1. Standard Characterization of Complexes:

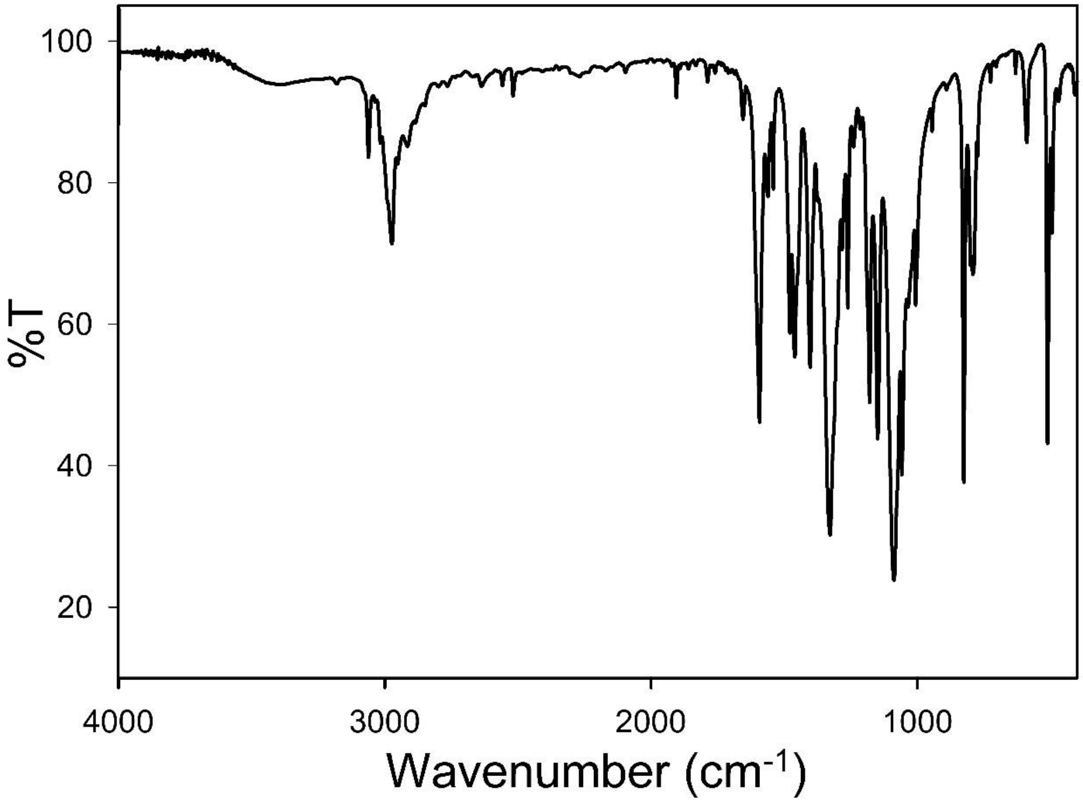

Figure S1. Solid-state FTIR spectrum of (Et_4N)(S-*p*-CF_3-Ph) in a KBr matrix at RT.

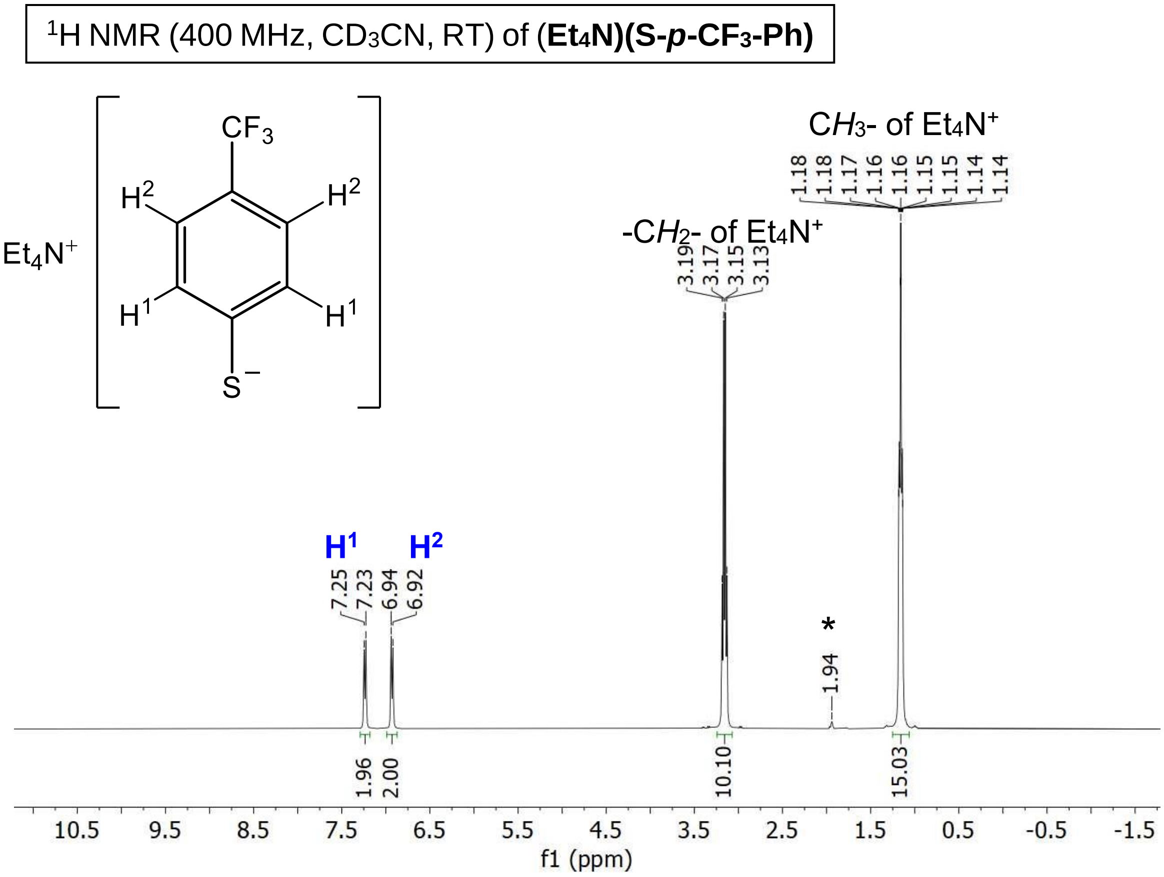

Figure S2. ^{1}H NMR spectrum of $(Et_4N)(S\text{-}p\text{-}CF_3\text{-}Ph)$ in CD_3CN at RT (δ vs. protio signal (*) at 1.94 ppm).

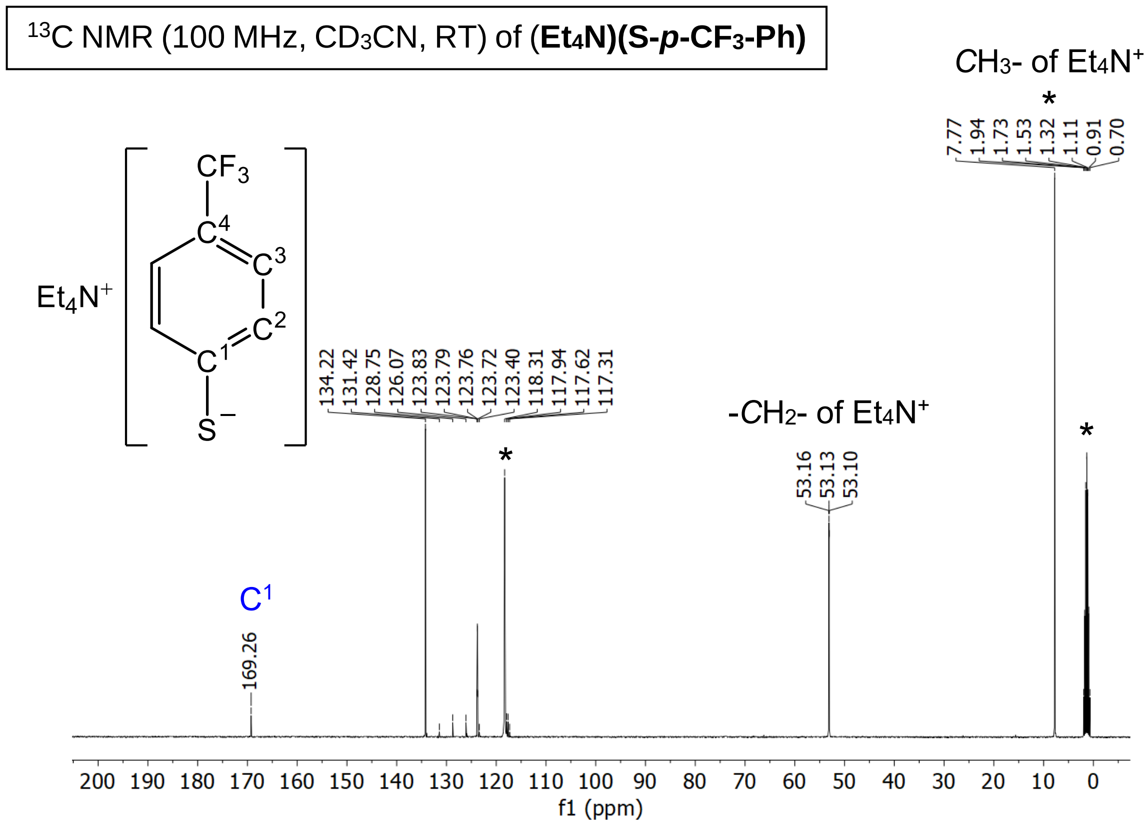

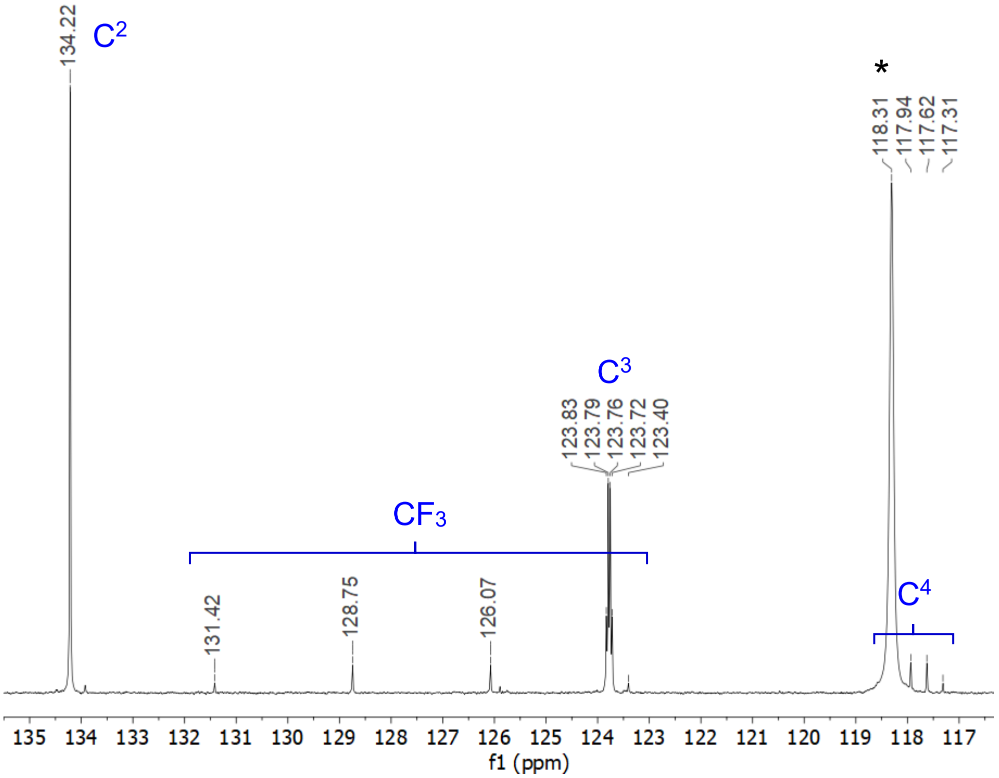

Figure S3. *Top*: ^{13}C NMR spectrum of (Et$_4$N)(S-*p*-CF$_3$-Ph) in CD$_3$CN at RT. *Bottom:* expansion of 117 – 135 ppm region. Solvent signal at 1.32 and 118.31 ppm are indicated with *.

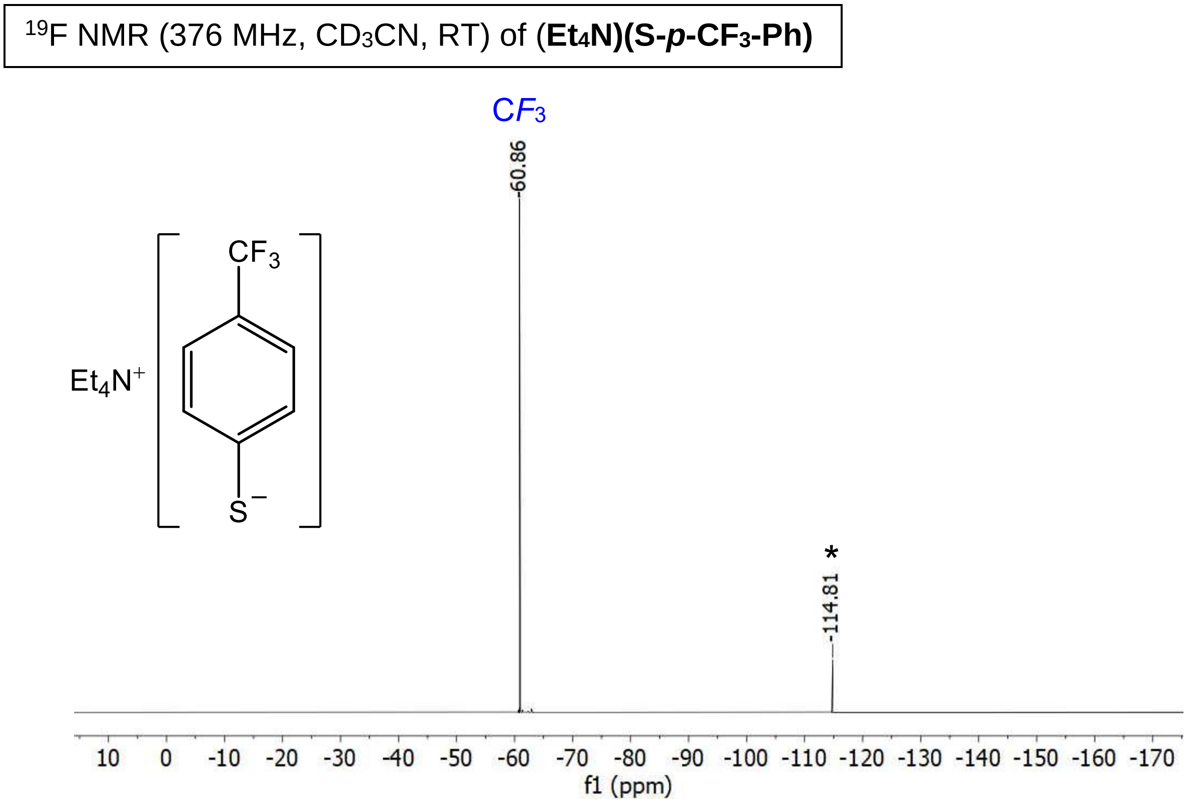

Figure S4. ^{19}F NMR spectrum of (Et_4N)(S-*p*-CF_3-Ph) in CD_3CN at RT. The peak at -114.81 ppm is from PhF added as an internal standard and indicated with *.

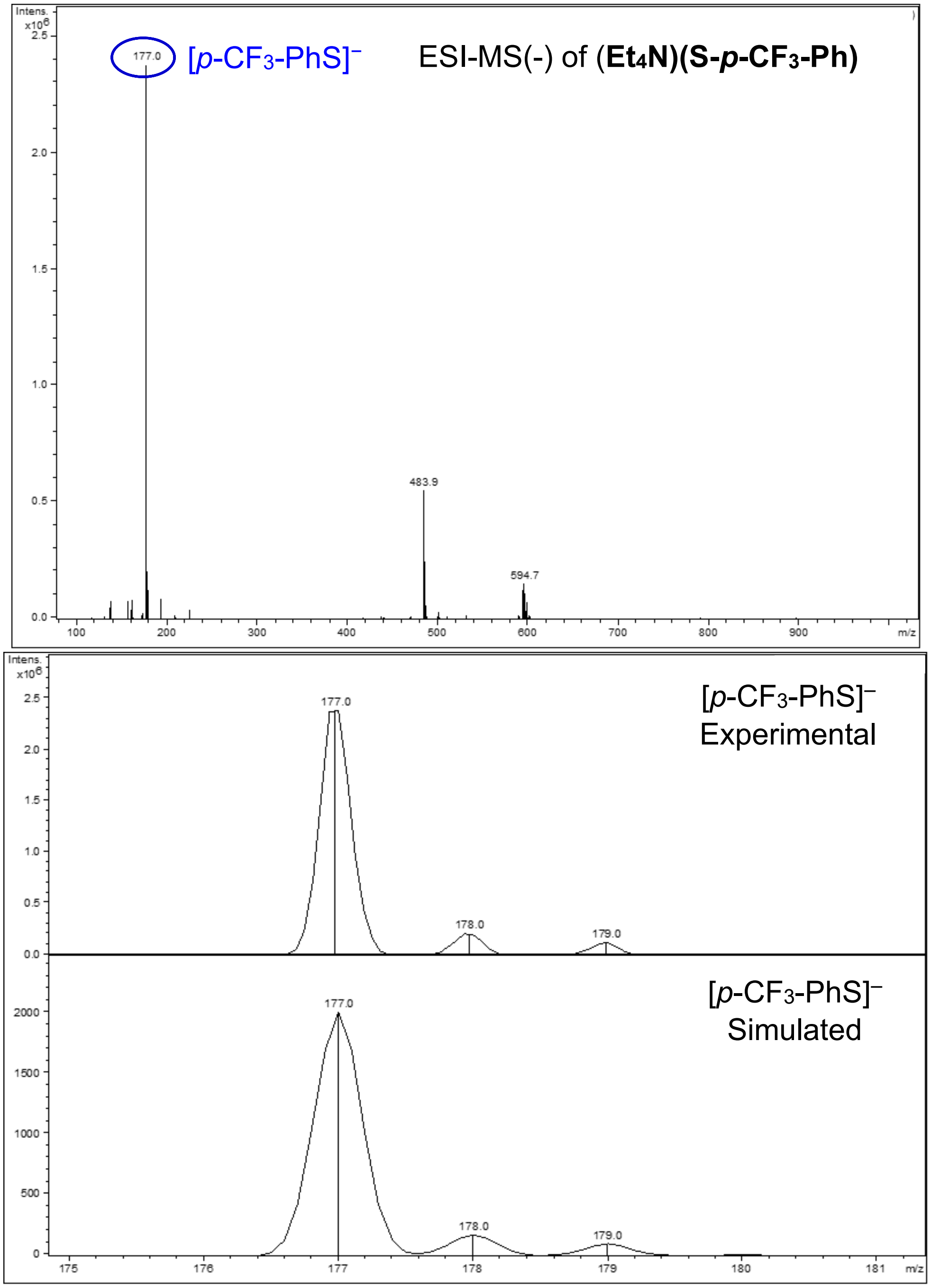

Figure S5. *Top*: Low-resolution ESI-MS(-) of $(Et_4N)(S\text{-}p\text{-}CF_3\text{-}Ph)$ in MeCN. *Bottom*: experimental and simulated theoretical isotopic distribution for *m/z*: 177.0 Other peaks have not been identified as reaction products.

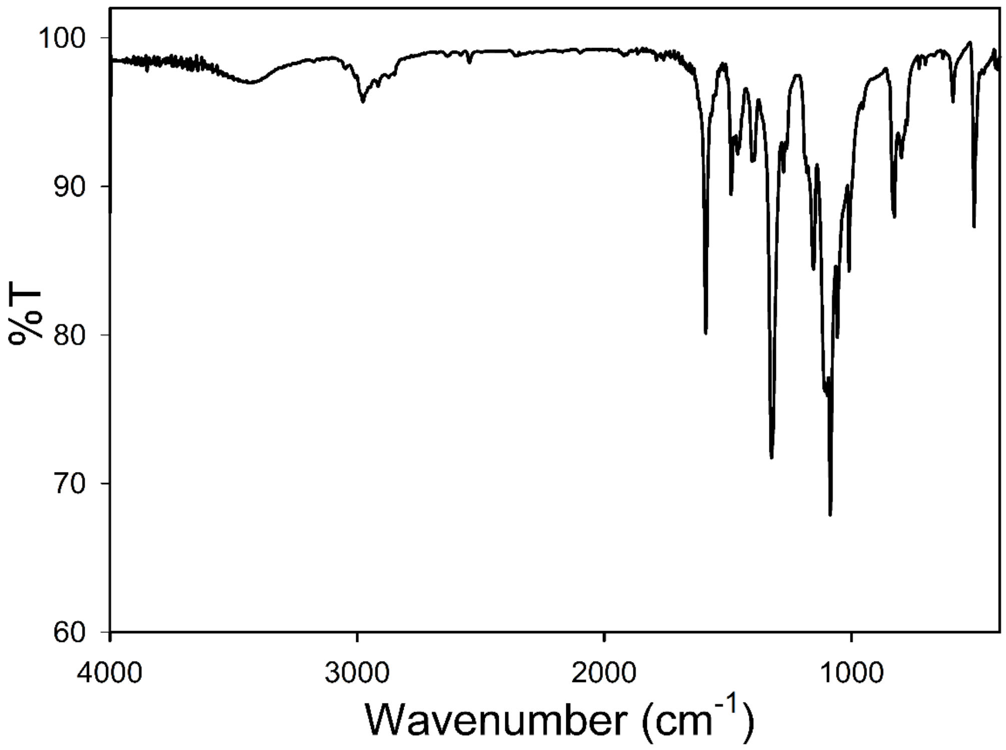

Figure S6. Solid-state FTIR spectrum of $(Et_4N)_2[Ni(S\text{-}p\text{-}CF_3\text{-}Ph)_4]$ (**1**) in a KBr matrix at RT.

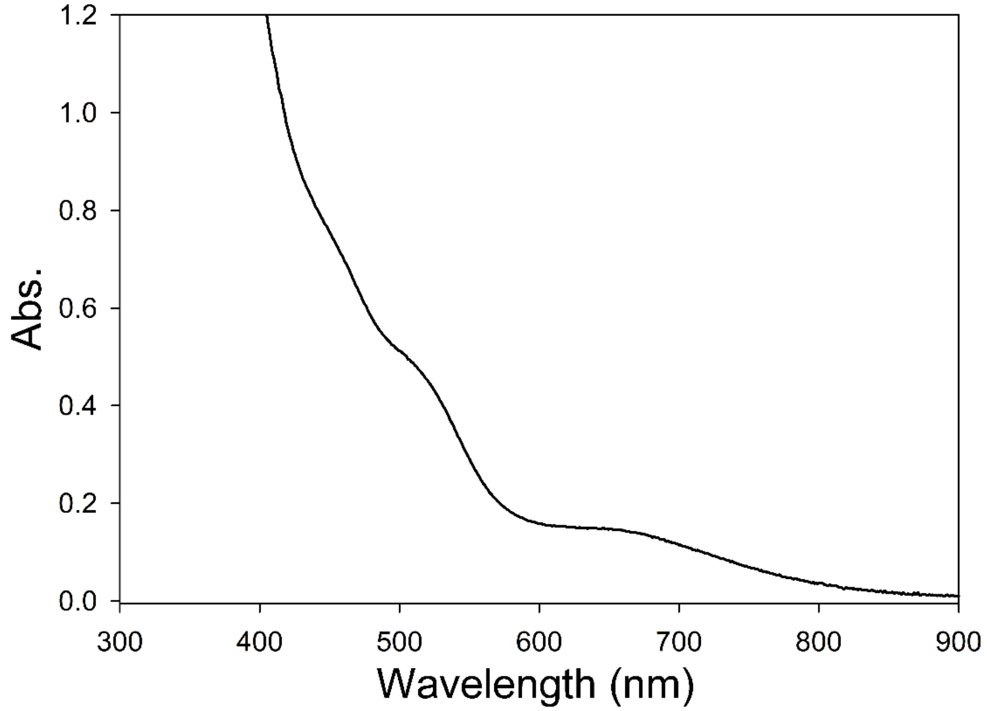

Figure S7. UV-vis spectrum of $(Et_4N)_2[Ni(S\text{-}p\text{-}CF_3\text{-}Ph)_4]$ (**1**) (0.15 mM) in MeCN at 25 °C.

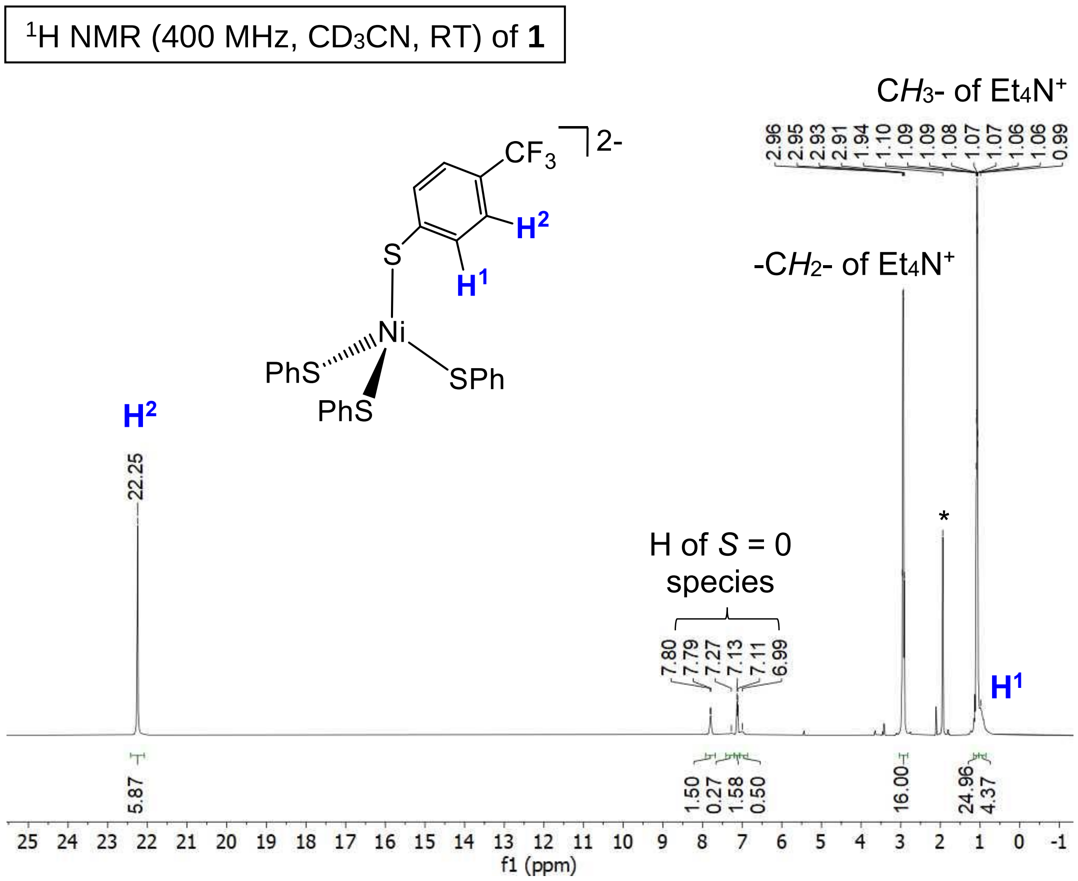

Figure S8. ^{1}H NMR spectrum of $(Et_4N)_2[Ni(S\text{-}p\text{-}CF_3\text{-}Ph)_4]$ (**1**) in CD_3CN at RT (δ vs. protio signal (*) at 1.94 ppm). Structure of **1** depicted in the inset displays one thiolate for clarity; all PhS^- = *p*-CF_3-PhS^-. Zoom-in views of the 1-3 ppm and 7-8 ppm regions are shown below in Figure S9.

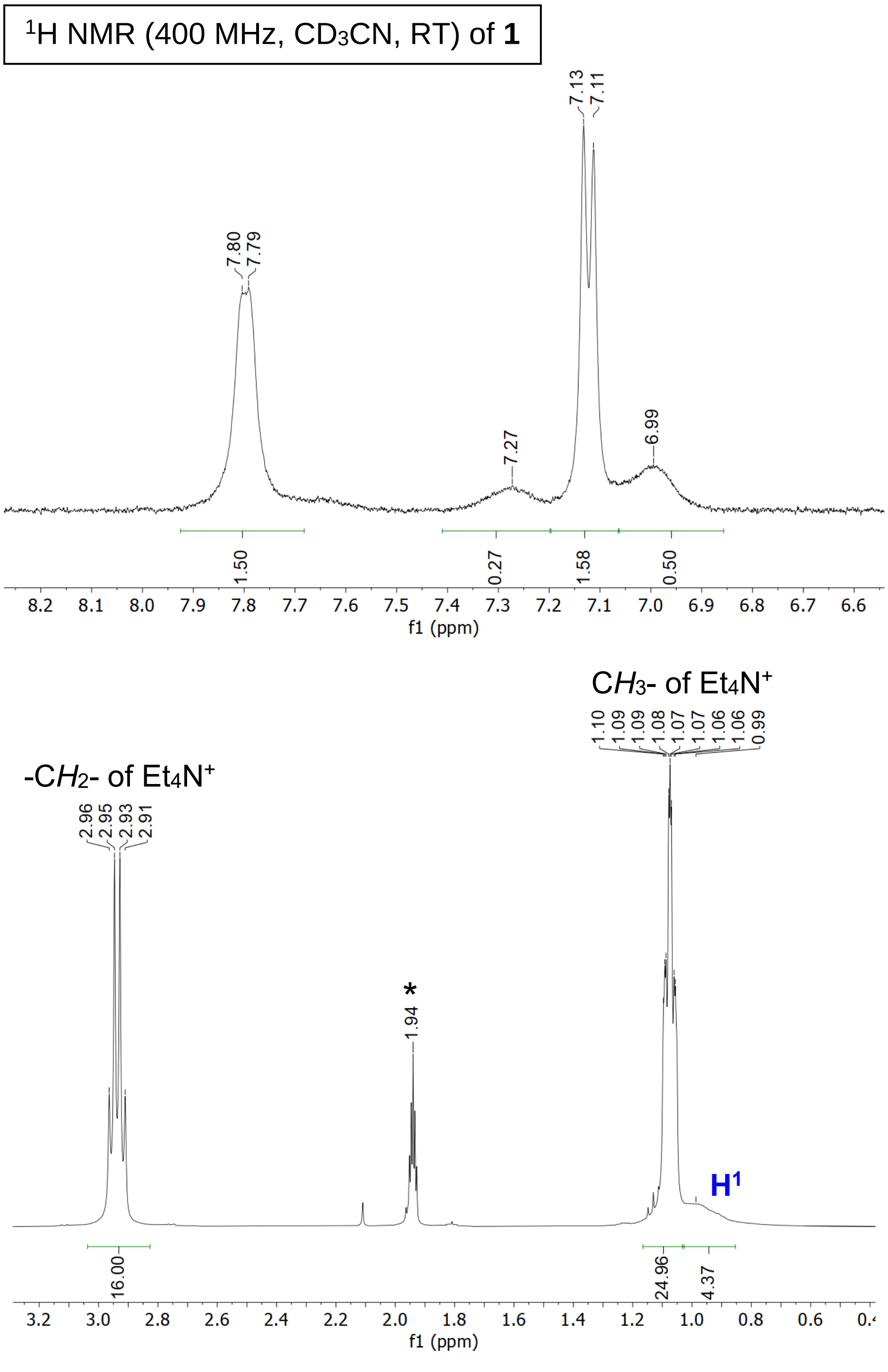

Figure S9. Magnified regions of the ^{1}H NMR spectrum of $(Et_4N)_2[Ni(S\text{-}p\text{-}CF_3\text{-}Ph)_4]$ (**1**) in CD_3CN at RT (δ vs. protio signal (*) at 1.94 ppm). Structure of **1** depicted in the inset displays one thiolate for clarity; all PhS^- = p-CF_3-PhS^-. *Top*: zoom-in of the 7-8 ppm region depicting H of the $S = 0$ species. *Bottom*: zoom-in of the 1-3 ppm region that contains Et_4N^+ and H^1. Full spectrum in Figure S8.

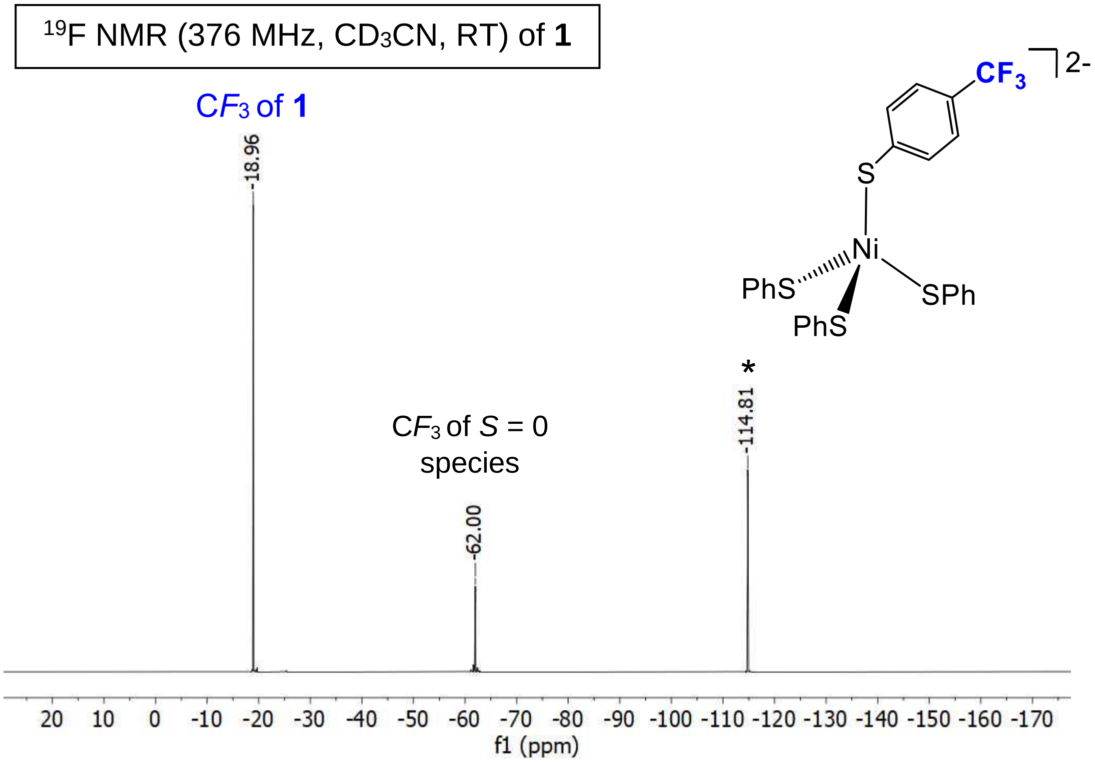

Figure S10. ^{19}F NMR spectrum of $(Et_4N)_2[Ni(S\text{-}p\text{-}CF_3\text{-}Ph)_4]$ (**1**) in CD_3CN at RT. Structure of **1** depicted in the inset displays one thiolate for clarity; all PhS^- = *p*-CF_3-PhS^-. Peak at -114.81 ppm is from PhF (internal standard) and indicated with *.

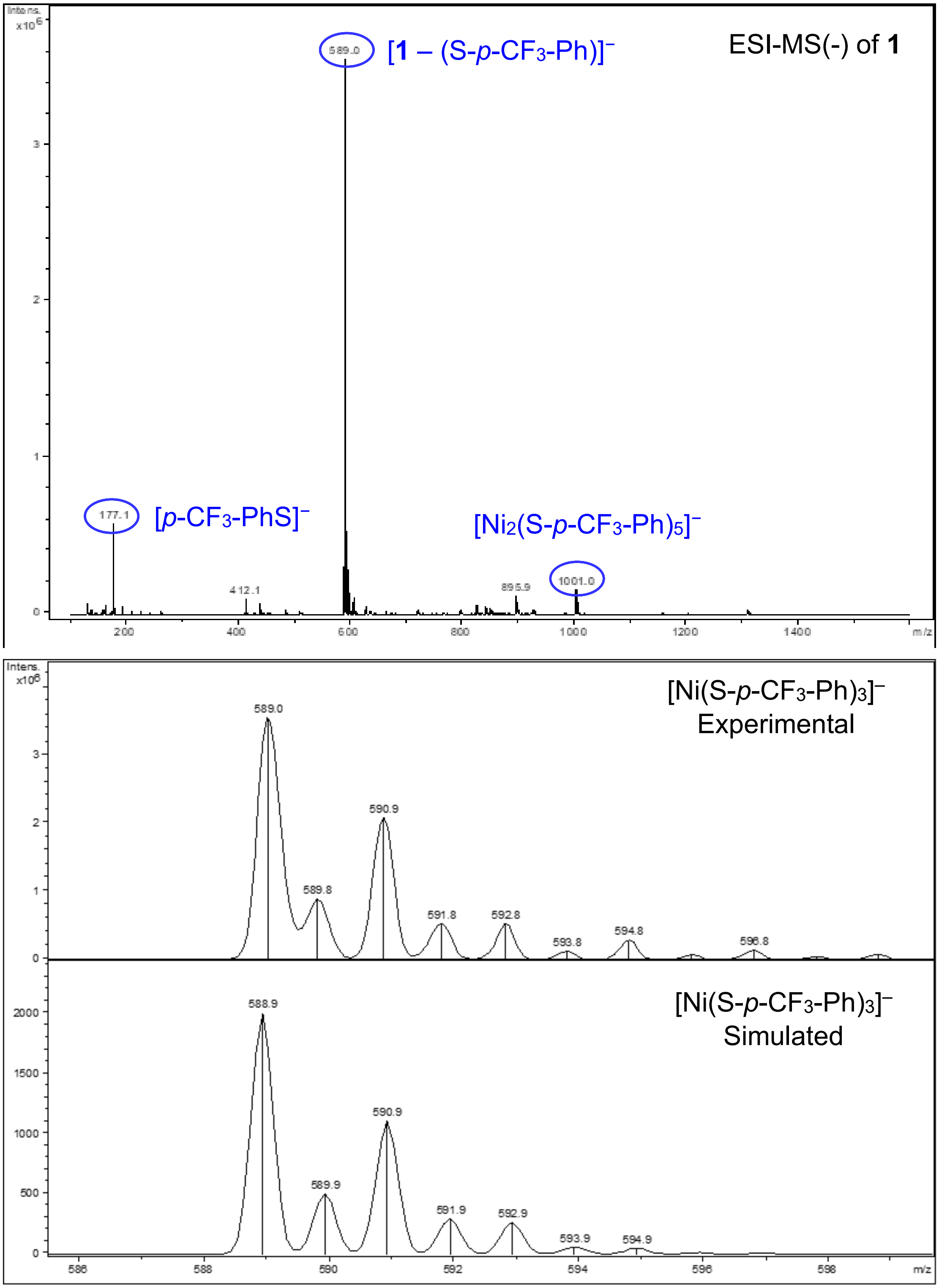

Figure S11. *Top*: Low-resolution ESI-MS(-) of **1** in MeCN. *Bottom*: experimental and simulated theoretical isotopic distribution of peak at *m/z*: 589.0.

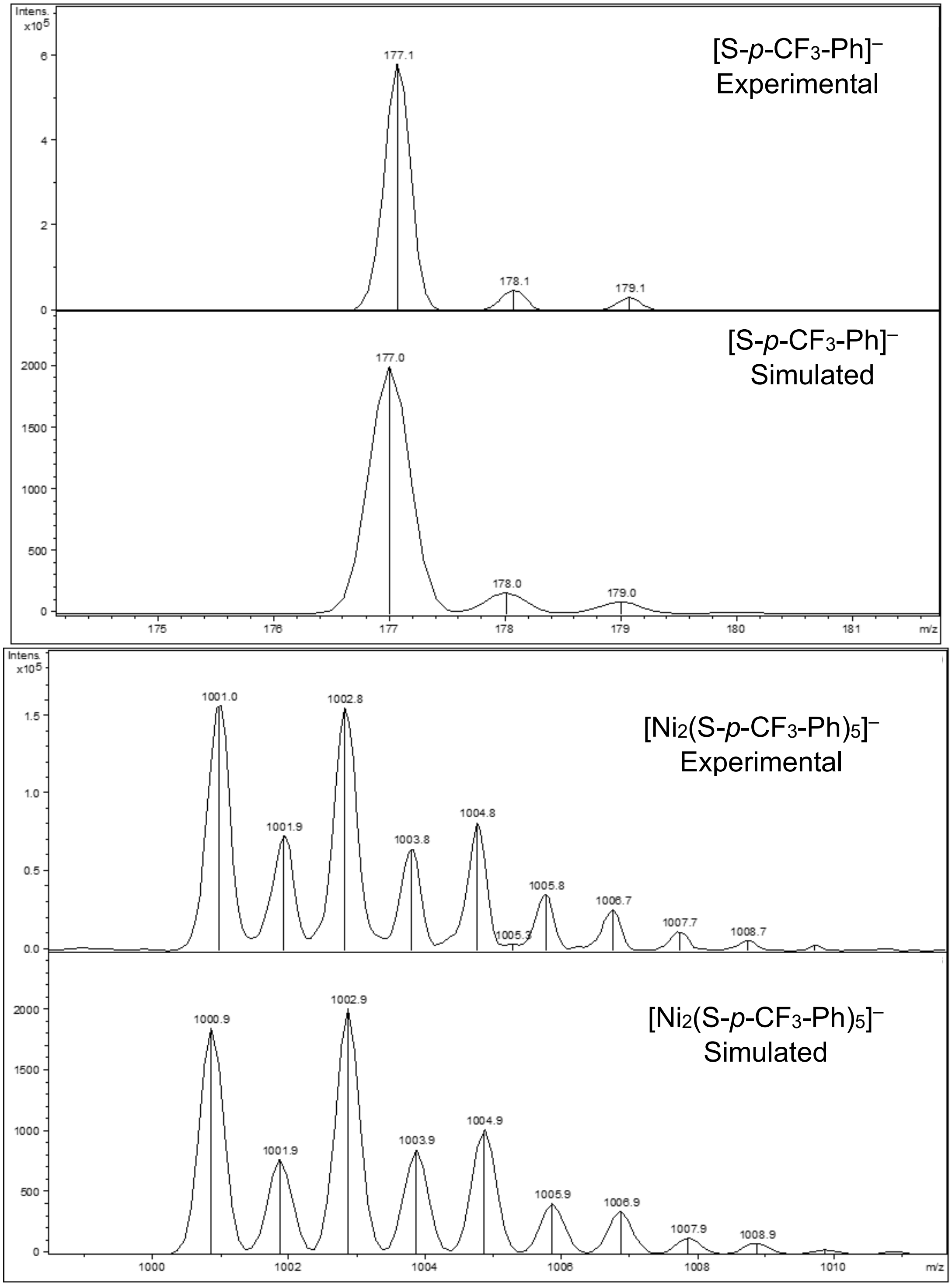

Figure S12. Zoom-in of low-resolution ESI-MS(-) of **1** in MeCN (full MS in Figure S11). *Top*: experimental and simulated theoretical isotopic distribution of peak at *m/z*: 177.1. *Bottom*: experimental and simulated theoretical isotopic distribution of peak at *m/z*: 1001.0.

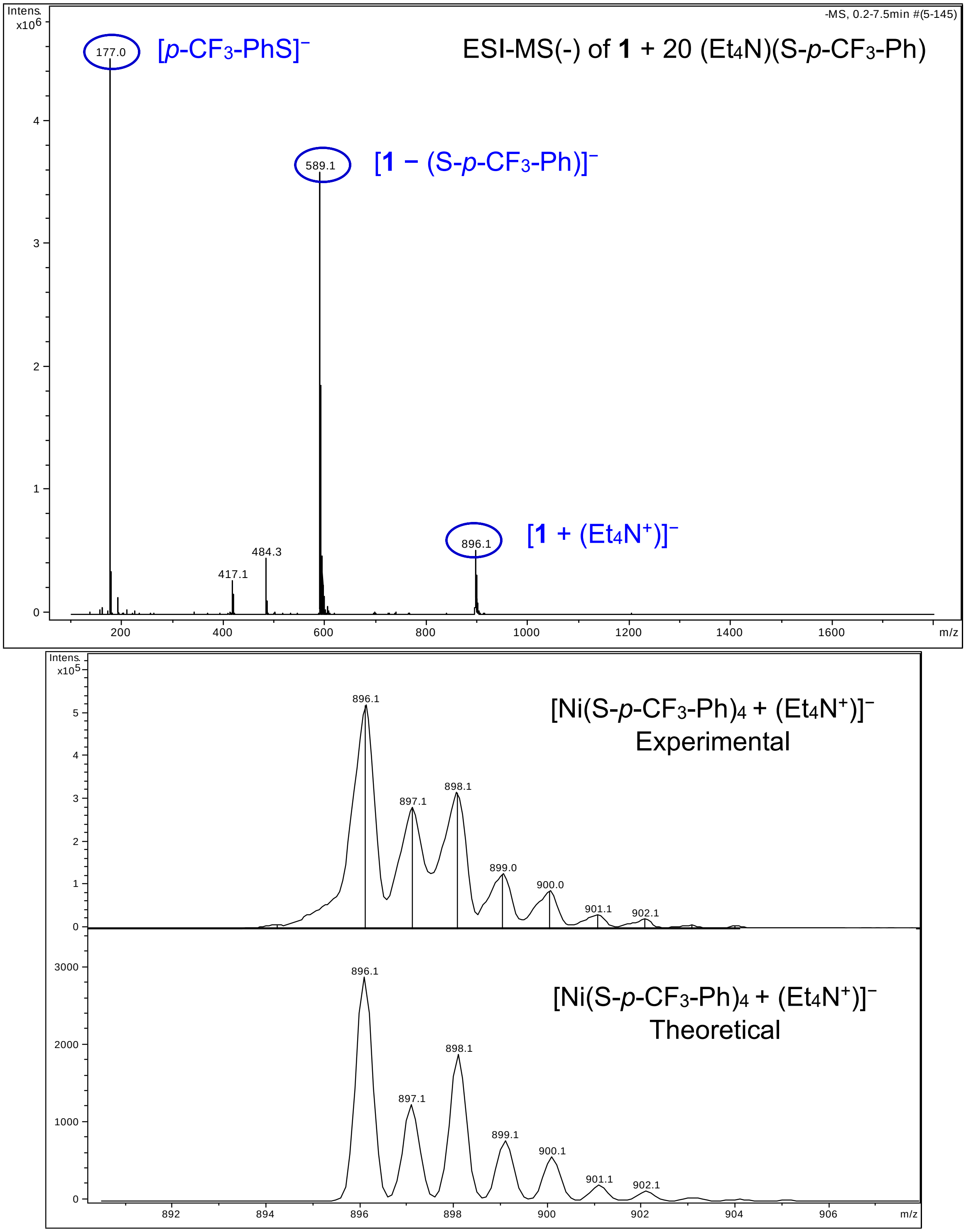

Figure S13. *Top*: Low-resolution ESI-MS(-) of $(Et_4N)_2[Ni(S\text{-}p\text{-}CF_3\text{-}Ph)_4]$ (**1**) with 20 mol-equiv. of $(Et_4N)(S\text{-}p\text{-}CF_3\text{-}Ph)$ in MeCN. *Bottom*: experimental and simulated theoretical isotopic distribution peak at *m/z*: 896.1.

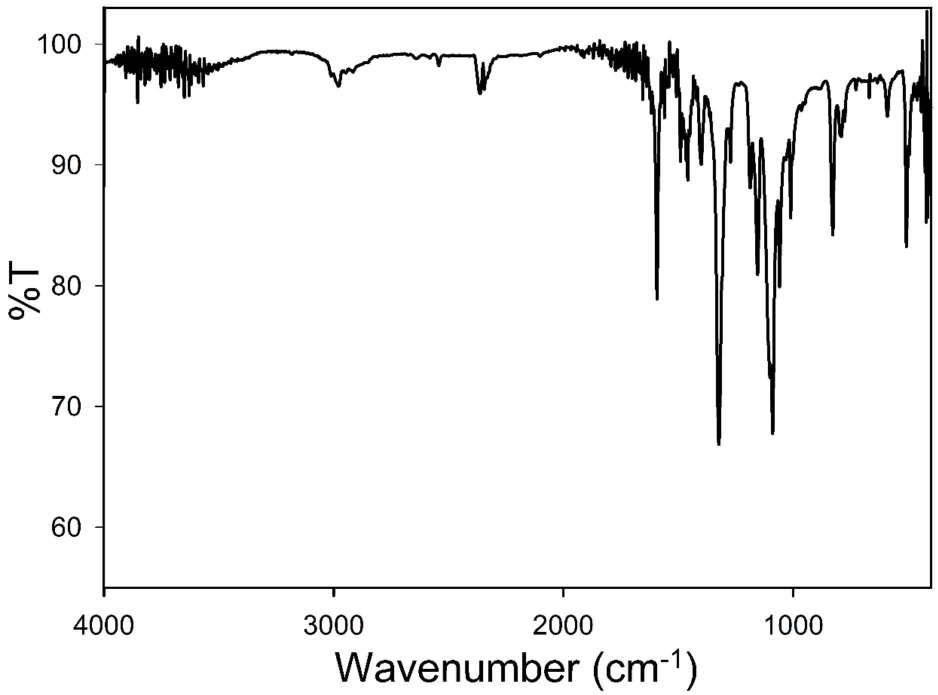

Figure S14. Solid-state FTIR spectrum of $(Et_4N)_2[Zn(S\text{-}p\text{-}CF_3\text{-}Ph)_4]$ (**2**) in a KBr matrix at RT.

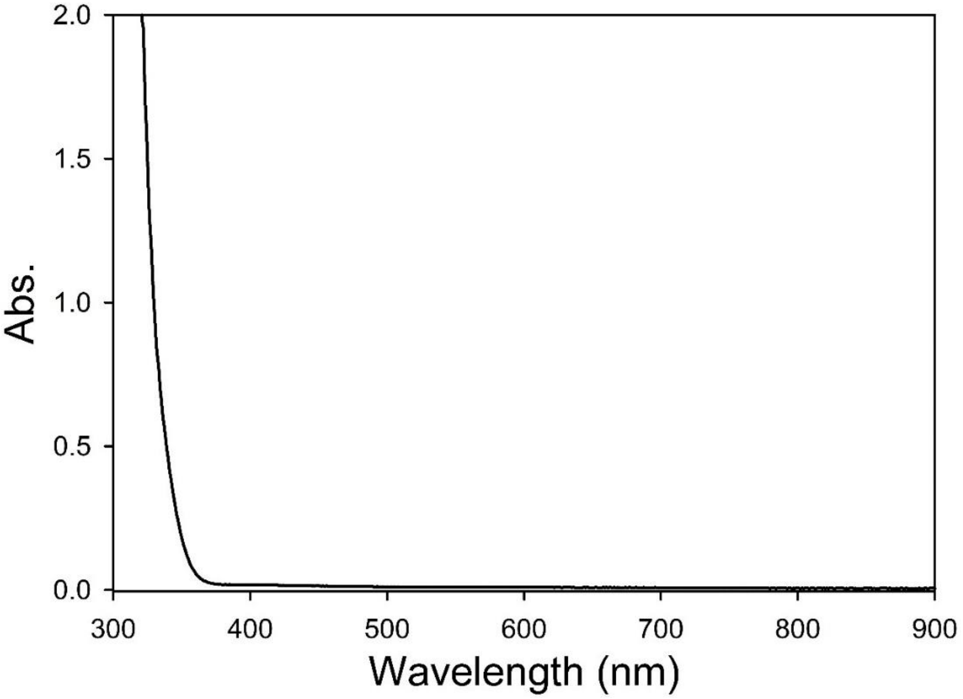

Figure S15. Qualitative UV-vis spectrum of $(Et_4N)_2[Zn(S\text{-}p\text{-}CF_3\text{-}Ph)_4]$ (**2**) in MeCN at 25 °C.

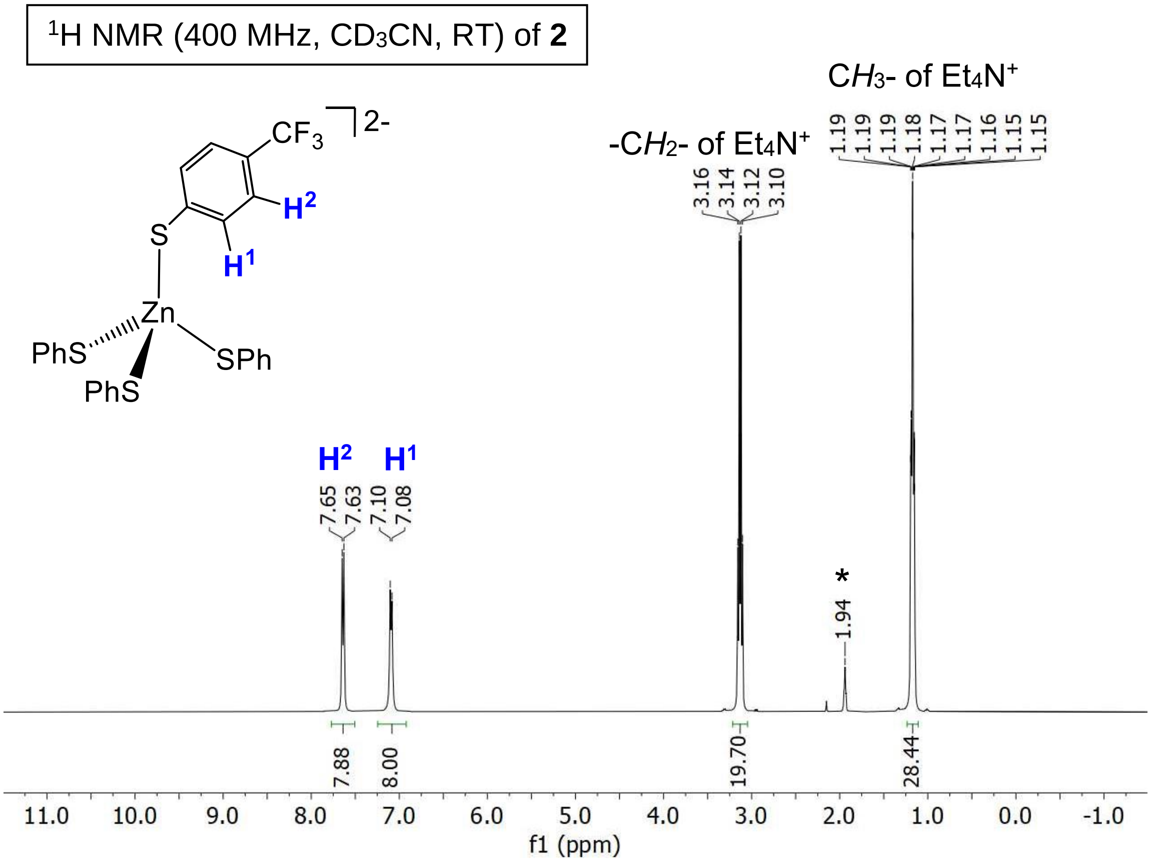

Figure S16. ^{1}H NMR spectrum of $(Et_4N)_2[Zn(S\text{-}p\text{-}CF_3\text{-}Ph)_4]$ (**2**) in CD_3CN at RT (δ vs. protio signal (*) at 1.94 ppm). Structure of **2** depicted in the inset displays one thiolate for clarity; all $PhS^{-} = p\text{-}CF_3\text{-}PhS^{-}$.

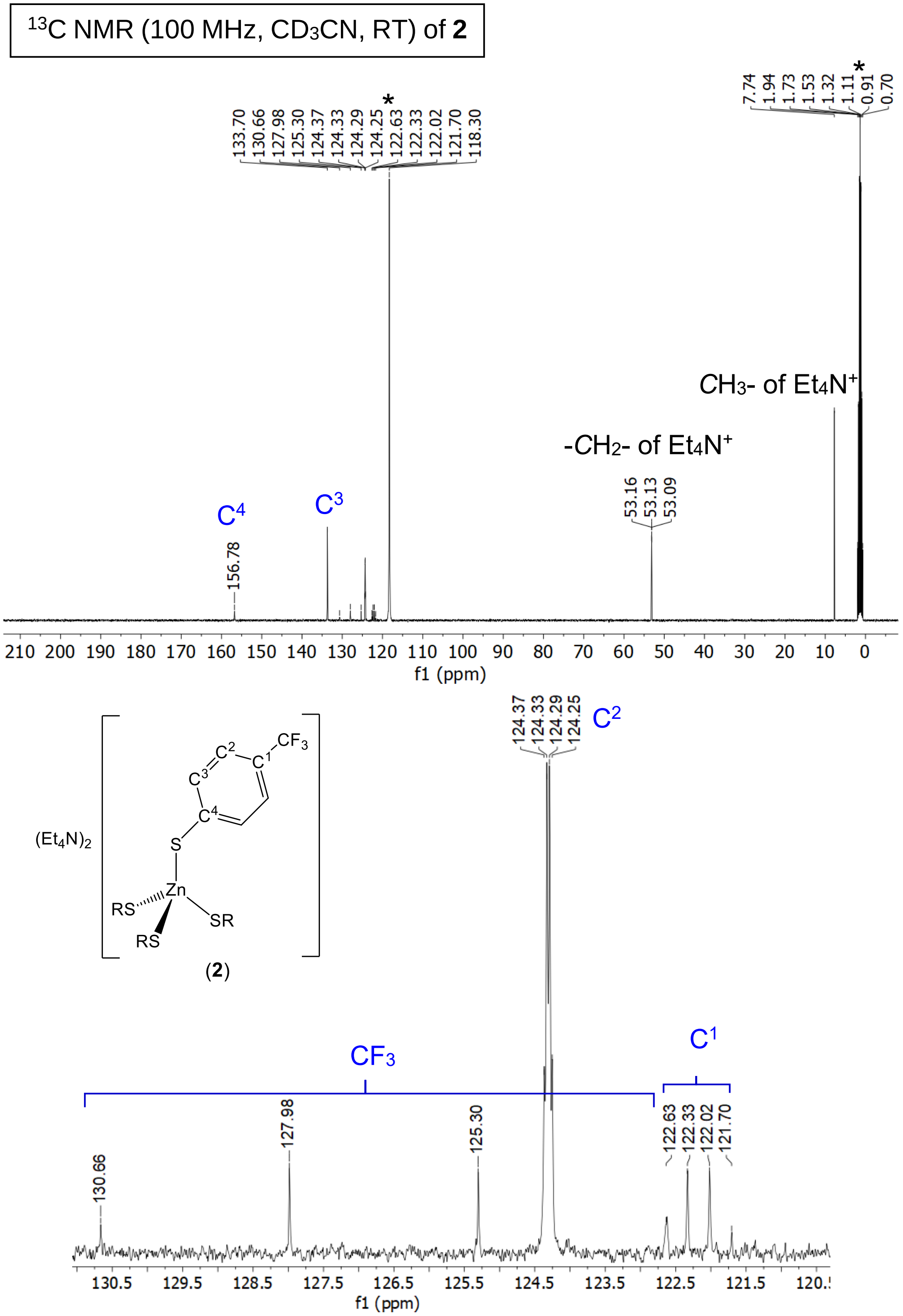

Figure S17. *Top*: ^{13}C NMR spectrum of $(Et_4N)_2[Zn(S\text{-}p\text{-}CF_3\text{-}Ph)_4]$ (**2**) in CD_3CN at RT. *Bottom:* expansion of 120-131 ppm region. Solvent signals at 1.32 and 118.31 ppm are indicated with *.

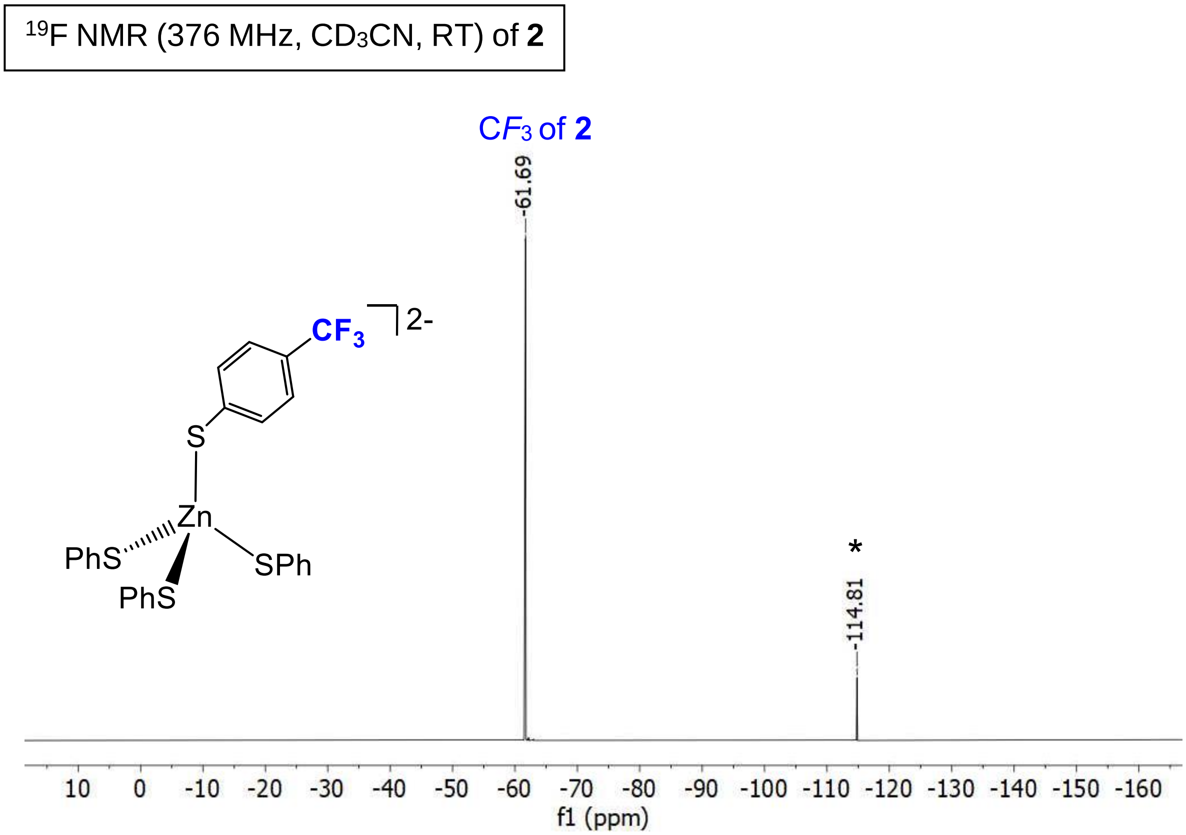

Figure S18. ^{19}F NMR spectrum of $(Et_4N)_2[Zn(S\text{-}p\text{-}CF_3\text{-}Ph)_4]$ (**2**) in CD_3CN at RT. Structure of **2** depicted in the inset displays one thiolate for clarity; all PhS^{-} = $p\text{-}CF_3\text{-}PhS^{-}$. Peak at -114.81 ppm is from PhF (internal standard) as indicated with *.

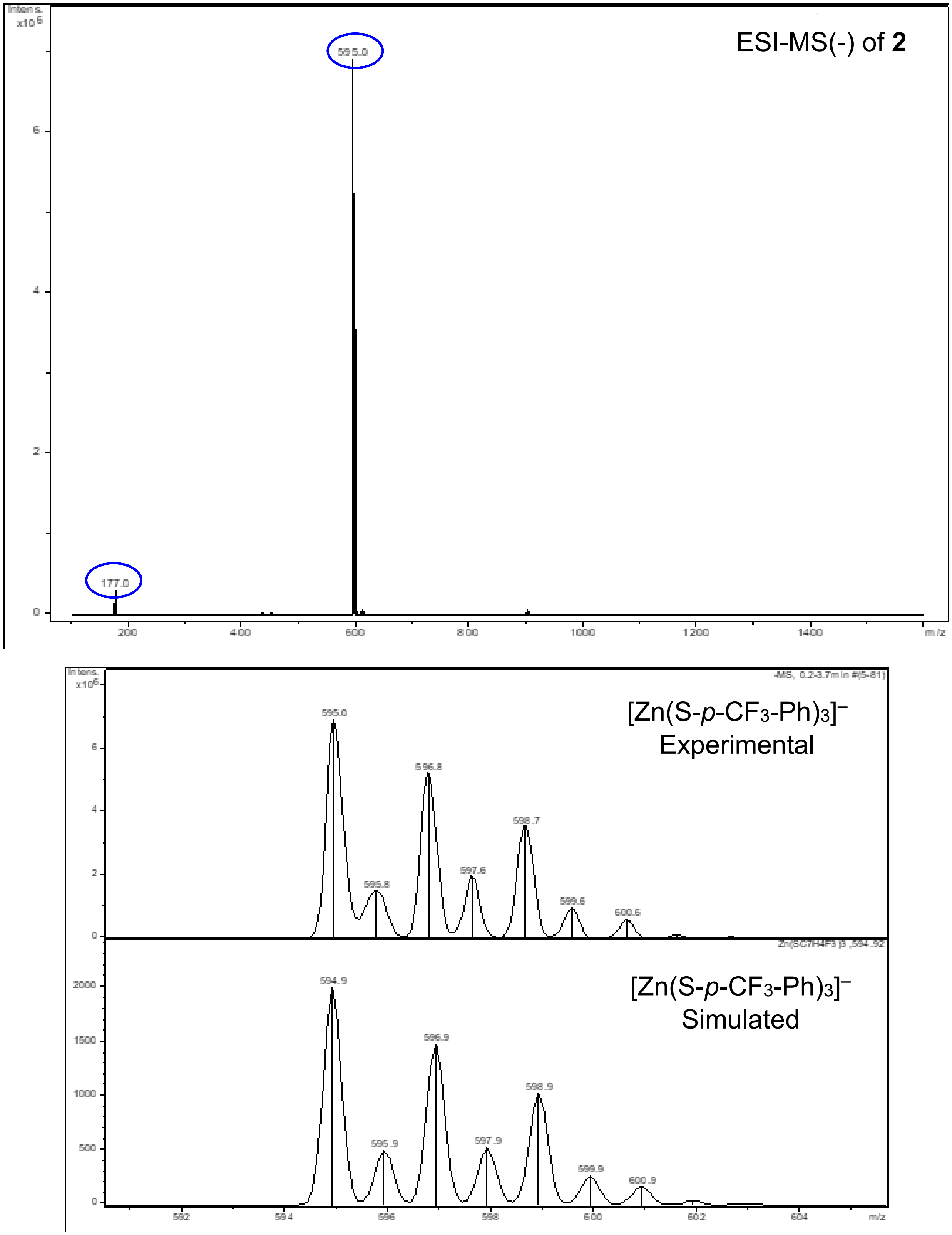

Figure S19. *Top*: Low-resolution ESI-MS(-) of $(Et_4N)_2[Zn(S\text{-}p\text{-}CF_3\text{-}Ph)_4]$ (**2**) in MeCN. *Bottom*: experimental and simulated theoretical isotopic distribution of peak at *m/z*: 595.0.

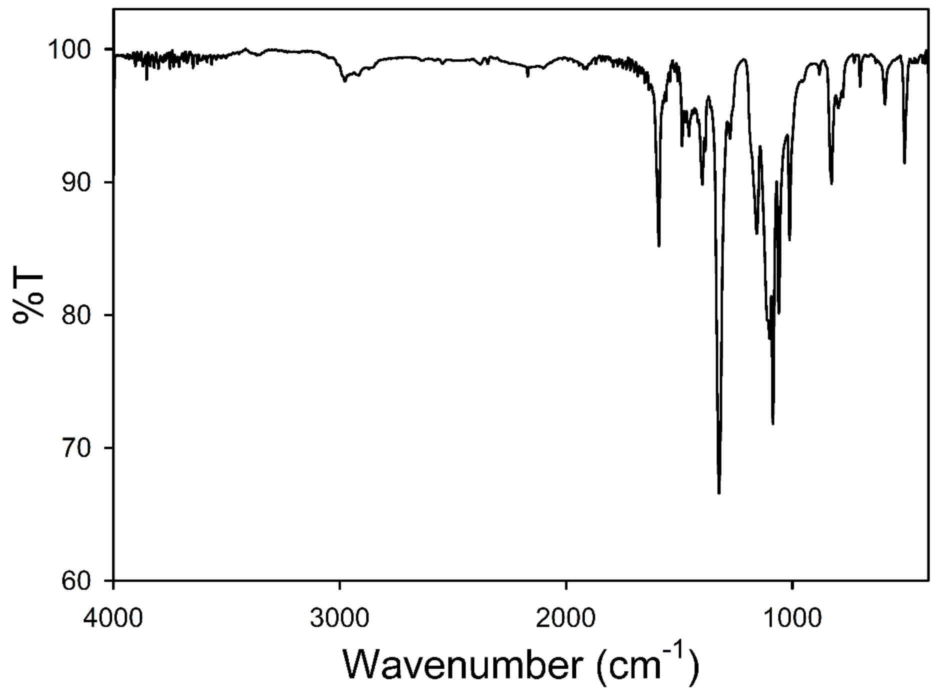

Figure S20. Solid-state FTIR spectrum of isolated product from the attempted synthesis of $(Et_4N)_2[Ni_2(S\text{-}p\text{-}CF_3\text{-}Ph)_6]$ (**3**) in a KBr matrix at RT.

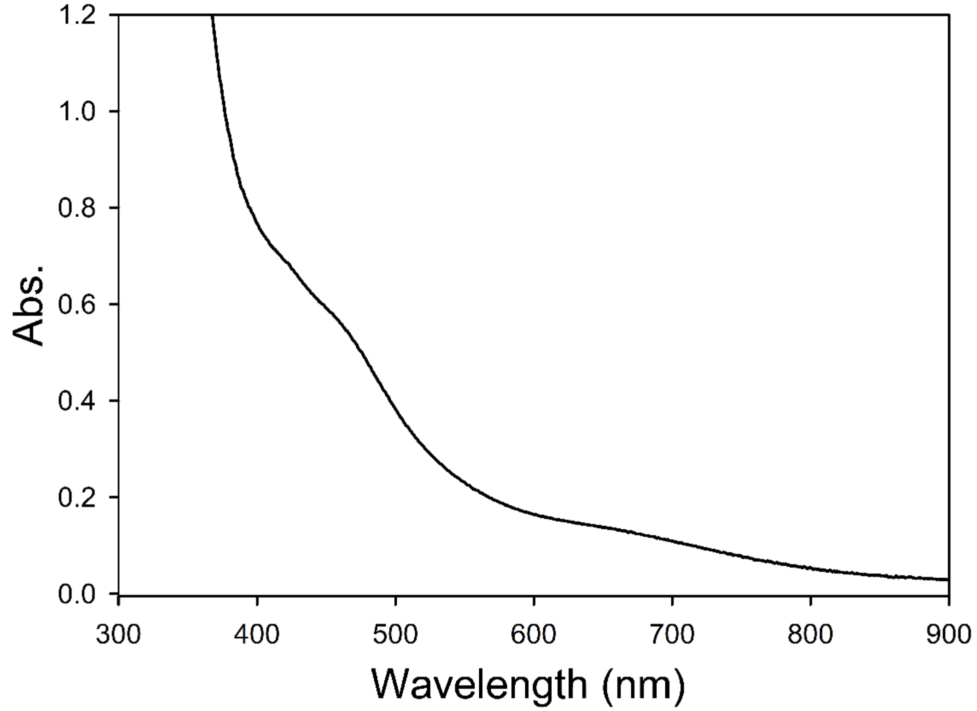

Figure S21. Qualitative UV-vis spectrum of isolated product from the attempted synthesis of $(Et_4N)_2[Ni_2(S\text{-}p\text{-}CF_3\text{-}Ph)_6]$ (**3**) in MeCN at 25 ˚C.

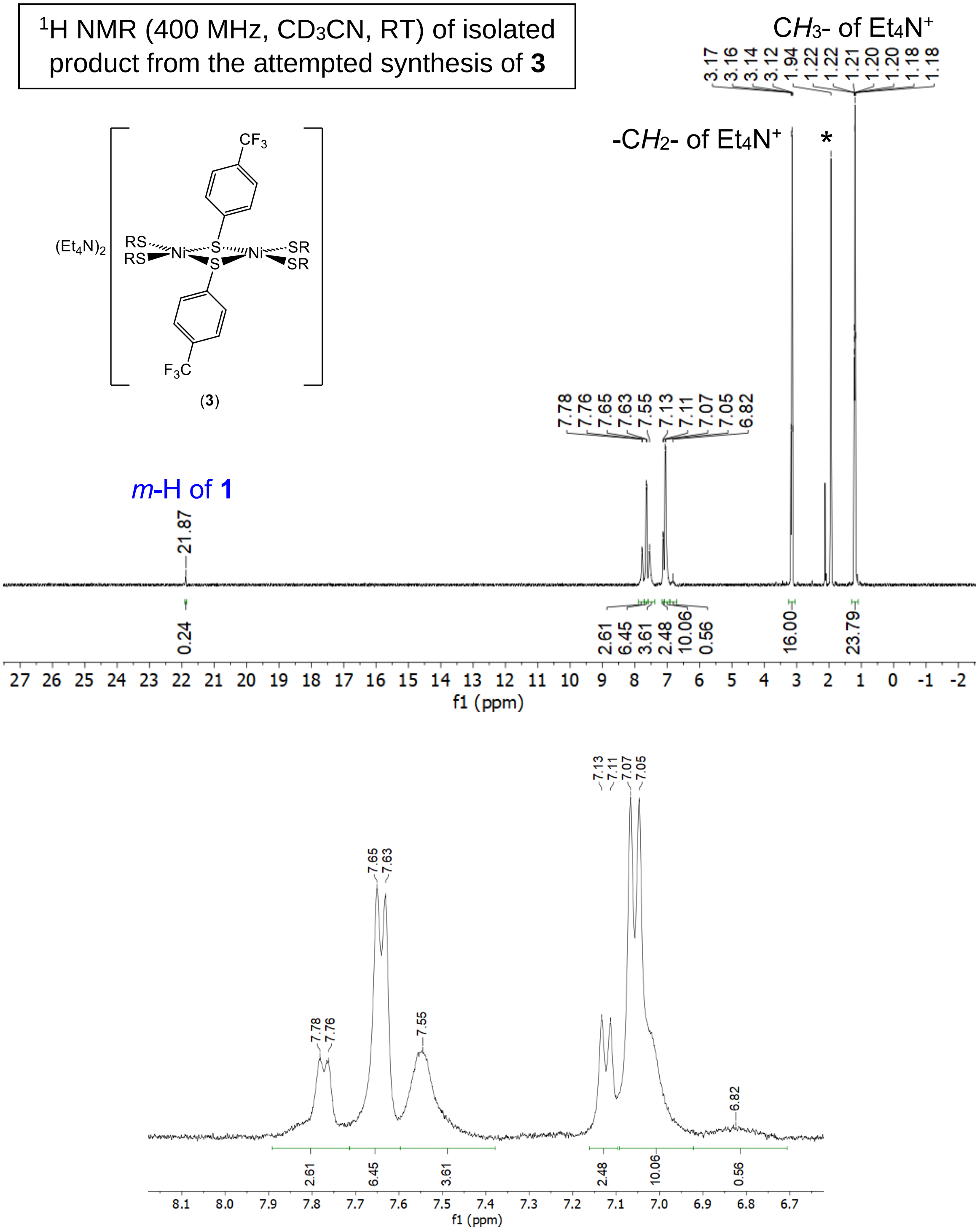

Figure S22. *Top*: ^{1}H NMR spectrum of isolated product from the attempted synthesis of $(Et_4N)_2[Ni_2(S\text{-}p\text{-}CF_3\text{-}Ph)_6]$ (**3**) in CD_3CN at RT (δ vs. protio signal (*) at 1.94 ppm). Proposed structure of **3** depicted in the inset displays only the bridging thiolate for clarity; all R = *p*-CF_3-PhS^-. *Bottom:* Zoom-in view of the 6-8 ppm region.

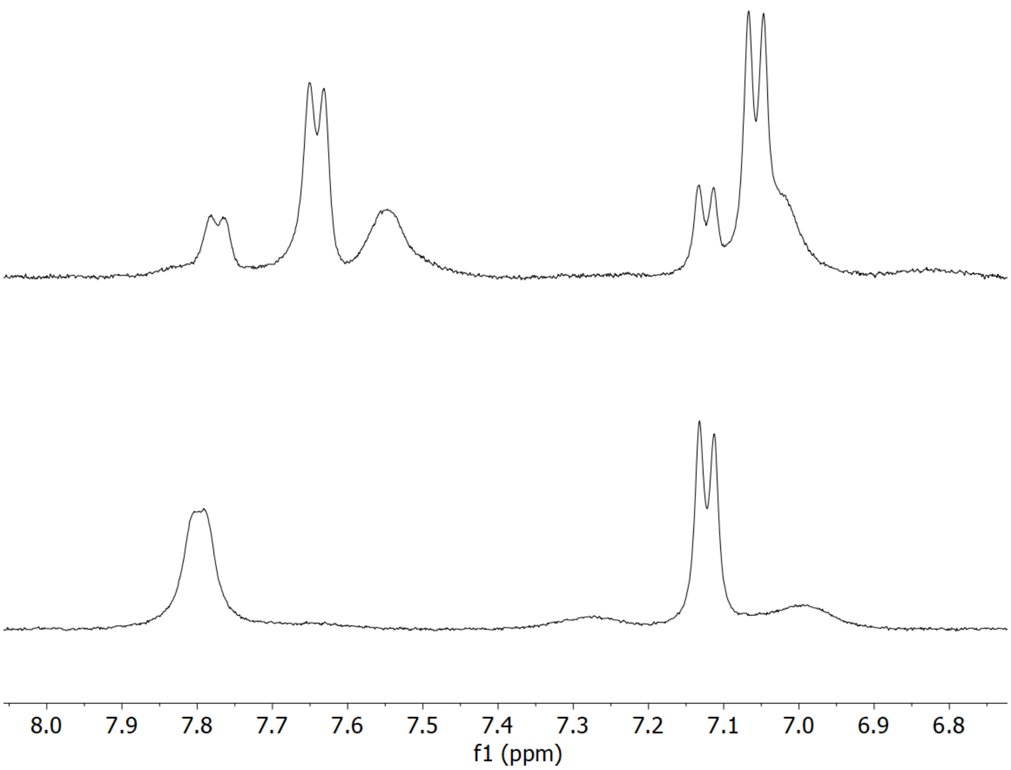

Figure S23. Comparison of the 6-8 ppm region in the ^{1}H NMR spectrum of the product from the attempted synthesis of **3** (top; see Figure S22 for full spectrum) and as-isolated **1** (bottom; see Figure S8 for full spectrum).

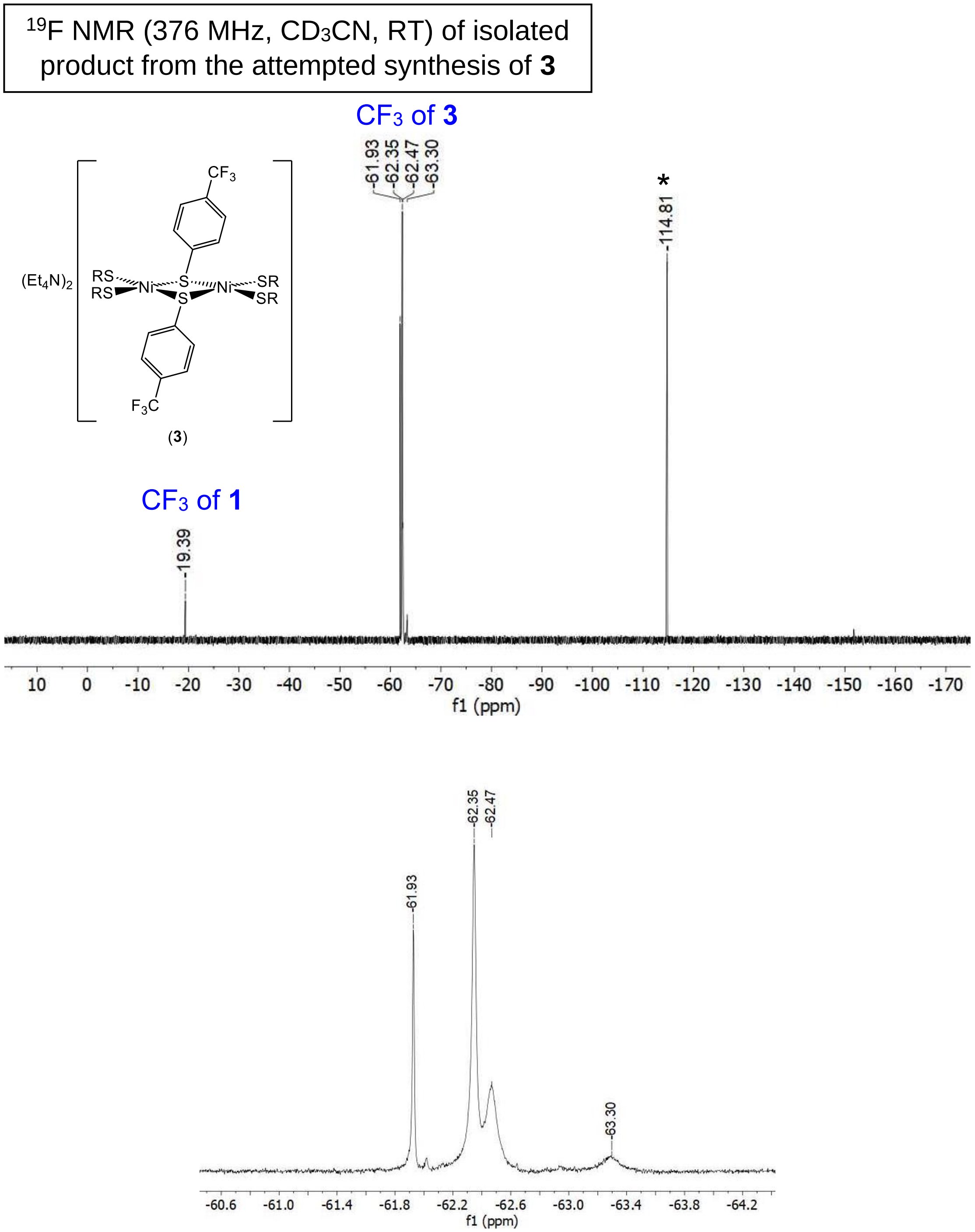

Figure S24. *Top*: ^{19}F NMR spectrum of isolated product from the attempted synthesis of $(Et_4N)_2[Ni_2(S\text{-}p\text{-}CF_3\text{-}Ph)_6]$ (**3**) in CD_3CN at RT. Proposed structure of **3** depicted in the inset displays only the bridging thiolate for clarity; all R = *p*-CF_3-PhS$^-$. *Bottom:* Zoom-in view of the -64 to -61 ppm region. Peak at -114.81 ppm is from PhF (internal standard) as indicated with *.

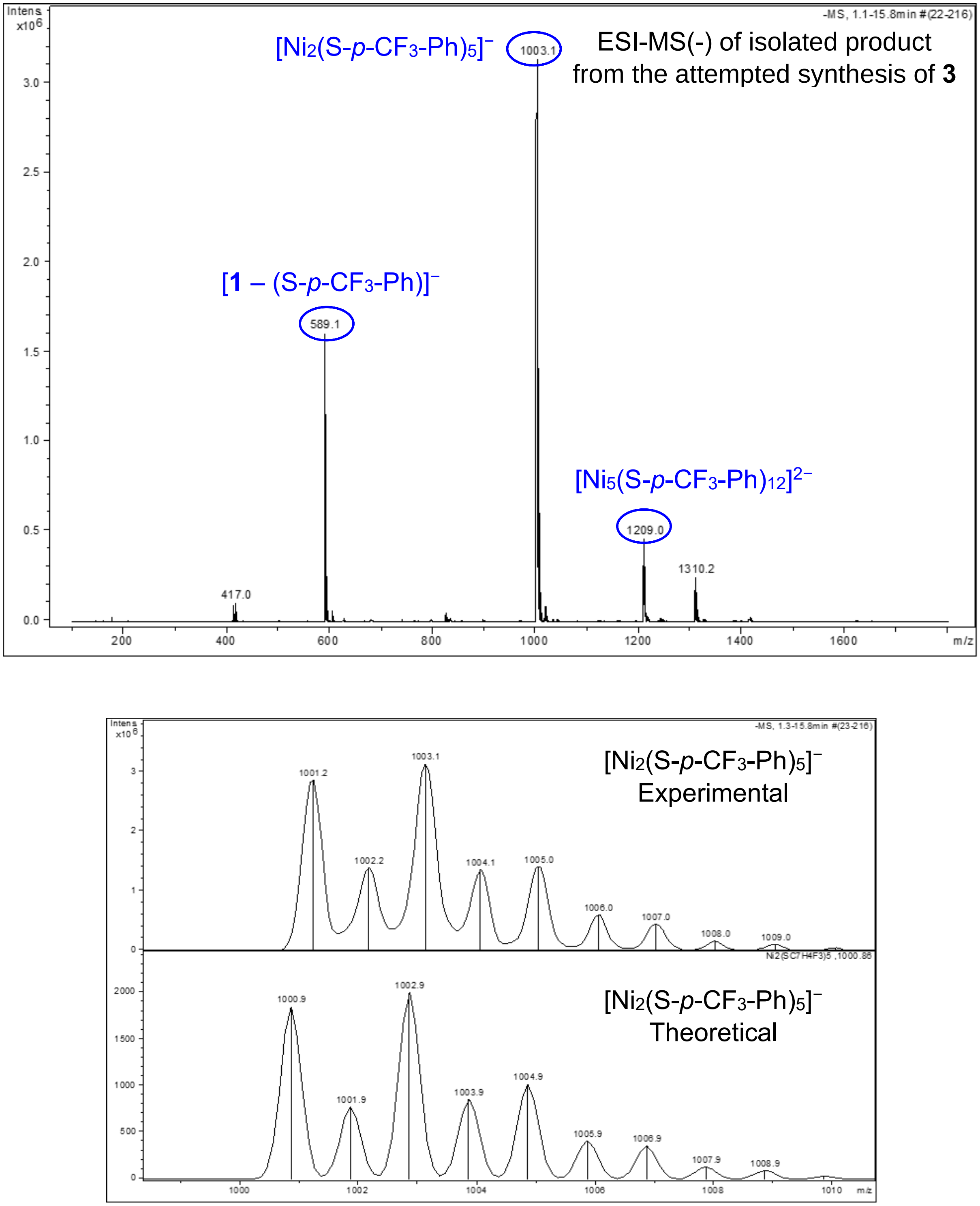

Figure S25. *Top*: Low-resolution ESI-MS(-) of product isolated from the attempted synthesis of **3** in MeCN. *Bottom*: experimental and simulated theoretical isotopic distribution of peak at *m/z*: 1001.2 peak.

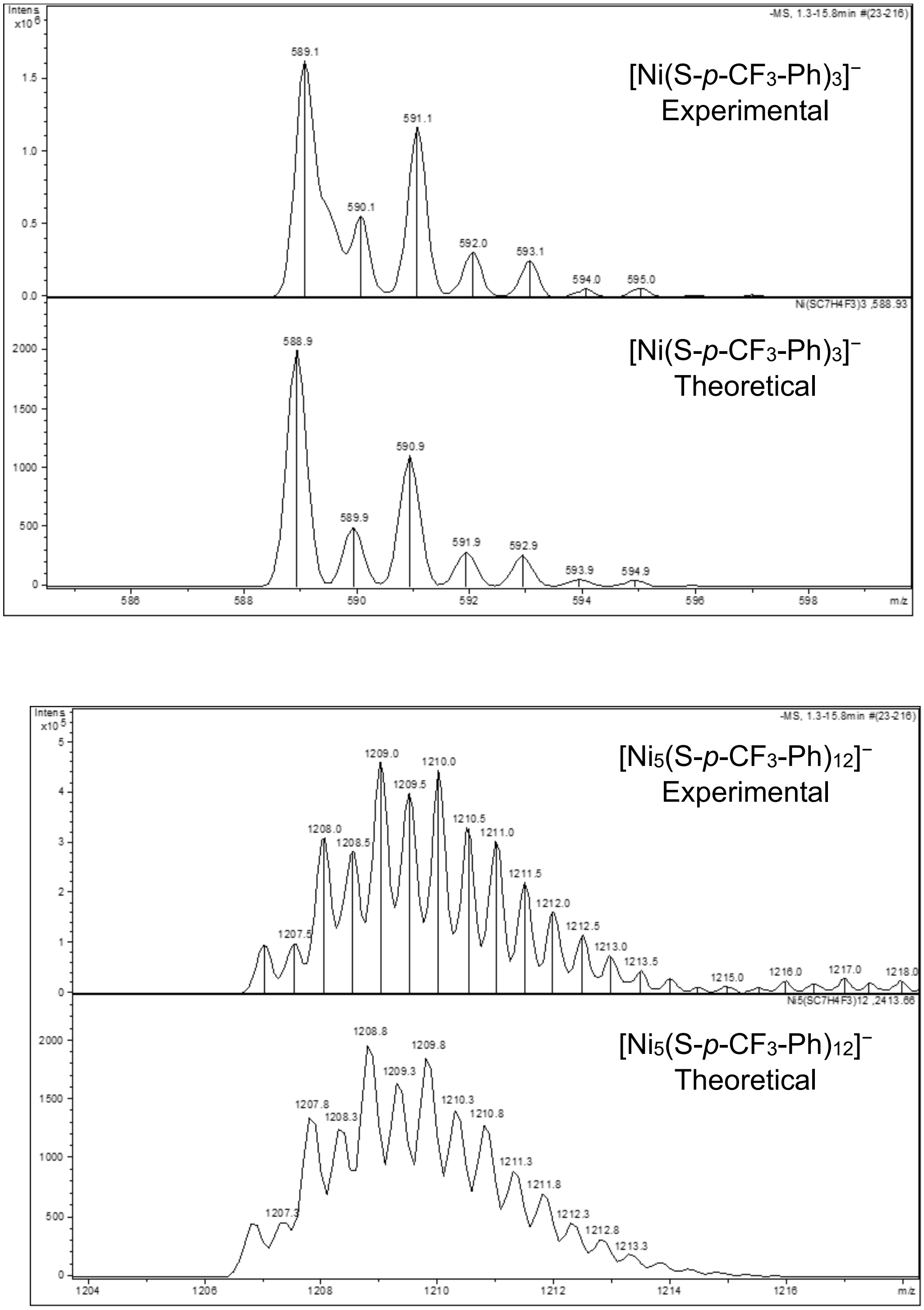

Figure S26. Low-resolution ESI-MS(-) of product isolated from the attempted synthesis of **3** in MeCN. *Top*: experimental and simulated theoretical isotopic distribution of peak at *m/z*: 589.1 peak. *Bottom*: experimental and simulated theoretical isotopic distribution of peak at *m/z*: 1209.0 peak.

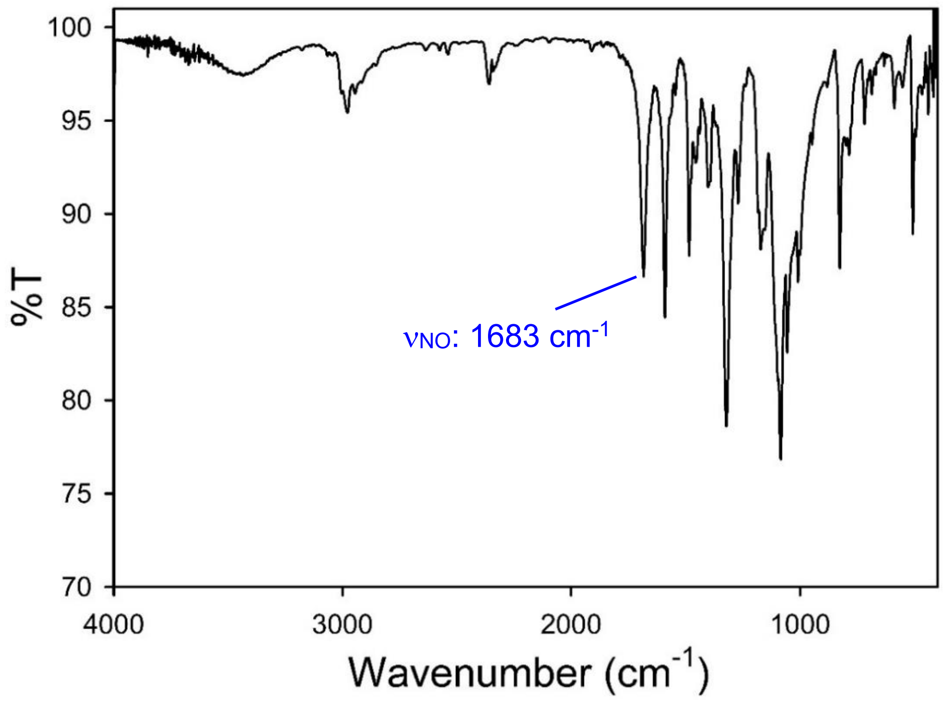

Figure S27. Solid-state FTIR spectrum of $(Et_4N)_2[Ni(S\text{-}p\text{-}CF_3\text{-}Ph)_3(NO)]$ (**4**) in a KBr matrix at RT.

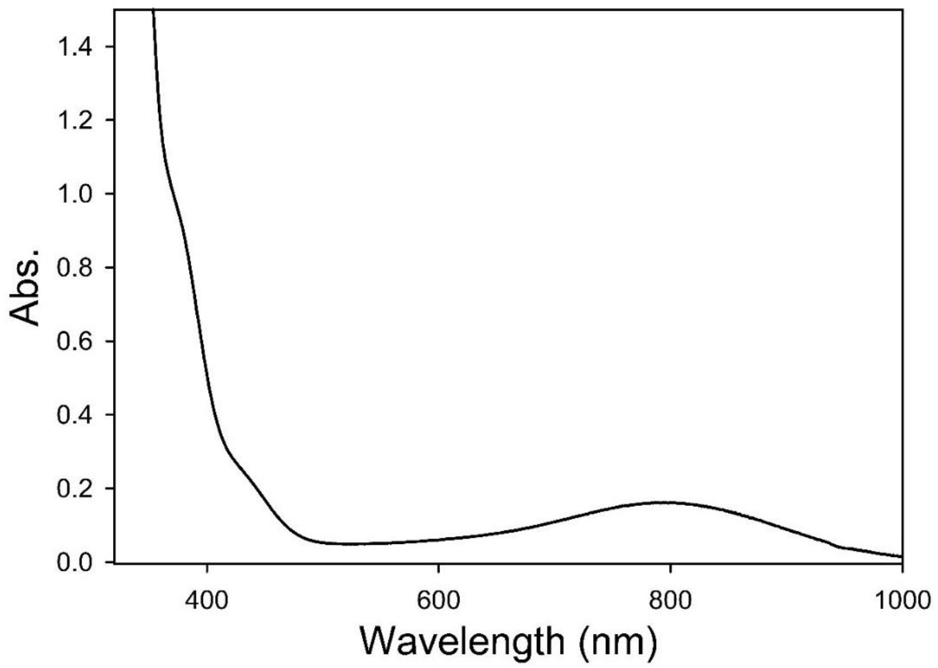

Figure S28. Qualitative UV-vis spectrum of $(Et_4N)_2[Ni(S\text{-}p\text{-}CF_3\text{-}Ph)_3(NO)]$ (**4**) in MeCN at 25 °C.

^{1}H NMR (400 MHz, CD_3CN, RT) of **4**

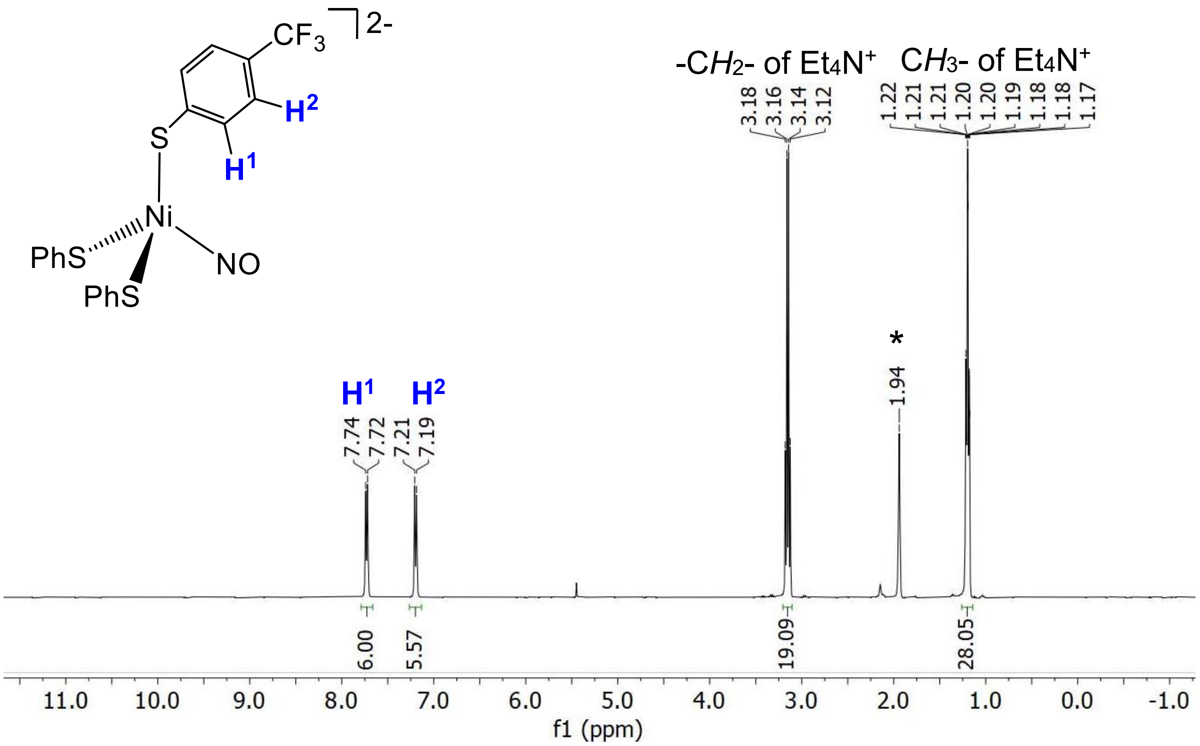

Figure S29. ^{1}H NMR spectrum of $(Et_4N)_2[Ni(S\text{-}p\text{-}CF_3\text{-}Ph)_3(NO)]$ (**4**) in CD_3CN at RT (δ vs. protio signal (*) at 1.94 ppm). Structure of **4** depicted in the inset displays one thiolate for clarity; all PhS^- = p-CF_3-PhS^-.

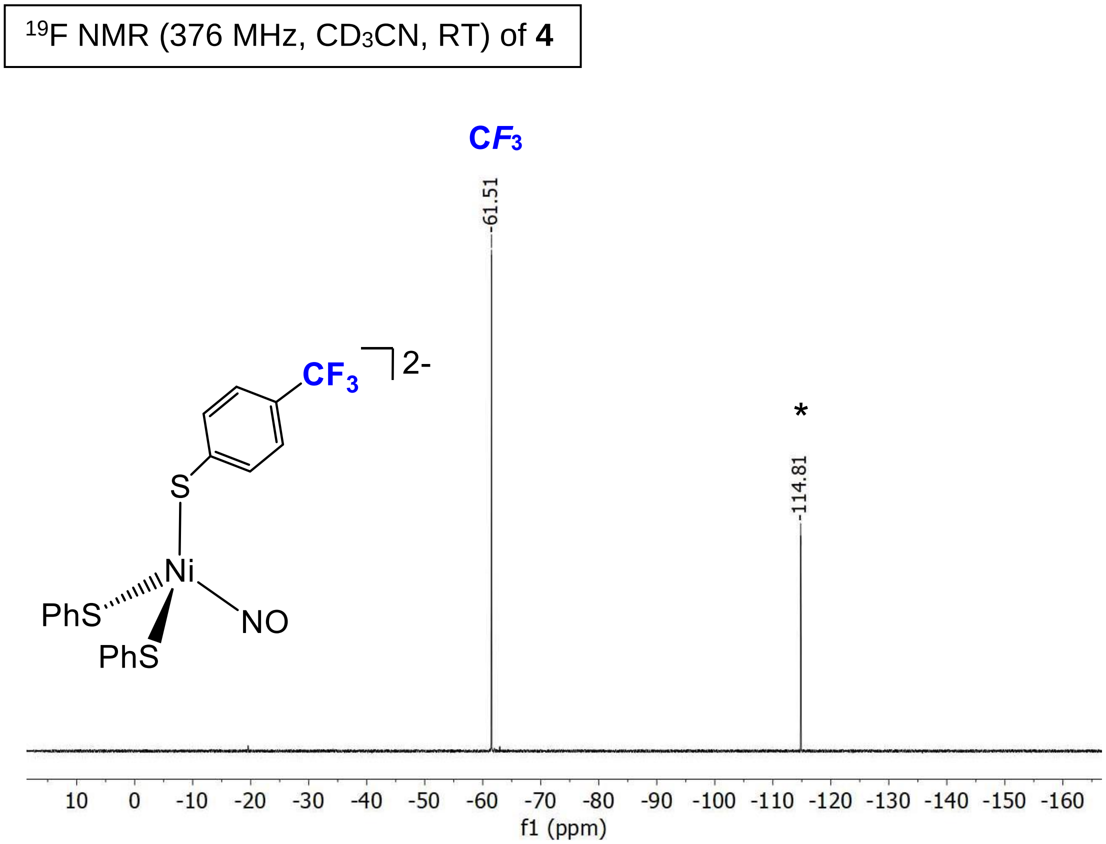

Figure S30. ^{19}F NMR spectrum of $(Et_4N)_2[Ni(S\text{-}p\text{-}CF_3\text{-}Ph)_3(NO)]$ (**4**) in CD_3CN at RT. Structure of **4** depicted in the inset displays one thiolate for clarity; all $PhS^- = p\text{-}CF_3\text{-}PhS^-$. Peak at -114.81 ppm is PhF (internal standard) as indicated with *.

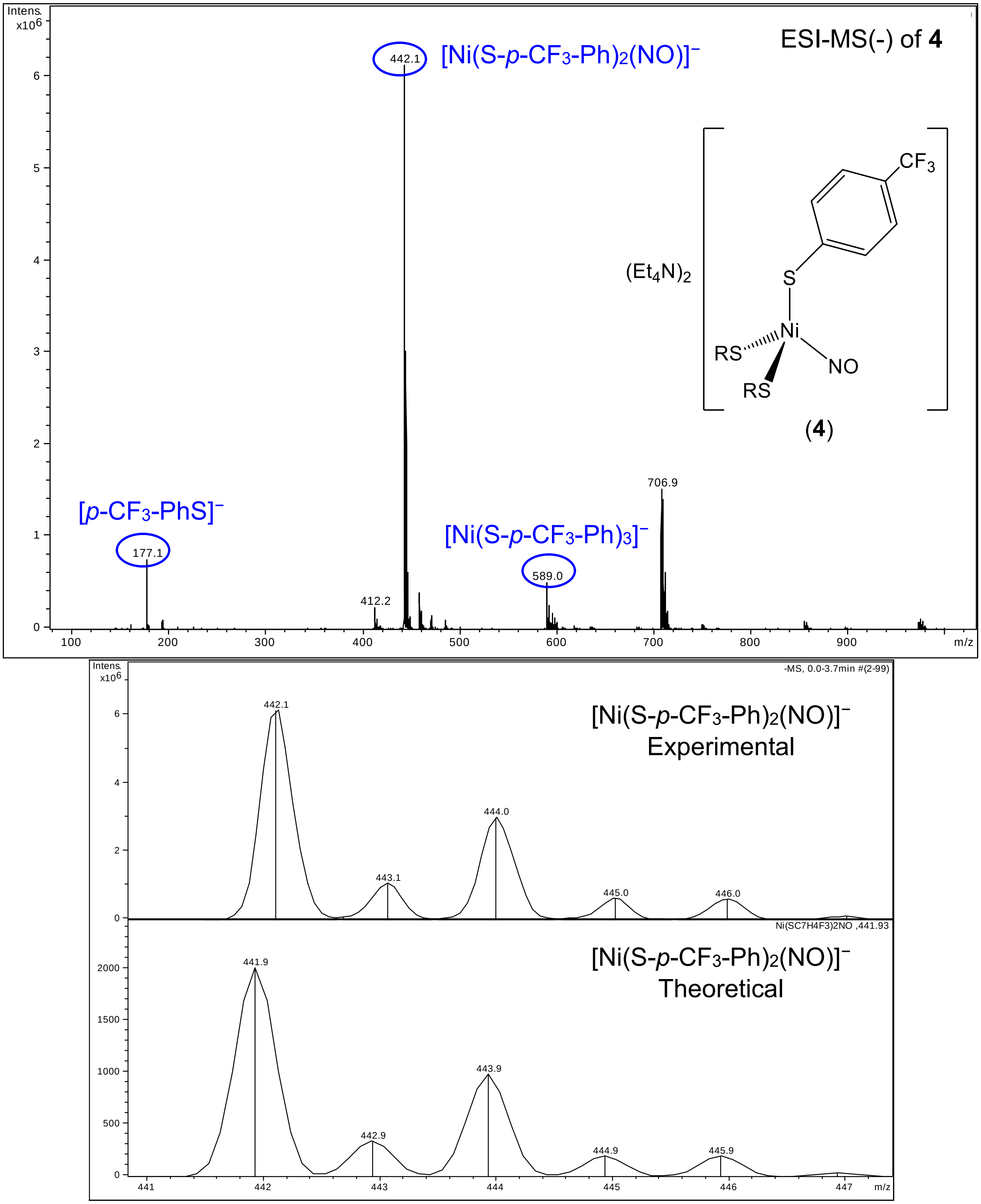

Figure S31. *Top*: Low-resolution ESI-MS(-) of $(Et_4N)_2[Ni(S\text{-}p\text{-}CF_3\text{-}Ph)_3(NO)]$ (**4**) in MeCN. *Bottom*: experimental and simulated theoretical isotopic distribution peak at *m/z*: 442.1.

2. X-ray Crystallography and X-ray Absorption:

Table S1. Summary of Crystal Data and Intensity Collection and Structure Refinement Parameters for $(Et_4N)_2[Ni(S\text{-}p\text{-}CF_3\text{-}Ph)_4]$ (**1**) and $(Et_4N)_2[Zn(S\text{-}p\text{-}CF_3\text{-}Ph)_4]$ (**2**).

Parameters	**1**	**2**
Formula	$C_{44}H_{56}F_{12}N_2NiS_4$	$C_{44}H_{56}F_{12}N_2ZnS_4$
MW	1027.85	1034.51
Crystal system	Monoclinic	Tetragonal
Space group	$P2_1/c$	$I4_1/a$
Crystal color, habit	dark, needle	colorless, prism
a, Å	16.3645(17)	13.4420(13)
b, Å	16.1498(17)	13.4420(13)
c, Å	19.206(2)	25.774(3)
α, deg	90	90
β, deg	110.128(3)	90
γ, deg	90	90
V, $Å^3$	4765.7(9)	4657.1(10)
Z	4	4
$\rho_{calcd,}$ g/cm^3	1.433	1.475
T, K	100(2)	90(2)
abs coeff, μ (Mo Kα), mm^{-1}	0.663	0.789
θ limits, deg	2.4-36.5	2.6-27.5
total no. of data	244656	30445
no. of unique data	23240	2682
no. of parameters	626	268
GOF of F^2	1.011	1.025
R_1,[a]	0.0482	0.0591
wR_2,[b]	0.1278	0.1801
max, min peaks, e/$Å^3$	1.45, -1.30	0.46, -0.34

[a]$R_1 = \Sigma| |F_o| - |F_c| | / \Sigma |F_o|$; [b]$wR_2 = \{\Sigma[w(F_o^2 - F_c^2)^2]/\Sigma[w(F_o^2)^2]\}^{1/2}$

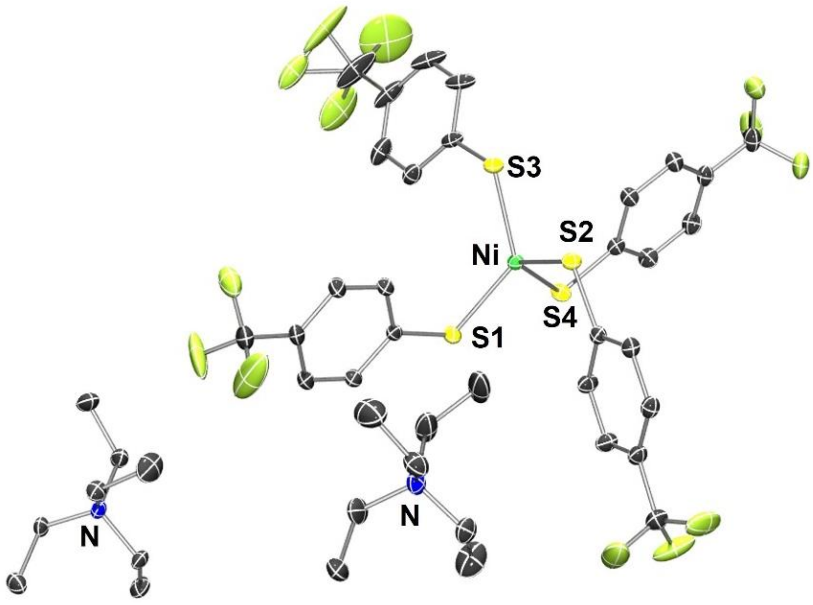

Figure S32. ORTEP diagram of $(Et_4N)_2[Ni(S\text{-}p\text{-}CF_3\text{-}Ph)_4]$ at 50% thermal probability ellipsoids for all non-hydrogen atoms. Disorder in one of the trifluoromethyl (CF_3) groups consisting of F7-F9 is shown.

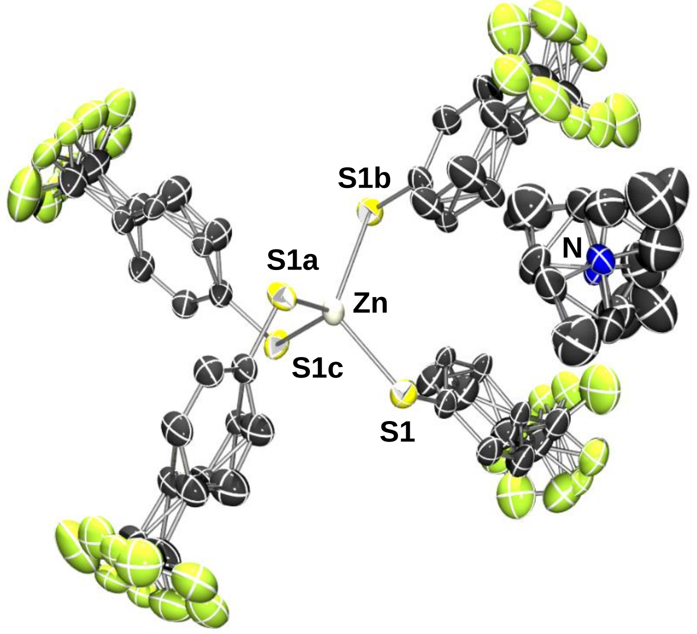

Figure S33. ORTEP diagram of $(Et_4N)_2[Zn(S\text{-}p\text{-}CF_3\text{-}Ph)_4]$ at 50% thermal probability ellipsoids for all non-hydrogen atoms. Disorder occurs in all trifluoromethyl-phenyl groups (Zn sits on a center of inversion) and in the C-atoms of the Et_4N^+ cations. These positions (% occupancy) have been effectively modeled as described in Experimental section 1.4 of the SI.

Table S2. Selected bond distances (Å) and bond angles (deg) for $(Et_4N)_2[Ni(S\text{-}p\text{-}CF_3\text{-}Ph)_4]$ (**1**) and $(Et_4N)_2[Zn(S\text{-}p\text{-}CF_3\text{-}Ph)_4]$ (**2**).

**1**		**2**	
**Atoms**	**Bond Distance (Å)**	**Atoms**	**Bond Distance (Å)**
Ni-S_1	2.290	Zn-S_1	2.3500
Ni-S_2	2.266		
Ni-S_3	2.275		
Ni-S_4	2.296		
**Atoms**	**Bond Angle (°)**	**Atoms**	**Bond Angle (°)**
S_1-Ni-S_2	87.8	S_1-Zn-S_{1a}	105.6
S_1-Ni-S_3	122.3	S_1-Zn-S_{1b}	117.5
S_1-Ni-S_4	113.5	S_1-Zn-S_{1c}	105.6
S_2-Ni-S_3	124.7		
S_2-Ni-S_4	124.5		
S_3-Ni-S_4	87.3		
**∠Ar-(Ni-S-C)**	**Angle (°)**		
Ar_1-(Ni-S_1)	1.2°		
Ar_2-(Ni-S_2)	5.0°		
Ar_3-(Ni-S_3)	38.0°		
Ar_4-(Ni-S_4)	6.5°		

Table S3. Summary of best fit simulation of EXAFS of **1**.

	**Nearest-Neighbor Ligand Environment [a]**				
**Sample**	**Atom [c]**	**R(Å) [d]**	**C.N. [e]**	**σ^2 [f]**	**F' [g]**
**1**	S	2.274	4	3.16	0.399

[a]/[b] - Independent metal-ligand scattering environments.
[c] - Scattering atoms: N (nitrogen), O (oxygen), C (carbon.
[d] - Metal-ligand bond length.
[e] - Metal-ligand coordination number.
[f] - Debye-Waller factor (Å x 10^3).
[g] - Number of degrees of freedom weighted mean square deviation between data and fit.

3. DFT (Geometry Optimization, MOs) and TD-DFT:

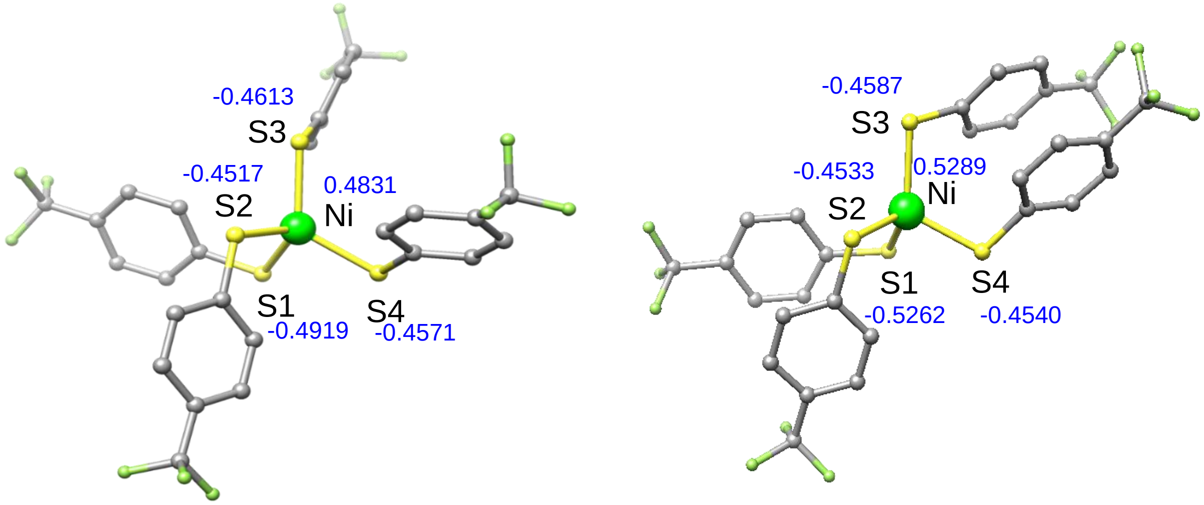

Figure S34. Geometry-optimized structures of the tetrahedral (S = 1) dianion of **1**. Gas-phase (left) and in MeCN (right). Input coordinates taken from the X-ray crystal structure of **1**. Mulliken atomic charges on Ni and S atoms are shown in blue.

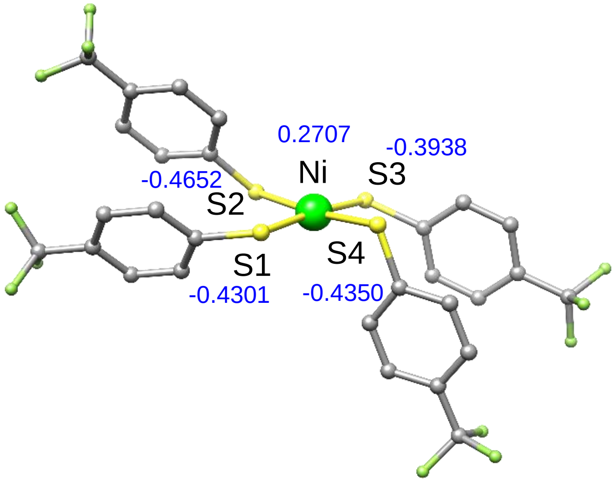

Figure S35. Geometry-optimized structure of the planar (S = 0) dianion of **1** in MeCN. Input coordinates taken from the crystal structure of **1** with the aryl-thiolato ligands positioned in an approximate square-planar geometry. Mulliken atomic charges on Ni and S atoms are shown in blue.

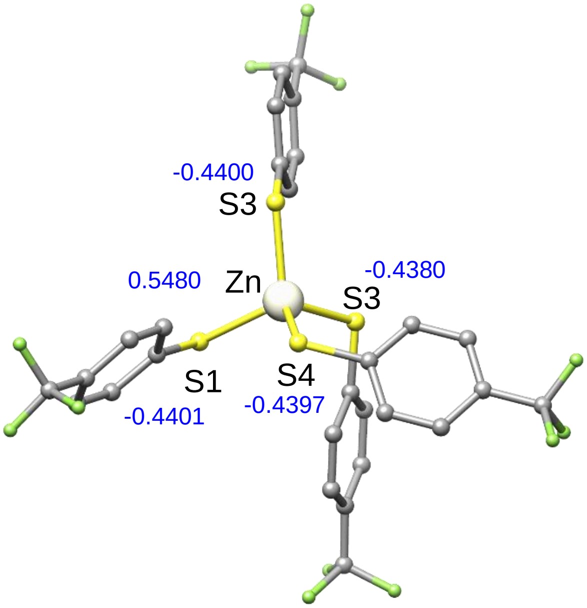

Figure S36. Geometry-optimized structure of the tetrahedral dianion of **2** in the gas-phase. Input coordinates taken from the X-ray crystal structure of **2**. Mulliken atomic charges on Zn and S atoms are shown in blue.

Table S4. Selected bond distances (Å) and bond angles (°) for geometry-optimized structures of the dianions of **1** (S = 0, S = 1) and **2**.

	**1** (S = 1, Gas)	**1** (S = 1, MeCN)	**1** (S = 0, MeCN)	**2** (S = 0, Gas)
	**Bond Distance (Å)**			
	M = Ni			M = Zn
M-S_1	2.323	2.331	2.251	2.392
M-S_2	2.335	2.349	2.252	2.403
M-S_3	2.332	2.312	2.266	2.385
M-S_4	2.320	2.336	2.254	2.392
	**Bond Angle (°)**			
S_1-M-S_2	119.5	110.7	98.7	113.1
S_1-M-S_3	118.6	91.7	175.0	102.4
S_1-M-S_4	94.4	130.1	84.2	105.9
S_2-M-S_3	90.1	112.0	82.9	111.5
S_2-M-S_4	129.2	99.0	94.7	106.0
S_3-M-S_4	118.6	113.6	173.9	112.9
	$\boldsymbol{\tau_4}$			
	0.79	0.82	0.08	0.95
**∠Ar-(Ni-S-C)**	**Angle (°)**			
Ar_1-(Ni-S_1)	23.6	59.6		
Ar_2-(Ni-S_2)	11.2	30.3		
Ar_3-(Ni-S_3)	26.4	52.5		
Ar_4-(Ni-S_4)	23.6	30.2		

Table S5. Löwdin population analysis derived from the DFT computations (B3LYP/def2-TZVPP) for selected MOs of tetrahedral **1** ($S = 1$), with an MeCN solvent model. Only s and p contributions were tabulated for S-atoms.

**α Orbitals**	**MO#**	**Energy (eV)**	**%Ni**	**%S**
LUMO + 3	198	-0.5305	0.4	6.1
LUMO + 2	197	-0.6036	1.0	6.2
LUMO + 1	196	-0.6363	1.2	5.7
LUMO	195	-0.7468	2.1	3.5
HOMO	194	-4.9573	10.6	55.1
HOMO - 1	193	-5.0063	9.6	55.2
HOMO - 2	192	-5.2089	8.3	60.7
HOMO - 3	191	-5.2154	2.3	64.5

**β Orbitals**	**MO#**	**Energy (eV)**	**%Ni**	**%S**
LUMO + 3	196	-0.4938	1.1	9.3
LUMO + 2	195	-0.7033	16.2	4.8
LUMO + 1	194	-1.8772	70.2	18.0
LUMO	193	-1.9242	63.3	13.6
HOMO	192	-4.8433	11.9	51.5
HOMO - 1	191	-5.1047	1.4	60.4
HOMO - 2	190	-5.2702	24.5	51.9
HOMO - 3	189	-5.4739	7.3	45.8

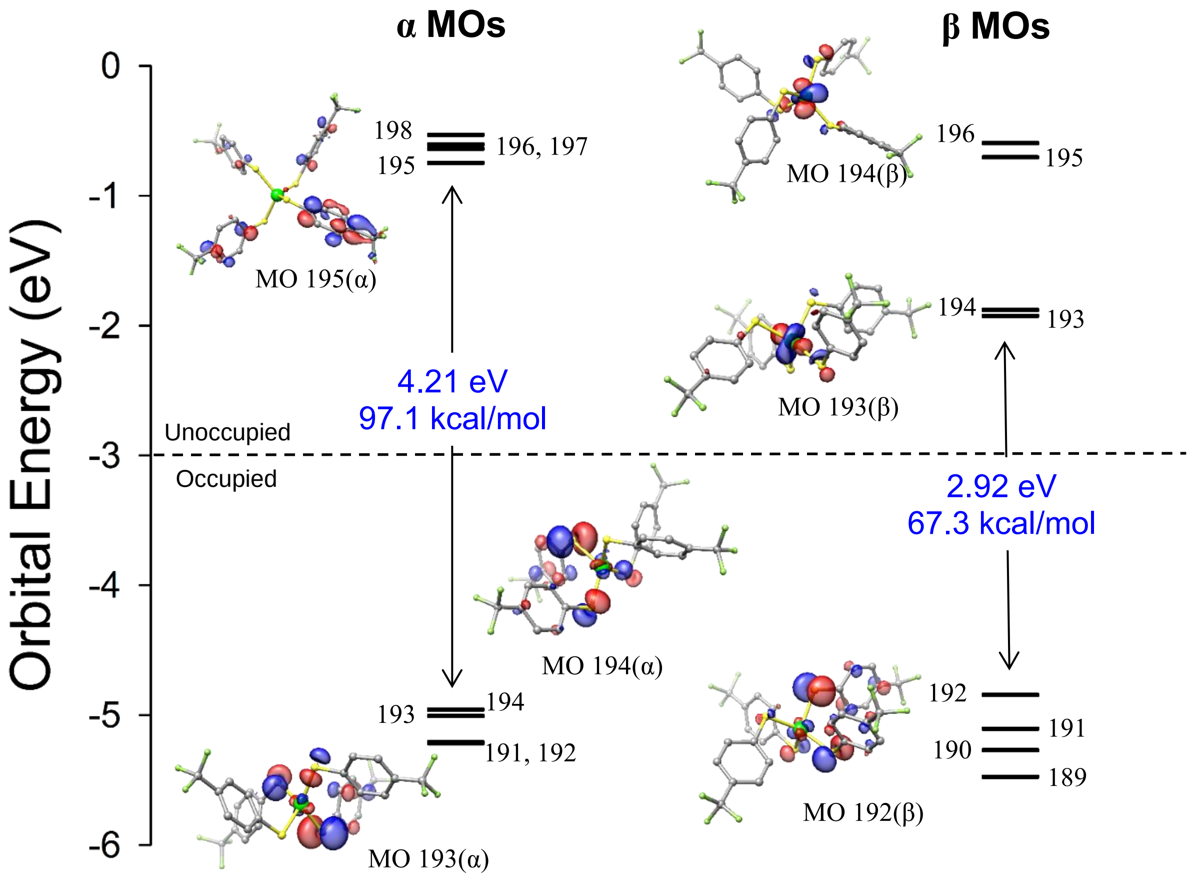

Figure S37. DFT-generated isosurface plots of the frontier MOs for tetrahedral ($S = 1$) **1** with an MeCN solvent model. Dashed line indicates the level below which MOs are occupied. Spin-up (α) MOs (*left*); spin-down (β) MOs (*right*). See Table S5 for contributions.

Table S6. Löwdin population analysis derived from the DFT computations (B3LYP/def2-TZVPP) for selected MOs of square-planar **1** (S = 0), with an MeCN solvent model. Only s and p contributions were tabulated for S-atoms.

Orbitals	MO#	Energy (eV)	%Ni	%S
LUMO + 3	197	-0.3373	1.2	5.3
LUMO + 2	196	-0.4232	0.8	4.5
LUMO + 1	195	-0.5161	3.2	5.0
LUMO	194	-1.2968	39.2	23.9
HOMO	193	-4.5471	34.7	52.2
HOMO - 1	192	-4.6915	17.7	52.9
HOMO - 2	191	-4.8444	8.6	65.4
HOMO - 3	190	-5.1671	83.8	12.8

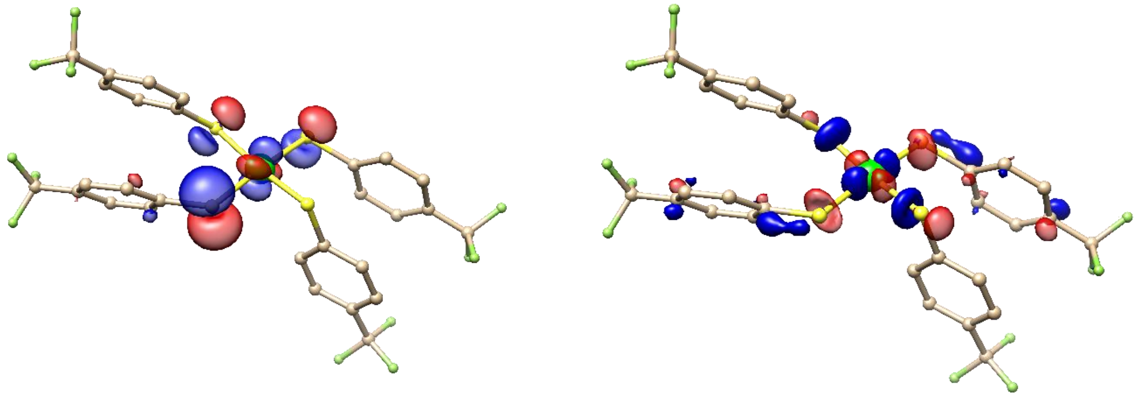

Figure S38. DFT-generated isosurface plots (B3LYP/def2-TZVPP) of the frontier MOs of planar (S = 0) **1**: MO 193 = HOMO (left) and MO 194 = LUMO (right). See Table S6 for contributions.

Table S7. Löwdin population analysis derived from the DFT computations (B3LYP/def2-TZVPP) for selected MOs of **2** ($S = 0$), with MeCN solvent model. Only s and p contributions were tabulated for S-atoms.

Orbitals	MO#	Energy (eV)	%Zn	%S
LUMO + 3	198	-0.5307	1.5	6.0
LUMO + 2	197	-0.5305	2.0	5.1
LUMO + 1	196	-0.5476	1.8	2.1
LUMO	195	-0.5622	2.5	4.8
HOMO	194	-5.0965	5.0	63.6
HOMO - 1	193	-5.1441	5.0	62.4
HOMO - 2	192	-5.1754	4.1	62.0
HOMO - 3	191	-5.2513	5.5	64.6

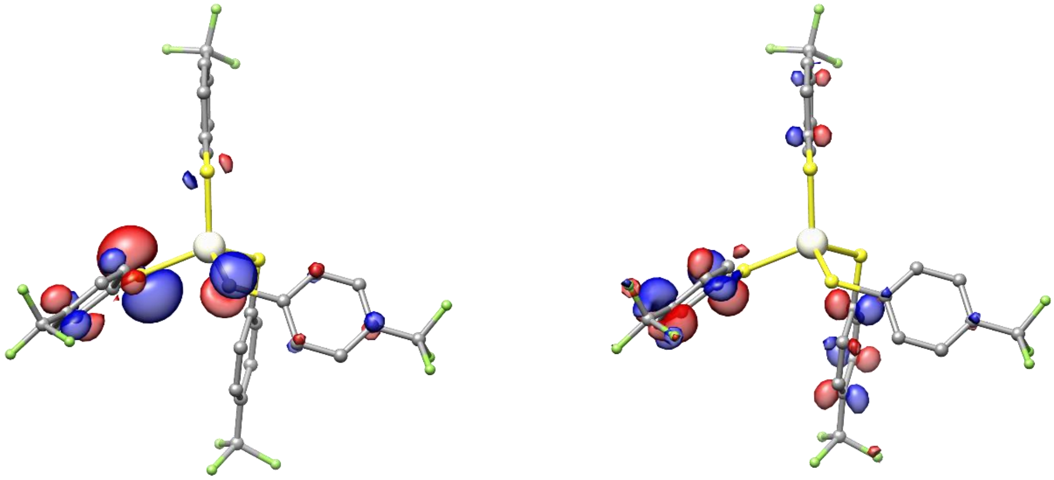

Figure S39. DFT-generated isosurface plots (B3LYP/def2-TZVPP) of the frontier MOs of **2**: MO 194 = HOMO (left) and MO 195 = LUMO (right). See Table S7 for contributions.

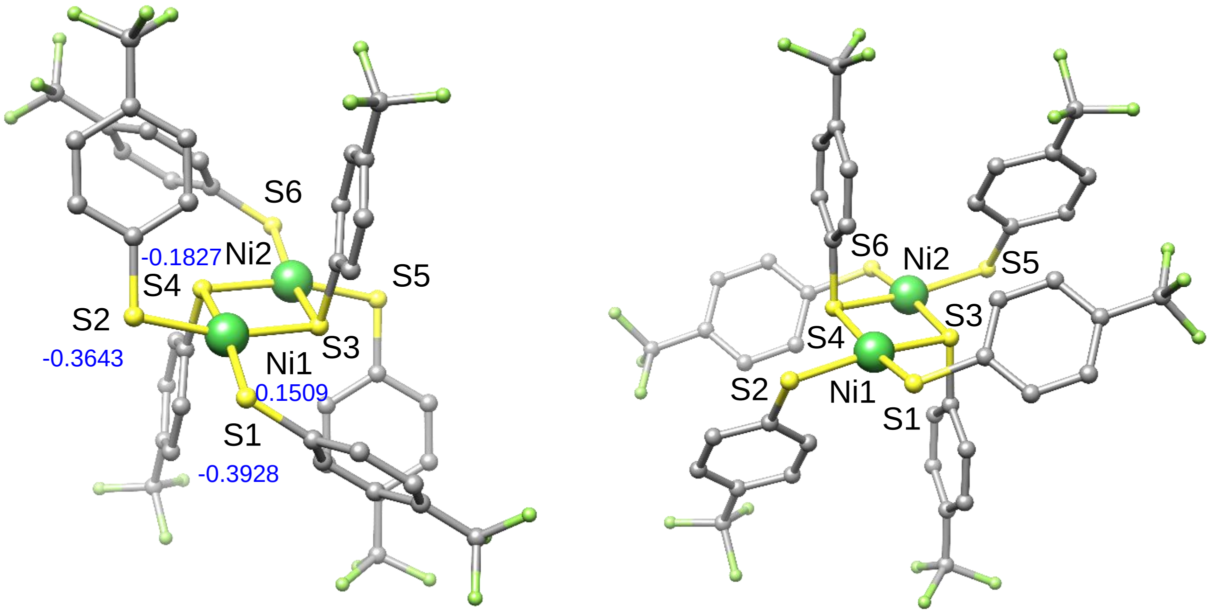

Figure S40. Geometry-optimized structure of the planar (S = 0) dianion of **3** in MeCN. Input coordinates taken from the X-ray crystal structure of the *p*-Cl-PhS⁻ analog of **3** (Maroney, M. J. and coworkers, *Inorg. Chem.* **1990**, *28*, 4779). Mulliken atomic charges on Ni and S atoms are shown in blue. Two different orientations are displayed.

Table S8. Selected bond distances (Å) and bond angles (deg) for geometry-optimized structure of the dianion of **3** (S = 0) in MeCN.

Atoms	Bond Distance	Atoms	Angle
Ni_1-S_1	2.211	S_1-Ni_1-S_2	90.1
Ni_1-S_2	2.243	S_1-Ni_1-S_3	99.2
Ni_1-S_3	2.246	S_1-Ni_1-S_4	169.7
Ni_1-S_4	2.247	S_2-Ni_1-S_3	164.5
Ni_2-S_3	2.247	S_2-Ni_1-S_4	89.9
Ni_2-S_4	2.246	S_3-Ni_1-S_4	83.1
Ni_2-S_5	2.211	S_3-Ni_2-S_4	83.1
Ni_2-S_6	2.243	S_3-Ni_2-S_5	89.9
Ni_1---Ni_2	3.363	S_3-Ni_2-S_6	169.7
		S_4-Ni_2-S_5	164.4
		S_4-Ni_2-S_6	99.1
		S_5-Ni_2-S_6	90.3
	τ_4	Ni_1	0.18

Table S9. Löwdin population analysis derived from the DFT computations (B3LYP/def2-TZVPP) for selected MOs of **3** ($S = 0$), with MeCN solvent model. Only s and p contributions were tabulated for S-atoms.

Orbitals	MO#	Energy (eV)	%Ni	%S
LUMO + 3	299	-0.8315	4.8	5.7
LUMO + 2	298	-0.8782	8.2	10.1
LUMO + 1	297	-1.7137	35.0	23.2
LUMO	296	-1.8672	41.9	21.4
HOMO	295	-5.0963	22.8	54.1
HOMO - 1	294	-5.1292	15.3	55.4
HOMO - 2	293	-5.2043	15.2	58.2
HOMO - 3	292	-5.2129	26.1	53.7

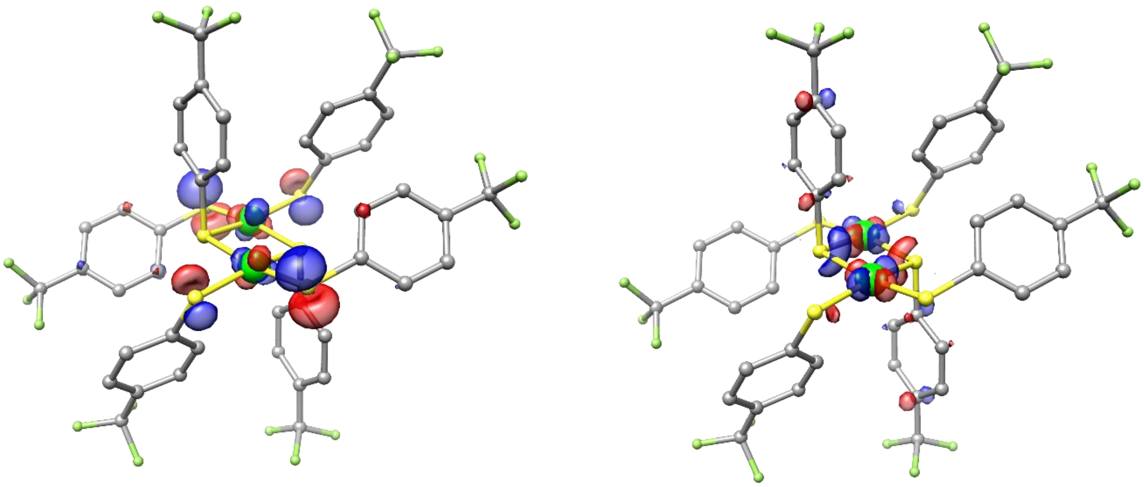

Figure S41. DFT-generated isosurface plots (B3LYP/def2-TZVPP) of the frontier MOs of **3**: MO 295 = HOMO (left) and MO 296 = LUMO (right). See Table S9 for contributions.

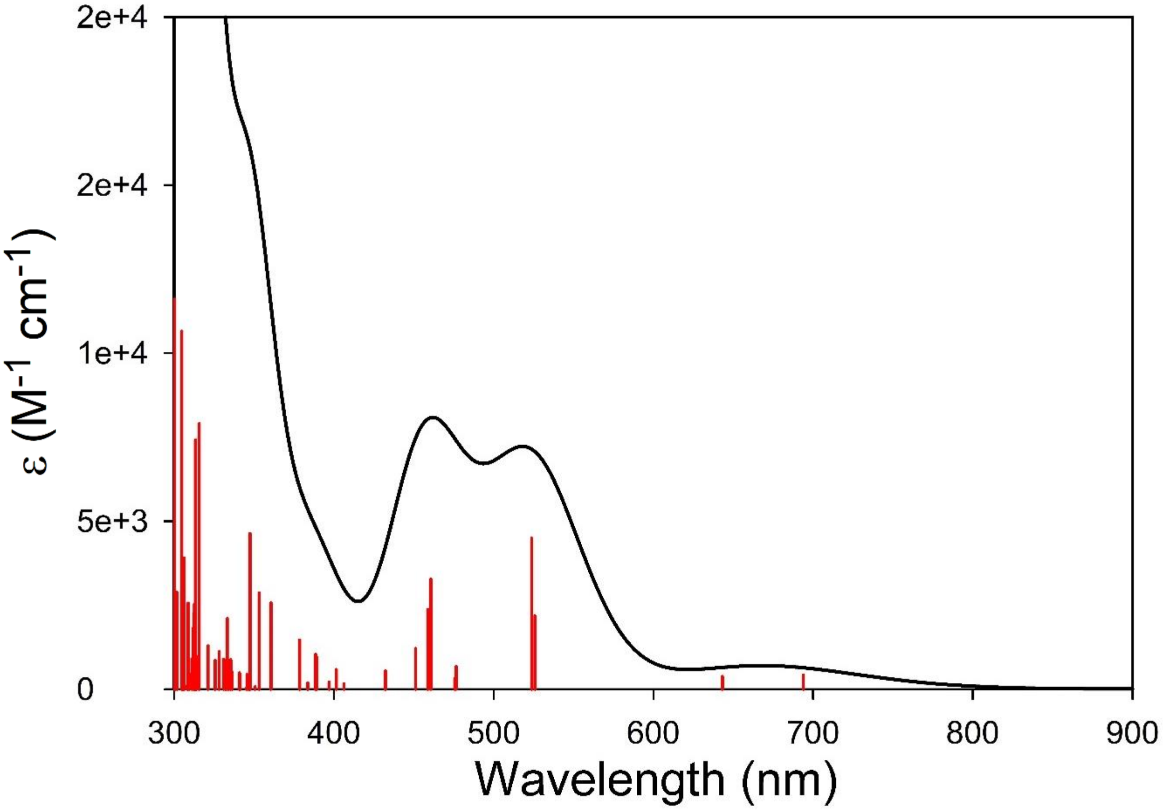

Figure S42. TD-DFT calculated optical absorption spectrum of **1*** with Gaussian (black) distribution (black line) and optical transitions (red sticks).

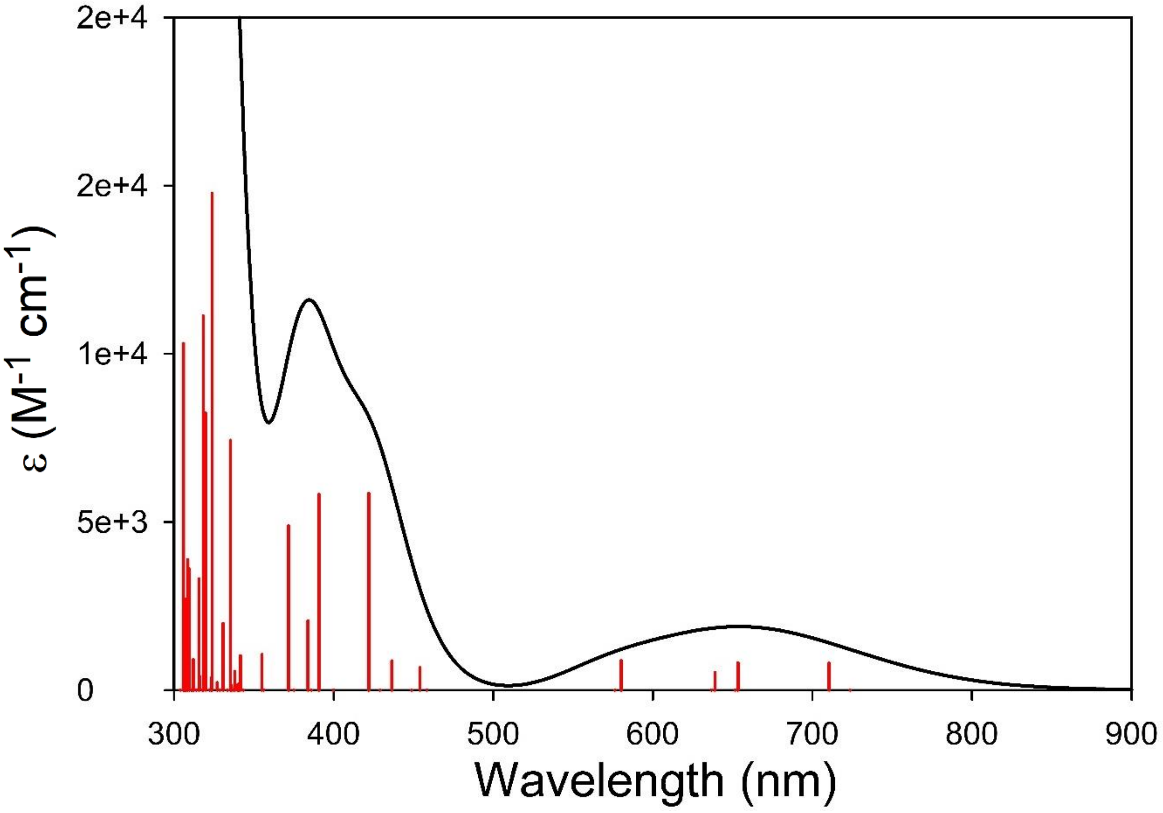

Figure S43. TD-DFT calculated optical absorption spectrum of **3*** with Gaussian (black) distribution (black line) and optical transitions (red ticks).

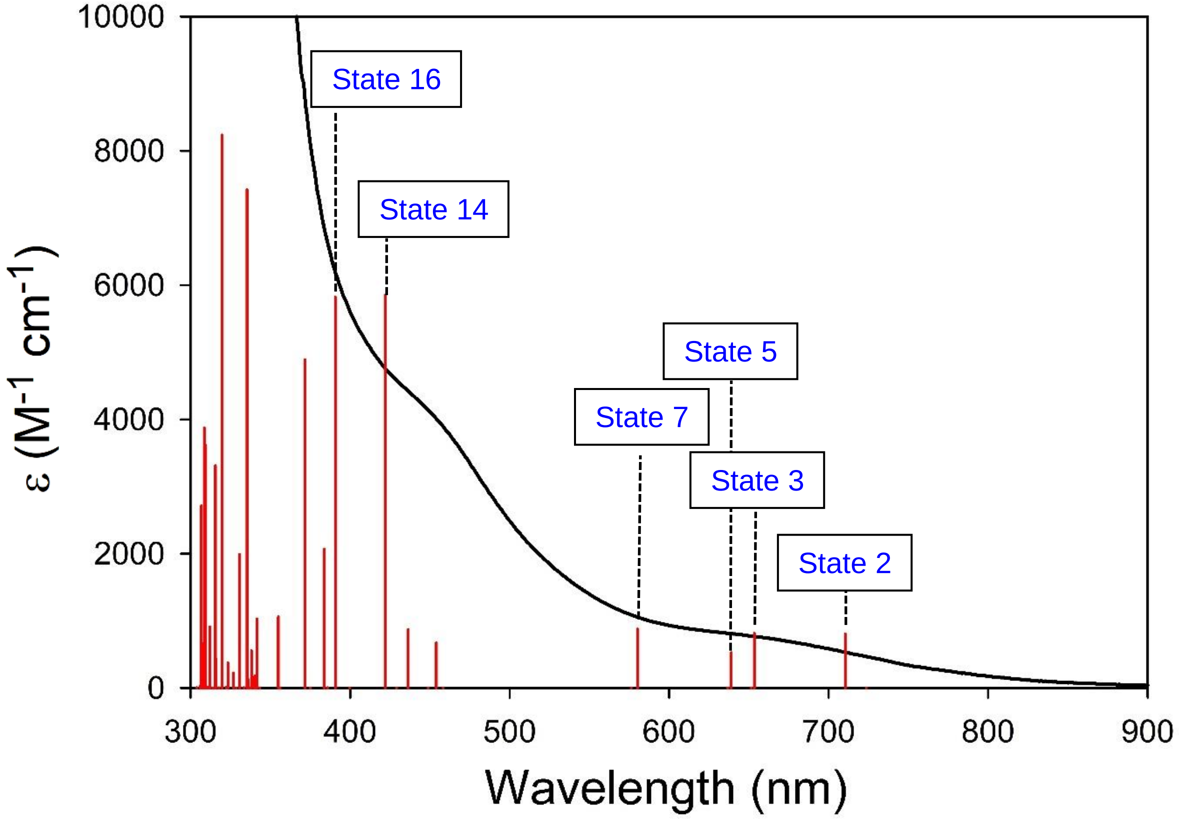

Figure S44. UV-vis spectrum of the thiolate-bridged mixture containing the dianion of **3** in MeCN (black trace) superimposed with TD-DFT computed optical transitions for **3*** (red sticks). Experimental ε for thiolate-bridged mixture containing **3** is estimated by assuming the isolated product is exclusively $[Ni_2(S\text{-}p\text{-}CF_3\text{-}Ph)_6]^{2-}$.

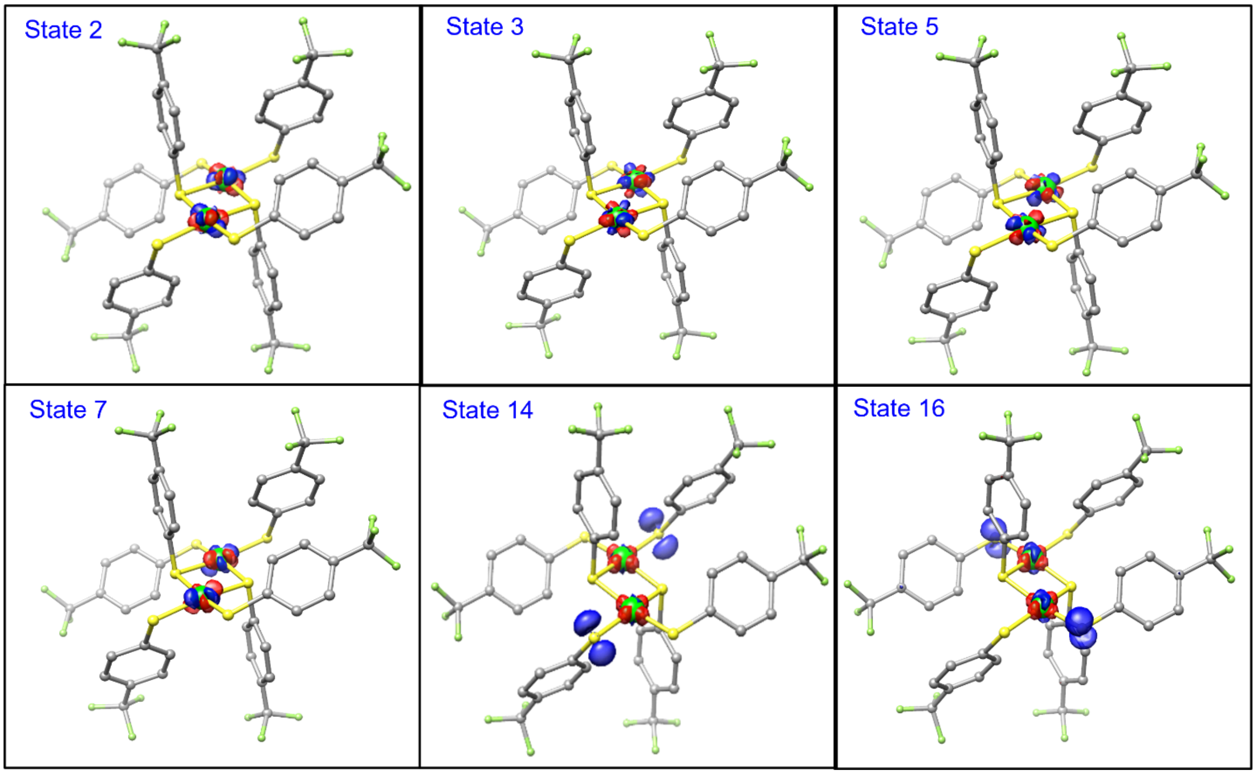

Figure S45. Electron difference densities for select transitions of **3***, shown in Figure S44, blue lobes reflect loss of electron density, while red lobes reflect increased electron density in the transitions.

4. Spectroscopic Characterization of Speciation:

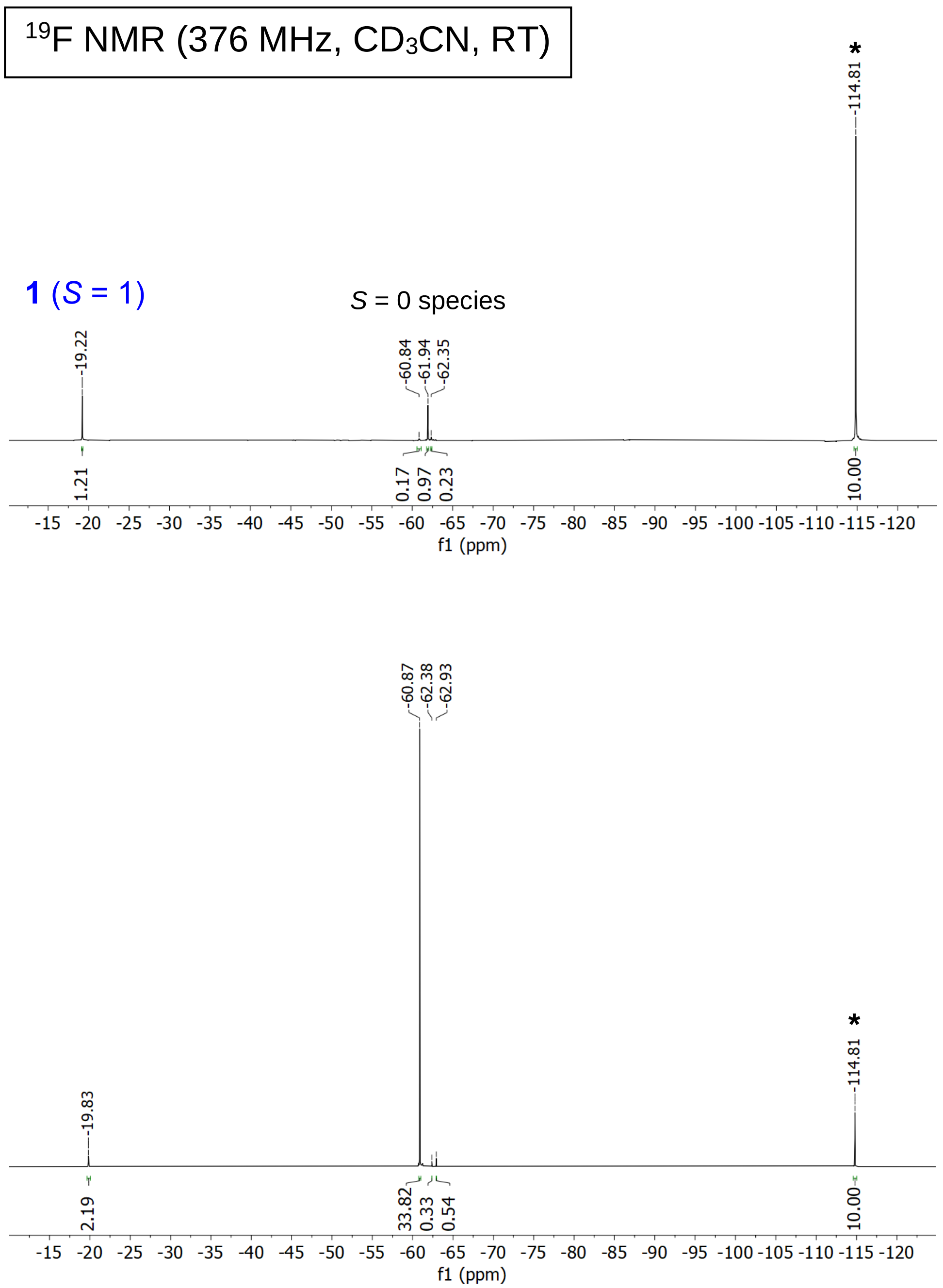

Figure S46. ^{19}F NMR of as-isolated **1** (2.0 mM) (*top*) and with 20 mol-equiv (Et_4N)(S-*p*-CF_3-Ph) (*bottom*) in CD_3CN (δ vs. $CFCl_3$). Mononuclear **1** is estimated based on the relative integration of the δ ~-19 ppm peak assigned to **1** (*S* = 1) before and after thiolate addition. Peak at -114.81 ppm is PhF (internal standard; assigned an arbitrary integration value) as indicated with *.

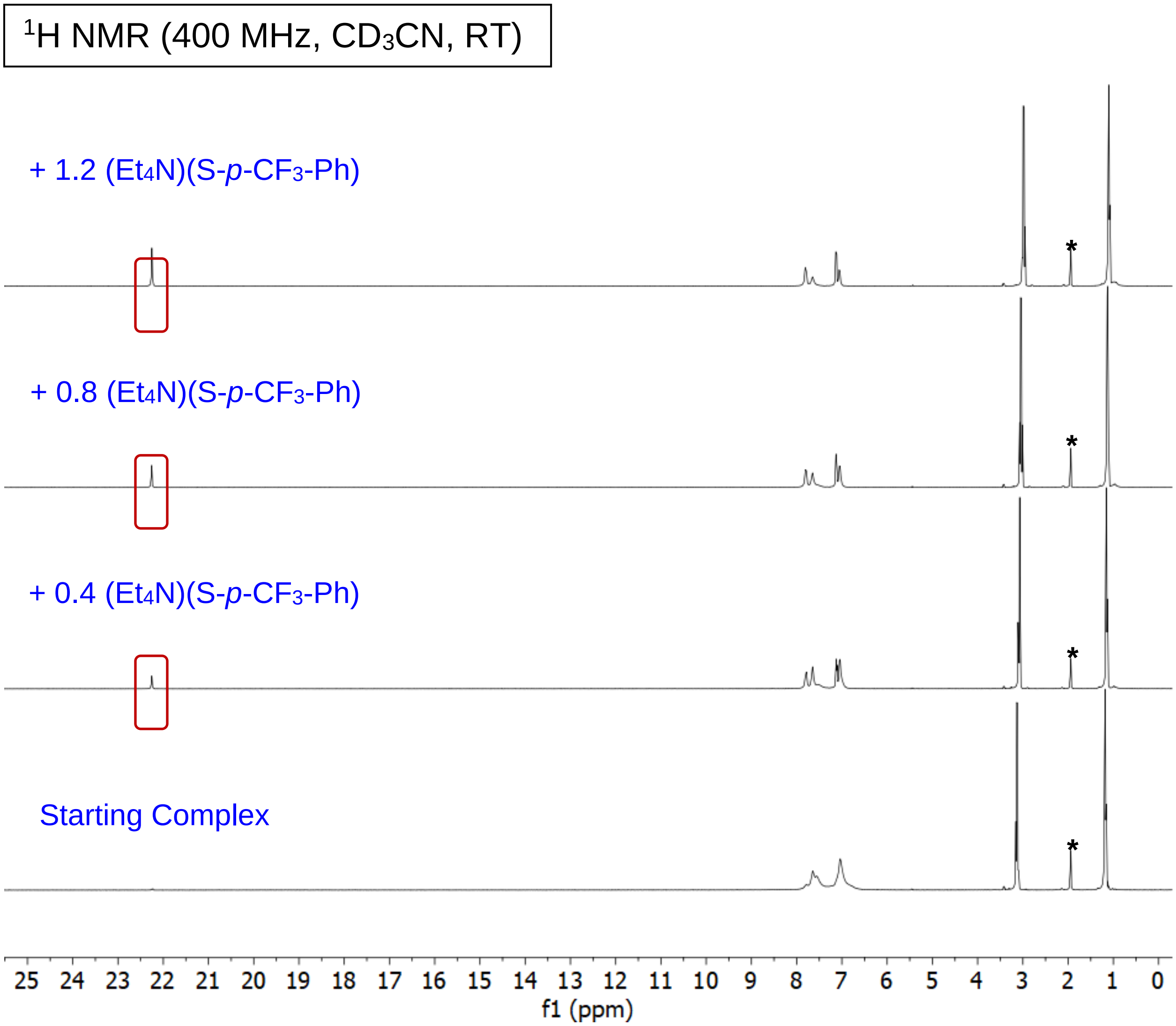

Figure S47. ^{1}H NMR of thiolate-bridged Ni complexes, including **3**, with various amounts of $(Et_4N)(S\text{-}p\text{-}CF_3\text{-}Ph)$ in CD_3CN at RT (δ vs. protio signal (*) at 1.94 ppm; equiv estimated by assuming mixture is exclusively **3**). Zoom-in of ~7 ppm region is shown in Figure S48. *Note*: growth of peak at ~22 ppm (red box) is due to formation of tetrahedral complex **1**.

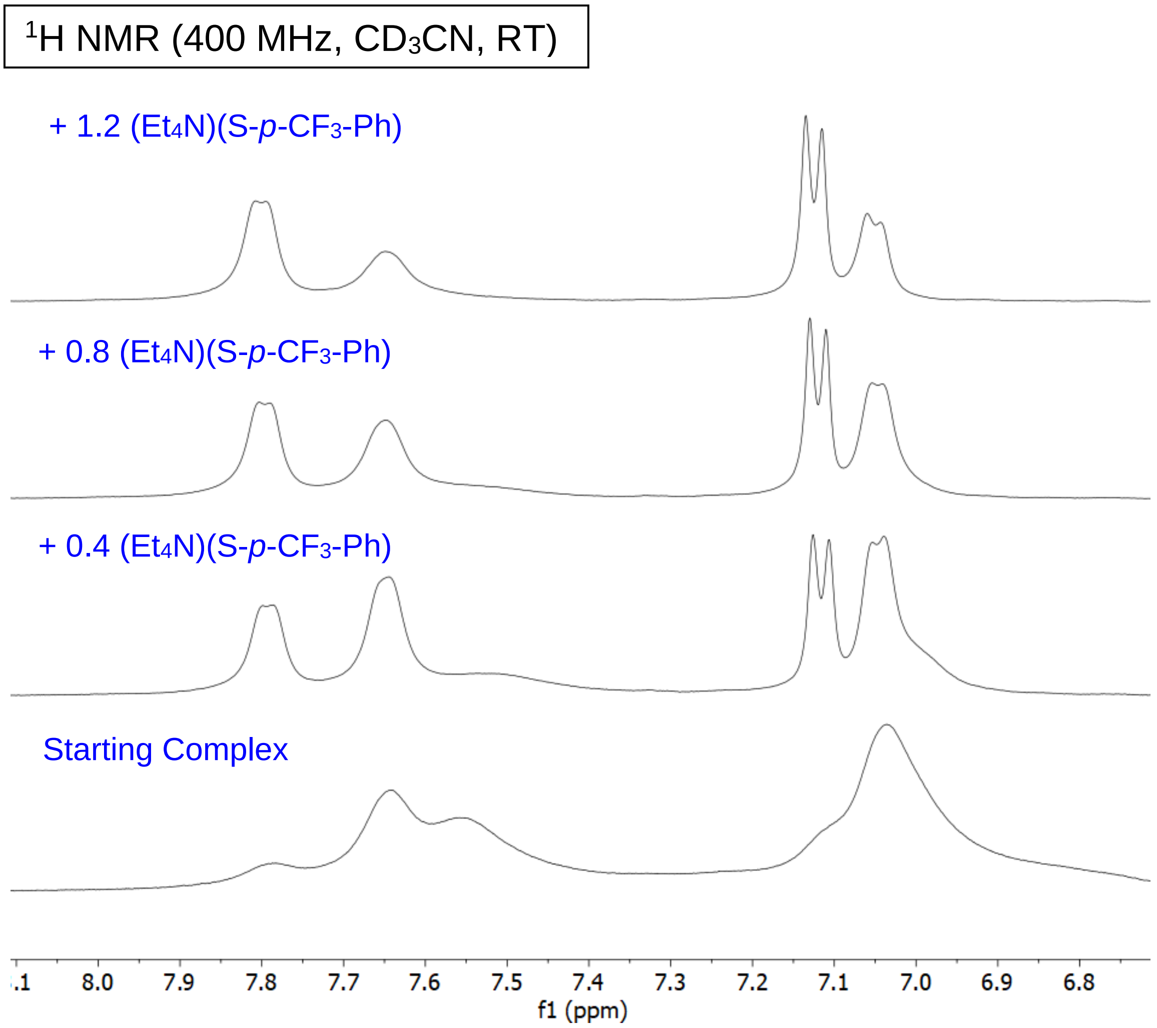

Figure S48. Zoom-in of the ~7 ppm region of the ^{1}H NMR of thiolate-bridged Ni complexes, including **3**, with various amounts of $(Et_4N)(S$-*p*-CF_3-Ph) in CD_3CN at RT (δ vs. protio signal (*) at 1.94 ppm; equiv estimated by assuming mixture is exclusively **3**). Full spectrum is shown in Figure S47.

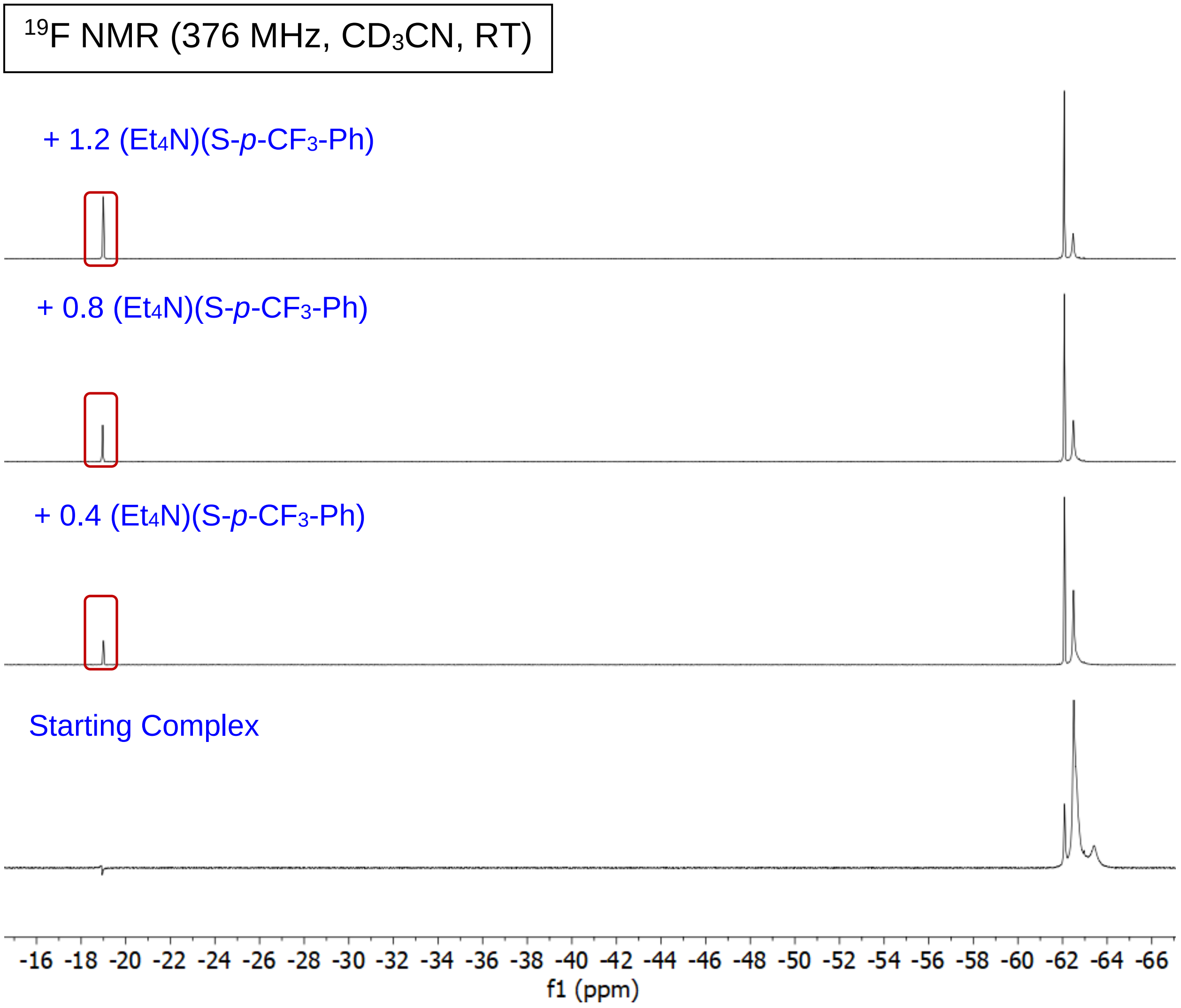

Figure S49. ^{19}F NMR of thiolate-bridged Ni complexes, including **3**, with various amounts of $(Et_4N)(S\text{-}p\text{-}CF_3\text{-}Ph)$ in CD_3CN at RT (equiv estimated by assuming mixture is exclusively **3**). *Note*: growth of peak at -19 ppm (red box) is due to formation of tetrahedral complex **1**.

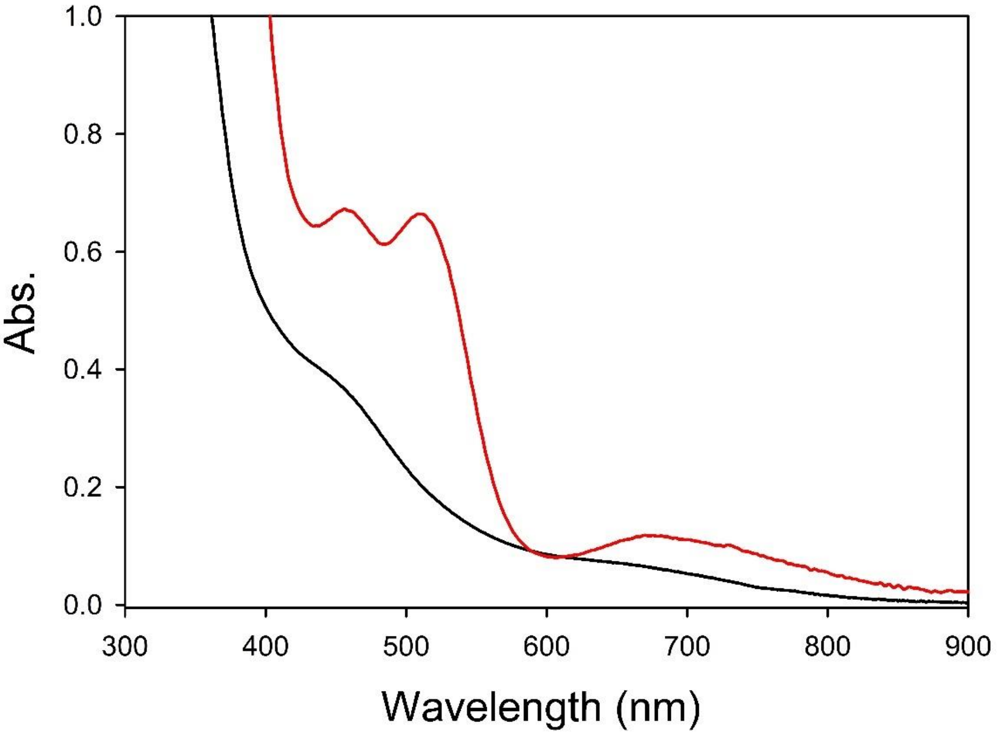

Figure S50. Qualitative UV-vis spectrum of thiolate-bridged Ni complexes, including **3** (black trace), and after adding 20 mol-equiv $(Et_4N)(S$-*p*-CF_3-Ph) (red trace; equiv estimated by assuming mixture is exclusively **3**) in MeCN at 25 °C. *Note*: appearance of CT bands at 460 and 510 nm is due to formation of tetrahedral complex **1**.

5. DFT Analysis on Solution Speciation:

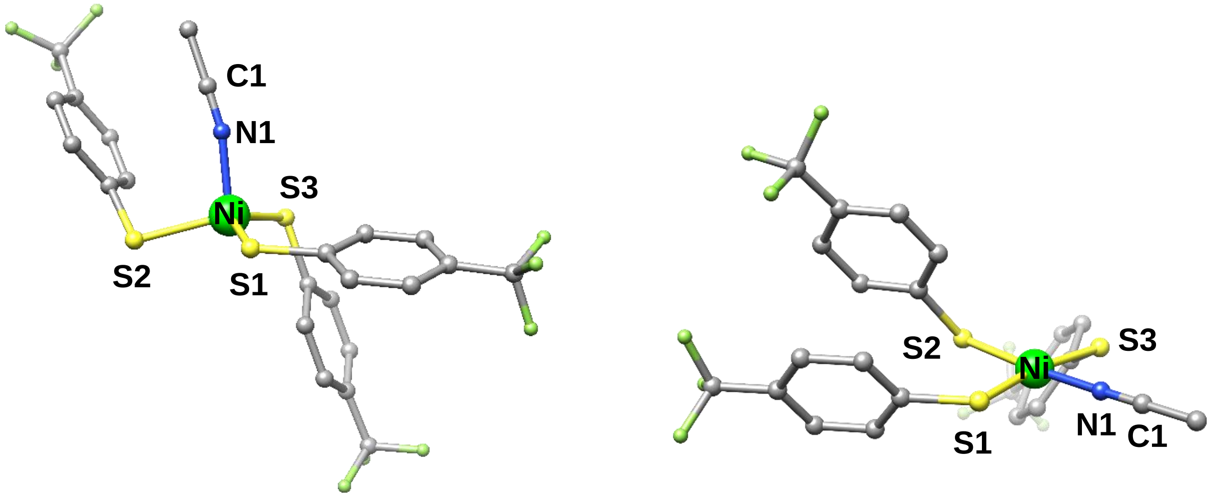

Figure S51. Geometry-optimized structures of putative Ni^{2+} intermediates with the general formula $[Ni(S\text{-}p\text{-}CF_3\text{-}Ph)_3(MeCN)]^-$ (**I**) as the tetrahedral complex anion (S = 1; *left*); and as the square-planar complex anion (S = 0; *right*). B3LYP/def2-TZVPP (CPMC to model MeCN).

Table S10. Selected bond distances (Å) and angles (deg) for geometry-optimized structures of putative Ni^{2+} intermediates with the general formula $[Ni(S\text{-}p\text{-}CF_3\text{-}Ph)_3(MeCN)]^-$ (**I**). B3LYP/def2-TZVPP (CPMC to model MeCN).

**$[Ni(S\text{-}p\text{-}CF_3\text{-}Ph)_3(MeCN)]^-$**	***S* = 1**	***S* = 0**
**Atoms**	**Bond Distance (Å)**	
$Ni\text{-}S_1$	2.289	2.261
$Ni\text{-}S_2$	2.335	2.208
$Ni\text{-}S_3$	2.289	2.262
$Ni\text{-}N_1$	2.003	1.900
$N_1\text{-}C_1$	1.147	1.148
	**Bond Angle (deg)**	
$S_1\text{-}Ni\text{-}S_2$	93.5	97.8
$S_1\text{-}Ni\text{-}S_3$	126.5	169.7
$S_1\text{-}Ni\text{-}N_1$	110.5	84.0
$S_2\text{-}Ni\text{-}S_3$	129.0	92.2
$S_2\text{-}Ni\text{-}N_1$	99.5	176.4
$S_3\text{-}Ni\text{-}N_1$	94.1	86.1
$Ni\text{-}N_1\text{-}C_1$	166.1	179.0
	**τ_4**	
	0.74	0.08

Table S11. Final single point energies from geometry-optimization (B3LYP/def2-TZVPP) with the CPMC model for MeCN of selected complexes.

Compound	*S*	Final Single Point Energy (E_h)
**MeCN**	0	-132.657217321421
***p*-CF$_3$-PhS$^–$**	0	-966.696932471075
**[Ni(S-*p*-CF$_3$-Ph)$_4$]$^{2–}$** (tetrahedral dianion of **1**)	1	-5374.96479120396
**[Ni(S-*p*-CF$_3$-Ph)$_4$]$^{2–}$** (planar dianion of **1**)	0	-5374.9594861337
**[Ni(S-*p*-CF$_3$-Ph)$_3$(MeCN)]$^–$** (tetrahedral anion of **I**)	1	-4540.90388629218
**[Ni(S-*p*-CF$_3$-Ph)$_3$(MeCN)]$^–$** (planar anion of **I**)	0	-4540.89966773846
[**Ni$_2$(S-*p*-CF$_3$-Ph)$_6$]$^{2–}$** (planar dianion of **3**)	0	-8816.5207723853

Table S12. Gibbs free energies from frequency calculations (B3LYP/def2-TZVPP) with the CPMC model for MeCN of selected complexes.

Compound	*S*	Gibbs Free Energy (E_h)
**MeCN**	0	-132.7254553
***p*-CF$_3$-PhS$^–$**	0	-966.928222
**[Ni(S-*p*-CF$_3$-Ph)$_4$]$^{2–}$** (tetrahedral dianion of **1**)	1	-5375.809771
**[Ni(S-*p*-CF$_3$-Ph)$_4$]$^{2–}$** (planar dianion of **1**)	0	-5375.787262
**[Ni(S-*p*-CF$_3$-Ph)$_3$(MeCN)]$^–$** (tetrahedral anion of **I**)	1	-4541.590423
**[Ni(S-*p*-CF$_3$-Ph)$_3$(MeCN)]$^–$** (planar anion of **I**)	0	-4541.586544
[**Ni$_2$(S-*p*-CF$_3$-Ph)$_6$]$^{2–}$** (planar dianion of **3**)	0	-8817.751314

6. Bulk Protonation Studies:

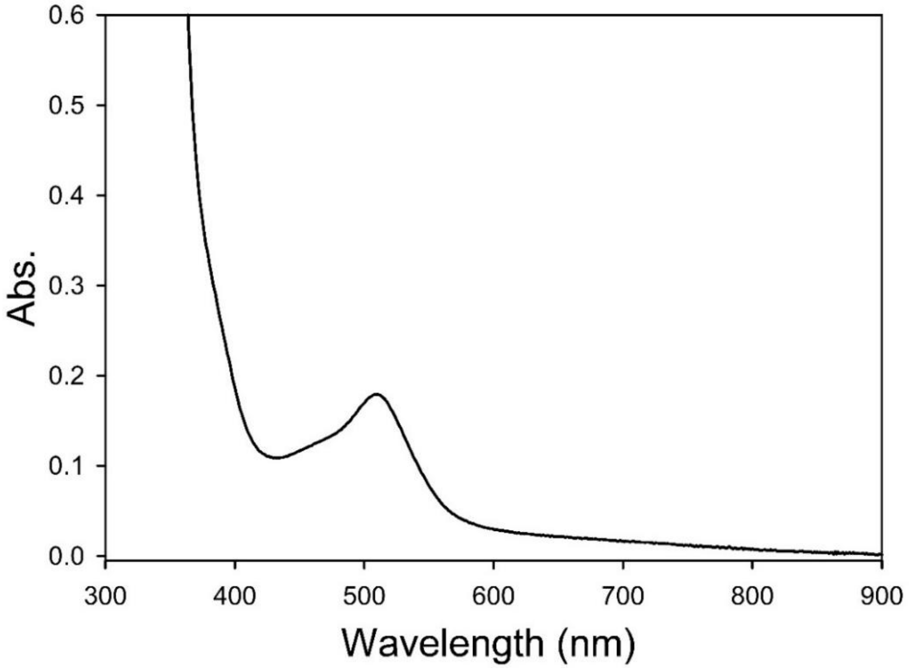

Figure S52. Qualitative UV-vis spectrum of the insoluble material isolated from the reaction of complex **1** with HBF_4•Et_2O (1:1) in MeCN. Spectrum recorded in DMSO at 25 °C.

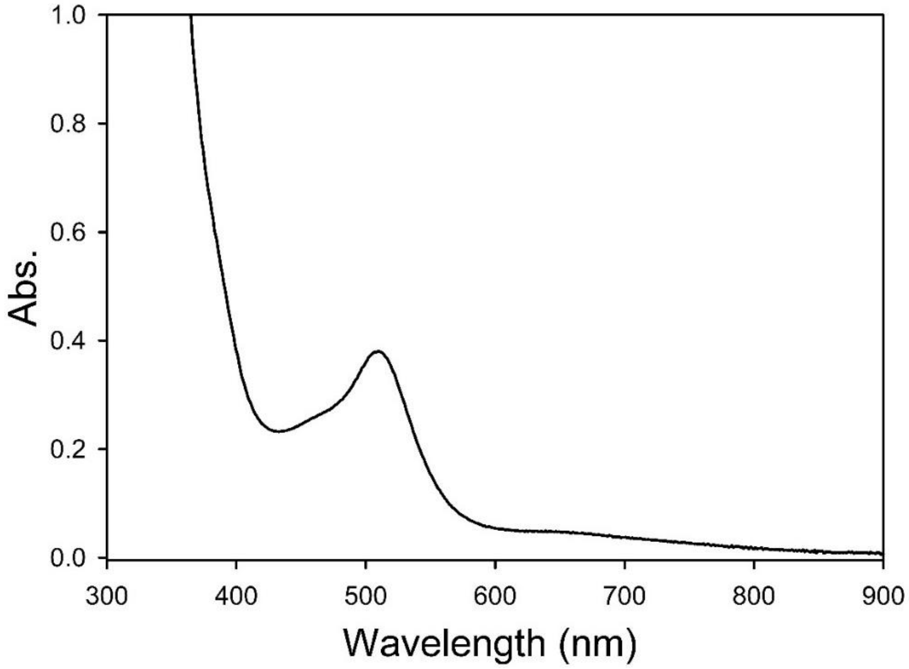

Figure S53. Qualitative UV-vis spectrum of the insoluble material isolated from the reaction of complex **1** with AcOH (1:25) in MeCN. Spectrum recorded in DMSO at 25 °C.

^{1}H NMR (400 MHz, DMSO-d_6, RT) of **1** + HBF_4•Et_2O (1:1)

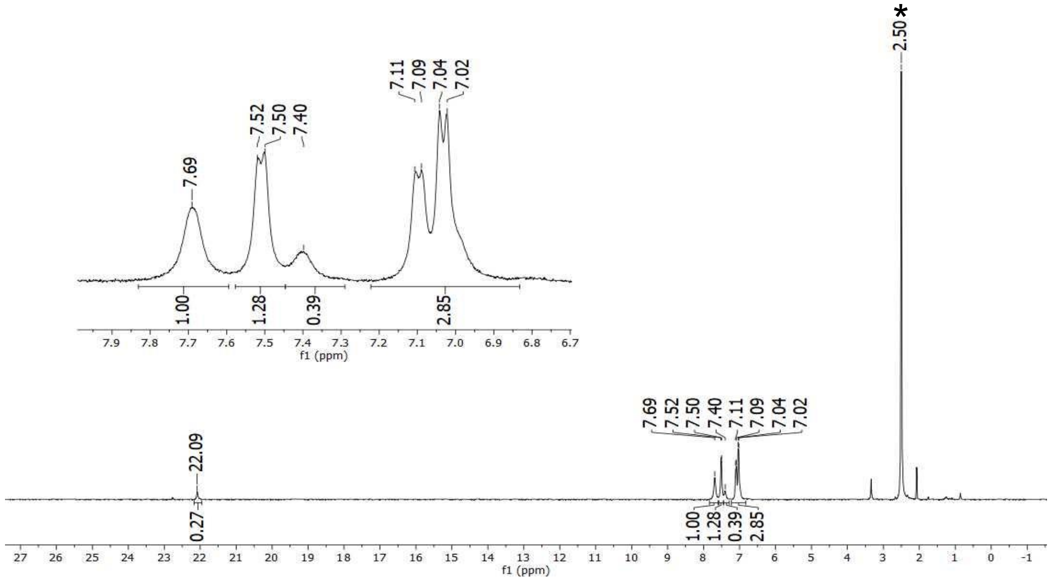

^{1}H NMR (400 MHz, DMSO-d_6, RT) of **1** + AcOH (1:25)

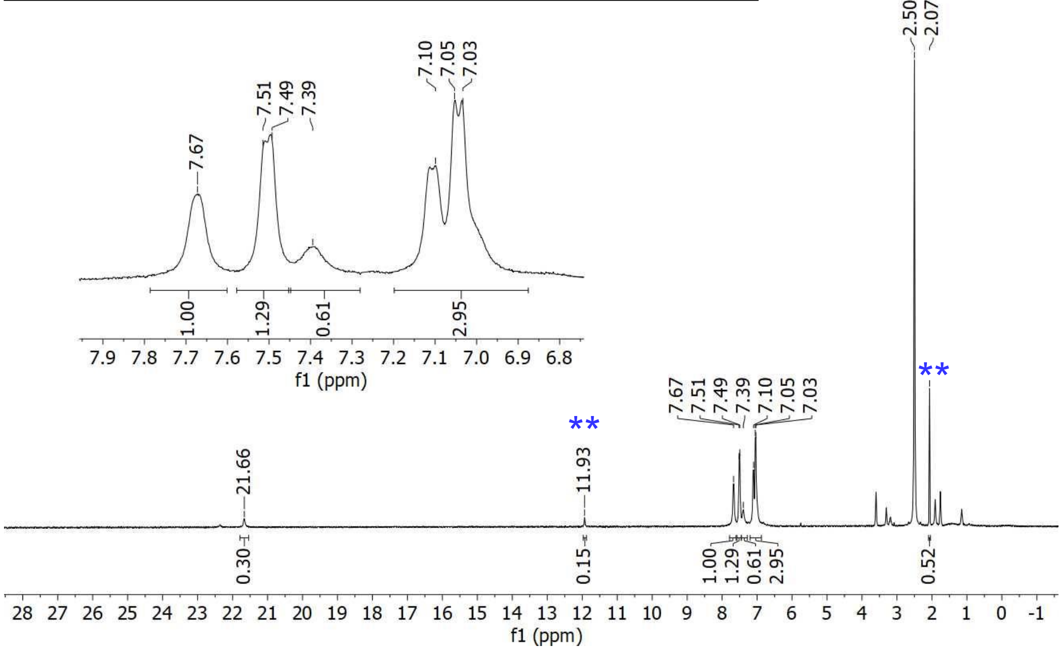

Figure S54. ^{1}H NMR spectrum of the insoluble material isolated from the reaction of complex **1** with HBF_4•Et_2O (1:1) (*top*) or **1** with AcOH (1:25) (*bottom*) in MeCN. Spectrum recorded in DMSO-d_6 at RT (δ vs. protio signal (*) at 2.50 ppm; residual protio acetic acid is observed (**) at 2.07 and 11.93 ppm in the bottom spectrum). *Insets*: Zoom-in of aromatic-H region.

^{19}F NMR (376 MHz, DMSO-d_6, RT) of **1** + HBF_4•Et_2O (1:1)

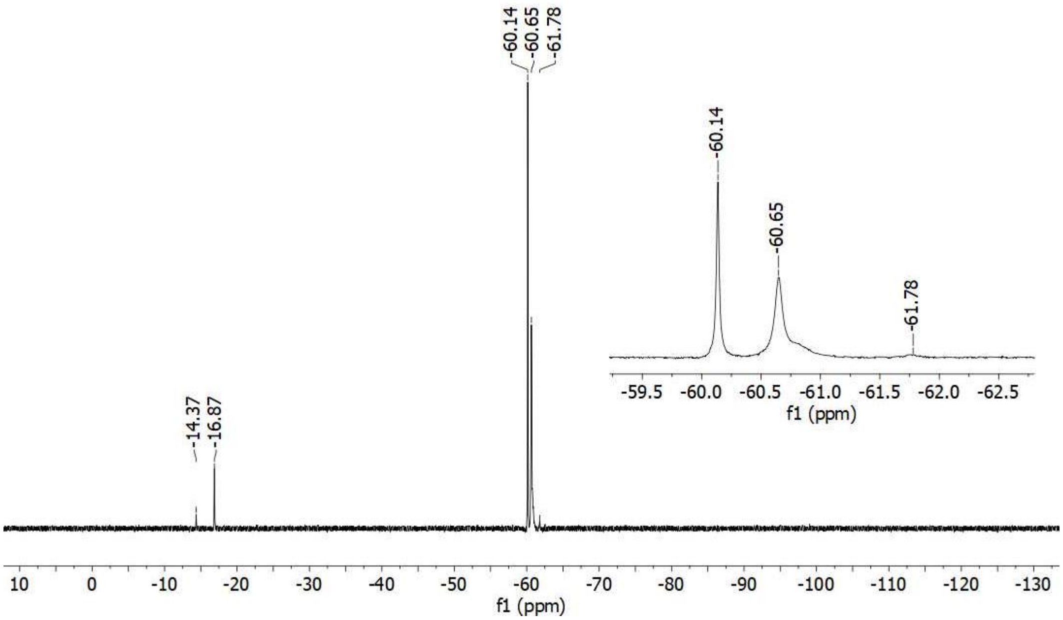

^{19}F NMR (376 MHz, DMSO-d_6, RT) of **1** + AcOH (1:25)

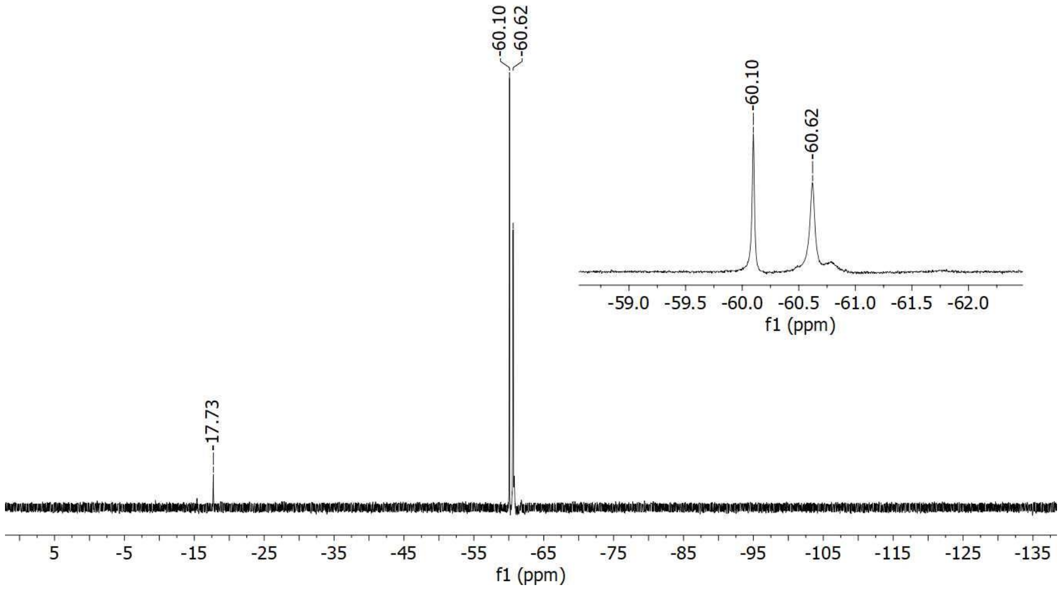

Figure S55. ^{19}F NMR spectrum of the insoluble material isolated from the reaction of complex **1** with HBF_4•Et_2O (1:1) (*top*) or **1** with AcOH (1:25) (*bottom*) in MeCN. Spectrum recorded in DMSO-d_6 at RT (δ vs. $CFCl_3$). *Insets*: Zoom-in of -60 ppm region.

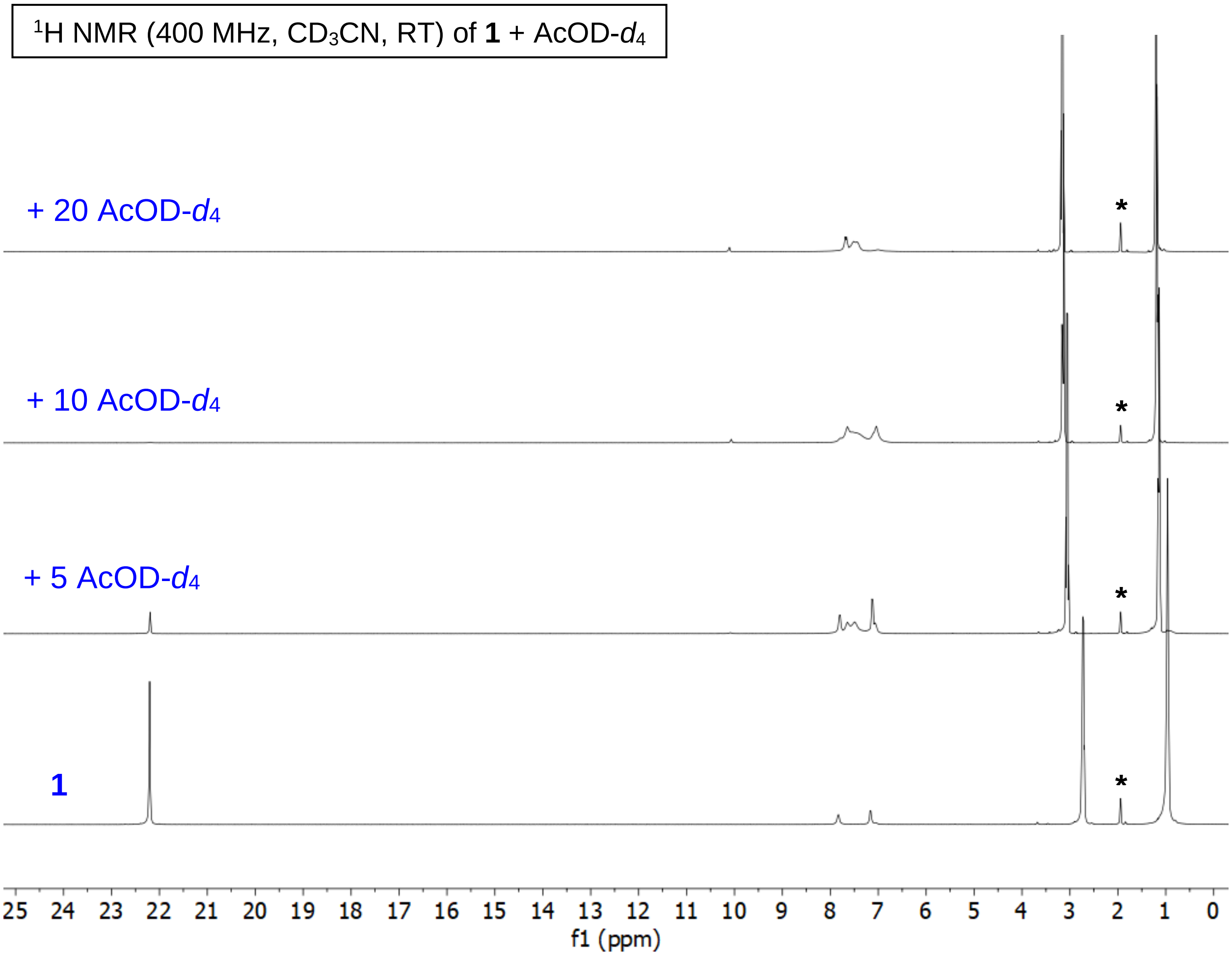

Figure S56. ^1H NMR monitor of the addition of AcOD-d_4 to complex **1** in CD_3CN at RT (δ vs. protio signal (*) at 1.94 ppm). Zoom-in of aromatic region shown in Figure S57.

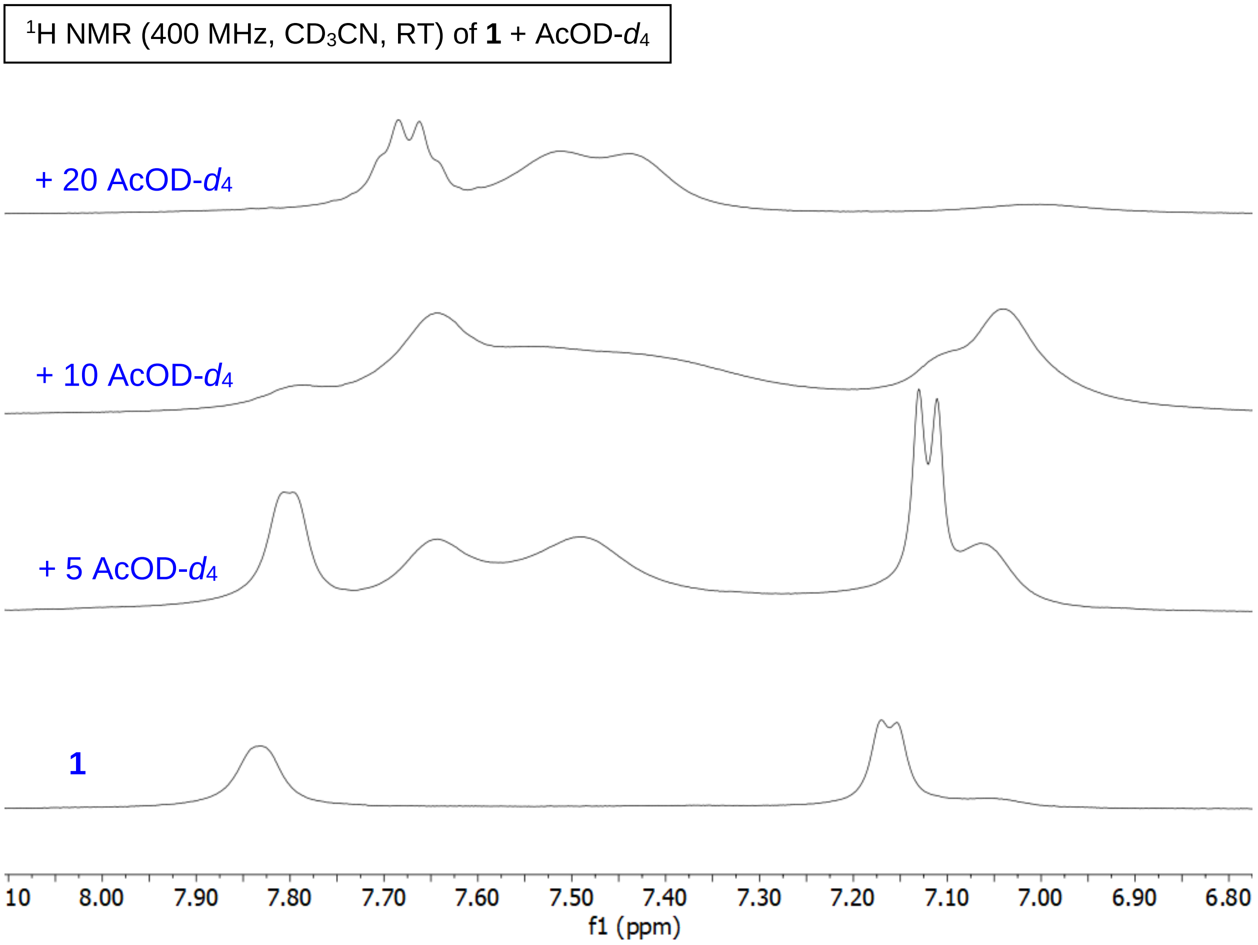

Figure S57. Zoom-in of the aromatic region of the ^{1}H NMR monitor of the addition of AcOD-d_4 to complex **1** in CD_3CN at RT (δ vs. protio signal (*) at 1.94 ppm). Full spectrum in Figure S56.

Table S13. Integration of peak for **1** at ~20 ppm and aromatic-H region obtained from the addition of AcOD-d_4 into CD_3CN solutions of **1** (see Figures S56-57). Integrations are relative to the -CH_2- peak at ~3 ppm from the Et_4N^+ counter ion.

Equivs AcOD-d_4	Integration (~20 ppm)	Integration (6 - 8 ppm)
0	6.08	2.52
5	3.98	7.67
10	1.09	13.99
20	0.05	16.06

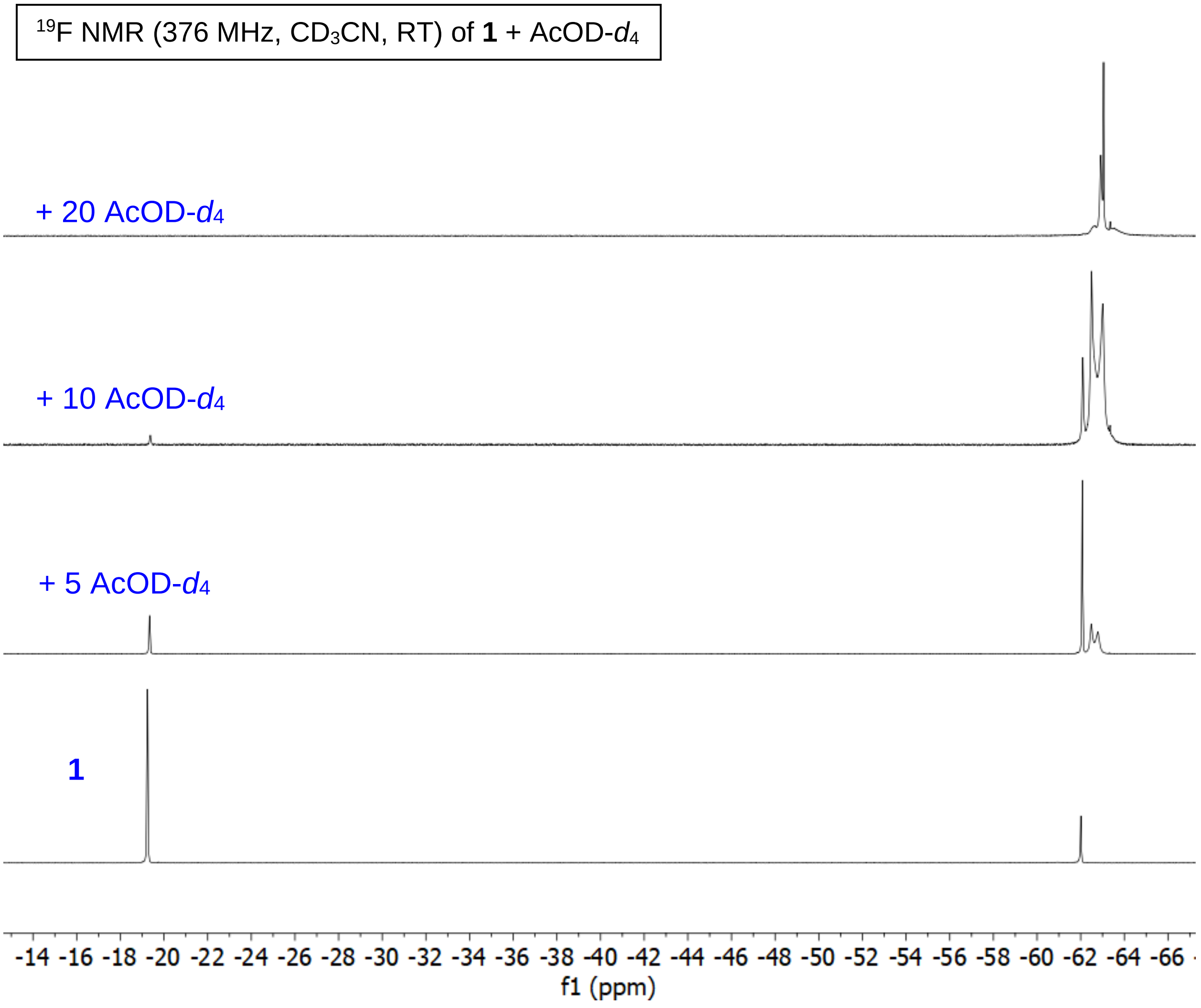

Figure S58. ^{19}F NMR monitor of the addition of AcOD-d_4 to complex **1** in CD_3CN at RT (δ vs. $CFCl_3$).

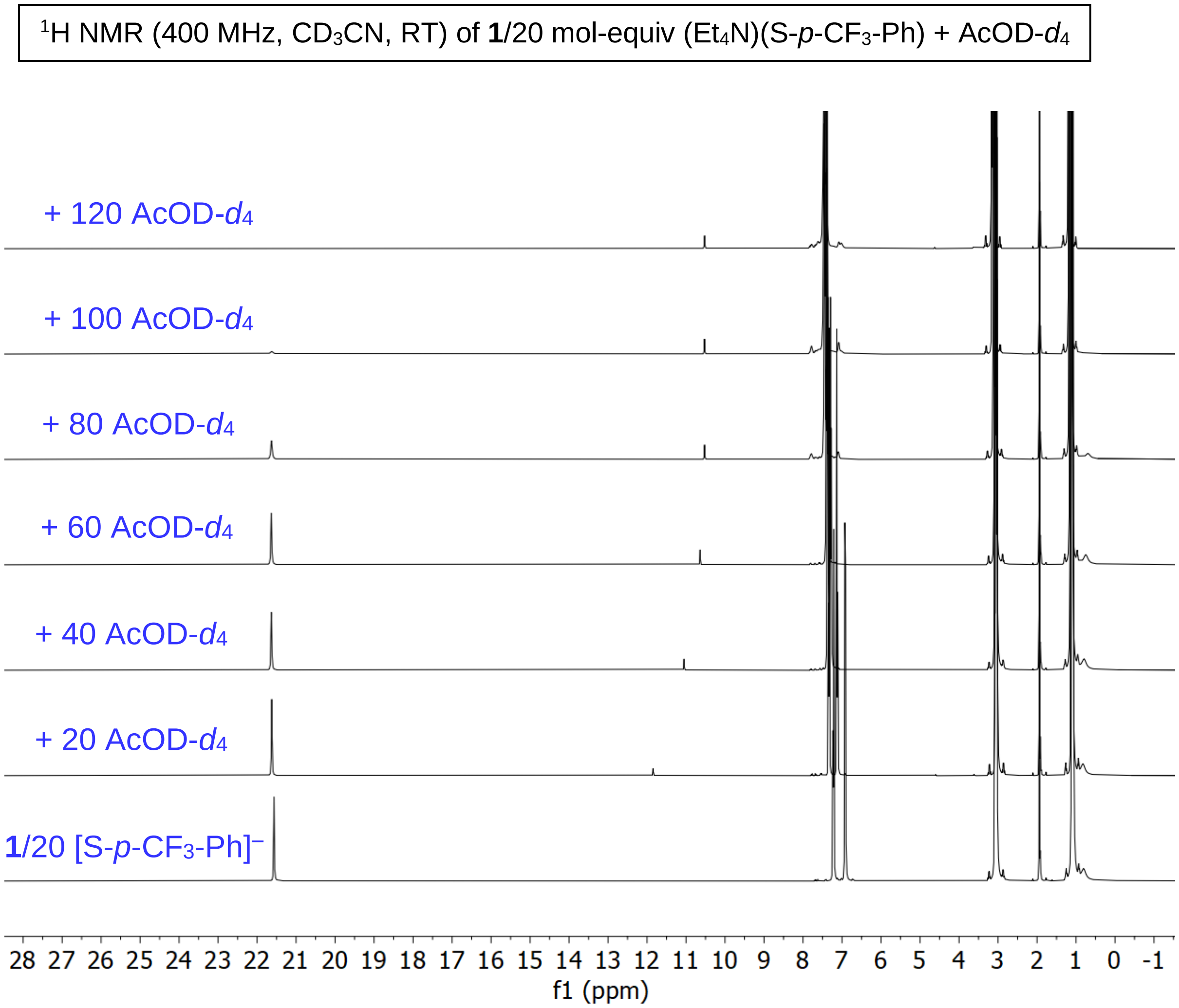

Figure S59. ^{1}H NMR monitor of the addition of AcOD-d_4 to complex **1**/20 mol-equiv (Et_4N)(S-*p*-CF_3-Ph) in CD_3CN at RT (δ vs. protio signal (*) at 1.94 ppm). *Note*: Peak at ~20 ppm, originating from **1**, remains even after addition of 20-80 equiv of AcOD-d_4. See Table S14 for quantification. Zoom-in of aromatic region shown in Figure S60.

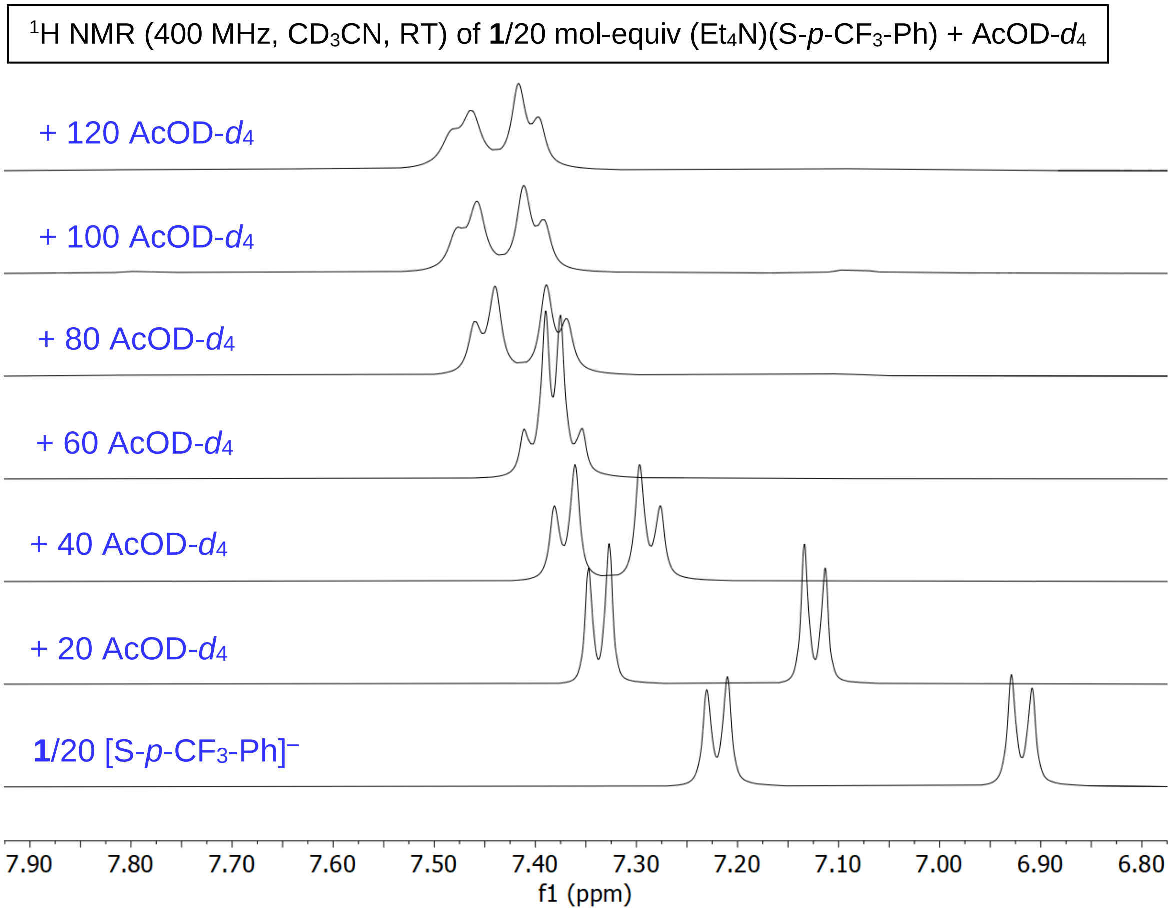

Figure S60. Zoom-in of the aromatic region of the ^{1}H NMR monitor of the addition of AcOD-d_4 to complex **1**/20 mol-equiv (Et_4N)(S-*p*-CF_3-Ph) in CD_3CN at RT. See Table S14 for quantification. Full spectrum in Figure S59.

Table S14. Integration of peak for **1** at ~20 ppm and aromatic-H region obtained from the addition of AcOD-d_4 into CD_3CN solutions of **1**/20 mol-equiv (Et_4N)(S-*p*-CF_3-Ph) (see Figures S59-S60). Integrations are relative to the -CH_2- peak at ~3 ppm from the Et_4N^+ counter ion.

Equivs AcOD-d_4	Integration (~20 ppm)	Integration (6-8 ppm)
0	7.59	69.46
20	7.57	67.09
40	7.56	71.92
60	7.18	73.24
80	4.62	75.31
100	1.32	78.64
120	0.19	80.36

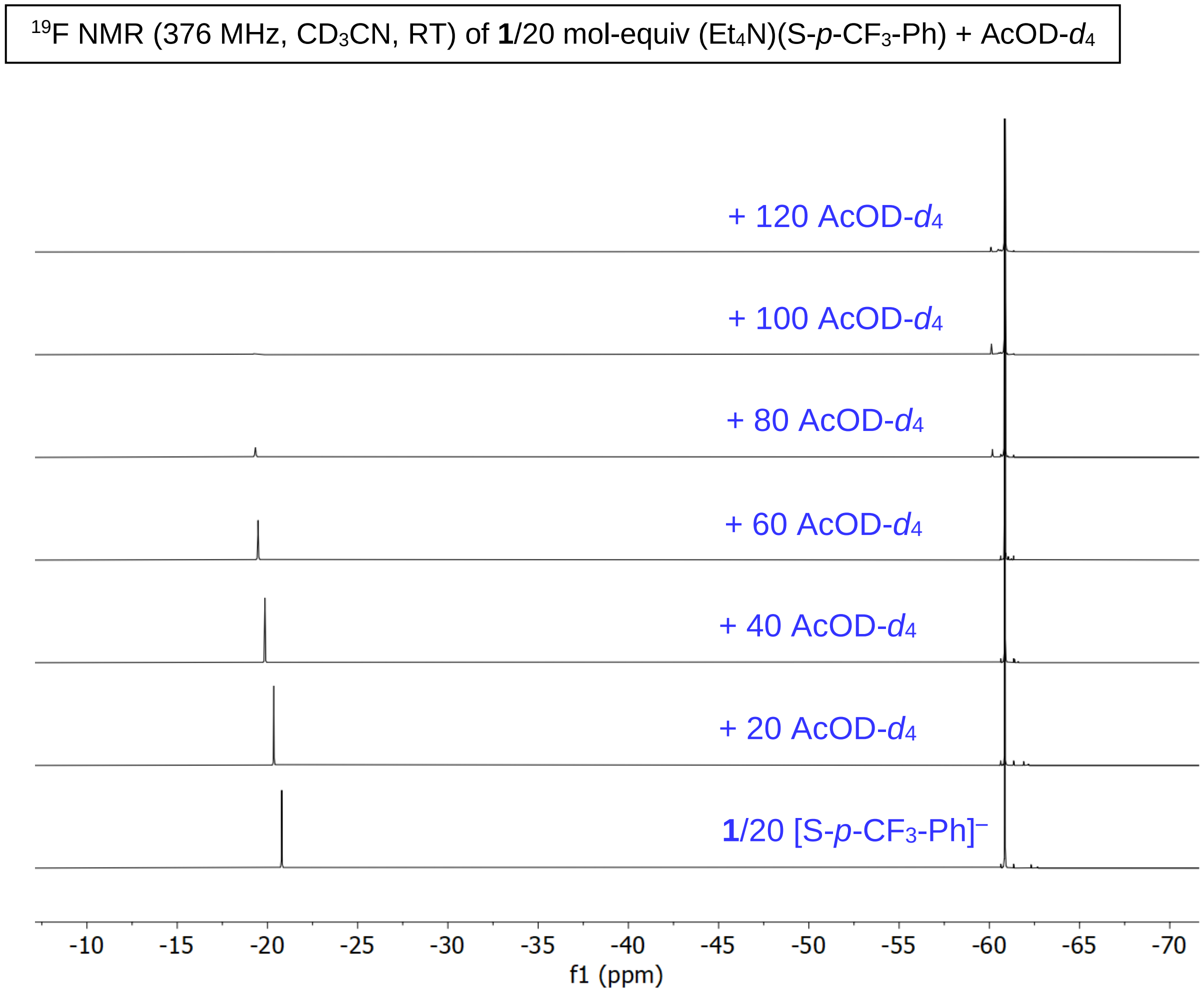

Figure S61. ^{19}F NMR monitor of the addition of AcOD-d_4 to complex **1**/20 mol-equiv (Et_4N)(S-*p*-CF_3-Ph) in CD_3CN at RT (δ vs. $CFCl_3$). *Note*: Peak at -20 ppm, originating from **1**, remains even after addition of 20-80 equiv of AcOD-d_4.

7. Electrochemical Characterization:

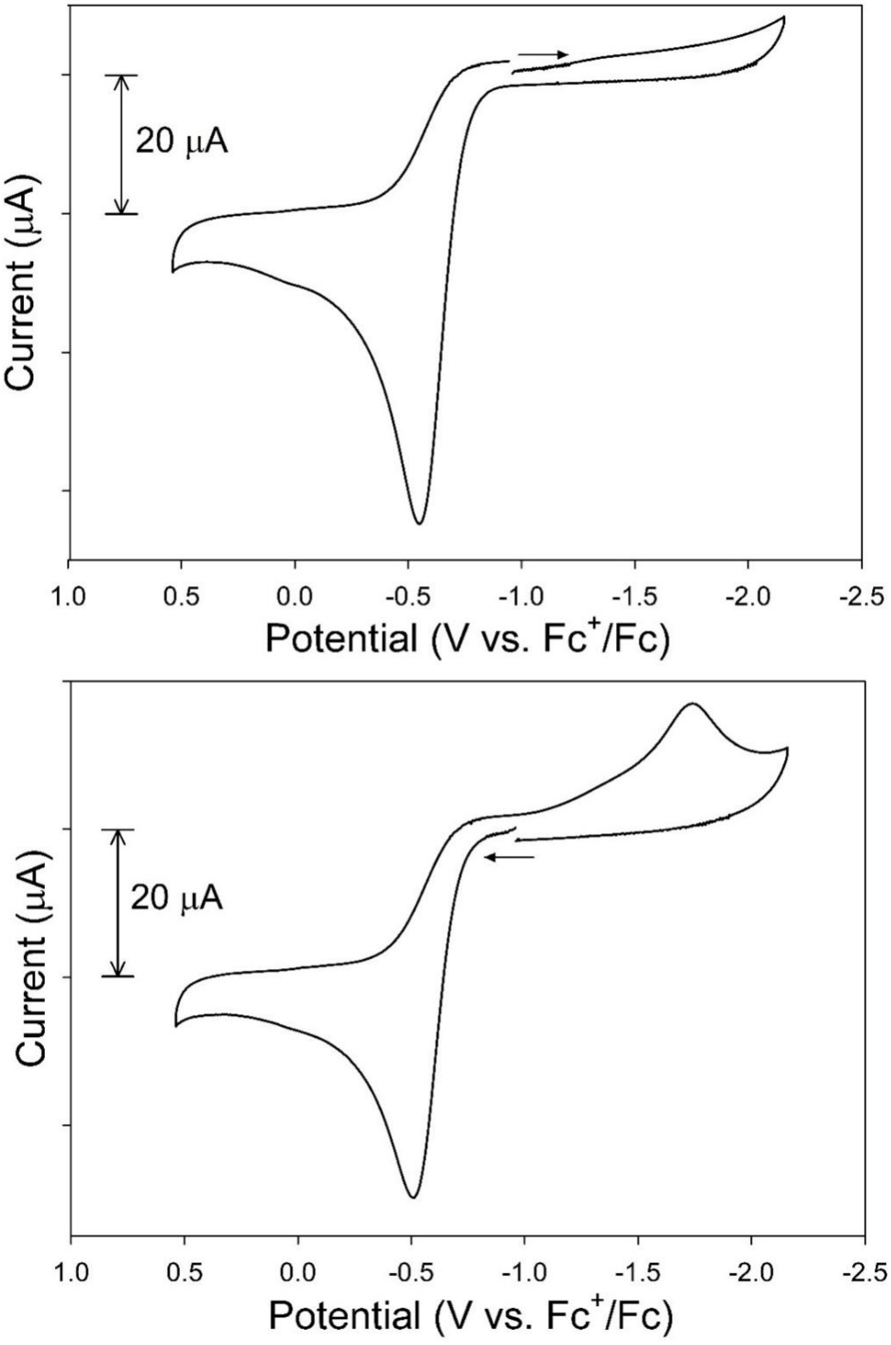

Figure S62. CV of $(Et_4N)(S\text{-}p\text{-}CF_3\text{-}Ph)$ in MeCN at RT (0.25 M $^{n}Bu_4NPF_6$ supporting electrolyte, GC working electrode, 100 mV/s scan rate). Arrow shows direction of scan.

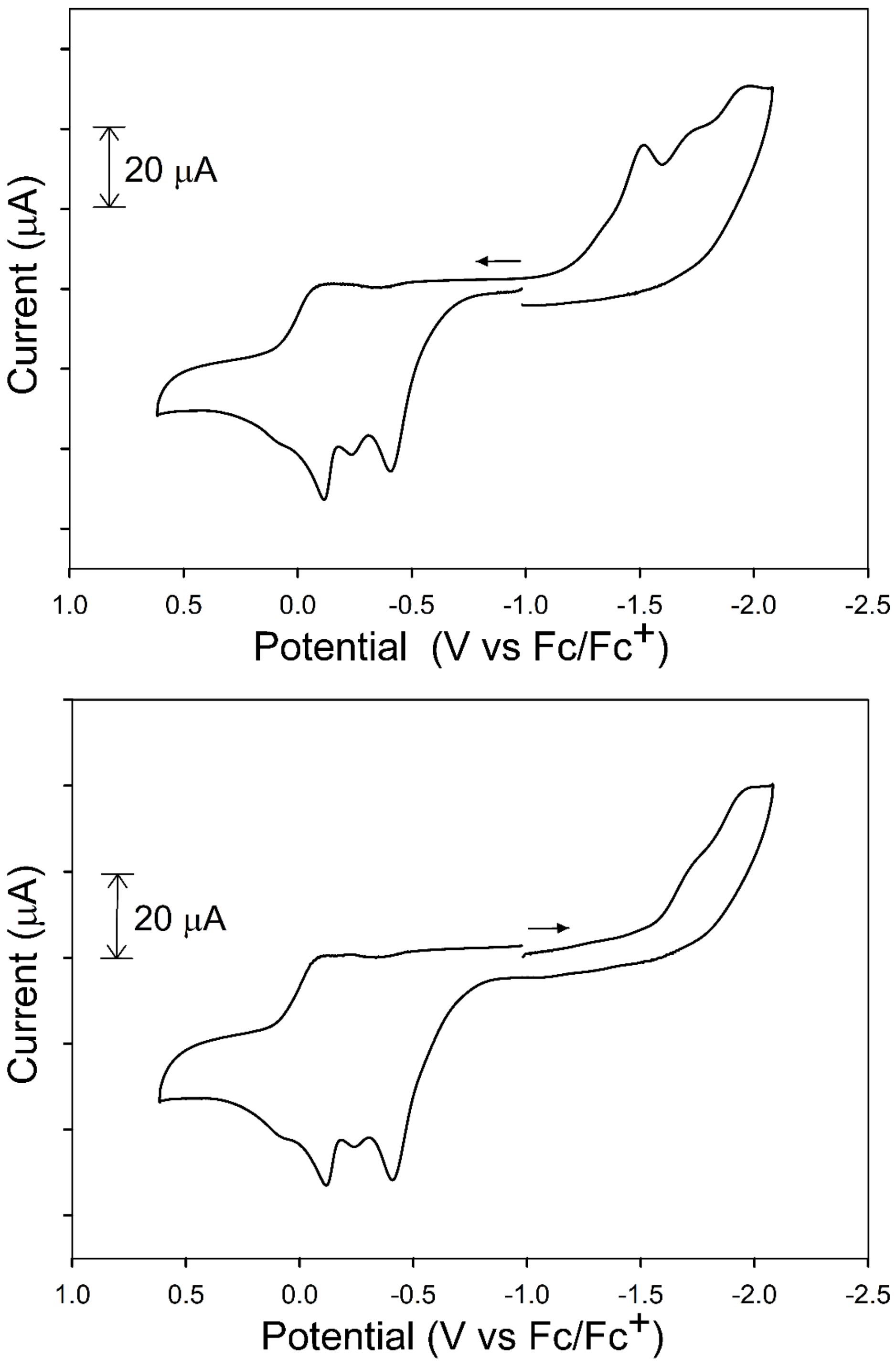

Figure S63. CV of $(Et_4N)_2[Ni(S\text{-}p\text{-}CF_3\text{-}Ph)_4]$ (**1**) in MeCN at RT (0.25 M $^{n}Bu_4NPF_6$ supporting electrolyte, GC working electrode, 100 mV/s scan rate). Arrow shows direction of scan.

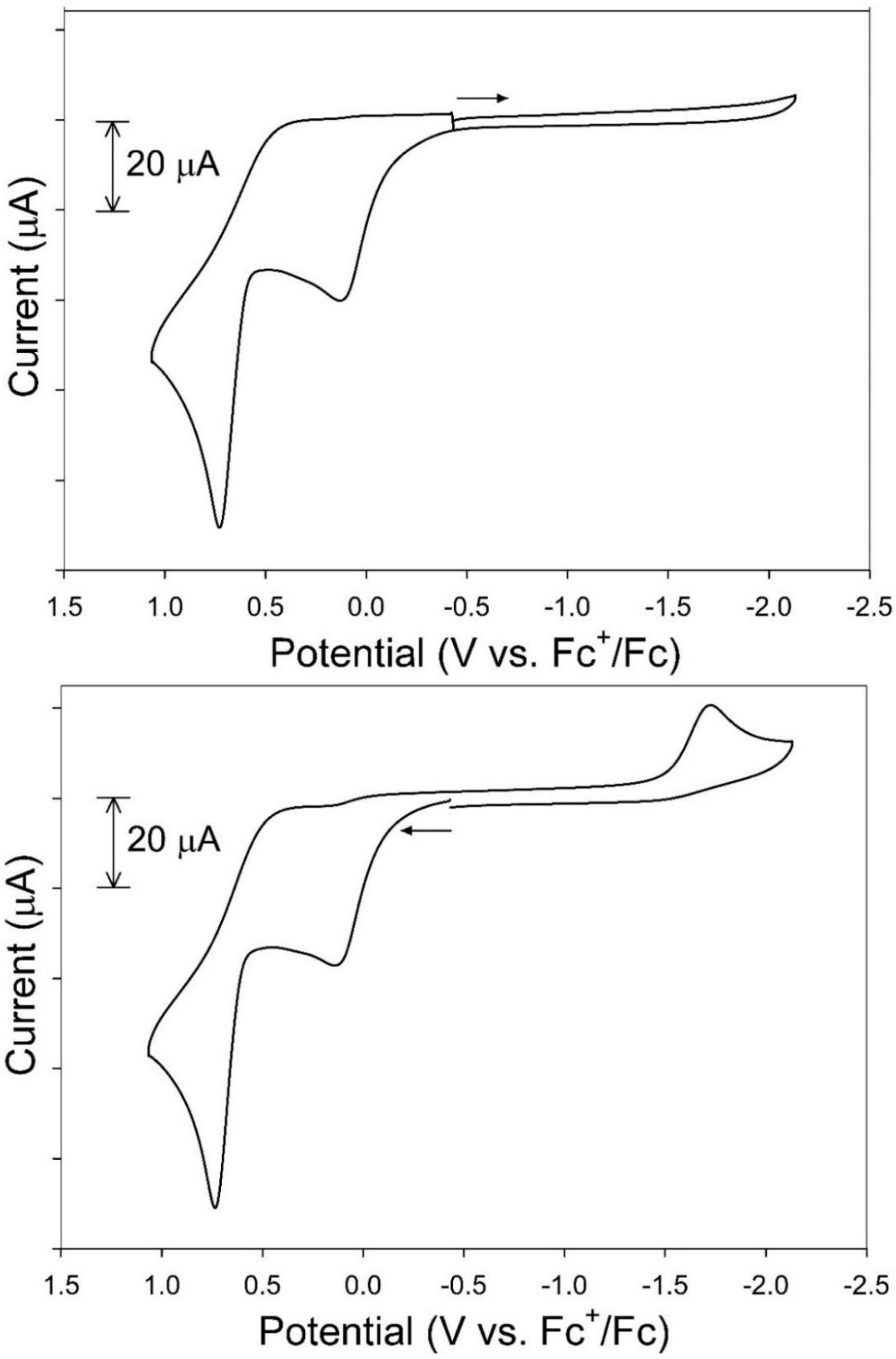

Figure S64. CV of $(Et_4N)_2[Zn(S\text{-}p\text{-}CF_3\text{-}Ph)_4]$ (**2**) in MeCN at RT (0.25 M $^{n}Bu_4NPF_6$ supporting electrolyte, GC working electrode, 100 mV/s scan rate). Arrow shows direction of scan.

8. Electrocatalysis:

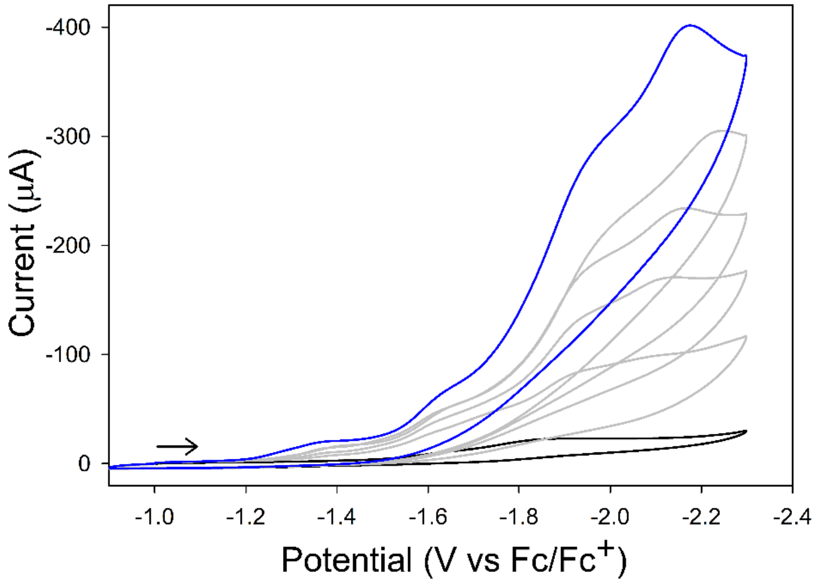

Figure S65. CV of complex **1** (1.87 mM; black trace) and after the addition of AcOH in 4 mol-equiv increments (gray traces) up to a final of 20 mol-equiv (blue trace) in MeCN at RT. Conditions: 0.25 M $^{n}Bu_4NPF_6$ supporting electrolyte, GC working electrode, 100 mV/s scan rate. Arrow displays the scan direction.

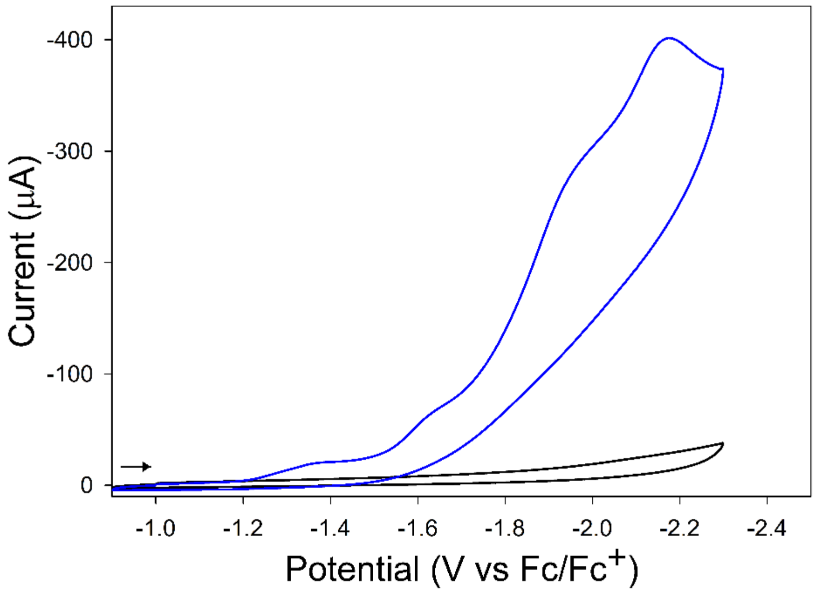

Figure S66. CV of complex **1** (1.87 mM) with 20 mol-equiv of AcOH (blue trace) in MeCN at RT, and subsequent CV after the rinse test of the working electrode and immersion into an electrochemical cell containing electrolyte/solvent and AcOH at identical concentrations (black trace). Conditions: 0.25 M $^{n}Bu_4NPF_6$ supporting electrolyte, GC working electrode, 100 mV/s scan rate. Arrow displays the scan direction.

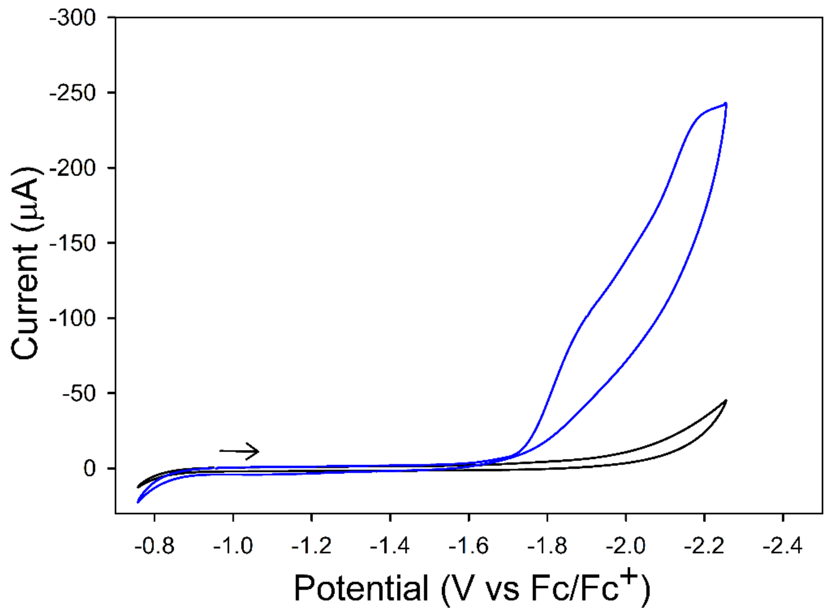

Figure S67. CV of complex **1** (1.89 mM) containing 20 mol-equiv of $(Et_4N)(S\text{-}p\text{-}CF_3\text{-}Ph)$ and 20 mol-equiv of AcOH (blue trace) in MeCN at RT. Subsequent CV after rinse test of the working electrode and immersion into an electrochemical cell containing electrolyte/solvent and 20 mol-equiv each of $(Et_4N)(S\text{-}p\text{-}CF_3\text{-}Ph)$ and AcOH (black trace). Conditions: 0.25 M $^{n}Bu_4NPF_6$ supporting electrolyte, GC working electrode, 100 mV/s scan rate. Arrow displays the scan direction.

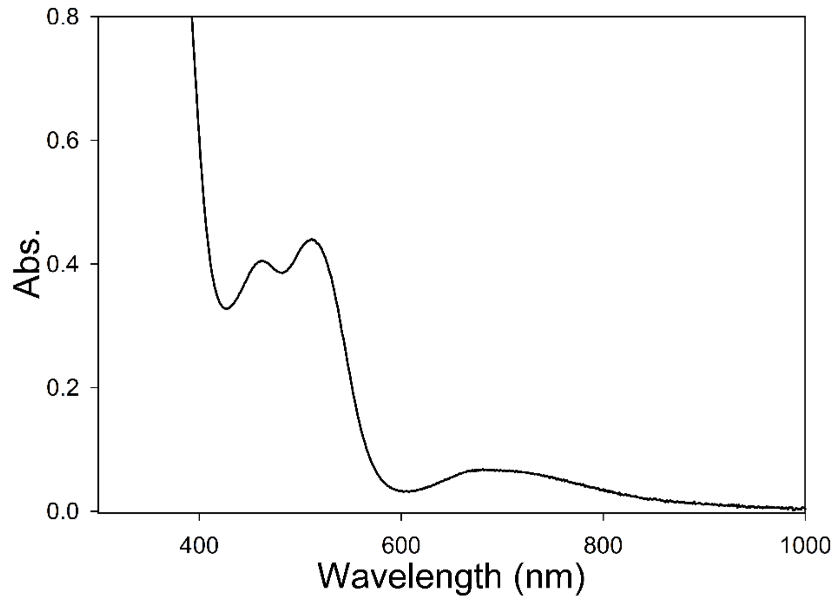

Figure S68. UV-vis spectrum of a 150 μL aliquot taken from the electrochemical cell containing **1**/20 mol-equiv $(Et_4N)(S\text{-}p\text{-}CF_3\text{-}Ph)$ and 20 mol-equiv AcOH in MeCN at RT.

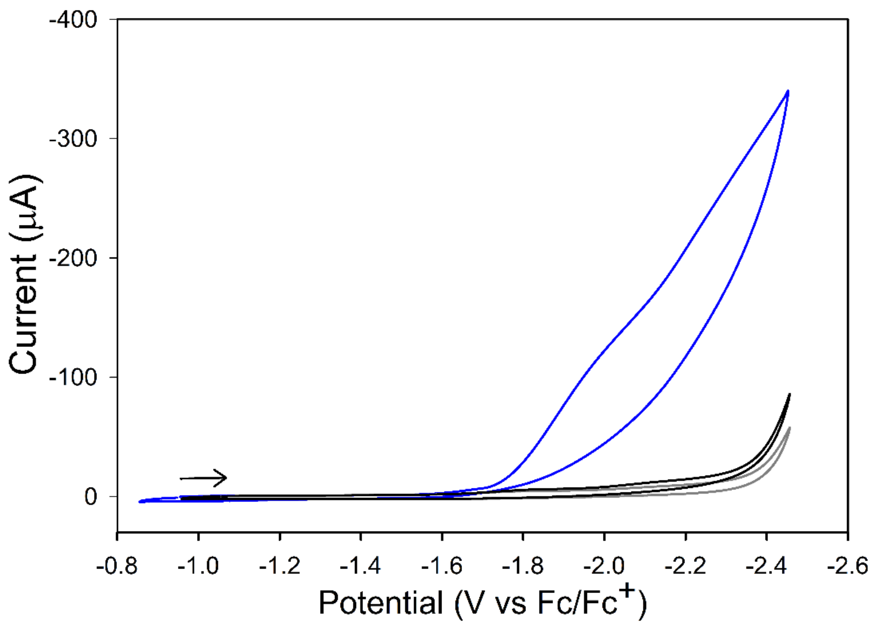

Figure S69. CV of compounds in the presence of 20 mol-equiv AcOH in MeCN at RT. Black trace = 38 mM solution of (Et_4N)(S-*p*-CF_3-Ph). Grey trace = 1.9 mM solution of Zn complex **2** with 20 mol-equiv of (Et_4N)(S-*p*-CF_3-Ph). Blue trace = 1.9 mM solution of Ni complex **1** with 20 mol-equiv of (Et_4N)(S-*p*-CF_3-Ph). Conditions: 0.25 M $^{n}Bu_4NPF_6$ supporting electrolyte, GC working electrode, 100 mV/s scan rate. Arrow displays the scan direction.

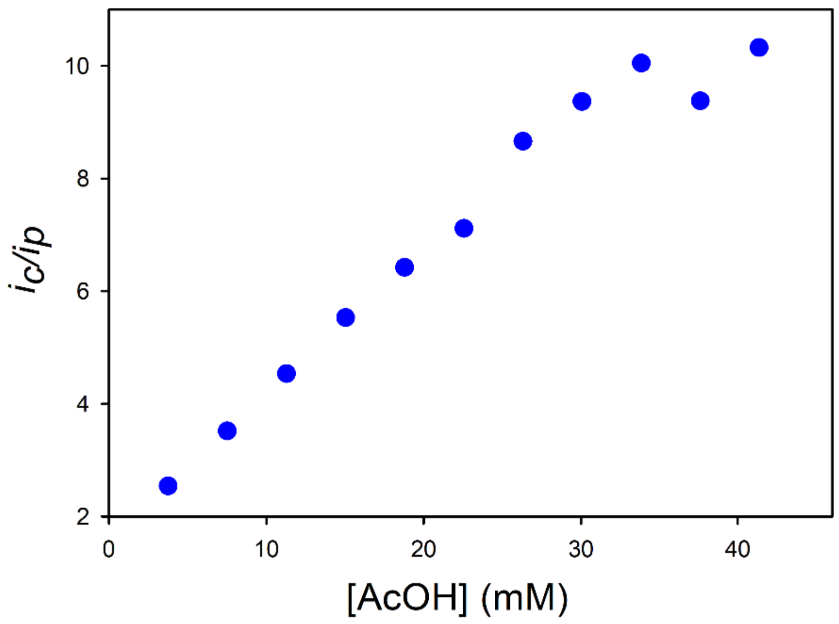

Figure S70. Normalized catalytic current response (i_c/i_p) versus [AcOH] taken from CV's of acid titrations of **1**/20 mol-equiv ($(Et_4N)(S$-*p*-CF_3-Ph) in MeCN (see Figure 9 in the main paper).

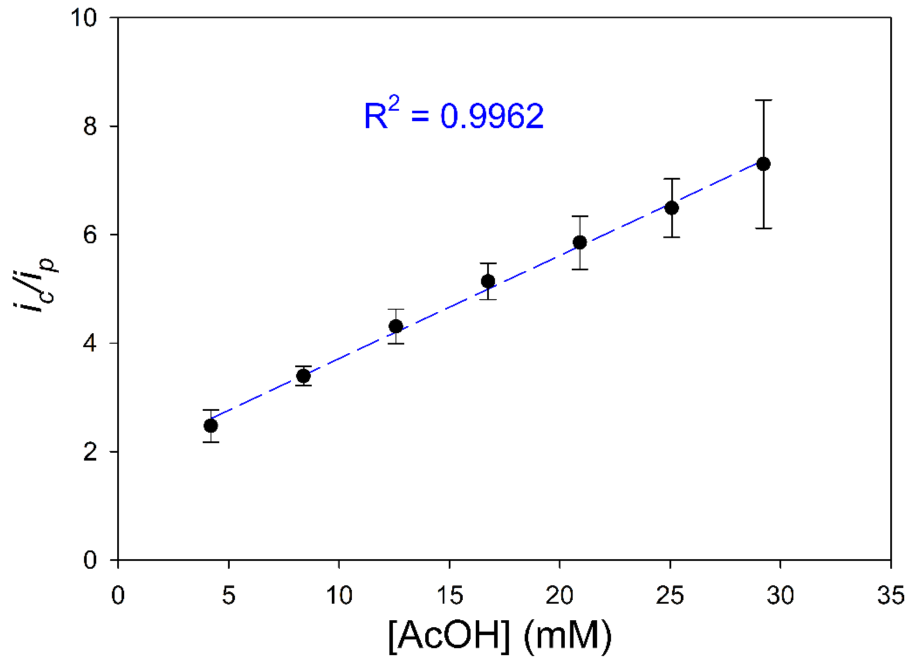

Figure S71. Ratio of catalytic current response (i_c/i_p) versus [AcOH] taken from CV's of AcOH titration to **1**/20 mol-equiv ((Et_4N))(S-*p*-CF_3-Ph) prior to the acid-independent region, (18 mol-equiv AcOH, ~36 mM). Each point represents the average of three trials with error bars showing standard deviations.

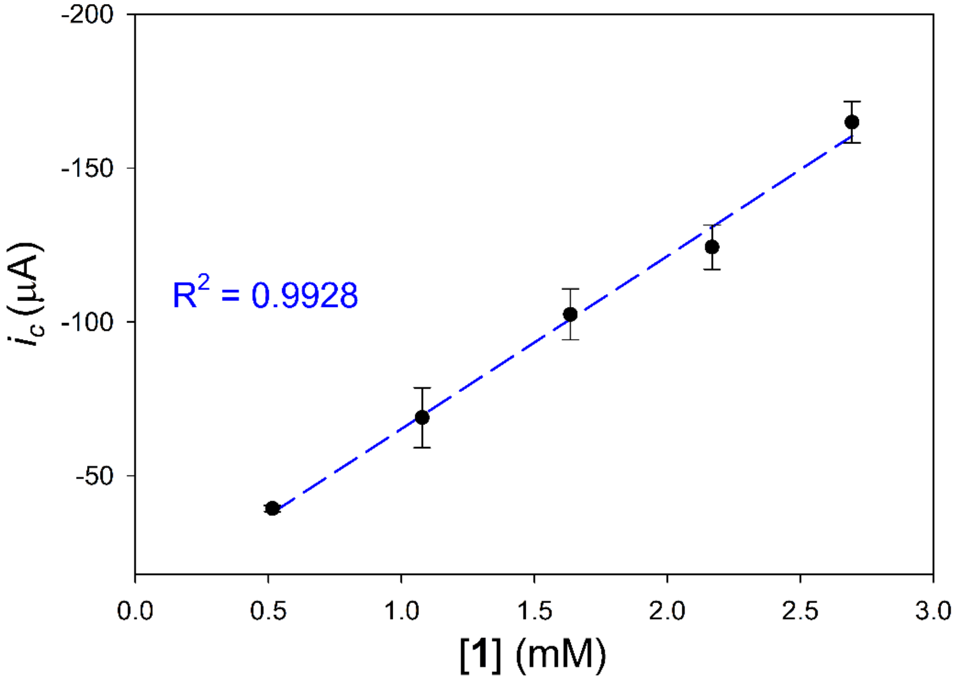

Figure S72. Catalytic current response (i_c) versus [**1**] in the presence of 20 mol-equiv of (Et_4N)(S-*p*-CF_3-Ph) and 5 mol-equiv AcOH. Each point represents the average of three trials with error bars showing standard deviations.

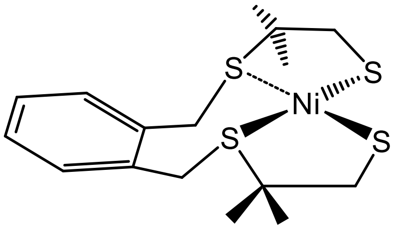

[Ni(xsbms)]

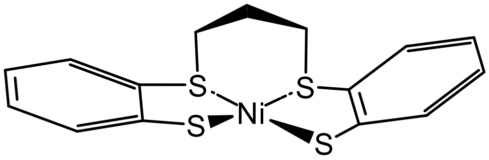

[Ni('S_4-C_3')]

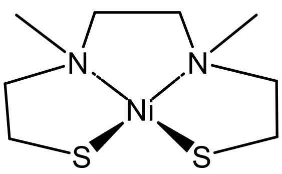

[NiN_2S_2]

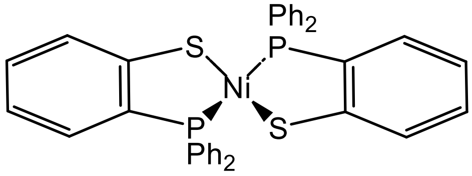

[$Ni(PS)_2$]

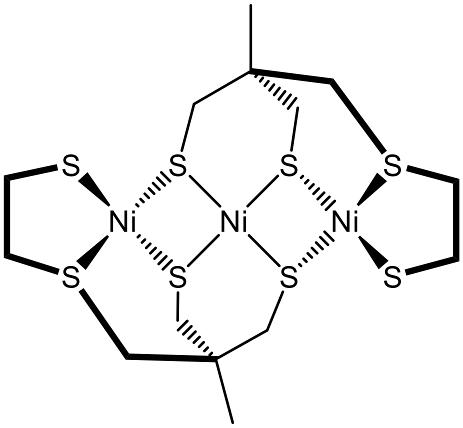

[Ni_3S_6]

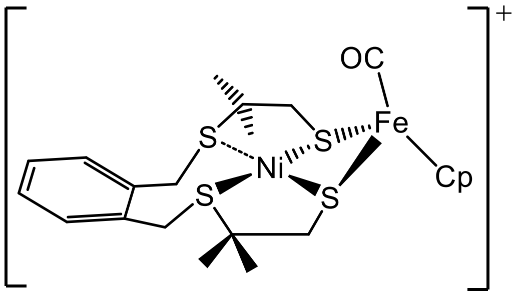

[Ni(xsbms)FeCpCO]$^+$

Chart S1. Structures of select Ni-thiolate HER electrocatalyst shown in Table 3 of the main paper.

9. DFT-Computed Mechanism:

DFT-computed redox potentials. DFT methods were employed to provide an estimate of the reduction potentials for proposed intermediates in the HER catalytic cycle of **1** (Figure S73, Scheme S3). Further details are described in previously published work where DFT has been used to compute the one-electron potentials of several transition metal complexes.[1-5] The reduction potential ($E°$) is related to the Gibbs free energy difference in solution (ΔG_{soln}) given by eq. ix:

$$E° = -\frac{\Delta G_{soln}}{nF} \qquad \text{(ix)}$$

Where F is Faraday's constant (23.06 kcal mol^{-1} V^{-1}) and n is the number of electrons transferred ($n = 1$). ΔG_{soln} can be determined from eq. x using the thermodynamic cycle depicted in Scheme S1 for the Ni(III/II) couple for the conversion of $\mathbf{^{4}1^{R}NiH,SH}$ to $\mathbf{^{3}1^{R2}NiH,SH}$:

$$\Delta G_{soln} = \Delta G_{gas} + \Delta G(^{4}1^{R2}NiH,SH)_{solv} - \Delta G(^{3}1^{R2}NiH,SH)_{solv} \qquad \text{(x)}$$

where ΔG_{gas} is the Gibbs free energy difference in the gas phase, and the $\Delta G(^{4}\mathbf{1}^{R}\mathbf{NiH,SH}$ $\mathbf{1}^{R}\mathbf{NiH,SH})_{solv}$ and $\Delta G(^{4}\mathbf{1}^{R2}\mathbf{NiH,SH})_{solv}$ are the solvation energies in acetonitrile. The difference of the gas-phase Gibbs free energies of $\mathbf{^{4}1^{R}NiH,SH}$ and $\mathbf{^{3}1^{R2}NiH,SH}$ yields $\Delta G(red)_{gas}$, and the difference of the Gibbs free energies of $\mathbf{^{4}1^{R}NiH,SH}$ and $\mathbf{^{3}1^{R2}NiH,SH}$ using CPCM(MeCN) yields $\Delta G(red)_{soln}$. Solvation energies (ΔG_{solv}) were obtained from the difference of single point energies, from single point energy calculations, of the intermediate in the absence and presence of solvent. The absolute potentials for the Fc^{+}/Fc couple was calculated at the same level of theory under an identical procedure to properly reference the redox couples. The results of the redox calculations for $\mathbf{^{4}1^{R}NiH,SH}$/$\mathbf{^{3}1^{R2}NiH,SH}$ are presented in Table S15. *Note*: calculation of the potentials for other proposed intermediates were performed in an identical manner.

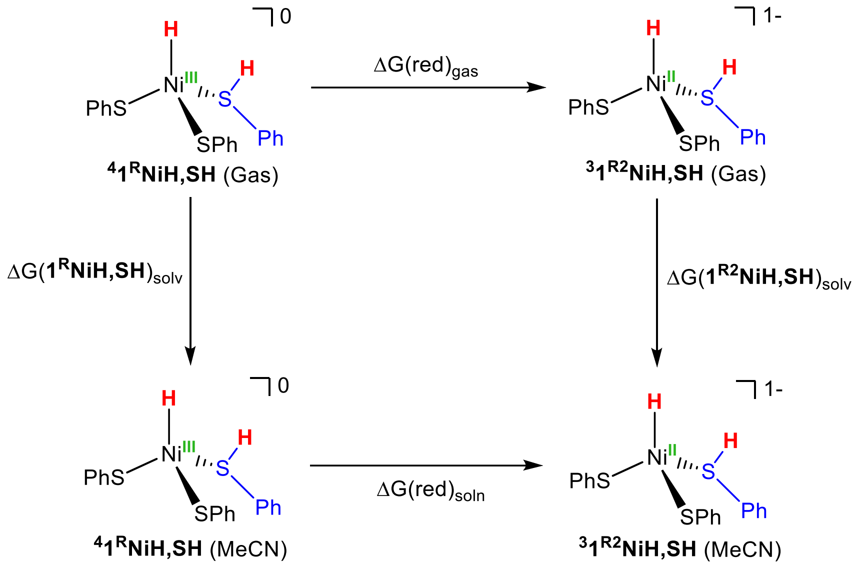

Scheme S1. Thermodynamic cycle used to calculate $E°$ for the ^{4}**1**^{R}**NiH,SH**/^{3}**1**^{R2}**NiH,SH** conversion.

Table S15. Redox Potential calculations for ^{4}**1**^{R}**NiH,SH**/^{3}**1**^{R2}**NiH,SH** and Fc^{+}/Fc in acetonitrile.

	**Fc^{+}**	**Fc**	**^{4}1^{R}NiH,SH**	**^{3}1^{R2}NiH,SH**
Gibbs free energy (G)	-1649.83178	-1650.08528	-4408.94134	-4409.07132
Δ*G* (gas)	-0.25350		-0.12997	
Single Point Energy (gas)	-1650.00833	-1650.26308	-4409.18475	-4409.31348
Single Point Energy (soln.)	-1650.08345	-1650.26876	-4409.20196	-4409.37955
Solvation Energy	-0.07512	-0.00567	-0.01721	-0.06606
ΔΔ*G* (solv.)[b]	0.06944		-0.04885	
Δ*G* (soln.)[c]	-0.18406		-0.17883	
Δ*G* (soln.) (kcal/mol)	-115.501		-112.217	
E° (V)[d]	5.009		4.866	
E° (V) (vs. Fc^{+}/Fc)	**0.000**		**-0.142**	

[a] Energies are in hartrees (E_h) unless noted otherwise. [b] Difference in solvation energies. [c] Calculated according to eq. x. [d] Calculated according to eq. ix.

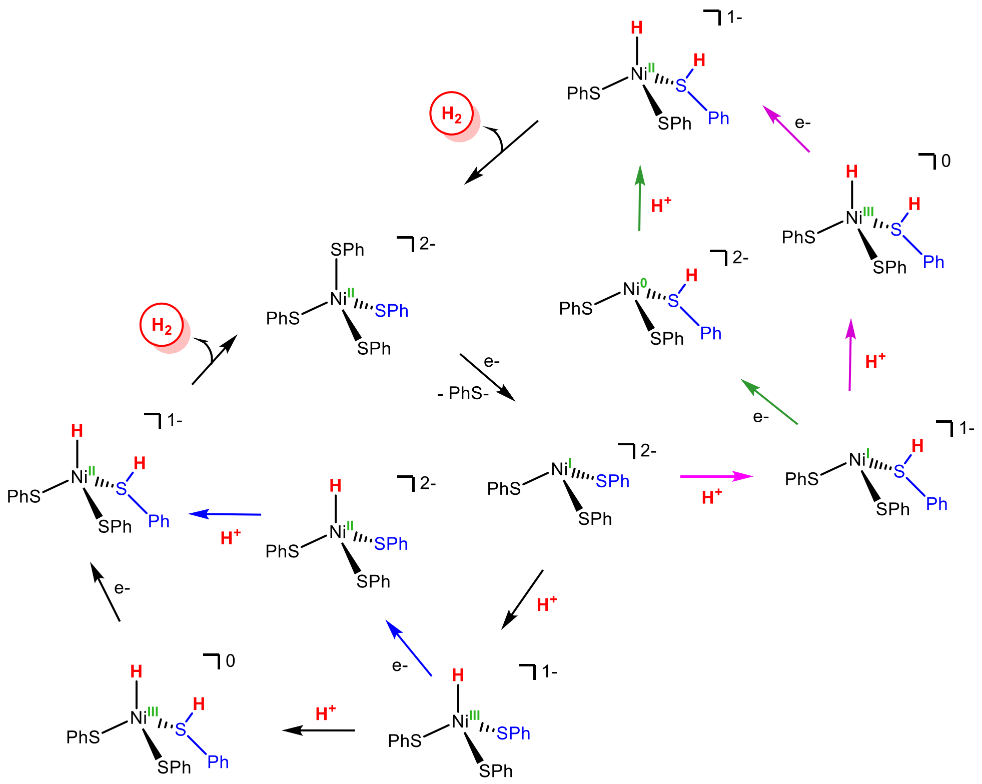

Scheme S2. All probable mechanistic paths for H_2 evolution from **1** starting from reduction of 1 to generate the 3C Ni^{1+} complex dianion $[Ni(SPh)_3]^{2-}$, i.e., ^{2}**1**^{R}. *Note*: for simplicity, the general abbreviation PhS^- was used in this scheme to represent the *p*-CF_3 thiolate ligand of **1**.

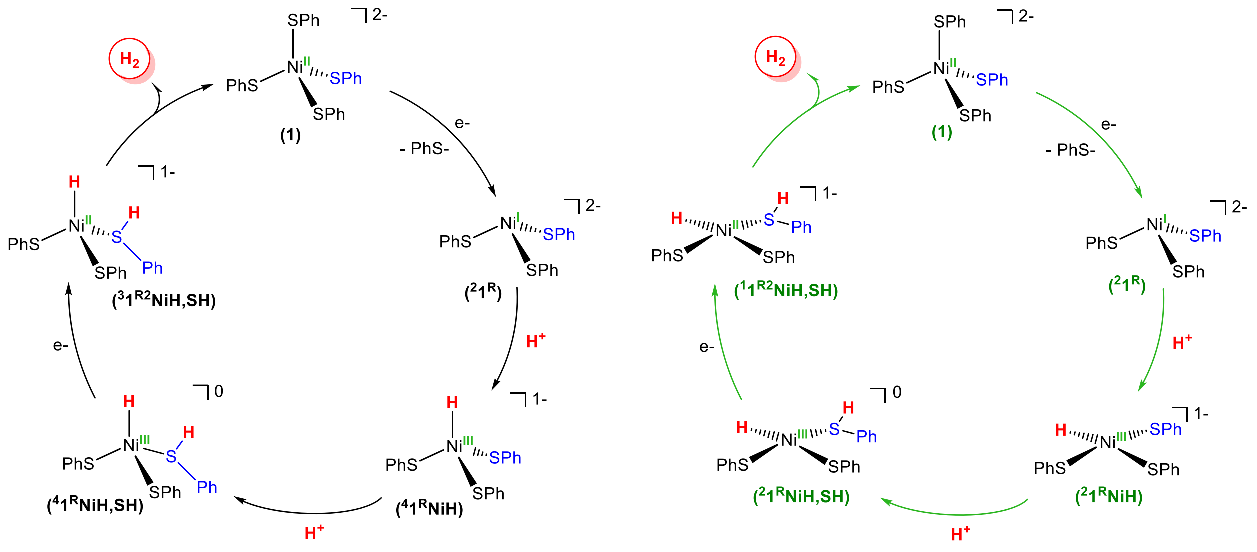

Scheme S3. Proposed ECCE mechanism of HER by **1** through tetrahedral (*left*) and square-planar intermediates (*right*). *Note*: for simplicity, the general abbreviation PhS^{-} was used in this scheme to represent the *p*-CF_3 thiolate ligand of **1**.

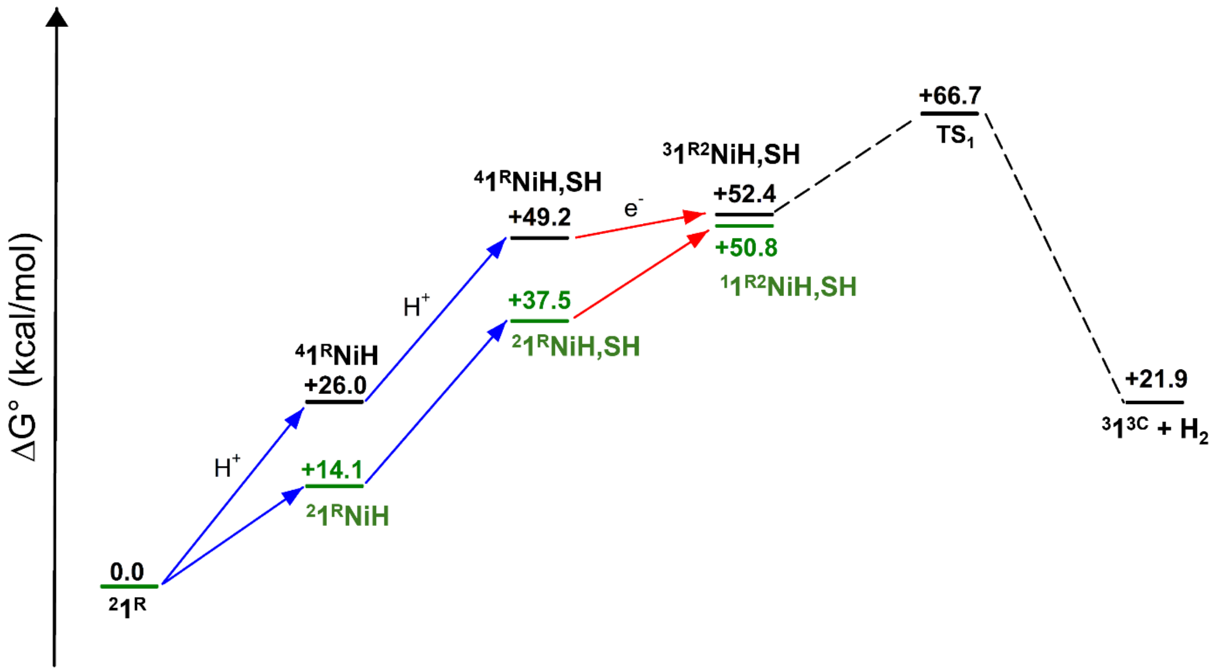

Figure S73. DFT-computed energy pathways for ECCE mechanism of HER by **1** through tetrahedral intermediates (*black*) and square-planar intermediates (*green*).

Table S16. Charge and Gibbs free energies from frequency calculations (B3LYP/def2-TZVPP, solution phase = MeCN) of intermediates in proposed mechanisms in Scheme S3, Figure S73.

Intermediate	Charge	Gibbs Free Energy (Eh)
$^{2}\mathbf{1}^{\mathbf{R}}$	2-	-4408.12685
$^{4}\mathbf{1}^{\mathbf{R}}\mathbf{NiH}^{a}$	1-	-4408.53953
$^{3}\mathbf{1}^{\mathbf{R2}}\mathbf{NiH}^{a}$	2-	-4408.69344
$^{4}\mathbf{1}^{\mathbf{R}}\mathbf{NiH,SH}^{a}$	0	-4408.95678
$^{3}\mathbf{1}^{\mathbf{R2}}\mathbf{NiH,SH}^{a}$	1-	-4409.13827
Et_3N	0	-291.91249
Et_3NH	1	-292.36662
$^{2}\mathbf{1}^{\mathbf{R}}\mathbf{NiH}^{b}$	1-	-4408.55845
$^{1}\mathbf{1}^{\mathbf{R2}}\mathbf{NiH}^{b}$	2-	-4408.69246
$^{2}\mathbf{1}^{\mathbf{R}}\mathbf{NiH,SH}^{b}$	0	-4408.97543
$^{1}\mathbf{1}^{\mathbf{R2}}\mathbf{NiH,SH}^{b}$	1-	-4409.14171
H_2	0	-1.17575
$^{3}\mathbf{1}^{\mathbf{3C}c}$	1-	-4408.00477

[a] Tetrahedral geometry. [b] Square planar geometry. [c] $[Ni(S\text{-}p\text{-}CF_3\text{-}Ph)_3]^-$

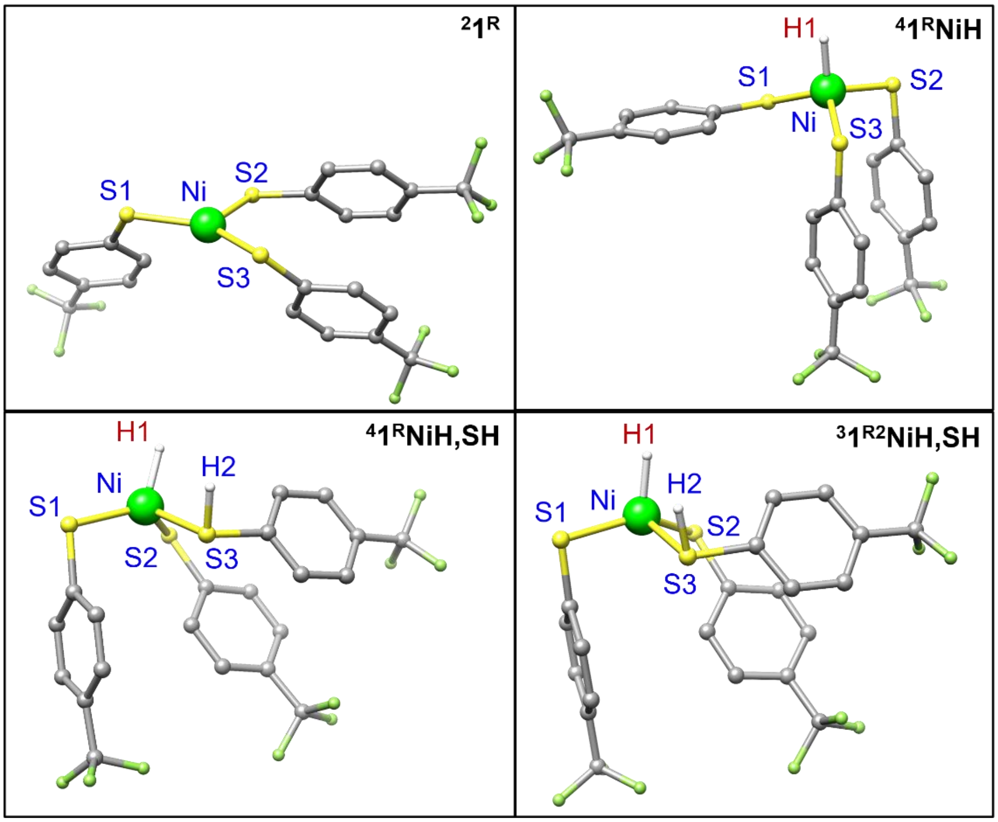

Figure S74. DFT-optimized structures (B3LYP/def2-TZVPP, gas-phase) for proposed *tetrahedral* intermediates for the ECCE mechanism of H_2 evolution from **1** (see Scheme S3, Figure S73).

Table S17. Select metric properties of the DFT-optimized structures of proposed *tetrahedral* intermediates for the ECCE mechanism of H_2 evolution from **1** (see Figure S74 for structures).

	^{2}**1**^{R}	^{4}**1**^{R}**NiH**	^{4}**1**^{R}**NiH,SH**	^{3}**1**^{R2}**NiH,SH**
Ni-S_1 (Å)	2.278	2.247	2.205	2.315
Ni-S_2 (Å)	2.311	2.232	2.193	2.311
Ni-S_3 (Å)	2.300	2.229	2.396	2.470
Ni-H_1 (Å)	---	1.578	1.574	1.572
S_3-H_2 (Å)	---	---	1.349	1.346
S_1-Ni-S_2 (°)	112.4	105.3	122.4	140.9
S_1-Ni-S_3 (°)	138.3	120.5	105.0	92.4
S_2-Ni-S_3 (°)	109.2	111.3	107.8	103.1
S_1-Ni-H_1 (°)	---	111.8	115.9	103.2
S_2-Ni-H_1 (°)	---	104.9	104.4	110.1
S_3-Ni-H_1 (°)	---	102.1	98.5	96.9
τ_3	0.04	---	---	---
τ_4	---	0.90	0.86	0.77

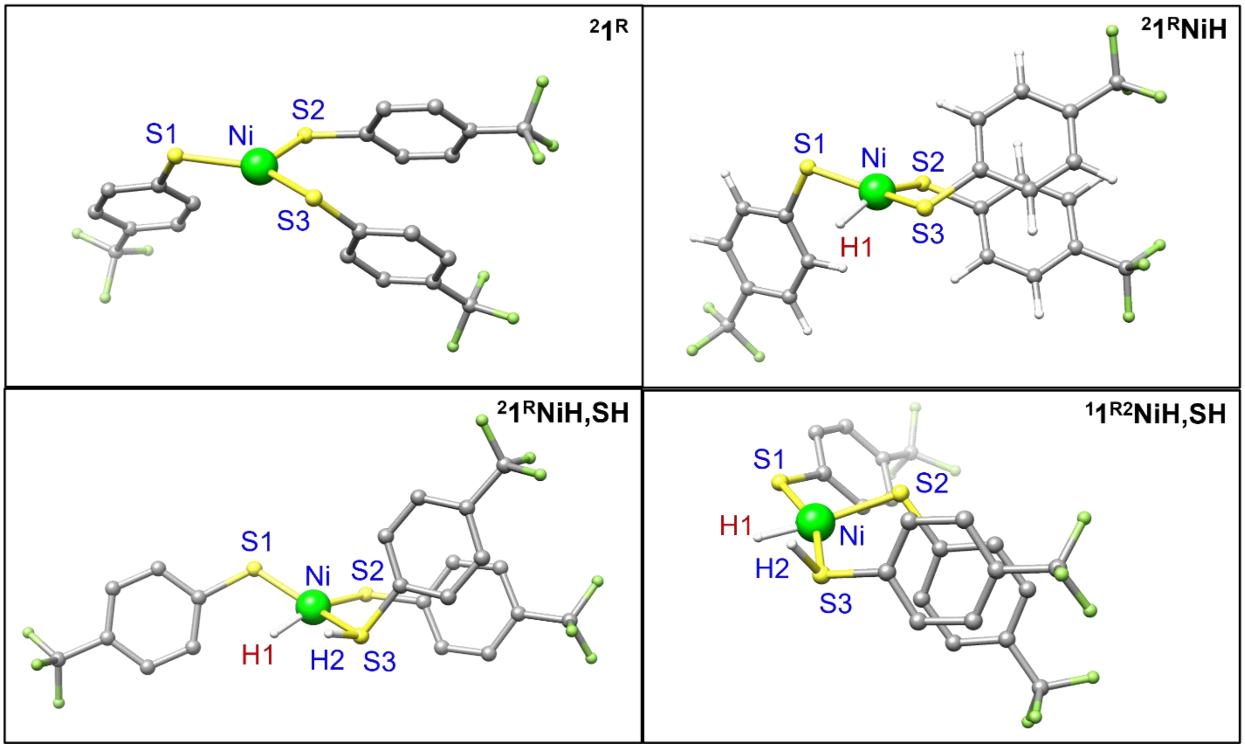

Figure S75. DFT-optimized structures (B3LYP/def2-TZVPP, gas-phase) for proposed *square-planar* intermediates for the ECCE mechanism of H_2 evolution from **1** (see Scheme S3, Figure S73).

Table S18. Select metric properties of the DFT-optimized structures of proposed *square-planar* intermediates for the ECCE mechanism of H_2 evolution from **1** (see Figure S75 for structures).

	^{2}**1**^{R}	^{2}**1**^{R}**NiH**	^{2}**1**^{R}**NiH,SH**	^{1}**1**^{R2}**NiH,SH**
Ni-S_1 (Å)	2.278	2.237	2.111	2.183
Ni-S_2 (Å)	2.311	2.219	2.194	2.272
Ni-S_3 (Å)	2.300	2.192	2.266	2.201
Ni-H_1 (Å)	---	1.458	1.457	1.484
S_3-H_2 (Å)	---	---	1.350	1.348
S_1-Ni-S_2 (°)	112.4	94.8	102.6	100.8
S_1-Ni-S_3 (°)	138.3	153.2	151.3	158.9
S_2-Ni-S_3 (°)	109.2	110.7	101.2	100.3
S_1-Ni-H_1 (°)	---	79.8	80.1	80.5
S_2-Ni-H_1 (°)	---	145.6	156.1	172.8
S_3-Ni-H_1 (°)	---	83.3	84.7	78.4
τ_3	0.04	---	---	---
τ_4	---	0.43	0.40	0.20

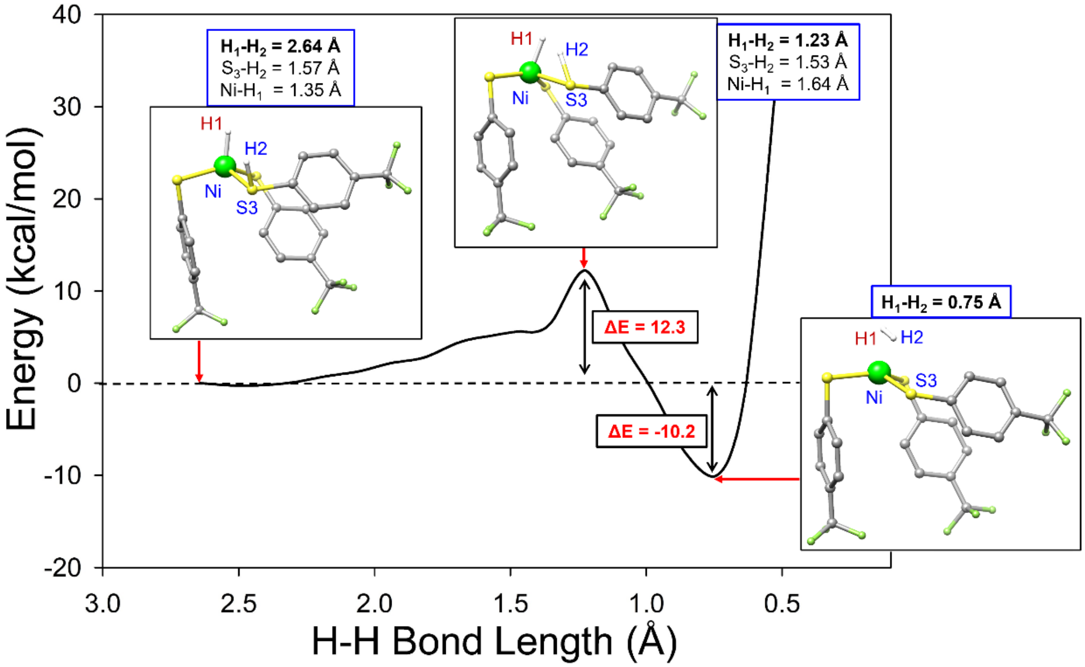

Figure S76. Relaxed surface potential energy scan of **^{3}1^{R2}NiH,SH**. H---H (2.64 to 0.40 Å), distances were scanned in 0.11 Å increments. *Insets*: optimized structures of **^{3}1^{R2}NiH,SH**, the transition state, and reaction well.

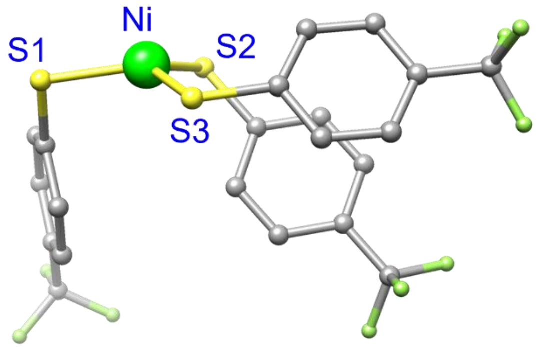

Figure S77. DFT-optimized structure (B3LYP/def2-TZVPP, gas-phase) of **^{3}1^{3C}** for the ECCE mechanism of H_2 evolution from **1** after heterolytic coupling to evolve H_2 (see Figure S76).

Table S19. Select metric properties of the DFT-optimized structure of **^{3}1^{3C}** for the ECCE mechanism of H_2 evolution from **1** (see Figure S77 for structures).

**Atoms**	**Bond Distances (Å)**
Ni-S_1	2.234
Ni-S_2	2.218
Ni-S_3	2.237
	**Angles (°)**
S_1-Ni-S_2	124.4
S_1-Ni-S_3	112.0
S_2-Ni-S_3	116.9
τ_3	0.12

Table S20. Cartesian coordinates for DFT-optimized structure of tetrahedral $[Ni(S\text{-}p\text{-}CF_3\text{-}Ph)_4]^{2-}$ (**1***, S = 1, B3LYP, def2-TZVPP, gas-phase).

Ni	0.46465529578580	-0.20143422215947	-0.04916990634501
S	-0.58184403872915	-1.12296462462491	-1.90315724370788
S	2.01431931367600	-1.92013521098563	0.15429566557847
S	1.13813005152755	2.00353942120657	0.32317853554982
S	-1.17904767294548	0.01742080072735	1.59034709393560
C	-5.28964333156493	2.37236410677879	-3.49110918101067
C	3.72172030496825	4.34968283327966	-4.63914116522548
C	-3.98455726032746	0.23995169635479	-3.58559140194731
C	-4.14481563079221	1.52989807866247	-3.05332724941286
C	-2.89678804597837	-0.53880259195763	-3.21913120756092
C	2.22416450600880	2.64366073349962	-3.54060615806805
C	-3.17845206646349	2.01493265184597	-2.15326681380428
C	3.11140761499444	3.72559523802001	-3.43450194816569
C	-1.91963428278885	-0.07615998918796	-2.30269048108518
C	-2.08992643548557	1.23242280144433	-1.78562493089247
C	1.65389099810031	2.07598544703933	-2.40660350818342
C	3.44989624168318	4.20490355340712	-2.15539091478997
C	1.94859751446931	2.57048456606994	-1.11358257905256
C	2.88041222042463	3.63754036286650	-1.02350765990853
C	-1.51535038270100	-2.73883258737955	1.32916076152213
C	-2.19662609853675	-3.92557367269026	1.56965463734696
C	2.78610239344400	-1.63116866739279	1.69479414321024
C	4.08063161196145	-2.14180057809536	1.95607502976137
C	-1.98047690892468	-1.50719478608651	1.84972943527410
C	-3.37429170337043	-3.93619375410584	2.33375872772639
C	-4.13052598202399	-5.18691986989997	2.59770092558447
C	2.15970063254318	-0.90204690689919	2.73520743754444
C	4.72072213150450	-1.92156436280794	3.17154454838283
C	-3.16601992615332	-1.53965182120776	2.63013450800485
C	-3.84477772245093	-2.72468418041290	2.87224372762015
C	2.79964597968751	-0.67140371246791	3.94204070678929
C	4.09102300241193	-1.17550450896157	4.17956637223463
C	4.76407608424307	-0.87041434383149	5.46903951839726
H	-4.72843699716033	-0.15557464777406	-4.28155155798106
H	1.94914678717836	2.25575203923376	-4.52464402964683
H	-2.78237300804639	-1.54277671127559	-3.63458213488761
H	-3.28337186754514	3.01623593599172	-1.72708236336305
H	0.95338638870162	1.24470700650014	-2.51502664903048
H	-1.35815030034026	1.63146432201882	-1.07903547310997
H	4.15052018055555	5.03854245367726	-2.05490308132659
H	3.13456725448621	4.02511508753655	-0.03380827935879
H	-0.60052534299331	-2.74881691876238	0.73559889267105
H	-1.81446576411174	-4.85553329315853	1.14305178045610

H	4.58333524518187	-2.71493439835040	1.17194531207409
H	1.14935110862718	-0.51926772384718	2.57981457165056
H	5.72052110658447	-2.32819753415986	3.34009604086010
H	-3.54752939155978	-0.59988832032518	3.03800831392246
H	-4.76221721444051	-2.71456650523020	3.46798757516556
H	2.28813741755491	-0.09508254680319	4.71797364221134
F	-5.05960529462144	3.05752292185870	-4.66098697171032
F	3.04391624584609	4.07522607736407	-5.78467044795965
F	-6.41524861068647	1.63833160359621	-3.73437439582070
F	3.78222038872094	5.71309513677835	-4.55580576140894
F	-5.64625390161354	3.32218857863732	-2.58471413609072
F	5.01974381430779	3.95949137527703	-4.86778817666046
F	-3.62688096081151	-6.27380022293331	1.95916304839497
F	-5.44570516261180	-5.10900115846959	2.22250126538024
F	-4.17669612747478	-5.52143926188720	3.93049568203505
F	5.09448440960713	0.45478908812772	5.60532214781806
F	5.92142595040723	-1.56676580243333	5.65113071928395
F	3.97939523806037	-1.13752848123544	6.55663504112891

Table S21. Cartesian coordinates for DFT-optimized structure of tetrahedral $[Ni(S\text{-}p\text{-}CF_3\text{-}Ph)_4]^{2-}$ (**1***, S = 1, B3LYP, def2-TZVPP, solvent phase = MeCN; modeled with CPCM, see Experimental section).

Ni	-0.22834257833900	0.34864284298828	0.30655586806436
S	-0.86238675278502	-0.79889300755078	-1.64217565392277
S	1.45966509284979	-1.17235768659345	0.73576587390892
S	0.41994305293603	2.57323970047395	0.59959798457272
S	-1.92029145223096	0.24867959000539	1.90736399884406
C	-6.81456917504926	0.34438038990793	-2.01020700477911
C	5.90841023760651	2.01592385839983	-1.94979426014827
C	-4.84521182127140	-1.16274152950164	-2.29677904135298
C	-5.34520782549010	0.09300906361050	-1.91949877049663
C	-3.48192114292710	-1.42276618448046	-2.21910157501138
C	3.44165463478103	1.62721376813975	-1.96304044614238
C	-4.46172194621184	1.08673650245649	-1.47691637356286
C	4.54938608253032	2.25872006473781	-1.37678128713854
C	-2.57647207825079	-0.43691574083412	-1.77081508887647
C	-3.09686578162536	0.82412360774940	-1.41141476302532
C	2.17948442105843	1.75982929953038	-1.39533206983575
C	4.37051472548819	3.04379044103261	-0.23158805382390
C	1.98611042722072	2.50942139392005	-0.21303947424828
C	3.10412527602147	3.16848899098111	0.33661515487758
C	-2.05950495591466	-2.41113408519215	1.16474961636941
C	-2.71044926671291	-3.55002910186447	0.70725494545729
C	2.83400963561364	-0.31805971833080	1.43175301162367
C	4.13124384347981	-0.57786124819653	0.94167072681268
C	-2.76992042145450	-1.22354153137977	1.43768699647141
C	-4.10302012918938	-3.54559313119568	0.53643979765859
C	-4.78155638130925	-4.73406364632576	-0.06565587323736
C	2.70321102958909	0.61259910121418	2.48595771597773
C	5.25069374206508	0.04256887971268	1.48962519066599
C	-4.17153985627896	-1.24240047771121	1.28493220893478
C	-4.83154940600792	-2.38744464582367	0.84283086409055
C	3.81841352993646	1.24234298153662	3.03121394979952
C	5.10302607931305	0.95499405743132	2.54313204145709
C	6.29323753329436	1.66338043265846	3.10755281434252
H	-5.52835552866686	-1.94479455450269	-2.63539065354560
H	3.57027769957728	1.01817819586081	-2.86099460785217
H	-3.10341066725408	-2.41079471523977	-2.48626278806445
H	-4.83865664199354	2.06704817366482	-1.18032767864684
H	1.32902134034916	1.25374216307222	-1.85578325863693
H	-2.41598911898824	1.60480621512727	-1.06859733848298
H	5.22328270498534	3.53889473877437	0.23388877445435
H	2.97926527801375	3.75533366405615	1.24799517260618
H	-0.97704646016254	-2.41938044679065	1.29297720812837

H	-2.13143435702812	-4.44508033158578	0.47105830657930
H	4.25500828178393	-1.27351555491423	0.10944248772793
H	1.70905690176986	0.85058708247263	2.86582245054488
H	6.24041493781171	-0.18146364691584	1.08751496922959
H	-4.74287266338806	-0.33360035767130	1.48377020870868
H	-5.91491563053805	-2.36868080837845	0.70850492131765
H	3.69071883143146	1.96431004513727	3.84106384123913
F	-7.29689069853130	0.14123672604127	-3.27302530939884
F	5.95458710065832	2.20916371052393	-3.30018207432946
F	-7.54857697913667	-0.48443759891901	-1.20846049141088
F	6.86842096426878	2.80947901509669	-1.41735618659124
F	-7.16243129449722	1.60722375540308	-1.66681067566440
F	6.33070410346524	0.72907441737088	-1.75550017733964
F	-4.92366074884188	-4.62824028258901	-1.42376227512016
F	-6.04037772038358	-4.93518717622216	0.41090485243432
F	-4.09622189794126	-5.89029811773206	0.13701277905677
F	6.47473496815288	2.91214882771902	2.57442822437772
F	7.45708635297447	0.99712200590062	2.89133422452612
F	6.20469256937357	1.85808162373365	4.45096606982568

Table S22. Cartesian coordinates for DFT-optimized structure of square-planar [Ni(S-*p*-CF_3-Ph)$_4$]$^{2-}$ ($\mathbf{1}^{\mathbf{sq\text{-}pl}}$, S = 0, B3LYP, def2-TZVPP, solvent phase = MeCN).

Ni	-0.43713852231881	0.84878180407403	-0.56851254930192
S	-1.47057408875865	-0.44694800344615	-2.09356991453536
S	0.52980309777665	2.33259761739463	0.82559407980068
S	0.97422673369358	1.26447771513670	-2.29220708634518
S	-1.69519942334551	0.33038154190726	1.22459911832380
C	-7.50374722038645	-0.04226465931843	-1.60386652371075
C	6.24839314022332	3.56775336207544	-0.31226520508437
C	-5.41049575647920	-1.31214977838856	-2.18417012023376
C	-6.01764923382575	-0.13698996461881	-1.71971050439700
C	-4.02537354579377	-1.38361348597638	-2.29342035537484
C	4.82375234631892	1.60553878038187	-1.01492964923839
C	-5.21926689929580	0.97035720634579	-1.39060247976767
C	4.96856672529491	2.98326082668482	-0.82262551817045
C	-3.20690159733650	-0.29031500966802	-1.94403705772391
C	-3.83572949024605	0.89441563037458	-1.50320082873859
C	3.60289328304140	1.09485222073942	-1.45715654296389
C	3.88909098921409	3.84315871017418	-1.08100753056579
C	2.50750043899574	1.93748861355105	-1.71707664120524
C	2.67614995087572	3.32396528711146	-1.52364853341258
C	-2.26332822883231	-2.31640303608219	0.57459625263404
C	-3.11596884006841	-3.40436555246108	0.41196876041675
C	1.98578942804326	1.56612027152071	1.40428550005054
C	2.19729208797054	0.17137648295783	1.30234641511799
C	-2.75630729601145	-1.06246087151130	0.98906227157275
C	-4.48874827006923	-3.26562902513457	0.66788640272738
C	-5.40758776910215	-4.43386207707545	0.50504910523559
C	3.01403885120251	2.34947534468106	1.97610882665108
C	3.38672363501442	-0.40777200011144	1.72882746793565
C	-4.13359529726059	-0.94932908755494	1.26126375953232
C	-4.99256878351845	-2.03489081088596	1.10822866979528
C	4.20539039220138	1.77293922913314	2.39983868444712
C	4.40461383932532	0.38850176877603	2.27572648220548
C	5.71490140175827	-0.21907106889986	2.65242721724630
H	-6.01966168763407	-2.18335451840536	-2.43127143753883
H	5.65196006723234	0.92935004859010	-0.80109274148697
H	-3.55823498833945	-2.31466812125600	-2.61999999973280
H	-5.68338455609501	1.89149663282481	-1.03098780111843
H	3.48417945130375	0.01788415998680	-1.58638860562231
H	-3.21795193921961	1.74949452922327	-1.22632769494715
H	3.99758769676723	4.91991920244889	-0.92817923889605
H	1.83625781301915	3.99193790060475	-1.71726204550838
H	-1.19861755402921	-2.42286521940680	0.36521462141555
H	-2.71589607136433	-4.36268455140752	0.07416269732591

H	1.41149324585171	-0.44872044600498	0.86778343539539
H	2.88018393582323	3.43019820804900	2.05595067537651
H	3.52964104599943	-1.48574237593122	1.62613510174980
H	-4.53667806230047	0.01753758454259	1.56737877339086
H	-6.06037577920793	-1.91559165776859	1.30133916169246
H	4.99263651743547	2.40494769938050	2.81532228941281
F	-8.08625114217579	0.56314617907847	-2.68792117969111
F	7.23130640431961	2.65131486361376	-0.15415881387383
F	-8.10719602856556	-1.25175731234617	-1.49260882142715
F	6.74933684973307	4.53110948047611	-1.14152022902377
F	-7.90267958976302	0.69042930567228	-0.52864107613226
F	6.09909746874516	4.18147694265583	0.89791117358245
F	-4.97951830244951	-5.31599864622032	-0.43547669475411
F	-6.66659788726157	-4.06922666855858	0.14943258975370
F	-5.54787700167301	-5.16393539721911	1.65603640831749
F	6.60977141937749	-0.24370565481440	1.61406433292314
F	5.60297579824390	-1.51026579470346	3.06211079259314
F	6.34318679792631	0.45220564500843	3.65388235390098

Table S23. Cartesian coordinates for DFT-optimized structure of $[Zn(S\text{-}p\text{-}CF_3\text{-}Ph)_4]^{2-}$ (**2***, $S = 0$, B3LYP, def2-TZVPP, gas-phase).

Zn	0.21000067934407	-0.61211084926248	0.65743105602139
S	-1.45621138815940	-0.84232255182931	-1.03360492263609
S	2.34087441465657	-0.63359781983742	-0.42823185818092
S	0.08262236015401	1.47975651843498	1.81034541399942
S	-0.00518338185127	-2.31154254769474	2.34311818416609
C	-0.22042036092712	2.88528732722103	-5.64769314982496
C	-1.06522896454875	0.73998369539870	-4.71360291643909
C	-0.47796266276461	1.98791059418419	-4.49204304034747
C	-1.34341592982258	-0.09820324412117	-3.65226062022332
C	-0.18284652697533	2.36887842202148	-3.18055819625372
C	-1.05705684208518	0.27000451755056	-2.32127195160725
C	-0.45282405560607	1.52405831796969	-2.11663190365363
C	-0.76778064042853	-5.36816499263051	-0.23031454301746
C	4.65120516193941	0.74449484756110	0.09857011645537
C	-0.44881247841039	-4.11475741700104	0.25391466029556
C	3.43293021713711	0.25002852051980	0.61189552801162
C	-1.23622698429800	-7.79059208635648	0.08243455908666
C	-2.67347881186480	1.33142314217883	1.51509897703425
C	5.54145243858523	1.45253615206806	0.88928293689177
C	-0.94115035349971	-6.45046774408231	0.64081534915440
C	-3.92223289657316	1.89811526013942	1.34332457828770
C	-1.50394714826258	2.10950423394125	1.45580383619179
C	-0.30599017378342	-3.87682544565979	1.63729792611577
C	-4.05497396108683	3.26377388598871	1.09606808475952
C	-1.65667921166056	3.48184762182991	1.18002351291537
C	-5.39529753479704	3.86476763358691	0.89556584019124
C	-2.90352715737706	4.04803617447449	0.99656745400443
C	3.18849038814417	0.47675719123116	1.97987491408419
C	-0.75943036579048	-6.24591368864696	2.00643826761568
C	5.24804121980442	1.71784416051193	2.22991213376475
C	-0.44773350200093	-4.99005749454352	2.49093177884829
C	4.06011688876797	1.20840166187467	2.76289479490606
C	6.17602024865548	2.48941665875404	3.09947776419361
H	-1.28693098865708	0.41905652288104	-5.72304099560970
H	-1.77747289797510	-1.07199888607458	-3.83451923250682
H	0.27948631178877	3.32840164870573	-2.99058385369949
H	-0.18761439649294	1.82523481540002	-1.11283313076213
H	4.88141757992611	0.56798333484327	-0.94378330771854
H	-0.90071371077219	-5.50319801683266	-1.29600365730779
H	-0.34005623288512	-3.29668080638940	-0.44346832105137
H	-2.58764891679392	0.27444488630307	1.72008067276108
H	6.45633293686093	1.83140024897319	0.45437133588752
H	-4.80460590780757	1.27625824330958	1.41289481248560

H	-0.76750134673585	4.09454393837004	1.11571399348211
H	2.28555153500204	0.08354738089601	2.42605363982904
H	-2.98905284736606	5.10632652038666	0.78897489920036
H	-0.87572789264255	-7.07415910219042	2.69333297696356
H	-0.31695274808161	-4.84415697498408	3.55479095633179
H	3.80952895476917	1.39034843441117	3.80006698188678
F	0.12455202816354	2.19461410175192	-6.77413606349389
F	0.76828931793556	3.78666108367739	-5.43223142516849
F	-1.30865821113061	3.63259510263113	-6.01621103059398
F	-2.17315646700853	-7.74058882675015	-0.90181577669371
F	-5.82081535515014	3.83794203814264	-0.40238121243187
F	-0.14714649223935	-8.39437808764478	-0.48768077887206
F	-1.68900821513434	-8.69073428779149	0.99387196148511
F	-6.36747267768190	3.23264757415111	1.59869441866725
F	7.25531643215362	2.97799030953561	2.43698530344697
F	-5.45125780462539	5.17491663308863	1.26422460278649
F	6.69025472134395	1.75989678380081	4.13660161669937
F	5.58754060662200	3.56277473162343	3.70111604918588

Table S24. Cartesian coordinates for DFT-optimized structure of **[Ni^{I}(S-*p*-CF$_3$-Ph)$_4$]$^{3-}$** (S = 1/2, B3LYP, def2-TZVPP, gas-phase).

Ni	0.71918085167846	0.23770318813261	-0.00846471120726
S	-0.08974961837850	-0.48695347442595	-2.17437491048599
S	2.24339987774593	-1.59280306503486	0.19246281919626
S	1.35715816588236	2.51396351267405	0.15018443337440
S	-0.71816282486654	0.29120016092126	1.93398307616558
C	-6.01468261614079	0.66585535758132	-2.70955578310689
C	3.77200942164296	5.11256389548986	-4.77663187475883
C	-3.85062044446612	-0.22404412574230	-3.63660452040898
C	-4.57882222900346	0.35877235333144	-2.57647329686635
C	-2.49235569282514	-0.46860551720474	-3.50748154252747
C	2.24109879056461	3.39077688560864	-3.71929913147781
C	-3.88504157609840	0.68176139934437	-1.38865074971717
C	3.18110863504285	4.44383853395037	-3.60375567113686
C	-1.77018668772163	-0.14523259522222	-2.31790799749615
C	-2.52825343926297	0.44070905036929	-1.25997447890458
C	1.70012219359273	2.77791308873269	-2.59813996080367
C	3.54106482419091	4.86889481890695	-2.30427209639954
C	2.04704740554243	3.19413292524076	-1.27583775808812
C	2.99433346616052	4.26389482280385	-1.18576248073729
C	-1.57693107192574	-2.20208011524406	1.03063726510258
C	-2.45229473101275	-3.28106304388667	1.03707970249049
C	3.03821293495969	-1.60795717149356	1.71993525438191
C	4.06670653715831	-2.55864929473629	2.01402842695520
C	-1.75608669390960	-1.08991343753640	1.90418115736335
C	-3.56514969572476	-3.31518789707004	1.90686903724151
C	-4.50116819191954	-4.45780375526571	1.95869598483450
C	2.70880942408200	-0.70036088031123	2.77186529047307
C	4.70031205409076	-2.60279747097937	3.24579670413891
C	-2.88847167404317	-1.14803339497475	2.77551928844049
C	-3.76468081883652	-2.22062254860172	2.77862390940443
C	3.33307954546387	-0.75099052592645	4.00639798939148
C	4.34349040497585	-1.70264780177960	4.27597149777492
C	5.01945846244014	-1.78510252054975	5.58349058457400
H	-4.36513099247820	-0.49383129193884	-4.56390749324003
H	1.94398099694852	3.03628876300665	-4.71061929108361
H	-1.93861015757908	-0.92792259325374	-4.33142095334890
H	-4.43055383491634	1.12461296662730	-0.55087910830141
H	1.00103447845925	1.94154719064464	-2.70850014041570
H	-2.01056690789751	0.68515644250204	-0.32926518827738
H	4.26959303392232	5.67787692828016	-2.18571599960816
H	3.29136791378377	4.59760496570986	-0.18751487920867
H	-0.73027337360407	-2.18337828269850	0.34055035546383
H	-2.27254821249837	-4.11251335042025	0.35057926286718

H	4.34922958548009	-3.26479092911507	1.22735320577278
H	1.91573564799273	0.02486962488228	2.58589195380134
H	5.48559628112169	-3.34420129606315	3.42861898819939
H	-3.06430787459726	-0.30016110597833	3.44318308600355
H	-4.63105258133590	-2.21286779930315	3.44804203217891
H	3.02929174270890	-0.04540347183897	4.78418385214586
F	-6.29681989370737	1.98878710314173	-3.03899869781475
F	3.59508952446557	4.44066412422152	-5.94979262665489
F	-6.64356164591611	-0.06151306838542	-3.68702967078311
F	3.28108875428437	6.39372152978893	-5.02898603780157
F	-6.73369269326635	0.45509666912247	-1.56237963542110
F	5.13221070654487	5.32620556587211	-4.66934695646394
F	-4.26105887788562	-5.41508281620323	1.02288978703848
F	-5.82941777053851	-4.11345189940961	1.81338657702487
F	-4.49487462567778	-5.14335334468070	3.16920263885318
F	4.67362664275744	-0.79044736837352	6.44952683592831
F	6.40085041200236	-1.74004630579951	5.49698901642735
F	4.79085873234790	-2.96603830743950	6.28237362953819

Table S25. Cartesian coordinates for DFT-optimized structure of tetrahedral **[Ni^{II}(S-*p*-CF$_3$-Ph)$_3$(MeCN)]$^-$** (intermediate **I** in eq. 1 of Scheme 1 in the paper; S = 1, B3LYP, def2-TZVPP, solution-phase = MeCN).

Ni	2.37078006377514	-0.46570690128490	1.38076172437739
S	3.11919217971070	0.33244838019245	3.44397525131615
S	2.27572946517686	-2.63727432568984	2.09705986786120
S	0.98030027047729	0.52898646550847	-0.14094633198390
F	-4.91704423544791	-0.91000688830175	2.81481959721748
F	6.00371600807000	5.90267779038438	0.92788713930168
F	7.77596270702234	4.90812867349451	1.71038203399377
F	-5.37561980182362	-1.36142829702632	0.73988585394059
F	-5.55613883565934	0.67704833745088	1.46649511093313
F	6.96749428209748	4.33186454777355	-0.22252474250990
F	-0.44943397597335	-7.49490926631205	-1.67738372621178
F	-2.20936917931775	-6.29239231753105	-1.26631541221347
F	-0.92944930103063	-5.79411784251570	-2.95096436150676
C	5.51128350668381	1.64747106364983	3.01841337573002
C	6.31848035100775	2.64094960012418	2.46730677225494
C	4.11471280763768	1.65316751385200	2.82433899612684
C	5.74320077080055	3.67101700630384	1.70985031550527
C	-2.32406575500117	-0.93189290682779	1.75401968028089
C	6.61198127442171	4.69651936131455	1.04864250534036
C	3.55196998236069	2.70752866330437	2.07424290187171
C	-4.78131738552061	-0.43462280807149	1.55155075327904
C	-1.00226209626819	-0.72394816717969	1.36804596959580
C	4.35388170509828	3.70016651310641	1.51700666802292
C	1.09427621471698	-4.97643870666943	1.30996728049462
C	-3.35459268694174	-0.17586015374463	1.17810899935111
C	1.37221281274648	-3.63541743178068	0.95888751990402
C	0.37499983811444	-5.80863946766369	0.45839929328138
C	-0.67305074055264	0.24468569757021	0.39626509524661
C	-3.04626365026681	0.78709431179580	0.20522579029699
C	0.90120851703729	-3.16590709920025	-0.28407695446629
C	-1.72388449988776	0.99386107006978	-0.17722408855906
C	-0.08684806822991	-5.32397294993977	-0.77583776634205
C	0.17900041504472	-3.99682232026266	-1.13797563059791
C	-0.90208114138326	-6.21252666576068	-1.66197833956744
H	5.96626796524549	0.83823788850882	3.59312156819376
H	7.39901164073904	2.60874707388600	2.62033574882137
H	-2.55179642467873	-1.68825344017822	2.50780839622079
H	1.45006046969729	-5.36373969316728	2.26754011615976
H	-0.21338803967718	-1.32616269613972	1.82243663561187
H	2.47326820827614	2.72840956771954	1.91000752779314
H	0.17342939952825	-6.84087694128076	0.75351144692626
H	3.89750306211596	4.49892665636059	0.92874046624996

H	1.09387268155448	-2.13285626968683	-0.57752566868762
H	-3.84084883408231	1.38067024511620	-0.25154911574644
H	-1.49276583078548	1.74887436465810	-0.93199894899200
H	-0.17799405132777	-3.60561970986515	-2.09265162618734
N	4.02204972812882	-0.15754664889310	0.28997372699137
C	5.00123551653475	0.23439053118289	-0.16142467711800
C	6.23510785583738	0.75427832715404	-0.71707198968491
H	7.06561350936934	0.54740111943150	-0.02639239510879
H	6.14149682636230	1.84157508887064	-0.85451138029568
H	6.43800449846633	0.27904405619007	-1.68813097271307

Table S26. Cartesian coordinates for DFT-optimized structure of *square-planar* **[Ni^{II}(S-*p*-CF$_3$-Ph)$_3$(MeCN)]$^-$** (planar isomer of intermediate **I** in eq. 1 of Scheme 1 in the paper; *S* = 0, B3LYP, def2-TZVPP, solution-phase = MeCN).

Ni	0.17661408914478	1.12040001664692	0.63758832169368
S	-0.44104187589865	0.65729862723206	-1.43375310641602
N	0.52206547501065	1.79033338289611	2.39185344037359
S	1.65132091371311	2.71416363610268	-0.01020213861328
S	-0.87462822215790	-0.59001737654998	1.65568687957674
C	0.71205738379550	2.36705313123029	3.36660706406272
C	0.96543404948875	3.12940026218003	4.57451141090006
H	1.62507654297405	3.97733119933623	4.33588227616544
H	1.45036704382063	2.49099190419227	5.32755631727677
H	0.01685589895086	3.51462002909794	4.97895097355877
H	-5.01162579427159	1.76981610522192	0.09053723430345
C	-6.49406609201365	0.22865300903179	-1.58140319965244
C	3.76827351096907	1.65624059385061	-5.60119588964385
C	-4.21268882430375	-0.31124534504481	-2.49925694860399
C	-5.00883079770876	0.39265749705841	-1.58621822732544
C	-2.82652624889498	-0.20610350439246	-2.43951198615505
C	3.18418629992794	0.86259583920945	-3.28748608901963
C	-4.39897979047007	1.21157775289976	-0.62144180310300
C	3.20624806713909	1.89444128669081	-4.23491428158215
C	-2.19485336228815	0.58863931609185	-1.46001791245641
C	-3.01318271319828	1.31081690786784	-0.56293246070158
C	2.70413671353594	1.11003665656328	-2.00484060425060
C	2.74216474383631	3.17263300600173	-3.88993985764120
C	2.22459825833466	2.38448578769589	-1.64179252043765
C	2.25171211230778	3.41068039453540	-2.60788779497466
C	-1.19979835927054	-2.43449578931497	-0.40251360613331
C	-1.95133800071089	-3.31940279372741	-1.17329601740422
C	-1.80683031188559	-1.65296428007855	0.59792523767390
C	-3.32879589725554	-3.44761189565662	-0.95205233610014
C	-4.15067058626105	-4.40587818558443	-1.75967776717043
C	-3.19763143683229	-1.77267082263759	0.78763133379169
C	-3.95300565164481	-2.66274687326676	0.02849546922697
H	-4.67451246781448	-0.96292138705735	-3.24242908953634
H	3.54801645171235	-0.13169254028669	-3.55174885491480
H	-2.21473268677029	-0.77900774474137	-3.13807223245867
H	2.69160958575300	0.30646262380269	-1.26662473181396
H	-2.54552976196933	1.94035045704554	0.19512166116869
H	2.75406467683164	3.97923495168152	-4.62679008665458
H	1.86564377251686	4.39914590121846	-2.34825303923941
H	-0.12863358420544	-2.33769816562255	-0.57984076959761
H	-1.46053449679258	-3.91745272989149	-1.94224674046383
H	-3.68868081207959	-1.14782879776036	1.53539538049104

H	-5.02903846167985	-2.74083947401515	0.19965250159982
F	-6.95796791522419	-0.43329664535700	-2.66978093708386
F	3.81596006714834	0.34267435832025	-5.93194379970745
F	-6.93848259762377	-0.47136852602106	-0.49180767126712
F	3.05267562581854	2.28022426405646	-6.57201023147280
F	-7.16108420680093	1.41569052318622	-1.53806059901867
F	5.04698163100139	2.12345452291754	-5.72895663618484
F	-3.39691308992930	-5.22574061286486	-2.53252357768018
F	-5.02210764559794	-3.78129498307856	-2.60418850044926
F	-4.92314122217680	-5.21335547091200	-0.97651345693455

Table S27. Cartesian coordinates for DFT-optimized structure of *S*,*S*-bridged/planar **[Ni$_2$(S-*p*-CF$_3$-Ph)$_6$]$^{2-}$** (**3***, *S* = 0, B3LYP, def2-TZVPP, solution-phase = MeCN).

Ni	0.94022508022728	1.37444569182013	-0.26127765728738
C	1.63742537957748	-3.02052482297127	-5.53309261552756
C	-4.59055700434348	4.00393957415823	-3.92887485044358
C	3.47411967615451	0.31907806521691	6.11396198398259
S	1.21798810756215	-0.81693117842340	0.14866952144106
S	0.75395131629104	3.43514375828367	-1.03976078881884
S	3.10741695001585	1.73007567364639	0.19264243817923
C	-0.77918622280194	3.57851314243660	-1.90696734081130
C	-1.56748381031880	4.74647824128642	-1.74490524854961
C	-2.79828427254068	4.88277339011189	-2.39765916731302
C	-3.27936175418536	3.84184212331888	-3.21969708767040
C	-2.50269742974458	2.67917528770687	-3.40091540376793
C	-1.26666272398253	2.55623359028339	-2.76102908351022
C	3.19611736786553	1.29084845297177	1.89229978695183
C	4.45349525134007	1.01027927170513	2.49048789512645
C	4.55107068809973	0.68782237365702	3.84833989881831
C	3.39089844182301	0.62000969643791	4.64929756369117
C	2.13260481864997	0.88874861492399	4.06816773603594
C	2.03883788474687	1.22171420733002	2.71630930232360
C	1.40241125955708	-1.43186908700922	-1.52020794505439
C	1.52263562804041	-0.55787640677337	-2.62327588228210
C	1.59605698720288	-1.06385137124042	-3.92800090781199
C	1.55571826802434	-2.45397840509720	-4.14291996614222
C	1.47331676391437	-3.33599377835633	-3.04257465589399
C	1.40087146375926	-2.82948071243347	-1.74215255215397
H	-1.21036710609974	5.54793182051404	-1.08030028124333
H	-3.39722245350226	5.79404655651344	-2.25061766855682
H	-2.87208231468989	1.85745507249759	-4.02892687185659
H	-0.67160505284628	1.64366334814119	-2.90604071618704
H	5.36471805706357	1.04224891245118	1.87406567824582
H	5.53607507179252	0.47378042476052	4.28700546173289
H	1.21658784758497	0.84075820405954	4.67543153500004
H	1.05576854597401	1.44514186721967	2.27763164994777
H	1.53276597445320	0.53100408836929	-2.44881786699521
H	1.67206478228253	-0.37016817642148	-4.77733442592462
H	1.45048613596051	-4.42363748887576	-3.20510301007752
H	1.30203659695743	-3.51287635655267	-0.88735507675404
S	-1.23887631875049	0.84524871259174	-0.13032247319328
Ni	-0.96102454267469	-1.34810699065725	0.27202607978912
C	-1.40968042310397	1.45379849102616	1.54255374886894
S	-0.77928689307993	-3.41189994175485	1.04277522266142
S	-3.12813234117380	-1.70377191318552	-0.18510191342338
C	-1.53344016396560	0.57518280182840	2.64176427857756

C	-1.39091222580770	2.85005128932923	1.77156209576051
C	0.75982068268573	-3.56910760970920	1.89717745577714
C	-3.20693850957691	-1.27963427533736	-1.88903500971406
C	-1.59263143324301	1.07526102222917	3.94931934718446
H	-1.55655678411359	-0.51259958376306	2.46167685124772
C	-1.44969090881110	3.35062618914947	3.07518112766577
H	-1.28680571760784	3.53659366272395	0.91970025842757
C	1.54334534925598	-4.73750472404614	1.71683552023081
C	1.25706773256182	-2.55712785642833	2.75782118286507
C	-4.46095542886194	-1.00566374801622	-2.49737841096684
C	-2.04456728070336	-1.21662411570944	-2.70644154696089
C	-1.53469493218343	2.46387399551499	4.17127389946002
H	-1.67094104266948	0.37815697946216	4.79570843218697
H	-1.41285075687174	4.43694097381874	3.24335868062786
C	2.77915769432394	-4.88411505173428	2.35802898191305
H	1.17847739919969	-5.53120843423501	1.04715536265246
C	2.49795325078529	-2.69011211408440	3.38611763596495
H	0.66573009325040	-1.64453039134772	2.91711314841875
C	-4.55042337890235	-0.69614698004507	-3.85874331170055
H	-5.37591776850475	-1.03278438330294	-1.88630730241319
C	-2.13045589930065	-0.89685076013997	-4.06202840589199
H	-1.06391802841038	-1.43456864958412	-2.25937155692623
C	-1.60497062659794	3.02036666628287	5.56614733262545
C	3.26999423491143	-3.85292564342367	3.18630503628083
H	3.37408692729003	-5.79556018989383	2.19689086280304
H	2.87482871034855	-1.87576461803782	4.01936999469669
C	-3.38551030824511	-0.63508853291363	-4.65330003734269
H	-5.53292076107751	-0.48713617820241	-4.30531682085866
H	-1.21097169221185	-0.85362532346843	-4.66437174851215
C	4.58560846500018	-4.02488798364751	3.88513837703632
C	-3.46001175134028	-0.34759465200641	-6.12116179441937
F	-2.86186420300294	3.48928854344240	5.87970928478676
F	4.66860628406130	-0.22892370568191	6.48514833499453
F	2.49425458604362	-0.55139875444247	6.52979297075302
F	3.31293360187058	1.44625357679652	6.89742203659970
F	5.53017157865943	-4.63014896663157	3.09420723740023
F	4.48569521392191	-4.82069912306210	5.01093777580321
F	5.12500416006820	-2.84397929014723	4.30830830576457
F	-5.55051355273991	4.58779606730434	-3.13969408647425
F	-5.11052674105807	2.82116514257095	-4.37016003463224
F	-4.49131400802450	4.81436116273952	-5.04421997087303
F	-4.65378274247422	0.19335909527696	-6.50503079103019
F	-3.29034032727966	-1.48118253361499	-6.89336312245509
F	-2.48020672754837	0.52224407752927	-6.53838034809105
F	1.37885727707323	-2.09362120272552	-6.50170396727388
F	0.75578700340721	-4.05237018555710	-5.72834187154251

F	2.88329524725009	-3.53611830132084	-5.81517551687885
F	-0.75594448018879	4.08045071725723	5.74917072626793

Table S28. Cartesian coordinates for DFT-optimized structure of the free thiolate anion **[S-*p*-CF$_3$-Ph]$^-$** (S = 0, B3LYP, def2-TZVPP, solution-phase = MeCN).

C	2.61813412392873	-1.30103351376578	1.55638005440262
C	3.54703977502784	-2.30171815268448	1.95105313919301
C	2.49550788309998	-0.17797387232172	2.41779107561136
C	4.29588476159137	-2.19054915873939	3.11702847596228
C	3.24241912350424	-0.06094366416692	3.58541076472008
C	4.14896180443332	-1.06942442134354	3.95212867782471
C	4.99314142473830	-0.93059425687716	5.17325866600768
H	3.66901460736212	-3.18206017533455	1.31546651743240
H	1.79053863339885	0.61273087050649	2.15014493332672
H	4.99898458906031	-2.98295037713300	3.38539020254810
H	3.11749991462100	0.81915392989671	4.22035771164322
F	6.23114041451917	-0.39722001172427	4.91295398083050
F	5.24136591241202	-2.12136378891650	5.78464139214947
F	4.44138181759986	-0.12001329691243	6.11336867971990
S	1.67683521470289	-1.44607011048346	0.09193572862793

Table S29. Cartesian coordinates for DFT-optimized structure of MeCN (*S* = 0, B3LYP, def2-TZVPP, solution-phase = MeCN).

C	-0.97355336199766	1.37215539850376	2.75078980103774
C	-0.50054247043789	0.65981970791616	1.57241093473730
H	-0.12886835205264	1.37818326864975	0.82649113802045
H	-1.32410918106222	0.07760564864442	1.13285162828702
H	0.31500856726520	-0.02362401733246	1.85219924464511
N	-1.34779520171479	1.93588999361836	3.68384725327237

Table S30. Cartesian coordinates for DFT-optimized structure of intermediate **[Ni^{II}(S-*p*-CF$_3$-Ph)$_3$]$^-$** (^{3}**1**^{3C}, *S* = 1, B3LYP, def2-TZVPP, gas-phase).

Ni	-1.16524556046077	2.67696996037419	-1.96707863177391
S	-2.02815823974039	1.16903233488640	-3.37569033314263
S	0.70138124109232	3.60395945036456	-2.77203879636769
S	-1.59786414771335	2.39885717138189	0.19094086185531
C	-6.69589575104170	-1.10927895543471	-0.21843878038888
C	3.92562631405989	-0.67206119763381	0.13802353624600
C	-4.67611920448942	-1.40237130490547	-1.70212904688941
C	-5.56866601272607	-0.55917438240873	-1.03207326919457
C	-3.61847841907619	-0.85979795456264	-2.42932083968057
C	2.90368690153177	0.27417428250241	-1.96185971705865
C	-5.40620986735518	0.83200971766509	-1.12101922347045
C	3.22838183919751	0.42603946927801	-0.60839346021814
C	-3.42858497597924	0.53247255258712	-2.51407900374005
C	-4.36069125674372	1.36823007553983	-1.86150788469690
C	2.16583054022869	1.25660076534800	-2.62089526136988
C	2.80732292024732	1.57770997354964	0.07494513240032
C	1.72262877378422	2.40964283053038	-1.94651474417755
C	2.06868077225578	2.55422985433096	-0.58594090091889
C	-0.72602112940810	-0.19480675587930	-0.28004428807217
C	-0.86791078970192	-1.57615252137594	-0.23289041831449
C	-1.67893285327374	0.64445710513190	0.33533671520700
C	-1.95727931816312	-2.15525510458208	0.43619629210683
C	-2.16801844481433	-3.63301002428430	0.35542897237836
C	-2.74090820784631	0.04906261570439	1.04130082255499
C	-2.88462475314022	-1.33554354037448	1.08871689280830
H	-4.77296620820275	-2.48544237507642	-1.61428150976677
H	3.22433301863898	-0.61883950644696	-2.50158321420266
H	-2.89679422763580	-1.52111213569490	-2.91110841440136
H	-6.09324870350881	1.49510437458732	-0.59077966749456
H	1.88968145250594	1.12150770317008	-3.66809431248416
H	-4.23302092157823	2.44983751233441	-1.92195664046819
H	3.05431748476604	1.70509884333723	1.13115380792459
H	1.71787396313575	3.43231805283699	-0.04286218647208
H	0.11642661032922	0.24322817772925	-0.81680223377417
H	-0.13459917507951	-2.20976235980225	-0.73557870241281
H	-3.48792642888145	0.68734523535090	1.51498003682790
H	-3.74082709401628	-1.77861030938127	1.59995644380900
F	-7.91094689536071	-0.95795582768713	-0.82619718610681
F	4.72725092510299	-1.42168039747310	-0.66051095135894
F	-6.56784384195874	-2.43260244034096	0.03946476594941
F	4.70911180304790	-0.20661964295252	1.14757474884103
F	-6.81567143431881	-0.48734195511138	0.98840402103334
F	3.05195022063100	-1.54504879522977	0.71405682631792

F	-2.72679461491003	-4.01365434757350	-0.83766064584922
F	-2.99858473223512	-4.10760740261009	1.31492840460880
F	-1.00788157119485	-4.33402882169917	0.45261198339769

Table S31. Cartesian coordinates for DFT-optimized structure of intermediate **[Ni^{I}(S-*p*-CF$_3$-Ph)$_3$]$^{2-}$** (^{2}**1**^{R}, S = 1/2, B3LYP, def2-TZVPP, gas-phase).

Ni	0.40640505264361	-0.40532106384529	1.83984656987211
S	0.51269167878150	-0.96733222419871	-0.39882951277323
S	1.45020190628014	-1.93232340650363	3.17023620962001
S	-0.83505549917331	1.51625577623869	2.07955018628263
C	-3.34657146718590	2.62967348010081	-3.41146521102060
C	1.01698327639603	5.17804653823158	-2.39733590669956
C	-2.83560254117767	0.99109767794642	-1.59511405796312
C	-2.39391736713361	1.73773706995862	-2.70478541202235
C	-1.95719259904819	0.17984064175396	-0.89388008744412
C	1.47544716544733	3.42740075049522	-0.66420224618076
C	-1.04377993142136	1.65616449867275	-3.08107514526669
C	0.57209464957920	4.32234166173697	-1.26840641765918
C	-0.59092949700663	0.06748420041619	-1.26478406932215
C	-0.16865995974662	0.83627631585502	-2.37663834980651
C	1.05940788109876	2.58025162067441	0.35150125545823
C	-0.76211161504905	4.33526962414902	-0.82924795628562
C	-0.28158939996472	2.58254502180597	0.81942573313107
C	-1.17280191453679	3.48977802091223	0.19377661412304
C	3.12934214526513	-2.07790391064656	2.76174542877236
C	4.01304796911428	-2.79968492690499	3.61374783492721
C	3.69954615060097	-1.51787958376355	1.58450272195047
C	5.35979599943844	-2.94891897209015	3.31900190628312
C	5.04740728551869	-1.66730788540612	1.28922701811314
C	5.90279705149321	-2.38788405968494	2.14649838285474
C	7.35415314590657	-2.50232784473646	1.86868034864287
H	-3.88015798737314	1.05963362486486	-1.27813554144925
H	2.51378842867122	3.39270100031799	-1.00441460103334
H	-2.30012014212494	-0.37934519136366	-0.02320844297307
H	-0.67308432221751	2.25419242420492	-3.91557652331920
H	1.75995416492269	1.87344549683870	0.79836905250016
H	0.88429082397511	0.79513317153639	-2.66268514891119
H	-1.48651846061969	4.99812609994282	-1.30679948584825
H	-2.21734440045560	3.49192346204881	0.51164734922302
H	3.60125448242227	-3.24403560875453	4.52369514528976
H	3.04522368289524	-0.97272582963538	0.89970894843288
H	6.00654934267145	-3.51125219699704	3.99862826193983
H	5.44642357671590	-1.22616981764790	0.37242218719833
F	-3.73603166529113	3.72460166336848	-2.67756489790802
F	2.27669924731474	5.68812685603105	-2.22257965582400
F	-2.86136976262770	3.13081850924370	-4.57949160778248
F	1.07815844513701	4.52532529390567	-3.60556657019453
F	-4.53016047277392	2.00797221477082	-3.73487732461310
F	0.20533017196722	6.25209770334610	-2.61553486971088

F	7.66906334012377	-2.37248180417912	0.54997575279298
F	7.88976814447134	-3.69721449215176	2.27261511520192
F	8.12588379607548	-1.55371160085849	2.50988701940137

Table S32. Cartesian coordinates for DFT-optimized structure of the tetrahedral intermediate **[Ni^{III}H(S-*p*-CF$_3$-Ph)$_3$]$^-$** (**^{4}1^{R}NiH**, *S* = 3/2, B3LYP, def2-TZVPP, gas-phase).

Ni	1.26708713033881	-1.27751056447538	0.02755545940783
S	0.34617734476683	-1.86990720742594	-1.91340666441321
S	2.94571718351299	0.16092461606015	-0.27737711958210
S	0.00901342971834	-0.38199769384380	1.65947580085756
H	1.95951865366405	-2.61780601853962	0.48885896701667
C	-3.28455599866788	2.38106573673062	-4.28887809308165
C	-0.06230090379909	4.63695420194807	-3.08450050412426
C	-1.76128102805991	0.40437992496716	-4.49157124090774
C	-2.33987827945821	1.39027689213003	-3.67845484301485
C	-0.93687358411207	-0.56525270132124	-3.93333015709921
C	1.24281250161128	2.47993000215800	-3.15096805587101
C	-2.07532975244077	1.38941342594646	-2.30295537532902
C	0.63794638113815	3.49823513867520	-2.40438869731635
C	-0.67448851092567	-0.58715765511862	-2.54519444785144
C	-1.25652544942286	0.41293972071217	-1.74126916142560
C	1.94059264868563	1.46275803535655	-2.50446542325787
C	0.72666692908767	3.48201539826144	-1.00549183660625
C	2.04890244864213	1.43969203706575	-1.10060479384317
C	1.41882211303256	2.46122668718212	-0.36171752743434
C	-1.94730791321498	-2.26339392694710	0.98939853236755
C	-3.11998283291318	-2.96784956445620	1.24798284293485
C	-1.44221602848413	-1.33428114971738	1.92295606890324
C	-3.82752197260117	-2.76207204164058	2.44032717712799
C	-5.05956905433541	-3.55432459785922	2.74701814757253
C	-2.16396379739979	-1.13988088767062	3.12259755084197
C	-3.33719332318900	-1.84041287886691	3.37805618840953
H	-1.95569899604043	0.40346262303582	-5.56651699967309
H	1.14982443023696	2.47336993851909	-4.23795416720155
H	-0.47919734892196	-1.32647859861585	-4.56894215279941
H	-2.51035581257640	2.16001440453819	-1.66473242528030
H	2.39309185187738	0.65376188679486	-3.07975057297778
H	-1.05447876061596	0.42530255757407	-0.66930048229853
H	0.24019278148865	4.26545723002821	-0.42056421295855
H	1.47255159494682	2.43372746093767	0.72777664399094
H	-1.41346018369359	-2.42407245977672	0.05130481524018
H	-3.49370125223106	-3.67927882329788	0.50888218849138
H	-1.78562003452512	-0.42398911671007	3.85567223768888
H	-3.88050136317039	-1.67161318075278	4.31050077514417
F	-2.85232759506329	2.84168926966709	-5.49224765154809
F	-0.44782436179348	4.34117566577347	-4.34601640670410
F	-4.51542501380678	1.83367408260816	-4.53081007730920
F	-1.16834207731385	5.04273919500621	-2.41260603589010
F	-3.50130180800619	3.45990179485303	-3.50487188507529

F	0.73672283866318	5.74357873706449	-3.18251823452479
F	-5.68474233901706	-4.01056928802499	1.63342148790232
F	-5.98036763726095	-2.83901015233412	3.44793791792375
F	-4.79944724835070	-4.65956815619911	3.50865244357763

Table S33. Cartesian coordinates for DFT-optimized structure of the square-planar intermediate **[Ni^{III}H(S-*p*-CF$_3$-Ph)$_3$]$^-$** (**^{2}1^{R}NiH**, S = 1/2, B3LYP, def2-TZVPP, gas-phase).

Ni	-0.83031208185382	1.18608139918060	-0.17534993837112
S	-1.09414615413026	0.05352919689177	-2.03355596068749
S	-0.15605224140161	2.96727026212370	0.99768925539154
S	-2.18168870884776	0.42122097972698	1.41001594405410
H	0.55006153121232	1.28977559797189	-0.63384347324879
C	-7.03048645384843	-1.22538602816389	-2.35380203023541
C	-4.61286045116277	-1.84287883600403	-2.68022197456940
C	-5.57250062534267	-0.88579615089856	-2.32947120090086
C	-3.25736217486795	-1.53773884611363	-2.58406336146623
C	-5.15869426327473	0.37761653176165	-1.88236938133317
C	-2.82571417096617	-0.27689343478136	-2.13147229935701
C	-3.80434236726972	0.67872277489897	-1.79092449997785
C	-1.85055937096181	-2.20397426434452	0.55672531367782
C	-2.31836306061153	-3.46218448675309	0.19404460712510
C	1.05486385768088	2.46268023954098	2.14995896755097
C	1.48283529086593	1.12636946809454	2.31002334699469
C	-2.74380766522786	-1.18061585001565	0.93306091805497
C	-3.69455307533373	-3.73409128723998	0.20303133105778
C	-4.18988078988306	-5.06840792444181	-0.26009383629109
C	1.64366723261455	3.44616552430904	2.98023592666141
C	2.45581289366875	0.79306283073832	3.24703177273074
C	-4.12094724710420	-1.47084501865909	0.94240857657448
C	-4.59446639809760	-2.72890120868943	0.57722688131350
C	2.61265152112642	3.11171171003843	3.91729310376912
C	3.02981036398375	1.77863479775849	4.06226173813293
C	4.11378196709674	1.43698235873766	5.03276949692194
H	-4.92347031285267	-2.83604526575646	-3.00782252424580
H	-2.51182623052322	-2.29397422873053	-2.83488800579314
H	-5.90215418013774	1.12808997962250	-1.60561006278282
H	-3.48942994038665	1.66224958872991	-1.44060501850740
H	-0.78114208751414	-1.99263914867094	0.53880851482381
H	-1.61163175023785	-4.24072666361263	-0.10131846546885
H	1.02967332499207	0.34688605316842	1.69781874417601
H	1.32376118968617	4.48544955475819	2.87579723075921
H	2.76726035231768	-0.24814885689943	3.35219058071479
H	-4.82582109770510	-0.68479187804167	1.21837398669491
H	-5.66799626437706	-2.92304252500808	0.56709177958435
H	3.04992080688158	3.88931036343103	4.54776515679732
F	-7.80843578040723	-0.16649343555581	-2.70437304056446
F	-7.32460757204449	-2.22846211365608	-3.21724478848304
F	-7.49425318720274	-1.63252017906591	-1.13415619641994
F	-4.26921024667968	-5.15500793750210	-1.62302387374337
F	-5.42922864298046	-5.36280600566982	0.20328822196860

F	-3.37658035290925	-6.08995510694318	0.12076801242732
F	5.36695096150270	1.65167381473333	4.52607485375158
F	4.08991262522845	0.13562094203896	5.41635109612383
F	4.05322102728615	2.18147271296234	6.17059457461443

Table S34. Cartesian coordinates for DFT-optimized structure of the tetrahedral intermediate **[Ni^{II}H(S-*p*-CF$_3$-Ph)$_3$]$^{2-}$** (**^{3}1^{R2}NiH**, S = 1, B3LYP, def2-TZVPP, gas-phase).

Ni	1.53143816010691	-1.27852365879263	-0.06397297660396
S	0.42891992809182	-1.97268072463188	-2.04109273315201
S	3.22513785609028	0.39998749982947	-0.07338026662084
S	0.21929462868257	-0.47702251137111	1.70747070468442
H	2.20719474549221	-2.62830464047887	0.38271459709328
C	-3.52012812106627	2.20842968724323	-4.00109946383575
C	-0.10961569994359	4.53247033249970	-3.03730352325198
C	-2.02181339576411	0.24137297089447	-4.37690967600057
C	-2.50713223378237	1.23685329293764	-3.51043860837171
C	-1.12810724031637	-0.71598309101909	-3.91731874988174
C	1.25214249737605	2.41740014109053	-3.00480080140747
C	-2.06554626378545	1.24443816908150	-2.17637660113117
C	0.64138423310741	3.47309135819214	-2.30959229094555
C	-0.67583259416023	-0.73149509614614	-2.57132158579594
C	-1.17036594706658	0.28576214221760	-1.71730126270485
C	2.02240264165127	1.48130095985766	-2.32514022705938
C	0.80099714456176	3.55389637799689	-0.91587400250196
C	2.22141748682471	1.55844461660379	-0.92439941339281
C	1.56644807659895	2.61198828957979	-0.23958930900132
C	-1.78447125139994	-2.22923731117247	0.89155210346551
C	-3.01219170137099	-2.85868012414777	1.05011556719554
C	-1.30529591752204	-1.29407321725830	1.84701989330142
C	-3.82775143748785	-2.58771118168206	2.16495263073933
C	-5.10800283676328	-3.30668169262354	2.37660558288603
C	-2.14674590765652	-1.03137885303173	2.96383313621099
C	-3.37200232464546	-1.66161177576286	3.12270463969727
H	-2.34934293467251	0.22581930047424	-5.42025748867517
H	1.09842142850936	2.31698197224022	-4.08124939856810
H	-0.75114003459981	-1.48337967193099	-4.59828265458632
H	-2.41725312720408	2.01838065891438	-1.49150871775347
H	2.46919350528764	0.64388120433201	-2.86330632461890
H	-0.82801800288924	0.32033258615074	-0.68100300257320
H	0.30817563587245	4.35604154647421	-0.36060396239429
H	1.66800658428131	2.66099134444801	0.84615547046699
H	-1.16969141339979	-2.44190490837418	0.01371296848299
H	-3.35016951682209	-3.56829896454870	0.29098087933786
H	-1.80327573127937	-0.31013727098838	3.70993529172266
H	-3.99204904135281	-1.43528256560324	3.99472201898268
F	-3.27606293826207	2.64585005636271	-5.27372134624171
F	-0.62318124088339	4.11944227663461	-4.22126063400531
F	-4.79394174143205	1.67800764942874	-4.06530347276740
F	-1.14154556161938	5.05251228517952	-2.31810048024694
F	-3.63522537653190	3.31452516224664	-3.22711556699288

F	0.67856706482344	5.62567159748836	-3.35293667313771
F	-5.65675786436532	-3.79360798947428	1.23057151231552
F	-6.07271742068556	-2.53353084427125	2.96450362726598
F	-4.99990679862763	-4.40310738508932	3.20998059037202

Table S35. Cartesian coordinates for DFT-optimized structure of the square-planar intermediate **[Ni^{II}H(S-*p*-CF$_3$-Ph)$_3$]$^{2-}$** (^{1}**1^{R2}NiH**, S = 0, B3LYP, def2-TZVPP, gas-phase).

Ni	-0.51126073176139	1.56712137069049	-0.96312157913434
S	-1.30535636821387	0.35958529919978	-2.66043342905583
S	0.70541336054778	2.98164560576480	0.25169132253479
S	-1.99263525430285	0.98517161930968	0.73648024408954
H	0.36760651224684	2.05229325684374	-2.03663409636939
C	-7.05608267807069	-1.41892120417300	-1.83601396648637
C	-4.68553842728461	-1.87717622439502	-2.54717987291502
C	-5.65712834518208	-0.98056683295692	-2.07632850896946
C	-3.36940504257191	-1.45792774017062	-2.71160287798339
C	-5.27336412145198	0.33776615437521	-1.76439978230106
C	-2.96241974503484	-0.13766783331334	-2.40848051785714
C	-3.95856790971982	0.74903666830555	-1.92793093685008
C	-1.50283316269759	-1.67259767566587	0.12816236357257
C	-1.86249320231569	-3.00767057726107	0.02423948128272
C	1.28706738185088	2.31181163673183	1.73881193657685
C	1.18737360466085	0.93858441455702	2.09348086407975
C	-2.40971139628489	-0.69277836743236	0.61480678901411
C	-3.14860825362822	-3.43818361054378	0.40236208541915
C	-3.54528874617065	-4.85477500582179	0.21106668534204
C	1.96043096927161	3.16009245213183	2.66424914572362
C	1.72824238848828	0.45406313074015	3.27587476932635
C	-3.69723847105510	-1.15229265691001	0.99570859642208
C	-4.06358966868801	-2.48686593829915	0.88665821945488
C	2.50202357130749	2.67613415534766	3.84465913638601
C	2.39374912988358	1.31057500934968	4.17526808800918
C	3.03376796240075	0.79069020591347	5.40567922686417
H	-4.95600694820433	-2.91300733394529	-2.76108982176584
H	-2.61344082492435	-2.16814451037948	-3.05248940022462
H	-6.01790793903740	1.04281494890942	-1.38510128755709
H	-3.66359735774520	1.76585378377881	-1.67079630182219
H	-0.51528660395447	-1.34615281421946	-0.19883252316254
H	-1.14176551109820	-3.73191778172813	-0.36471296791047
H	0.65555252047423	0.26917865660266	1.41859224954371
H	2.04478580113670	4.22286846120426	2.42176598212054
H	1.62733834846746	-0.60761097735307	3.51421450496241
H	-4.42339521677274	-0.41825052799893	1.35085647947991
H	-5.07553464840878	-2.79446179124109	1.15869959185997
H	3.01113601966226	3.35968028038837	4.53019028494140
F	-7.98474432453899	-0.47955266549452	-2.20602266350673
F	-7.39072096833744	-2.55755271756973	-2.50543055610402
F	-7.34134906666239	-1.67865795330569	-0.51799897467930
F	-3.91995910917912	-5.16335590148741	-1.07771815378207
F	-4.60389319525231	-5.23190035362676	0.98391579190055

F	-2.53891499237757	-5.74096697126525	0.49131065983694
F	4.38527632921412	0.52901317603169	5.26871459778449
F	2.49867070287294	-0.37990925282588	5.84867922009423
F	2.97126362844191	1.66313493320757	6.46066990181495

Table S36. Cartesian coordinates for DFT-optimized structure of the tetrahedral intermediate **[Ni^{III}H(HS-*p*-CF$_3$-Ph)(S-*p*-CF$_3$-Ph)$_2$]** (**^{4}1^{R}NiH,SH**, S = 3/2, B3LYP, def2-TZVPP, gas-phase).

Ni	1.58359891053471	-0.65594093971223	0.71882044793857
S	0.91155416347343	-1.92209353001372	-0.94067156398958
S	3.19617474178923	0.83103504584775	0.49060548669161
S	-0.30519117165560	0.59306143531650	1.50054077170917
H	1.72723701307214	-1.64661133125539	1.93337930466300
H	-0.04067187194284	0.41190087306778	2.81095459848892
C	-4.12266763373992	0.75218481731617	-3.03231791744589
C	0.54330199935218	4.51945640700633	-3.54529349803870
C	-2.82568633839224	-1.29344610855990	-2.34003829892518
C	-2.84906766692244	0.08461378044129	-2.59045003209700
C	-1.67030277561843	-1.89383460204762	-1.84705710567201
C	2.14928625850870	2.65759661295980	-2.97547395232735
C	-1.70499245066999	0.85703497173528	-2.35631715599470
C	1.27176494446175	3.66048289612211	-2.54489311842431
C	-0.52042617061239	-1.12407617818390	-1.59034465315343
C	-0.54864500658772	0.25698088751058	-1.86243117871806
C	2.75542208307827	1.81563250684637	-2.04491855772978
C	1.01876046588795	3.82962576123443	-1.17717068327635
C	2.48445112441511	1.95718391848507	-0.67109577736500
C	1.62495906037749	2.98834133307511	-0.24758998198971
C	-2.91168093688891	0.07953854657031	0.86973850600869
C	-4.02933848144490	-0.73416672900452	0.68146531121539
C	-1.73396754460459	-0.47363128786564	1.37913958101418
C	-3.96270989803569	-2.08995674754832	1.01279695260116
C	-5.14220263800346	-2.98632919215764	0.72550779413365
C	-1.66166557537388	-1.83150088743636	1.71402625380709
C	-2.78348384438220	-2.63748487618048	1.53424693391701
H	-3.71640756448691	-1.89783143095368	-2.51854577709569
H	2.35125053949331	2.52992404386381	-4.04012729895620
H	-1.65843136609934	-2.96521009227156	-1.64010720690894
H	-1.71073841759005	1.92754568350707	-2.56853264055620
H	3.42424057924334	1.01991684317191	-2.37685579648854
H	0.34277429567604	0.85902925931021	-1.70078026913451
H	0.34581336055818	4.62076435827963	-0.84107446331274
H	1.42769283439530	3.11862508920351	0.81742067647706
H	-2.95299271769624	1.13573073579344	0.60119404504307
H	-4.94451855947203	-0.31073023008021	0.26634342106184
H	-0.72876669898800	-2.25537542470325	2.08752635285231
H	-2.73829057365430	-3.69770195118042	1.78813356149368
F	-4.94535422663628	-0.08820462334285	-3.69504101433444
F	1.18860578765025	4.59506466819742	-4.72790058490232
F	-4.83270054693551	1.21954338517576	-1.96083661528735
F	-0.69851664612334	4.02613508701470	-3.80811305237500

F	-3.89840727438448	1.81695237860861	-3.83010844595815
F	0.36542365637964	5.78326732522509	-3.09867085104774
F	-6.31727801109124	-2.33004387504069	0.81800040703691
F	-5.19859981452117	-4.04190261506151	1.56553682307876
F	-5.07336939579276	-3.49240599828619	-0.53482973772706

Table S37. Cartesian coordinates for DFT-optimized structure of the square-planar intermediate **[Ni^{III}H(HS-*p*-CF$_3$-Ph)(S-*p*-CF$_3$-Ph)$_2$]** (^{2}**1^{R}NiH,SH**, S = 1/2, B3LYP, def2-TZVPP, gas-phase).

Ni	-0.90186239164210	-0.22831680206489	1.42353980730155
S	-0.92397915597209	-1.29483508873511	-0.57557927474999
S	-0.64568983509967	1.51273156675135	2.59000434102760
S	-2.50230311169319	-1.28722546477987	2.48756885909227
H	0.47318228650178	0.10561727139888	1.07827786833591
H	-0.17505267751840	-0.44601910068866	-1.31082138914584
C	-6.38345287677983	-0.25090309345349	-3.02990632995229
C	-4.29137737895971	-1.62579438472014	-2.80113756802284
C	-4.99165000003782	-0.45932054281439	-2.48261441975697
C	-3.03980049077324	-1.86039859412935	-2.22983422767098
C	-4.44490479457329	0.48059691198369	-1.60006591931374
C	-2.50267821406533	-0.92477792632371	-1.34339584593090
C	-3.19755256961852	0.24802998108099	-1.02572295760913
C	-2.73171851026516	-3.58209134155100	0.95147130456433
C	-3.40397513042825	-4.44838579917044	0.09179608754609
C	1.02103401222363	1.98890615366417	2.92908017244666
C	2.11373866925069	1.10487963064123	2.92438812753716
C	-3.33706187705382	-2.38574906342437	1.38119641432198
C	-4.69692052528080	-4.13773170072438	-0.34750598927695
C	-5.37391202021871	-5.04809175557851	-1.33432522748444
C	1.23363005092493	3.33306500651393	3.29015117843252
C	3.38946277626751	1.55770779208624	3.25156585771924
C	-4.64203113979611	-2.09446383616050	0.94516024259596
C	-5.31675613287224	-2.95970170665234	0.08638068639992
C	2.50956676598752	3.78512052040879	3.61740624994396
C	3.59359364032260	2.89894095336110	3.59859655922950
C	4.98133380466052	3.40060762144495	3.89865976747453
H	-4.72514157945013	-2.36214020316775	-3.47843594301367
H	-2.49961798042694	-2.78049085808256	-2.45505738604973
H	-4.99786183864581	1.38863309961371	-1.35496437648793
H	-2.77561713364867	0.96391420877053	-0.31834639079972
H	-1.72910408253606	-3.82975097930390	1.30466948322174
H	-2.92397056581870	-5.37300390508877	-0.23598811810965
H	1.95700985462901	0.05562392016550	2.67104809990618
H	0.38910207984540	4.02517345411431	3.30792062731567
H	4.23067631123614	0.86235936801499	3.24686243151597
H	-5.12151574952838	-1.17267738571742	1.27827308297336
H	-6.32280073558288	-2.71314037382753	-0.25582238508288
H	2.66391161983368	4.83042023408238	3.89078289168398
F	-6.69010450066024	1.05833484515986	-3.15479721299371
F	-6.54619657967170	-0.82356587824486	-4.24031210613048
F	-7.31609165991260	-0.79592819271541	-2.20665912305811
F	-4.85514643248015	-4.89451716649073	-2.59065856935409

F	-6.69811515780825	-4.81503763886231	-1.43693142630720
F	-5.21366398108821	-6.35450877627130	-1.02323368325950
F	5.59928764001081	3.86779183753097	2.78163282608508
F	5.77855026345747	2.43125277306334	4.40283131125885
F	4.97493703475542	4.41931040889282	4.78872159163066

Table S38. Cartesian coordinates for DFT-optimized structure of the tetrahedral intermediate **[Ni^{II}H(HS-*p*-CF$_3$-Ph)(S-*p*-CF$_3$-Ph)$_2$]$^-$** (**^{3}1^{R2}NiH,SH**, S = 1, B3LYP, def2-TZVPP, gas-phase).

Ni	1.65347605860115	-0.58148009822371	0.63103258187285
S	0.80769179274423	-1.86281814171588	-1.09634774756919
S	3.02894619483429	1.24251737560068	1.00826292645948
S	-0.33960114736349	0.61618553544472	1.46513595029174
H	1.88369106558290	-1.47461026114189	1.90414892895071
H	0.06031768702081	0.26250732683751	2.70051895158428
C	-4.26267934011296	0.98210046182211	-2.86164603916959
C	0.74579428323165	4.20786486145384	-3.79885361532139
C	-2.95127216831223	-1.11166767955686	-2.39561092440707
C	-2.98792267942853	0.28708131759835	-2.51210952171861
C	-1.78870652077948	-1.75053656699442	-1.98187018256705
C	2.26943931262891	2.45049950698317	-2.80806345418237
C	-1.83463779915076	1.02964683623862	-2.21828356060296
C	1.32829471521547	3.47427176863117	-2.63231532771234
C	-0.62013517432006	-1.02116912916919	-1.65946998108436
C	-0.67248240450304	0.38573524340319	-1.80434067694835
C	2.80114847550707	1.79040418842283	-1.70331987693333
C	0.94130240497660	3.83573118477135	-1.33202527108226
C	2.41451228374226	2.12705042272910	-0.38615185886910
C	1.47857740467620	3.17687796426884	-0.23176831892217
C	-2.95706326987167	0.00014300451852	0.92088716188555
C	-4.03298423249545	-0.86497099314560	0.72456379253027
C	-1.72350744042141	-0.50323344261767	1.34517218675664
C	-3.87480177594084	-2.23278938182305	0.96880691679865
C	-5.01128380709019	-3.17566349510178	0.69115350053710
C	-1.55862561965603	-1.87621123703662	1.57739891531040
C	-2.63858536568964	-2.73637572015857	1.39642493129994
H	-3.84477245744456	-1.70020967374418	-2.61332318534673
H	2.57015040948295	2.15508697949973	-3.81467358873843
H	-1.77297581213602	-2.83673694427800	-1.87487449154655
H	-1.83889327111475	2.11672350181308	-2.32193743220445
H	3.51121689699720	0.97401517462284	-1.84515755290962
H	0.21989239941901	0.97393903974137	-1.60031720035179
H	0.20546467981453	4.63042789354852	-1.18597180262531
H	1.16411374228183	3.45066692414438	0.77693403145041
H	-3.07061427982251	1.06612234012250	0.72035491597667
H	-4.98696783844152	-0.47306602653951	0.37015962079725
H	-0.57727204585380	-2.25992405068905	1.86106170033380
H	-2.51418091048757	-3.80697569230186	1.56740521926479
F	-5.08535295095325	0.22566620561130	-3.63383111446112
F	0.99705266962793	3.60529657089625	-4.98515207823792
F	-5.00544163622890	1.30846573075139	-1.75141097525279
F	-0.60541255162960	4.34444094486143	-3.70576009796452

F	-4.06883837783384	2.15074249000089	-3.52067849856512
F	1.23034569874132	5.48266489343992	-3.91175886505275
F	-6.22370676060989	-2.60083251002512	0.88610407252515
F	-4.97185736913191	-4.28285745029162	1.47437540705569
F	-5.00565316830235	-3.62203719322299	-0.59506847133409

Table S39. Cartesian coordinates for DFT-optimized structure of the square-planar intermediate **[Ni^{II}H(HS-*p*-CF$_3$-Ph)(S-*p*-CF$_3$-Ph)$_2$]$^-$** (^{1}**1^{R2}NiH,SH**, S = 0, B3LYP, def2-TZVPP, gas-phase).

Ni	-0.23128697234424	1.33886358430726	-1.28042606020033
S	-1.34557583460609	0.43346371429762	-2.94879831215596
S	1.35901777152821	2.41130642098100	-0.23769311075370
S	-1.71736303630478	1.03489759192751	0.41095358105413
H	0.63305180657030	1.68171328730997	-2.43711776713863
H	-1.64498716008667	1.54528872131936	-3.65026078538394
C	-6.95633065845538	-1.35290844941597	-1.45567350642847
C	-4.65485785176874	-1.82075289958650	-2.35189174521784
C	-5.57616737568292	-0.89990046134165	-1.84255363154721
C	-3.38389920023974	-1.38377104996697	-2.72581343417207
C	-5.23008544232088	0.45095914249413	-1.71093379036600
C	-3.03364928549038	-0.03780298526871	-2.58700584909112
C	-3.95462703861792	0.88238289188115	-2.06927542154490
C	-1.30056058221884	-1.64342381406168	-0.16457997858335
C	-1.69256878674146	-2.97553718807690	-0.22439091881538
C	1.48516898683865	1.94070426951412	1.44968646306633
C	1.20431568869322	0.64357610477568	1.93313266577051
C	-2.14330617269104	-0.64603807965022	0.39226217794878
C	-2.94179004943754	-3.37260196272920	0.27560151799648
C	-3.39485107991348	-4.78504078576328	0.11779757449408
C	1.97641852963142	2.88885697577213	2.37603641421281
C	1.39755793460672	0.31496291006807	3.26977371155624
C	-3.38314343404457	-1.07914657715057	0.92800963700883
C	-3.77861654723001	-2.40743191130887	0.86299165555095
C	2.17777514757219	2.56180250227959	3.71296197677854
C	1.88378366052946	1.27067002235248	4.17633210106186
C	2.14970140036547	0.90124028159520	5.59826347398097
H	-4.91428669603010	-2.87654331944944	-2.43114211765866
H	-2.65134070217145	-2.09815264994421	-3.10398683349205
H	-5.94710277145422	1.15974443046386	-1.29332149290709
H	-3.65981138371328	1.92037806056279	-1.91726458752087
H	-0.32625818956383	-1.34612045306217	-0.55857550309917
H	-1.02417189324955	-3.71736223078961	-0.66798383357082
H	0.82744636131407	-0.10886950453759	1.24239490911703
H	2.19139960779188	3.90089747782100	2.02503890593864
H	1.16437665597113	-0.69414983246316	3.61630747086586
H	-4.04810979533626	-0.33460284613127	1.37033881573731
H	-4.75368690125458	-2.70361469669464	1.25530775833911
H	2.55203510441440	3.31671171260055	4.40826740313484
F	-7.84960021367448	-1.19925603647327	-2.47623889826729
F	-6.99878152184037	-2.65911456132396	-1.10915203841953
F	-7.45834937241486	-0.64913314500500	-0.41104122654879
F	-4.34405950728047	-5.14438179769018	1.01776486115144

F	-2.38036989193153	-5.68358274538084	0.23704172475115
F	-3.94685762345915	-5.03199692776559	-1.11658022595986
F	3.42438340595755	0.43823300151558	5.80101416430783
F	1.33139288736798	-0.08245327855928	6.05407971967554
F	2.00984802241657	1.95136708575178	6.45312238534373

Table S40. Cartesian coordinates for DFT-optimized structure of the tetrahedral transition state (**TS₁**, *S* = 1, B3LYP, def2-TZVPP, gas-phase).

Ni	1.67682032317899	-0.59290947098857	0.56739353954460
S	0.95378441358562	-2.16703586451521	-0.91170944469944
S	3.10884831803472	1.18907748299694	0.77318889994626
S	-0.28982693546527	0.53294303811634	1.44388917522053
H	1.64686660959953	-1.14985955146401	2.10892225542136
H	0.63945661776941	-0.45307524895710	2.15122631623663
C	-3.96540395437772	0.89971868612827	-2.73994537772466
C	0.45069997859809	4.40705057907440	-3.67830623932687
C	-2.84379737422400	-1.25407461239349	-2.04575788652535
C	-2.75195196850144	0.10766459030134	-2.36461832344336
C	-1.70639790353128	-1.94908694121991	-1.63516853694386
C	2.13149922961799	2.69068129206264	-2.89582825554549
C	-1.51446184936890	0.75973130852177	-2.27657060718811
C	1.11416867293909	3.60596140344490	-2.59712172227289
C	-0.45655677066899	-1.30358542273134	-1.53424781101741
C	-0.38344911004718	0.06210765320463	-1.87072513729380
C	2.74347695272662	1.96518039215686	-1.87232520386221
C	0.71817825575481	3.79447407287041	-1.26342513392553
C	2.35397775534306	2.13381599524909	-0.52935555211352
C	1.33667948528540	3.07272531179373	-0.24581782354369
C	-2.83449826853205	0.03239544148177	0.59747386735617
C	-3.97218246174504	-0.74823928651757	0.41135082192041
C	-1.69073233710926	-0.51950242913739	1.19706715944123
C	-3.98230400621438	-2.07868466763692	0.85059012883526
C	-5.20496400505103	-2.91734666868998	0.63841848764608
C	-1.69321447386264	-1.86574757971399	1.59234665200672
C	-2.84040364098837	-2.63690620878618	1.43378944688497
H	-3.80522472853113	-1.77063158242770	-2.10090518911312
H	2.45030667504668	2.53920714621720	-3.92794168911909
H	-1.78754941700211	-3.00000968063624	-1.35609768252466
H	-1.43420418510325	1.81744954197485	-2.52835824159028
H	3.53017972685192	1.24830804923309	-2.11381471248139
H	0.57094242759687	0.58397025612561	-1.80264292626441
H	-0.07371356862064	4.50664551524908	-1.01879332677126
H	1.01993645815972	3.22640234002047	0.78726542947574
H	-2.83020058199831	1.07480590043256	0.27462525762830
H	-4.86104707220108	-0.31121739865056	-0.05047627046058
H	-0.80489206019806	-2.30581169932077	2.04445391899989
H	-2.84654468277317	-3.67524581234178	1.77026889022306
F	-4.99931814392438	0.13083817515180	-3.16333375674137
F	0.83888836576655	4.03639360551058	-4.92070162021549
F	-4.45048518625771	1.63902754410891	-1.69886978980721
F	-0.90845375602646	4.30803628386048	-3.64665719133932

F	-3.70848119453904	1.79879751396532	-3.72786285811618
F	0.71375064233623	5.74050662208370	-3.57692216753625
F	-6.34602632169274	-2.18299592088934	0.66611736038593
F	-5.34467183136482	-3.89499708769201	1.56718181541165
F	-5.18230311827062	-3.54824260662666	-0.57345894507782

10. References:

(1) Confer, A. M.; Sabuncu, S.; Siegler, M. A.; Moënne-Loccoz, P.; Goldberg, D. P. *Inorg. Chem.,* **2019**, *58*, 9576.
(2) Namazian, M.; Lin, C. Y.; Coote, M. L. *J. Chem. Theory Comput.,* **2010**, *6*, 2721.
(3) Castro, L.; Bühl, M. *J. Chem. Theory Comput.,* **2014**, *10*, 243.
(4) Yan, L.; Lu, Y.; Li, X. *Phys. Chem. Chem. Phys.,* **2016**, *18*, 5529.
(5) Kim, H.; Park, J.; Lee, Y. S. *J. Comput. Chem.,* **2013**, *34*, 2233.
